# Genome-wide characterization of sulphur metabolism gene families and recombination dynamics in mangrove-derived Bacillus aryabhattai NM1-A2 and Bacillus cereus NR1

**DOI:** 10.1099/mgen.0.001713

**Published:** 2026-05-22

**Authors:** Muhammad Kashif, Tingmei Li, Dan Wang, Can Meng, Yujia Luo, Qi Liang, Feng Guo, Saif Ur Rehman, Sheng He, Chengjian Jiang

**Affiliations:** 1 Guangxi Key Laboratory for Green Processing of Sugar Resources, Guangxi Technology Innovation Center of Liuzhou Luosi Rice Noodle, Innovation Research Center for Medical-Engineering Integration, College of Biological and Chemical Engineering, Guangxi University of Science and Technology, Liuzhou 545006, PR China; 2 Guangxi Technology Innovation Center for Microbial Resources Development and Utilization, College of Life Science and Technology, Guangxi University, Nanning 530004, PR China; 3 National Engineering Research Center for Non-Food Biorefinery, Guangxi Research Center for Biological Science and Technology, Guangxi Academy of Sciences, Nanning 530007, PR China; 4 Department of Public Health, Rawalpindi Medical University, Rawalpindi 46000, Pakistan; 5 Guangxi Birth Defects Prevention and Control Institute, Maternal and Child Health Hospital of Guangxi Zhuang Autonomous Region, Nanning 530033, PR China

**Keywords:** *Bacillus aryabhattai* NM1-A2, *Bacillus cereus* NR1, genome-wide analysis, marine mangrove habitats, Multiple Expectation Maximization for Motif Elicitation (MEME) motifs, recombination events, sulphur metabolism

## Abstract

This study presents a comprehensive genome-wide analysis of sulphur metabolism-related gene families in *Bacillus aryabhattai* strain NM1-A2 and *Bacillus cereus* strain NR1, isolated from marine mangrove habitats. We investigated phylogenetic relationships, conserved motifs, recombination events and physicochemical properties of sulphur metabolism genes. Phylogenetic analysis identified 4 major clades (35 genes in NM1-A2 and 34 in NR1), highlighting significant evolutionary relationships. Multiple Expectation Maximization for Motif Elicitation analysis revealed ten conserved motifs, including domains associated with cysteine/methionine metabolism and sulfurtransferases, validated by Pfam and CDD databases. Recombination analysis detected 87 and 64 putative recombination events in NM1-A2 and NR1, respectively, with significant PHI test results (*P*<0.00001), suggesting distinct parental contributions. Physicochemical characterization indicated that sulphur metabolism proteins in both strains exhibit an acidic nature, instability and hydrophobicity, with minimal thermostability. Eleven gene pairs in NM1-A2 and nine in NR1 were identified under purifying selection. Recombination breakpoints were detected at site 682 in NM1-A2 and site 2397 in NR1 using Genetic Algorithm Recombination Detection. Secondary structure analysis showed disorder percentages of 0–19% in NM1-A2 and 0–16% in NR1, with alpha-helical and beta-sheet and TM helix composition variations. These findings provide new insights into the evolutionary dynamics, functional diversity and structural adaptations of sulphur metabolism genes in marine *Bacillus* strains, enhancing our understanding of their ecological roles in mangrove ecosystems.

## Graphical Abstract

Mangrove-isolated *Bacillus aryabhattai* NM1-A2 and *Bacillus cereus* NR1 genome-wide analyses reveal conserved motif, recombination-driven evolution and purifying selection shaping structurally adaptive sulphur metabolism genes driving functional diversification and biogeochemical transformation.

**Impact Statement**

This study offers a genomic dissection of sulphur metabolism-related gene family in *Bacillus aryabhattai* NM1-A2 and *Bacillus cereus* NR1, uncovering fundamental evolutionary diversity and structural determinants that shape microbial sulphur cycling in mangrove ecosystems. Using integrated phylogenomics, conserved motif architecture, recombination dynamics and comprehensive physicochemical and structural features, we reveal that lineage-specific recombinant patterns, purifying selection signatures and distinct structural disorder patterns provide unprecedented insight into the adaptive strategies that enable *Bacillus* species to function as critical sulphur transformers in complex coastal environments. These findings advance our mechanistic understanding of microbial sulphur metabolism and offer a robust genomic framework for future ecological, evolutionary and biotechnological exploration of mangrove-associated bacteria.

## Data Summary

1. The datasets presented in this study for *Bacillus cereus* strain NR1 can be found in online repositories under the accession number of https://www.ncbi.nlm.nih.gov/genbank/, CP090421–CP090422, and https://www.ncbi.nlm.nih.gov/, SRR17081772–SRR17081783 [1].
2. The datasets of *Bacillus aryabhattai* strain NM1-A2 were deposited in the NCBI GenBank and NCBI SRA under the accession numbers CP083269, CP083270, CP083271, CP083272 and PRJNA760884, respectively [2].
3. All supporting figures and tables are provided in supplementary materials.

## Introduction

Microbial communities in marine mangrove habitats are essential to energy metabolism and biogeochemical cycling [3]. *Bacillus* strains exhibit diverse metabolic capabilities, particularly in sulphur metabolism, which is crucial for maintaining ecological balance [4]. Understanding the genetic mechanisms underlying sulphur metabolism in these bacteria is vital for deciphering their role in elemental cycling and environmental adaptation [2].

Advancements in genomic technologies have enabled comprehensive genome-wide analyses of sulphur metabolism genes in *Bacillus* species [5]. Researchers can identify key genes involved in sulphur transformation processes through high-throughput sequencing and annotation [1]. Such technological progress has facilitated recent studies to unravel the genetic foundations of sulphur metabolism and its evolutionary consequences [6]. Recent studies on *Bacillus subtilis* have revealed extensive genetic diversity in sulphur metabolism pathways, providing insights into their evolutionary adaptation to diverse environments [7]. This genomic exploration enhances our understanding of microbial biochemistry and ecosystem functioning [8].

Sulphur metabolism plays a pivotal role in microbial ecology by driving nutrient cycles and sustaining ecosystem health [9]. In *Bacillus* species, this metabolic process includes sulphate reduction to sulphide, followed by oxidation back to sulphate [9]. These biochemical transformations are essential for energy production and detoxification. Recent research highlights the role of sulphur metabolism in maintaining redox balance, stabilizing microbial communities and supporting biogeochemical cycles in mangrove ecosystems [10]. Additionally, sulphur metabolism regulates sulphur compound concentrations [11], fostering microbial interactions that enhance ecosystem stability and productivity [12].

Phylogenetic analysis of sulphur metabolism genes provides valuable insights into their evolutionary origins and relationships among various *Bacillus* strains [13]. Comparative studies using multiple sequence alignments and phylogenetic tree construction help identify conserved gene families and evolutionary trends [3]. MEME (Multiple Expectation Maximization for Motif Elicitation) motif analysis further detects conserved sequence motifs [13] critical for gene regulation and functional specialization [14]. These motifs often correspond to functional domains or regulatory elements crucial for sulphur metabolism, helping elucidate the roles and mechanisms of these genes in different *Bacillus* species [15]. These analytical tools collectively offer a deeper understanding of sulphur metabolism and the genetic and functional evolution in *Bacillus* strains [16].

Investigating conserved domain regions within sulphur metabolism genes is key to understanding their functional roles and evolutionary conservation [17]. Domain analysis identifies essential functional elements, while gene duplication studies reveal adaptive strategies enabling *Bacillus* strains to thrive in diverse ecological niches [18]. Genetic recombination also plays a significant role in microbial evolution, facilitating genetic exchange and trait acquisition [18]. The Genetic Algorithm Recombination Detection (GARD) tool is instrumental in identifying recombination events [19], shedding light on the mechanisms driving genetic variation and evolutionary adaptation [20].

Predicting protein structures is critical for elucidating the functional roles of sulphur metabolism-related proteins [21]. Structural modelling helps determine protein conformations and interactions [22], offering insights into their biochemical functions [23]. Additionally, analysing the cellular localization of sulphur metabolism genes provides valuable information on their spatial organization and functional integration within *Bacillus* cells [24]. Combining structural predictions with cellular localization studies [25] enables a comprehensive understanding of the functional dynamics of sulphur metabolism [26].

*Bacillus aryabhattai* and *Bacillus cereus* are two bacterial strains from marine mangrove habitats, which represent unique coastal ecosystems characterized by high salinity, fluctuating oxygen availability and sulphur-rich sediments [1,2]. These ecosystems are biodiversity hotspots, where microbial communities are pivotal for nutrient cycling, organic matter decomposition and overall ecosystem stability [27]. Members of the genus *Bacillus* are renowned for their metabolic versatility and environmental robustness, traits that enable them to survive and function under diverse and often extreme conditions [28]. *B. aryabhattai* exhibits exceptional tolerance to salinity, oxidative stress and nutrient variability and actively participates in sulphur transformations and other critical biogeochemical processes [29]. In contrast, *B. cereus* is distinguished by its extensive metabolic repertoire and the production of diverse enzymes that facilitate nutrient turnover and environmental detoxification [30]. The remarkable ecological adaptation of these strains underscores their probable key roles in sulphur cycling, redox homeostasis and microbial community dynamics within mangrove sediments [31]. Collectively, these attributes position them as exemplary model organisms for the systematic investigation of sulphur metabolism-related gene families, providing a foundation for understanding their functional diversity, evolutionary trajectories and ecological significance.

This study performed a genome-wide analysis of sulphur metabolism-related gene families on NM1-A2 and NR1 from the mangrove habitat, examining their phylogeny, conserved motifs, recombination events, physicochemical traits and structural features. This study provides novel insights into the evolutionary dynamics, functional diversity and structural adaptation of sulphur metabolism genes in marine *Bacillus* strains, thereby advancing our understanding of their ecological significance in mangrove ecosystems.

## Methods

### Bacterial collection, culture conditions and molecular identification

NM1-A2 and NR1 were isolated from subtropical mangrove sediments in the Beibu Gulf, South China Sea (21° 29′ 25.74″ N 109° 45′ 49.43″ E), and deposited in CCTCC (M20211548) and CGMCC (23142), respectively. Sulphur oxidizing bacteria (SOB) medium was prepared per litre with 0.5 g BaCl₂, 0.2 g NaHCO₃, 2 g KH₂PO₄, 2 g Na₂S₂O₃, 0.2 g Ca(NO₃)₂, 1 g yeast extract agar, 1 g sulphur powder and 15–20 g agar. Soil samples (1 g) were mixed with 50 ml sterile water and serially diluted (10⁰−10^−2^), and 100 µl of each dilution was plated on SOB plates and incubated for 3–4 days, and colony growth was recorded. Pure colonies were subcultured in SOB broth, followed by Luria-Bertani (LB) medium incubation (100 ml, 24 h, 37 °C). DNA was extracted from 1 ml of culture using standard protocols. The reaction conditions for amplification of the 16S rRNA gene with primers 27F (5′-GAGTTTGATCCTGGCTCAG-3′) and 1492R (5′-GGTTACCTTGTTACGACTT-3′) were as follows: 95 °C for 5 min; [95 °C for 45 s; 56 °C for 45 s and 72 °C for 1 min] ×30 cycles; 72 °C for 10 min. The TIANamp bacterial DNA kit [TIANGEN Biotech (Beijing) Co., Ltd., China] was used to purify the PCR products. The purified PCR product of the 16S rRNA gene was sequenced using Sanger sequencing (BGI: https://www.bgi.com/). Our previous study provides details [1,2].

### Phylogenetic and multiple sequence alignment analysis

Phylogenetic analysis was performed at the functional gene level to investigate the evolutionary relationships of sulphur metabolism-related genes in *B. aryabhattai* NM1-A2 and *B. cereus* NR1 [1,2]. Representative genes involved in sulphate transport, activation, reduction and downstream sulphur compound transformations were selected from the annotated genomes of both strains based on KEGG, COG and NR database annotations [1,2]. The corresponding protein sequences were used for phylogenetic reconstruction. Phylogenetic trees were constructed in mega 11 using the neighbour-joining method with 1,000 bootstrap replicates to assess branch support [32], and the phylogeny results were visualized and examined using iTOL v4.2.3 [33]. To identify insertion–deletion events (indels) and visualize sequence variation among sulphur metabolism-related proteins, multiple sequence alignments were performed using the Multiple Align Show tool (https://www.bioinformatics.org/sms/multialign.html) [34]. This gene-centric approach enabled a focused assessment of the evolutionary relationships and sequence variation of sulphur metabolism genes in the two mangrove-derived *Bacillus* strains [1,2].

### MEME motif and conserved domain elucidation

Moreover, the conserved protein domains in the sulphur-metabolizing gene from NM1-A2 and NR1 were blasted against the NCBI conserved domain database (CDD). Additionally, ten MEME motifs of conserved protein motifs for NM1-A2 and NR1 sulphur-metabolizing genes were analysed by using the MEME programs [35,36].

### Characterization of bacterial strains

The ProtParam tool (https://web.expasy.org/protparam/) (accessed on 18 December 2023) was used to analyse the physicochemical features of NM1-A2 and NR1 proteins, including the number of aa, instability index (II), molecular weight (MW), isoelectric point (pI), aliphatic index (AI) and grand average of hydropathicity (GRAVY) [37].

### Duplication analysis of bacterial strains

Furthermore, the ratio of nonsynonymous (Ka) to synonymous (Ks) nucleotide substitution rates was calculated to measure selective pressure, and pairwise combinations of genes were also determined using the TB tool [38].

### The subcellular localization of bacterial strains

Subcellular localization of the bacterial strains' proteins was predicted using Cello-life web servers (https://cello.life.nctu.edu.tw/) [39].

### Recombination analysis

For recombination analysis, the whole-genome nucleotide sequences of related bacterial strains were aligned using MAFFT, including BA_K13, BM_DSM319 and BM_QMB1551 for NM1-A2, and BC_E33L, BA_AmesAncestor and BA_Sterne for NR1. Potential recombination events were detected using RDP5, implementing multiple algorithms such as MaxChi, SiScan and Chimaera. The statistical significance of detected recombination events was confirmed using the PHI test across both closely and distantly related genomes. Additionally, major and minor parental sequences, as well as recombination breakpoints, were identified using a combination of recombination detection program (RDP), MaxChi, 3Seq, BootScan and Chimaera methods [40].

### Analysis of recombination breakpoints in the bacterial strains

The GARD tool was used to detect recombination breakpoints in multiple sequence-aligned bacterial strains [15].

### Prediction of secondary structures and structural analysis in three dimensions

Phyre2 was employed to forecast the secondary structure of the bacterial strains, along with assessing the functional traits of the resultant protein. Properties like alpha helix and beta strand were determined, as well as disorder [16]. Moreover, the amino acid sequences of all selected strains were submitted to Phyre2 (http://www.sbg.bio.ic.ac.uk/phyre2) to design the three-dimensional structure of each bacterial strain protein [41].

## Results

### Phylogenetic and multiple sequence alignment analysis

**Fig. 1.**
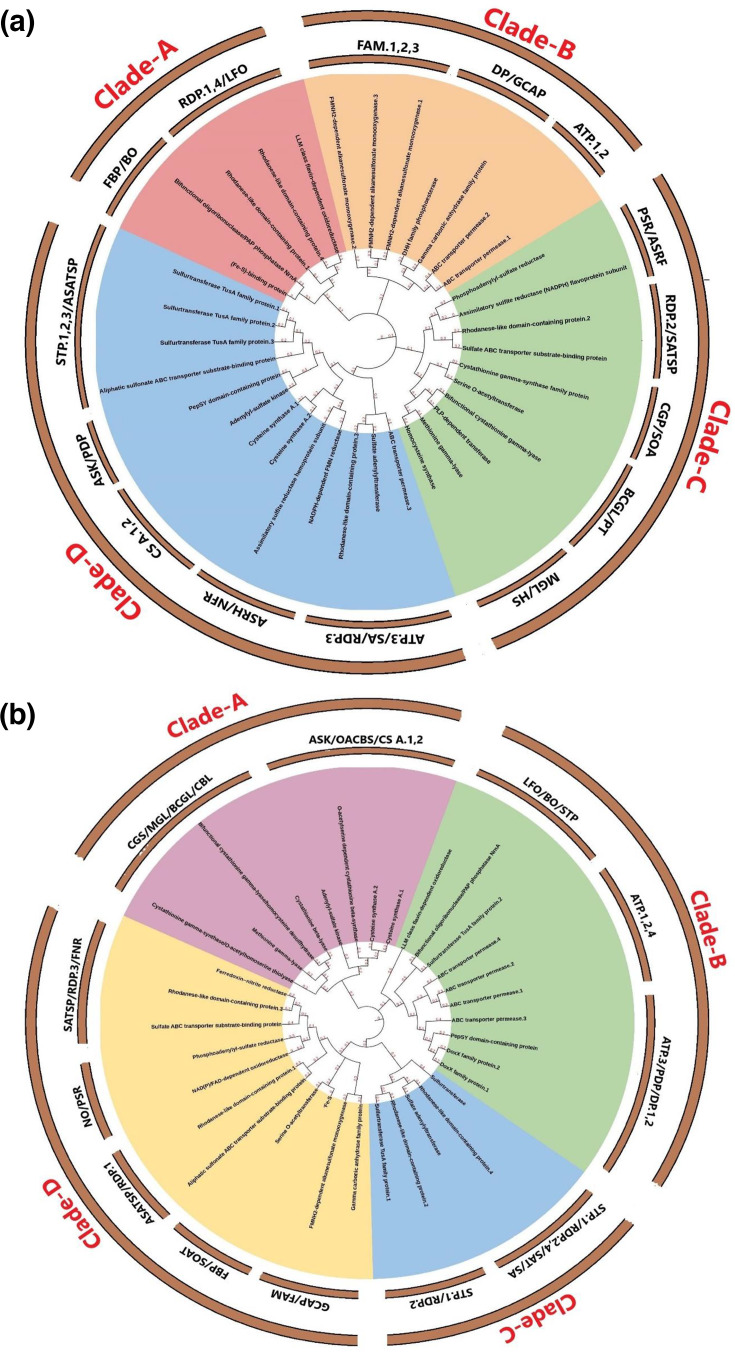
Molecular phylogenetic relationships of sulphur-metabolizing genes in (**a**) NM1-A2 and (**b**) NR1 bacterial strains. The outer circle represents the major phylogenetic clades (A–D), illustrating the evolutionary grouping of sulphur-metabolizing genes, while the inner circle indicates the functional category of each gene.

A molecular phylogenetic analysis of bacterial strains (NM1-A2 and NR1) harbouring the sulphur-metabolizing gene was constructed using a maximum likelihood method. This analysis used bootstrap consensus values for every node to derive their evolutionary history. Both the NM1-A2 and NR1 sulphur-metabolizing genes were used to generate a set of 69 amino acid sequences (35 genes of NM1-A2 and 34 genes of NR1), which were then divided into four major clades (Clade-A, Clade-B, Clade-C and Clade-D) by the degree of homology across gene sequences separately for each strain (Fig. 1a, b). The clade A of NM1-A2 has five genes, while NR1 has eight genes, respectively. Similarly, the clades B, C and D of NM1-A2 and NR1 have the genes 7 vs. 10, 10 vs. 5 and 13 vs. 11, respectively (Fig. 1a, b and Section S2, available in the online Supplementary Material).

### Phylogenetic relationship, MEME motif and conserved domain regions

**Fig. 2.**
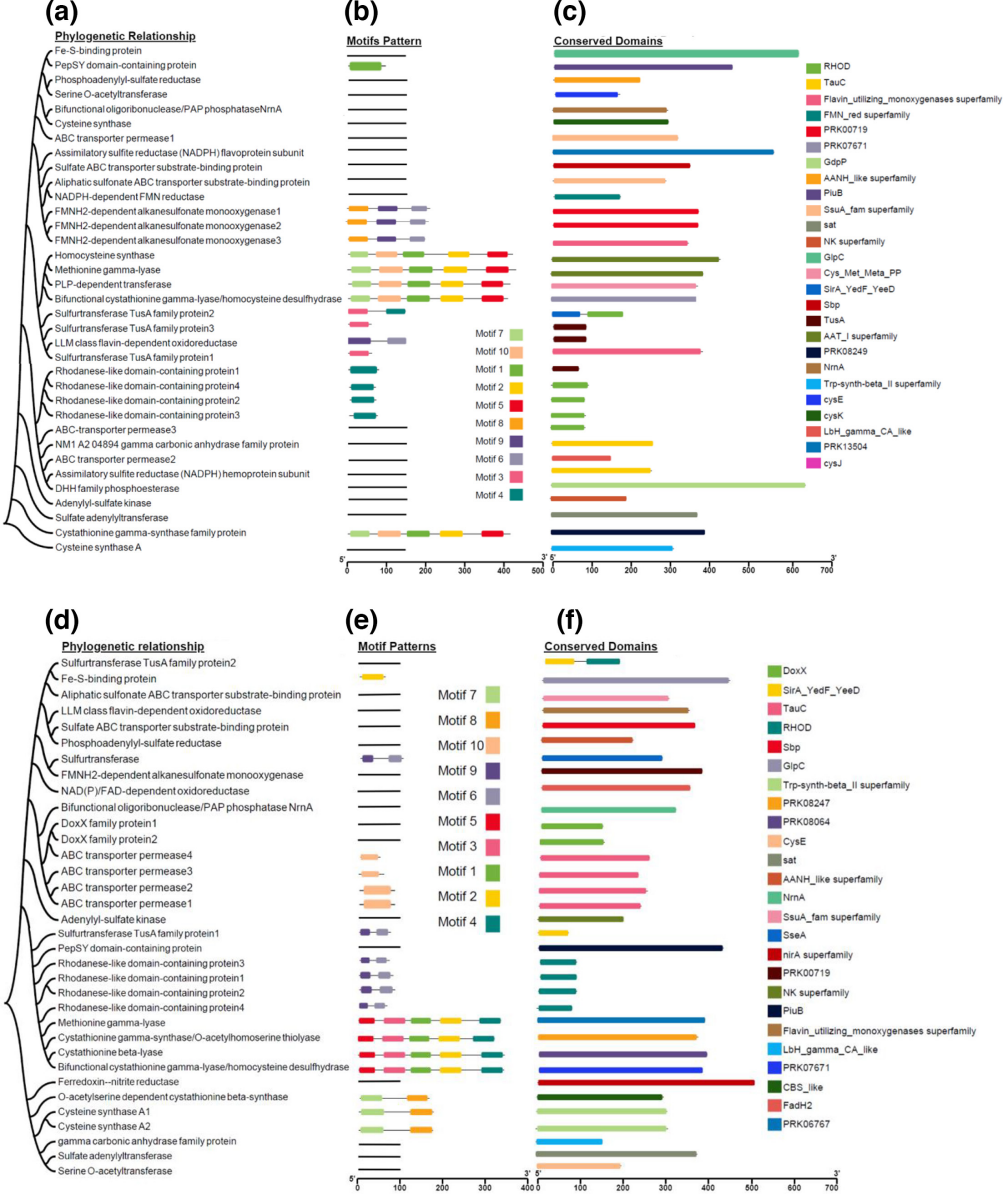
(**a**) Phylogenetic relationship, MEME motif and conserved domain regions of NM1-A2 sulphur-metabolizing gene. (**b**) Motif pattern of NM1-A2 sulphur-metabolizing gene. (**c**) Conserved domain regions of NM1-A2 sulphur-metabolizing gene. (**d**) Phylogenetic relationship of NR1 sulphur-metabolizing gene. (**e**) Motif pattern of NR1 sulphur-metabolizing gene. (**f**) Conserved domain regions of NR1 sulphur-metabolizing gene.

For the sulphur metabolism-related genes of both NM1-A2 and NR1, we examined the organization of the motifs and conserved domains along with their phylogenetic relationships (Fig. 2a, d). In both sulphur-metabolizing bacterial strains, ten MEME-conserved motifs were recognized (Fig. 2). The NM1-A2 MEME motifs 1, 2, 5, 7 and 10, having amino acids 50, 50, 48, 42 and 50, respectively, were noted as Cys/Met metabolism PLP-dependent enzyme domain, where MEME motifs 6, 8 and 9, consistent with 40, 50 and 50 amino acids, were recognized as Bac luciferase. In contrast, MEME motif 3, corresponding to 50 amino acids, had the domain of sulfurtransferase TusA, and MEME motif 4, with a width of 40 amino acids, was termed the domain of rhodanese in Pfam search (Table 1). The CDD NCBI searches were employed to verify the perceived domains (Fig. 2c). Moreover, RHOD, TauC, flavin-utilizing monooxygenase superfamily, FMN red superfamily, PRK00719, PRK07671, GdpP, AANH-like superfamily, PiuB, SsuA fam superfamily, sat, NK superfamily, GlpC, Cys Met Meta PP, SirA YedF YeeD, Sbp, TusA, AAT I superfamily, PRK08249, NrnA Trp-synth-betaII superfamily, CysE, CysK, LbH gamma CA-like, PRK13504 and CysJ domain have also been scoured up in NM1-A2 sulphur gene (Fig. 2c). Moreover, the MEME searches identified the conserved motifs only in 17 sulphur genes of the NM1-A2 bacterial strain (Fig. 2b and Section S2, Table 1).

**Table 1.** Ten conserved MEME motifs detected in NM1-A2 strain

MEME motif	Amino acid sequence	Length	Pfam domain
MEME-1	AKQHGJLVIVDNTFSTPYLQRPJELGADIVIHSATKFJGGHGDVVAGVVV	50	Cys_Met_Meta_PP
MEME-2	PFDSWLLLRGIKTLALRMERHEENALKIAKFLEAHEAVEKVYYPGLETHP	50	Cys_Met_Meta_PP
MEME-3	DAKGLACPMPIVKTKKAMNELEPGQVLEIQATDKGAKSDLTAWAKSTGHE	50	TusA
MEME-4	HJVDVREPEEFETGHIPGARNIPLSEJPARINELDKEKEVIIICQSGRR	49	Rhodanese
MEME-5	AESLGAVESLITHPASTTHAEIPEEERKELGITNGLIRJSVGJEDVED	48	Cys_Met_Meta_PP
MEME-6	YPPVQKPYPPLYFGGSSPAGQEVAAKHADVYLTWGEPPAQ	40	Bac luciferase
MEME-7	TGYVYSRTANPTVTALEERIAELEEGEAALAFASGMAAVSAV	42	Cys_Met_Meta_PP
MEME-8	WFIPTYGDGRYLGTNEGARSADYSYFKQVAQAADRLGYDGVLLPTGKSCE	50	Bac luciferase
MEME-9	PGIMSPTLAARMASTFDRISDGRLLINVVAGGDPVELAGDGVFLSHDERY	50	Bac luciferase
MEME-10	YGGTYRVLTEILPKIGITVSFVNTENLEEIEAAIRPNTKAIYIETPTNPT	50	Cys_Met_Meta_PP

* [Cys_Met_Meta_PP; Cys/Met metabolism PLP-dependent enzyme, TusA; sulfurtransferase TusA].

Furthermore, in NR1, the MEME motifs 1, 2, 3, 4 and 5, having amino acids 47, 50, 50, 50 and 37, were elucidated as Cys/Met metabolism PLP-dependent enzyme domain. In contrast, the 7 and 8, consisting of 50 and 48 amino acids, were recognized as PALP. Moreover, the MEME motifs 9 and 10 detected the rhodanese and BPD_transp 1 domain with a number of amino acids 21 and 41, respectively. All these conserved domains were searched in Pfam (Table 2), and the CDD NCBI searches were also utilized to verify the identified domains (Fig. 2f). Moreover, DoxX, SirA YedF YeeD, TauC, RHOD, Sbp, GlpC, Trp synth beta II superfamily, PRK08247, PRK08064, CysE, sat, AANH-like superfamily, NrnA, SsuA fam superfamily, SseA.nirA superfamily, PRK00719, NK superfamily, PiuB, flavin-utilizing monooxygenase superfamily, LbH gamma CA-like, PRK07671 and CBS-like FadH2 PRK06767 domain have also been scoured up in NR1 sulphur gene (Fig. 2f). Besides, the MEME searches identified that out of 35 sulphur genes of NR1, only 18 sulphur genes of the NR1 bacterial strain have the conserved motif (Fig. 2e) (Fig. 2, Table 2 and Section S2).

**Table 2.** Ten conserved motifs were detected in NR1 strain

MEME motif	Amino acid sequence	Length	Pfam domain
MEME-1	AKRKGLLTIVDNTFCTPYWQRPLELGADIVLHSATKYIGGHSDVVAG	47	Cys_Met_Meta_PP
MEME-2	GGILGPQDCWLLLRGLKTLAVRMEQHCDNARKIVHYLNDHDKVNNVYYPG	50	Cys_Met_Meta_PP
MEME-3	YGGTYRVITEVENRFGIRHTFVDMTNLEEIEQAIRPNTKAIYVETPTNPL	50	Cys_Met_Meta_PP
MEME-4	SLGAVESLISYPATMTHAAIPQERRKEMGICDSLIRLSVGIENWNDLISD	50	Cys_Met_Meta_PP
MEME-5	YDYSRTGNPTREALEEMIAVLEGGHQGFAFGSGMAAI	37	Cys_Met_Meta_PP
MEME-6	EYYVICRSGMRSDRACQYLKEQGFQ	25	–
MEME-7	YVKLEFFNPGRSVKDRIAYNLIEDAEEKGLIKQGDTIIEPTSGNTGIGLA	50	PALP
MEME-8	PNSFIPQQFENQANPRIHRYTTGPEIWEQMDGELDAFVAGAGTGGTIT	48	PALP
MEME-9	DVREVEEYAAGHIPEACNIPL	21	Rhodanese
MEME-10	DPLLVMSQTIPIVALAPLFVLWFGYGMWSKVMVVVLICFFP	41	BPD transp1

### Characterization of physicochemical properties of NM1-A2 and NR1 sulphur-metabolizing genes

Physicochemical attributes of the NM1-A2 and NR1 sulphur-metabolizing gene were analysed for protein length, number of aa in each peptide, MW (Da), pI, II, AI and GRAVY Index, as presented in Tables 3 and 4. The MW of NM1-A2 proteins ranged from 18,306.27 to 161,764.12 Da, and the values of pI ranged from 4.98 to 5.38. All the proteins show an acidic nature (Table 3), and the AI values were found to be <65, which shows no thermostable characteristics of all the NM1-A2 sulphur-metabolizing proteins. According to II, all the proteins appeared stable except homocysteine synthase, PLP-dependent transferase, sulphate ABC transporter substrate-binding protein, LLM class flavin-dependent oxidoreductase, PepSY domain-containing protein, bifunctional cystathionine gamma-lyase, gamma carbonic anhydrase family protein and ABC transporter permease 3, which exhibited thermostable characteristics, as values were >40 (Table 3). Similarly, all the NM1-A2 sulphur-metabolizing proteins appeared to be hydrophobic in nature (Table 3 and Section S3).

**Table 3.** Physicochemical properties of NM1-A2 sulphur-metabolizing genes

Genes	AA	MW	PI	II	AI	Gravy
Cysteine synthase A.1	939	75,991.35	5.12	36.55	33.97	0.806
Serine O-acetyltransferase	660	53,517.14	5.2	30.15	33.03	0.75
Homocysteine synthase	1,308	109,660.93	5.02	47.58	28.98	0.788
FMNH2-dependent alkanesulfonate monooxygenase.1	1,134	91,805.41	5.08	33.29	29.45	0.703
ABC transporter permease.1	744	62,296.4	5.18	33.38	24.6	0.536
PLP-dependent transferase	1,146	94,567.06	5.08	41	30.98	0.749
Methionine gamma-lyase	1,188	97,345.71	5.08	38.26	31.73	0.73
Cystathionine gamma-synthase family protein	1,215	98,658.35	5.08	37.44	34.57	0.795
Sulphate ABC transporter substrate-binding protein	1,065	86,090.31	5.11	41.15	36.06	0.809
LLM class flavin-dependent oxidoreductase	1,071	89,121.21	5.05	41.71	29.41	0.832
FMNH2-dependent alkanesulfonate monooxygenase.2	1,071	87,193.79	5.09	39.42	30.91	0.759
NADPH-hemoprotein subunit	1,722	140,560.13	4.99	38.88	31.94	0.774
NADPH-flavoprotein subunit	1,812	147,966.59	4.98	40.53	32.17	0.79
PepSY domain-containing protein	1,380	114,479.47	5.02	41.05	27.75	0.722
Sulfurtransferase TusA family protein.1	225	17,844.71	5.38	35.11	40	0.867
Sulfurtransferase TusA family protein.2	552	44,230.74	5.25	30.11	38.04	0.805
Rhodanese-like domain-containing protein.1	297	23,683.03	5.33	28.38	35.35	0.755
Rhodanese-like domain-containing protein.2	357	29,095.13	5.31	24.12	36.13	0.776
Sulfurtransferase TusA family protein.3	228	18,306.27	5.38	34.93	41.67	0.892
Rhodanese-like domain-containing protein.3	384	31,829.66	5.26	37.11	32.81	0.847
Bifunctional cystathionine gamma-lyase/homocysteine desulfhydrase	1,134	92,582.62	5.09	42.49	42.49	0.765
FMNH2-dependent alkanesulfonate monooxygenase.3	1,125	91,175.57	5.09	35.55	29.6	0.69
NADPH-dependent FMN reductase	555	45,721.72	5.22	37.44	34.05	0.777
ABC transporter permease.2	855	71,750.14	5.14	34.99	26.43	0.599
Aliphatic sulfonate ABC transporter substrate-binding protein	987	80,079.39	5.14	32.59	36.58	0.784
Bifunctional oligoribonuclease/PAP phosphatase NrnA	939	76,984.23	5.12	34.42	31.52	0.732
Cysteine synthase A.2	933	76,618.2	5.11	40.62	31.73	0.774
Rhodanese-like domain-containing protein.4	312	24,917.23	5.36	23.12	37.5	0.728
Gamma carbonic anhydrase family protein	528	43,862.98	5.21	41.37	31.25	0.792
ABC transporter permease.3	807	68,983.83	5.02	47.5	47.5	0.666
Adenylyl-sulphate kinase	600	49,016.1	5.21	31.38	33.83	0.739
Sulphate adenylyltransferase	1,152	94,142.61	5.08	39.97	34.64	0.81
Phosphoadenylyl-sulphate reductase	714	58,456.62	5.18	30.99	32.63	0.734
(Fe-S)-binding protein	2,112	172,768	4.98	28.67	30.82	0.664
DHH family phosphoesterase	1,974	161,764.12	5	33.28	31.86	0.686

**Table 4.** Physicochemical properties of NR1 sulphur-metabolizing genes

Genes	AA	MW	PI	II	AI	Gravy
Cysteine synthase A.1	924	74,127.29	5.16	28.22	34.42	0.712
Serine O-acetyltransferase	666	54,407.4	5.23	31.38	31.98	0.615
DoxX family protein.1	507	42,626.09	5.24	49.56	28.8	0.641
ABC transporter permease.1	774	64,497.08	5.18	34.34	31.01	0.66
ABC transporter permease.2	753	62,801.66	5.2	29.97	28.29	0.548
Sulfurtransferase TusA family protein.1	561	44,555.37	5.28	14.93	33.69	0.628
Sulfurtransferase TusA family protein.2	231	18,056.56	5.41	15.45	36.8	0.677
Rhodanese-like domain-containing protein.1	360	28,775.24	5.36	17.86	37.22	0.665
Rhodanese-like domain-containing protein.2	296	23,303.01	5.15	11.21	37.16	0.588
Sulphate ABC transporter substrate-binding protein	1,056	84,618.89	5.14	31.54	37.12	0.758
Phosphoadenylyl-sulphate reductase	705	56,937.08	5.23	25.73	34.18	0.647
Sulphate adenylyltransferase	1,137	92,010.06	5.13	31.8	35.62	0.73
Adenylyl-sulphate kinase	594	47,836.09	5.27	22.85	34.18	0.641
Ferredoxin-nitrite reductase	1,623	131,544.96	5.07	29.14	34.69	0.682
Sulfurtransferase	834	67,535.97	5.18	22.99	33.09	0.671
Cysteine synthase A.2	918	74,356.11	5.14	33.63	34.75	0.767
LLM class flavin-dependent oxidoreductase	1,056	86,395.47	5.12	34.59	33.9	0.748
FMNH2-dependent alkanesulfonate monooxygenase	1,113	88,886.04	5.14	26.01	34.95	0.686
ABC transporter permease.3	849	69,950.94	5.2	29.42	29.56	0.519
Aliphatic sulfonate ABC transporter substrate-binding protein	987	78,987.55	5.17	22.47	38.91	0.767
NAD(P)/FAD-dependent oxidoreductase	1,200	98,235.94	5.12	36.27	36.25	0.724
Rhodanese-like domain-containing protein.3	384	31,899.65	5.26	46.83	35.16	0.849
Cystathionine gamma-synthase/O-acetylhomoserine thiolyase	1,113	91,869.21	5.08	46.43	32.7	0.784
Cystathionine beta-lyase	1,164	98,177.86	5.06	49.79	31.79	0.798
DoxX family protein.2	501	41,396.25	5.27	34.21	30.14	0.586
Bifunctional cystathionine gamma-lyase/homocysteine desulfhydrase	1,134	93,191.22	5.1	39.49	35.63	0.785
O-acetylserine dependent cystathionine beta-synthase	924	75,060.5	5.15	33.98	33.01	0.698
Bifunctional oligoribonuclease/PAP phosphatase NrnA	933	75,846	5.17	29.92	32.9	0.655
Methionine gamma-lyase	1,176	95,726.81	5.12	30.05	31.63	0.641
Rhodanese-like domain-containing protein.4	306	24,260.29	5.39	17.62	39.54	0.693
PepSY domain-containing protein	1,302	108,251.77	5.07	39.32	33.18	0.727
Gamma carbonic anhydrase family protein	513	42,221.81	5.24	30.37	33.33	0.764
ABC transporter permease.4	807	68,711.83	5.02	27.76	27.76	0.727
(Fe-S)-binding protein	2,112	172,786.48	4.98	34.56	31.25	0.692

The MW of NR1 proteins ranged from 18,056.56 to 172,786.48 Da, and the values of pI ranged from 4.98 to 5.41. All the proteins show an acidic nature (Table 4), and the AI values were found to be <65, which indicates no thermostable characteristics of all the NR1 sulphur-metabolizing proteins. According to II, all the proteins appeared stable except the DoxX family protein.1, rhodanese-like domain-containing protein.3, cystathionine gamma-synthase and cystathionine beta-lyase, which exhibited thermostable characteristics as values were >40 (Table 4). Similarly, all the NR1 sulphur-metabolizing proteins appeared to be hydrophobic in nature (Table 4 and Section S3).

### Duplication and cellular distribution analysis of NM1-A2 and NR1 sulphur-metabolizing gene

**Fig. 3.**
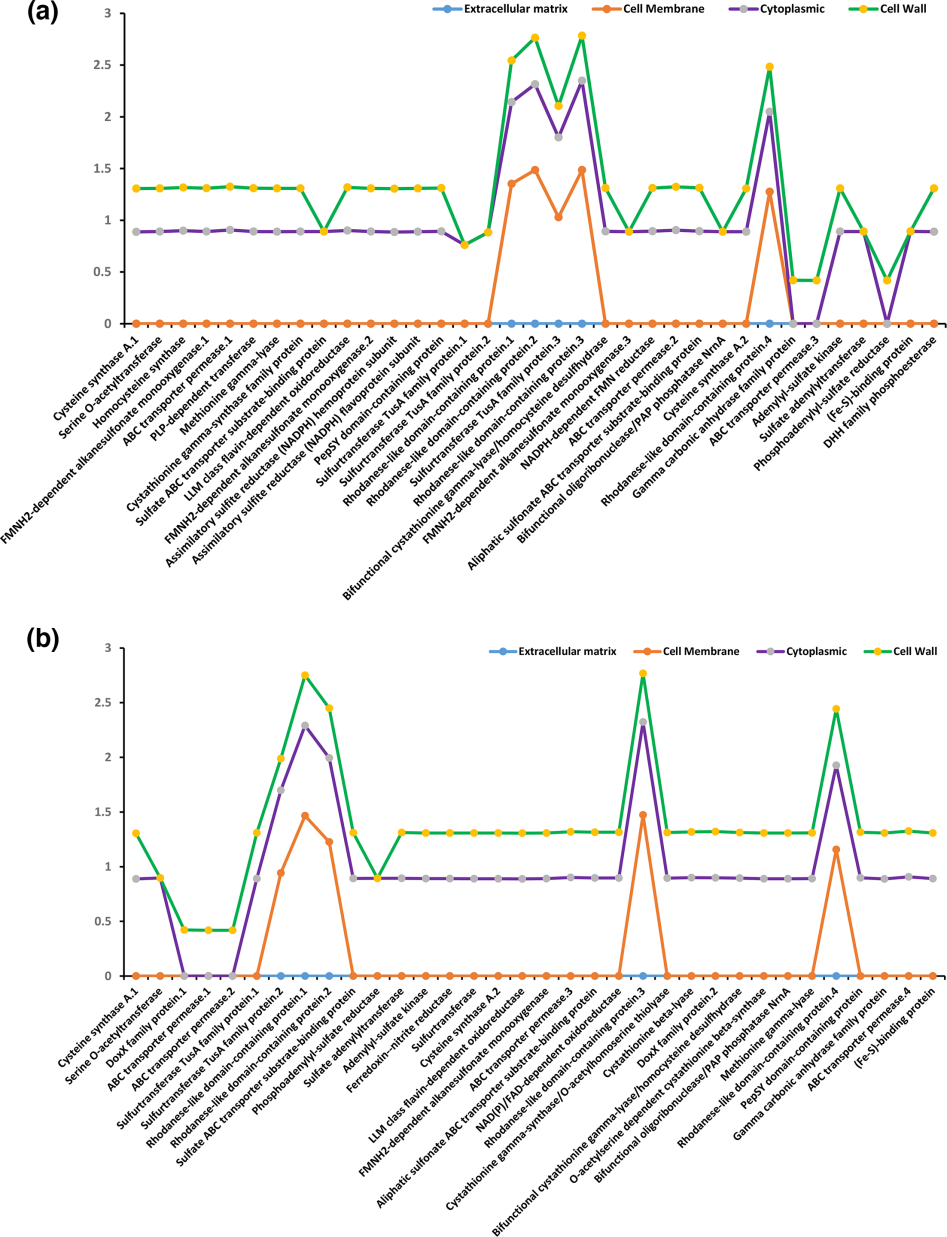
Cellular distribution of genes in (**a**) NM1-A2 and (**b**) NR1. Genes were classified into different cellular compartments, including cytoplasm, membrane and extracellular regions, based on subcellular localization prediction.

Additionally, for these homologous gene pairs, the ratio of nonsynonymous substitutions per nonsynonymous site (Ka) to synonymous substitutions per synonymous site (Ks) was examined (Table 5). A total of 20 homologous gene pairs were detected in NM1-A2 and NR1, where 11 gene pairs belong to NM1-A2 and 9 gene pairs to NR1, respectively (Table 5). Moreover, all the identified gene pairs had Ka/Ks ratios <1 except FNR/RDP.3 of NR1, demonstrating a purifying selection pressure (Table 5). Furthermore, all the NM1-A2 and NR1 sulphur-metabolizing genes’ cellular distribution illustrates that these genes are majorly expressed in the extracellular matrix, cell wall, cytoplasm and cell membrane (Fig. 3a, b and Table 5).

**Table 5.** Analysis of the Ka/Ks ratio for each duplicated gene pair of NM1-A2 and NR1 sulphur-metabolizing genes

Sr. no.	Gene pairs	Ka	Ks	Ka/Ks
**NM1-A2**				
1	PDP/ASK	1.056397877	2.233037351	0.473076671
2	ASRH/NFR	0.89875424	2.152140722	0.417609421
3	FAM.1/FAM.3	0.329433237	2.734842577	0.120457843
4	ATP.1/ATP.2	0.813998309	2.259822421	0.360204546
5	GCAP/DP	0.870224758	1.812278662	0.480182643
6	LFO/RDP.4	0.793185669	3.508616309	0.226067942
7	SATSP/RDP.2	0.854038436	2.584236858	0.330479938
8	SOA/CGP	0.84646412	2.844507186	0.297578478
9	HS/MGL	0.637174111	1.843816996	0.345573402
10	PT/BCGL	0.391434806	2.43963903	0.160447837
11	STP.1/STP.2	0.249390691	0.894921199	0.278673353
**NR1**				
1	FNR/RDP.3	0.809471396	0.774540146	1.045099
2	RDP.1/ASATSP	0.912760418	1.756744066	0.519575
3	FAM/GCAP	0.883947573	1.711786789	0.516389
4	STP.1/RDP.2	0.55498386	1.063671536	0.521762
5	SAT/RDP.4	0.716334018	2.560965301	0.279713
6	CSA.1/CSA.2	0.359016735	1.509012762	0.237915
7	CBL/BCGL	0.429812511	1.325614825	0.324236
8	ATP.1/ATP.2	0.53757238	1.73571085	0.309713
9	DP.1/DP.2	0.33211909	1.661019203	0.199949

### Recombination analysis

A total of 87 regions in whole-genome nucleotide sequences of NM1-A2 were perceived as putative regions of recombination, and our RDP analysis proposed that NM1-A2 could be a recombinant of BA_K13, BM_DSM319 and BM_QMB1551 (Section S1, Table S1). Significant evidence of recombination was also detected by the PHI test (*P*<0.00001). Moreover, our study showed that the BA_K13 shared the major parent genomic region of NM1-A2, while BM_DSM319 and BM_QMB1551 were determined as minor parents (Section S1, Table S1). Furthermore, all the detected recombinant events occurred towards the 5′ end of the NM1-A2 genome, as shown in the supplementary material (Section S1, Table S1).

From the complete genome sequence of NR1, a total of 64 regions were spotted as putative recombinant regions, and our RDP analysis anticipated that NR1 could be a recombinant of BC_E33L, BA_AmesAncestor and BA_Sterne (Section S1, Table S2). Moreover, the PHI test provided significant evidence of recombination (*P*-value<0.00001). Additionally, our study perceived BC_E33L as a major parental sharer for the genomic region of NR1, while BA_AmesAncestor and BA_Sterne as minor parents (Section S1, Table S2). Furthermore, all the recombinant events were dispersed in the whole genome of NR1, as illustrated in Section S1, Table S2.

**Fig. 4.**
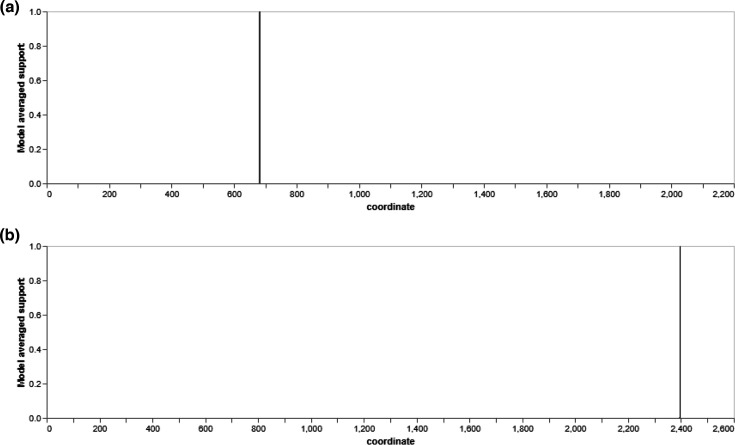
Model-averaged support for breakpoint placement in (**a**) NM1-A2 and (**b**) NR1 sulphur-metabolizing genes.

Additionally, we also performed recombination analysis using GARD and found evidence of recombination breakpoints by examining the 4,098 models in NM1-A2 and 3,041 in the NR1 sulphur-metabolizing gene. The model-averaged support for the breakpoint sites was calculated by quantifying the model-averaged frequency of seeing a breakpoint at a particular site across all points in the alignment. By performing multiple breakpoint analyses using a genetic algorithm, the multiple sequence alignment of 35 gene nucleotide sequences with 2,212 sites revealed 1 main recombination breakpoint at site 682 in NM1-A2 (Fig. 4a and Section S2), while the multiple sequence alignment of 34 gene nucleotide sequences with 2,624 sites revealed 1 main recombination breakpoint at site 2397 in NR1 (Fig. 4b and Section S2) (Fig. 4).

**Fig. 5.**
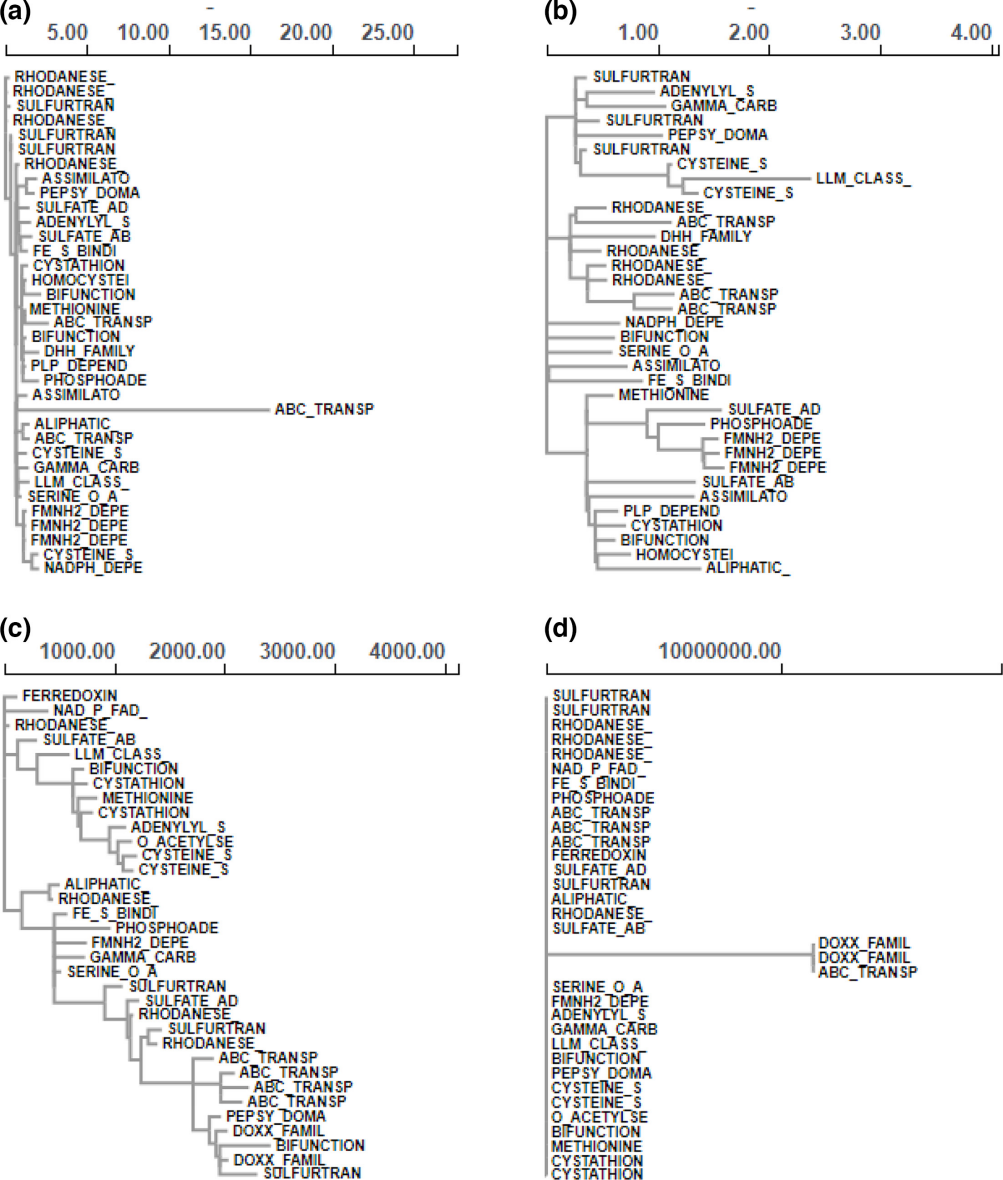
Trees for individual fragments in NM1-A2: (**a**) tree 1, coordinate range 1–682; (**b**) tree 2, coordinate range 682–2212; and the tree fragments for NR1: (**c**) tree 1, coordinate range 1–2397; (**d**) tree 2, coordinate range 2397–2624.

Analyses of fragmented sequences indicated by GARD-discovered recombination breakpoints revealed phylogenetic segregation (Fig. 5a, b) across the different recombination fragment trees in the coordinate range 1–682 for tree 1 (Fig. 5a) and 682–2212 range for tree 2 of the NM1-A2 sulphur-metabolizing gene. However, in NR1, the coordinate ranges are 1–2397 for tree 1 (Fig. 5c) and 682–2624 ranges for tree 2 (Fig. 5d) (Fig. 5).

### Protein structure prediction

**Fig. 6.**
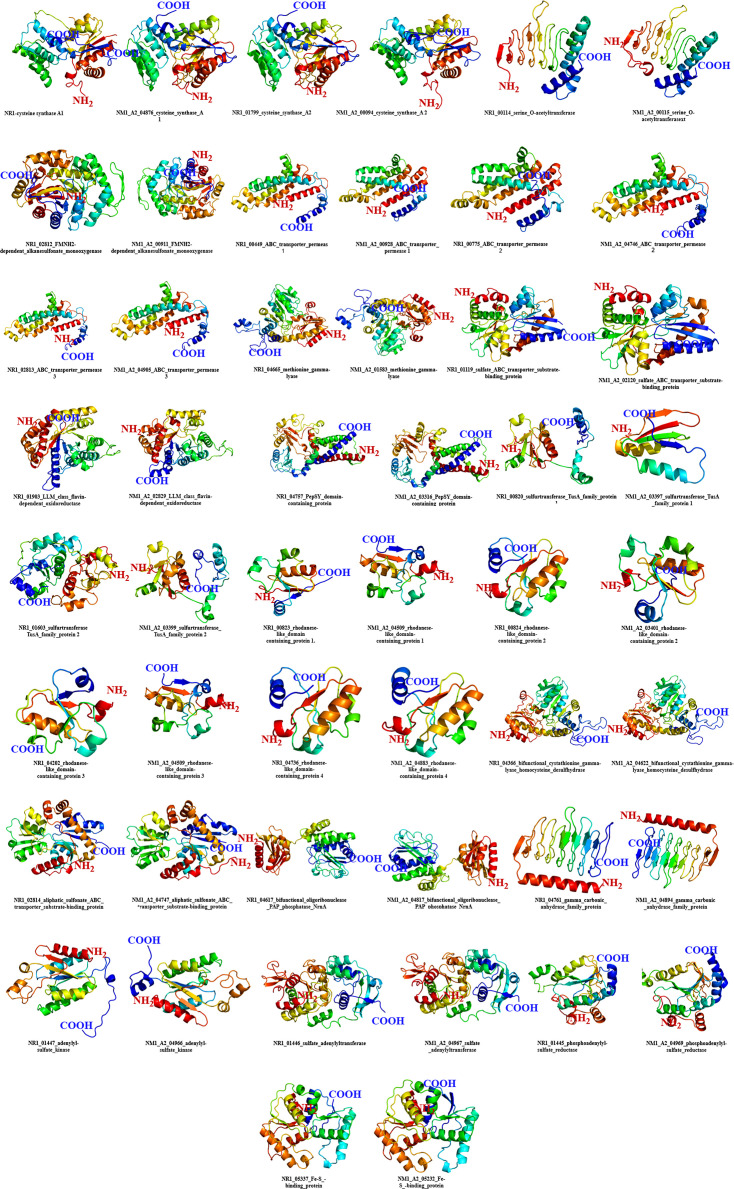
Predicted secondary structural features of sulphur metabolism-related proteins in *B. aryabhattai* NM1-A2 and *B. cereus* NR1.

The three-dimensional structural models and secondary structure attributes were predicted for common variants of the NM1-A2 and NM1 sulphur-metabolizing genes (Fig. 6). The secondary structure of a protein is considered a bridge between its tertiary structure and primary sequence. Accurate prediction of secondary structure enhances the precision of structure-based attribute analysis. For NM1-A2 proteins, disorder ranges from 0% to 19%, with alpha helices ranging from 14% (gamma carbonic anhydrase family protein) to 80% (ABC transporter permease.1), and beta sheets ranging from 0% (ABC transporter permease 1, 2 and 3) to 35% (sulfurtransferase TusA family protein 2), and the TM helix ranges from 4% (FMNH2-dependent alkanesulfonate monooxygenase.1) to 53% (ABC transporter permease.3) (Fig. 6 and Section S4).

Conversely, for selected NR1 proteins, the disorder percentage ranges from 0% to 16%, alpha helix spanning from 14% (gamma carbonic anhydrase family protein) to 80% (ABC transporter permease.2) and beta sheets from 0% (ABC transporter permease.1,2,3) to 41% (gamma carbonic anhydrase family protein), and the TM helix ranges from 4% (FMNH2-dependent alkanesulfonate monooxygenase.1) to 55% (ABC transporter permease.2), respectively (Sections S1 and S4, Table S3 and Figs 6 and S1).

## Discussion

The gene families’ genetic information evaluation could provide significant information about the genes' evolutionary history, including the instances of duplication events, as well as the diversification mechanisms [42]. High-throughput sequencing technologies such as next-generation sequencing and bioinformatics applications have made it possible to uncover genes’ fundamental functions, adaptations and their contribution to various biological processes [43]. Comparative genomics is also helpful in analysing and comparing gene sequences from different species to offer a substantial understanding of the evolutionary history [44]. This study comprehensively analysed the sulphur-metabolizing gene family of 2 bacterial strains, NM1-A2 and NR1, with 35 and 34 gene sequences in each, respectively, which were further divided into 4 major clades based on the degree of homology across gene sequences.

Phylogenetics is crucial for understanding the functional variations that evolved in different bacterial strains’ genes over time, which track down the genetic connections acquired by bacteria in terms of new genes for environmental adaptations or to develop resistance [45,47]. This consideration is decisive in identifying genes with shared ancestry in distant strains [48]. In this study, the sulphur-metabolizing genes of both strains were grouped into four different clades, where the clades’ structural relationship is probably due to functional similarities between both bacterial strains [49]. Besides, our results are also in line with previous studies that reported similar clade structures for sulphur metabolism genes, demonstrating preserved evolutionary trajectories [50] and underlining the conserved regions’ implication for protein functionality [51].

The MEME motif elucidation suggests comprehending proteins' functional and structural aspects; however, variable regions might demonstrate functional adaptations or mechanistic diversification [52]. Our results indicated that NM1-A2 contains motifs linked with Cys/Met metabolism, such as PLP-dependent enzyme domains, Bac luciferase, sulfurtransferase TusA and Rhodanese, and the domain distribution aligns with known functional motifs for sulphur metabolism, suggesting that NM1-A2 sulphur metabolism genes are well-equipped with these indispensable enzymatic functions. In contrast, NR1 MEME motifs also recognized comparable functional domains with diverse amino acid configurations. NR1 encompasses motifs of rhodanese and BPD_transp1, less often stated for sulphur metabolism, indicating that NR1 may acquire sole regulatory mechanisms or enzymatic adaptations for sulphur metabolism compared to NM1-A2. Recent studies also illustrated sulphur metabolism enzyme diversity among bacterial species [45,47]. Guo *et al*. reported that rhodanese and sulfurtransferase domains were predominant in sulphur-metabolizing bacteria, aligning with our findings for NM1-A2 [45,47]. However, Jones also mentioned the presence of unique motifs that were not previously characterized [47], similar to our novel findings for NR1. This underscores the evolutionary variability and potential functional specialization within sulphur-metabolizing bacterial strains [50].

The physicochemical attributes, including MWs, pIs and IIs of a protein, are important features as an adaptive mechanism, and both the NM1-A2 and NR1 proteins displayed an acidic nature and hydrophobic characteristics, with instability indices suggesting differential thermostability variability in thermostability [49]. Additionally, the GRAVY values characterize and measure a protein’s hydrophobic (positive GRAVY score) or hydrophilic (negative GRAVY score) properties [5]. The present study demonstrates that NM1-A2 and NR1 exhibit pronounced hydrophobic characteristics, consistent with earlier reports on sulphur-metabolism-related proteins [53]. This observation is further supported by the findings of Miller *et al*., who identified acidic and hydrophobic properties as common features of sulphur metabolism proteins, thereby reinforcing our study [49].

Moreover, the functionality and adaptability of *Bacillus* species, predominantly the chemical degradation and secondary metabolite production, are critically influenced by gene duplication [54]. The gene duplication is responsible for enhancing the biosynthetic pathways in various *Bacillus* species, including *Bacillus amyloliquefaciens* or *B. subtilis* [55]. For instance, bacA, bacB and bacC [56] and the toxin gene duplication [48] provided an inexpensive benefit in microbial ecosystems. Additionally, proteases, amylases, cellulases, lipases [57], peroxidase, nitrilase and dehydrogenase genes [58] are not only efficient for fermentation-associated substrate breakdown but also valuable for the bioremediation of toxic chemicals, including nitriles, hydrocarbons and phenols [59]. In this study, we identified 20 gene pairs of duplication, of which NM1-A2 has 11, and NR1 has 9, respectively, which could be imperative for agricultural, industrial and environmental applications, emphasizing their key role in biotechnology and sustainability. Furthermore, all the duplicated gene pairs of NM1-A2 and NR1 are under purifying selection with Ka/Ks ratios >1 except for one gene pair of NR1 FNR/RDP.3, demonstrating a positive selection pressure.

Recombination is crucially involved in bacterial genome evolution or adaptation in mangrove environments, where conjugation, transformation and transduction let those bacteria attain valuable genes such as stress response proteins and antibiotic resistance [60]. Especially, *Bacillus* species in mangroves could acquire the integrated DNA to acclimate to severe environmental circumstances and boost their subsistence [61]. Besides, recombination also aids in DNA repair [62], ensuring genetic stability under adverse conditions [63]. In this study, we also observed recombination patterns in both NM1-A2 and NR1, with 87 and 64 recombination regions in each strain that enhance the genetic flexibility, survival and industrial potential of *Bacillus* species in mangrove ecosystems, making them valuable for ecological and biotechnological applications.

Previous studies have demonstrated that core sulphur metabolism pathways, including sulphate transport (*cysPUWA*), assimilatory sulphate reduction (*cysH*, *cysI/J*), sulphur amino acid biosynthesis (*metE*, *metH*, *metB* and *metC*) [1,2] and redox homeostasis (*trx*, *grx*), are highly conserved across *Bacillus* species [64]. Beyond these canonical modules, other sulphur-related pathways, such as thiosulphate and tetrathionate oxidation (sox genes), sulphide detoxification (*sqr*, *pdo*), and organic sulphur compound utilization (*ssuABC*, *tauABC* and *dmdA*), have been reported in certain marine or soil-associated *Bacillus* strains, reflecting niche-specific metabolic adaptations [65]. In concordance with these canonical pathways, the majority of sulphur metabolism genes identified in NM1-A2 and NR1 map to these conserved modules, indicating that both strains retain a coherent and evolutionarily stable sulphur metabolic architecture [1,2]. Nevertheless, the observed heterogeneity in gene copy number, genomic organization and recombination signatures suggests ongoing strain-level diversification and microevolutionary adaptation, potentially driven by selective pressures associated with sulphur-rich mangrove environments [66]. Collectively, these findings underscore the coexistence of a conserved core sulphur metabolic repertoire with superimposed strain-specific genomic plasticity, highlighting the necessity for future large-scale comparative genomic and pan-genome analyses to rigorously delineate core versus accessory sulphur metabolism genes across *B. aryabhattai* and *B. cereus* lineages.

## Conclusion

This comprehensive study successfully characterized the sulphur metabolism-related gene family across the entire genomes of *B. aryabhattai* strain NM1-A2 and *B. cereus* strain NR1, both isolated from marine mangrove habitats. The analysis revealed significant phylogenetic relationships among the sulphur-metabolizing genes, with distinct MEME motifs and conserved domain regions identified within the gene family. Phylogenetic analysis and multiple sequence alignment provided insights into the evolutionary history of these genes. At the same time, duplication events and cellular distribution patterns further highlighted the complexity of sulphur metabolism in these strains. Recombination analysis using GARD identified potential recombination events that may have contributed to the diversity of these genes, which is ultimately helpful for environmental adaptation. Finally, protein structure predictions offered a deeper understanding of the functional aspects of sulphur metabolism in NM1-A2 and NR1. Overall, this study enhances our understanding of sulphur metabolism in *Bacillus* strains NM1-A2 and NR1 from mangrove environments and provides a foundation for future research in microbial sulphur metabolism.

## Supplementary material

10.1099/mgen.0.001713 Uncited Supplementary Material 1.

## Abbreviations

AI: aliphatic index
CDD: conserved domain database
GARD: Genetic Algorithm Recombination Detection
GRAVY: grand average of hydropathicity
II: instability index
MEME: Multiple Expectation Maximization for Motif Elicitation
MW: molecular weight
pI: isoelectric point
