# Supplementary material for: Genome-wide characterization of sulphur metabolism gene families and recombination dynamics in mangrove-derived Bacillus aryabhattai NM1-A2 and Bacillus cereus NR1
Source: Microb Genom. 2026 May 22;12(5):001713. doi: 10.1099/mgen.0.001713 (PMC13196889; doi:10.1099/mgen.0.001713)

Genome-wide characterization of sulfur metabolism gene families and recombination dynamics in mangrove-derived *Bacillus aryabhattai* NM1-A2 and *Bacillus cereus* NR1

Authors: Muhammad Kashif [1, 2, 3], Tingmei Li [1], Dan Wang [1], Can Meng [1], Yujia Luo [1], Qi Liang [1], Feng Guo [1], Saif-ur-Rehman [5], Sheng He [4] and Chengjian Jiang [1, 2, 3, *]

Affiliation:

[1] Guangxi Key Laboratory for Green Processing of Sugar Resources, Guangxi Technology Innovation Center of Liuzhou Luosi Rice Noodle, Innovation Research Center for Medical-Engineering Integration, College of Biological and Chemical Engineering, Guangxi University of Science and Technology, Liuzhou 545006, China.

[2] Guangxi Technology Innovation Center for Microbial Resources Development and Utilization, College of Life Science and Technology, Guangxi University, Nanning 530004, China.

[3] National Engineering Research Center for Non-Food Biorefinery, Guangxi Research Center for Biological Science and Technology, Guangxi Academy of Sciences, Nanning 530007, China.

[4] Guangxi Birth Defects Prevention and Control Institute, Maternal and Child Health Hospital of Guangxi Zhuang Autonomous Region, Nanning 530033, China.

[5] Department of Public Health, Rawalpindi Medical University, Rawalpindi 46000, Pakistan.

*: **Corresponding author**

Tel: +86-771-3237873, Fax: +86-771-3237873

E-mail: jiangcj0520@vip.163.com (*)

Section 1

Table S1. Recombination analysis of a total 87 regions for whole-genome nucleotide sequences of NM1-A2.

Sr.No.	Position of Break and Endpoint		Parents		Methods and *p*-values								
	Begin	End	Major	Minor	RDP	GENECONV	Bootscan	MaxChi	Chimaera	SiScan	PhyloPro	LARD	3Seq
1	3000502	3044724	BA_K13	BM_DSM319	8.155×10-312	---	---	7.281×10-37	5.387×10-40	---	--	--	--
2	2609296	2613570	BA_K13	unknown	2.249×10-289	---	---	4.033×10-31	3.907×10-35	2.237×10-23	---	---	---
3	3662854	3669569	BA_K13	unknown	4.637×10-272	---	5.564×10-46	1.988×10-28	1.523×10-22	8.723×10-44	---	---	---
4	1705621	1714363	BA_K13	unknown	1.045×10-246	---	---	7.146×10-25	4.613×10-12	3.676×10-31	---	---	---
5	3671996	3675785	BA_K13	unknown	9.109×10-220	---	---	2.508×10-25	2.435×10-14	1.929×10-34	---	---	---
6	1727279	1728788	BA_K13	unknown	2.267×10-182	---	---	9.036×10-26	2.413×10-27	1.282×10-18	---	---	---
7	1891725	1803756	BA_K13	unknown	3.789×10-153	---	---	---	3.888×10-08	9.788×10-16	---	---	---
8	2714198	2716648	BA_K13	BM_QMB1551	2.111×10-13	---	7.705×10-13	8.997×10-21	8.555×10-06	1.094×10-14	---	---	---
9	1765485	1785428	BA_K13	BM_QMB1551	2.516×10-102	---	1.058×10-57	---	4.235×10-04	8.982×10-23	---	---	---
10	1715340	1715759	BA_K13	unknown	2.989×10-77	---	---	8.130×10-10	5.264×10-12	---	---	---	---

11	4884594	4885025	BA_K13	BM_DSM319	1.624×10-72	---	---	2.047×10-14	2.477×10-15	1.039×10-08	---	---	---
12	5437832	1714913	BA_K13	unknown	3.837×10-66	---	---	---	6.469×10-03	---	---	---	---
13	6201034	6205323	BA_K13	BM_QMB1551	1.607×10-64	---	4.976×10-72	5.288×10-16	3.094×10-16	---	---	---	---
14	2661126	2662272	BA_K13	BM_DSM319	1.607×10-57	---	3.772×10-20	1.058×10-13	2.636×10-03	7.355×10-05	---	---	---
15	6220272	6224247	BA_K13	BM_QMB1551	1.913×10-52	---	2.611×10-112	8.535×10-10	1.364×10-09	5.109×10-14	---	---	---
16	3067245	3068244	BA_K13	BM_DSM319	2.731×10-49	---	---	5.011×10-12	4.106×10-12	---	---	---	---
17	3764613	3784320	BA_K13	BM_DSM319	1.234×10-48	---	---	6.061×10-07	1.961×10-09	---	---	---	---
18	2719699	2721672	BM_QMB1551	BA_K13	2.397×10-45	---	---	1.790×10-15	1.446×10-17	4.175×10-09	4.440×10-15	---	---
19	1852252	1854548	BA_K13	BM_QMB1551	1.366×10-44	---	9.083×10-54		2.446×10-03	---	---	---	---
20	6215066	6217967	BA_K13	BM_QMB1551	2.070×10-44	---	5.758×10-81	5.552×10-13		6.276×10-08	---	---	---
21	6213061	6265793	BA_K13	BM_QMB1551	4.795×10-39	---	6.175×10-36	2.132×10-07	3.121×10-07	5.007×10-04	---	---	---
22	3710531	3789325	BA_K13	BM_DSM319	5.326×10-39	---	2.597×10-14	2.744×10-08	1.457×10-07	3.326×10-05	---	---	---
23	3981943	3982948	BA_K13	BM_QMB1551	1.254×10-31	---	---	6.743×10-11	1.168×10-09	---	2.899×10-13	---	---
24	1849687	1850699	BA_K13	BM_QMB1551	8.619×10-	---	---	4.417×10-	2.849×10-	---	---	---	---

					28			07	08				
25	2777147	2777768	BA_K13	BM_DSM319	1.902×10-26	--	--	2.316×10-09	4.024×10-09	6.219×10-09	--	--	--
26	3961900	3962179	BA_K13	BM_DSM319	8.212×10-25	--	--	5.238×10-05	--	--	--	--	--
27	2302897	2303248	BA_K13	BM_DSM319	7.814×10-25	--	--	7.253×10-06	--	--	--	--	--
28	4229605	4240370	BA_K13	BM_QMB1551	1.742×10-22	--	3.582×10-26		--	--	2.882×10-13		
29	3778455	3778840	BA_K13	BM_DSM319	1.351×10-22	--	--	6.104×10-08	--	--	--	--	--
30	4345526	4345858	BA_K13	BM_DSM319	8.909×10-22	--	--	3.519×10-05	--	--	--	--	--
31	3431692	3432139	BA_K13	BM_DSM319	3.386×10-21	--	--	2.073×10-05	--	--	--	--	--
32	3983425	3976539	BA_K13	BM_DSM319	8.281×10-24	--	--	--	--	8.347×10-03	--	--	--
33	2181260	2181536	BA_K13	BM_QMB1551	6.962×10-20	--	--	1.834×10-03	--	--	--	--	--
34	1734403	1735117	BA_K13	BM_QMB1551	7.089×10-20	--	--	--	--	2.903×10-08	--	--	--
35	2110549	2111313	BA_K13	Unknown	2.336×10-19	--	--	4.243×10-07	4.552×10-06	--	--	--	--
36	1786535	1716455	BA_K13	BM_DSM319	6.571×10-43	--	7.150×10-20	--	--	--	--	--	--
37	4554017	4554457	BA_K13	Unknown	1.862×10-18	--	--	1.748×10--05	--	--	--	--	--

38	2191109	2191328	BA_K13	BM_DSM319	3.248×10-18	--		1.753×10-03	--		--	--	--
39	1718210	1718668	BA_K13	BM_QMB1551	5.205×10-18	--	2.638×10-10		--	1.944×10-05	--	--	--
40	3636068	3637118	BA_K13	BM_DSM319	5.858×10-18	--	--	8.652×10-08	--	--	--	--	--
41	2219983	2246563	BA_K13	BM_DSM319	9.113×10-18	--	--	5.900×10-04	--	--	--	--	--
42	5643298	5643677	BA_K13	BM_DSM319	1.577×10-17	--	--	3.430×10-03	--	--	--	--	--
43	1756389	1719417	BA_K13	BM_QMB1551	3.805×10-16	--	--	--	--	3.784×10-04	--	--	--
44	4818400	4818858	BA_K13	BM_QMB1551	8.999×10-16	--	--	1.586×10-05	3.285×10-05	--	--	--	--
45	2723402	2723580	BA_K13	BM_QMB1551	2.342×10-15	--	--	2.705×10-02	--	--	--	--	--
46	1913588	1914443	BA_K13	BM_QMB1551	8.782×10-15	--	--	1.398×10-03	--	--	--	--	--
47	5484846	5485699	BA_K13	BM_DSM319	1.779×10-14	--	--	1.177×10-03	--	--	--	--	--
48	2583570	2583977	BA_K13	BM_QMB1551	6.827×10-14	--	--	--	--	3.171×10-12	--	--	--
49	3241535	3241898	BA_K13	BM_DSM319	6.626×10-14	--	--	1.949×10-03	--	--	--	--	--
50	3253411	3253762	BA_K13	BM_DSM319	8.255×10-14	--	--	3.552×10-06	--	--	--	--	--
51	4242200	4242541	BA_K13	BM_QMB1551	3.388×10-	--	--	6.384×10-	--	--	--	--	--

					13			03					
52	2186067	2186447	BA_K13	BM_DSM319	3.776×10-13	--	--	7.919×10-04	--	--	--	--	--
53	5572816	5573955	BA_K13	BM_QMB1551	8.721×10-13	--	--	9.193×10-80	--	--	--	--	--
54	2627918	2628491	BA_K13	BM_QMB1551	1.028×10-11	--	--	7.522×10-06	--	--	--	--	--
55	2228398	2228640	BA_K13	BM_DSM319	1.800×10-11	--	--	5.157×10-05	--	--	--	--	--
56	1428943	1430138	BA_K13	BM_DSM319	1.888×10-11	--	--	6.484×10-03	--	--	--	--	--
57	2599119	2599347	BA_K13	BM_QMB1551	2.397×10-11	--	--	4.197×10-07	--	--	--	--	--
58	2383970	2384376	BA_K13	BM_QMB1551	6.366×10-11	--	--	2.756×10-02	--	--	--	--	--
59	4870632	4870832	BA_K13	BM_DSM319	9.277×10-11	--	--	2.715×10-03	--	--	--	--	--
60	3632469	3632952	BA_K13	BM_DSM319	9.318×10-11	--	--	4.147×10-04	--	--	--	--	--
61	3372222	33722450	BA_K13	BM_QMB1551	1.853×10-10	--	--	3.057×10-02	--	--	--	--	--
62	2772947	2773349	BA_K13	BM_QMB1551	1.803×10-10	--	--	3.757×10-03	--	--	--	--	--
63	3538151	3538881	BA_K13	BM_DSM319	5.827×10-08	--	--	6.629×10-10	3.235×10-05	--	--	--	--
64	5424906	5425037	BA_K13	BM_DSM319	6.862×10-10	--	--	1.536×10-10	--	--	--	--	--

65	2669705	2671045	BA_K13	BM_QMB1551	1.231×10-09	--	--	3.950×10-03	3.248×10-02	--	--	--	--
66	1857666	1858035	BA_K13	BM_QMB1551	1.246×10-09	--	--	1.846×10-02	--	--	--	--	--
67	1759655	1759746	BA_K13	BM_DSM319	4.068×10-09	--	--	--	--	1.788×10-24	--	--	--
68	4185861	4186860	BA_K13	BM_QMB1551	1.503×10-07	--	--	9.708×10-09	3.388×10-04	--	--	--	--
69	1781266	1781424	BA_K13	BM_DSM319	2.371×10-10	--	--	--	--	9.533×10-53	--	--	--
70	3349471	3349778	BA_K13	BM_DSM319	1.680×10-08	--	--	6.760×10-03	--	--	--	--	--
71	1871300	1871730	BA_K13	BM_QMB1551	2.145×10-08	--	--	1.345×10-04	--	--	--	--	--
72	3132712	3132870	BA_K13	BM_DSM319	3.449×10-08	--	--	2.482×10-07	--	--	--	--	--
73	5818574	5819390	BA_K13	BM_QMB1551	4.833×10-08	--	--	1.315×10-03	--	--	--	--	--
74	5100646	5101136	BA_K13	BM_QMB1551	5.010×10-08	--	--	4.210×10-09	--	--	--	--	--
75	4976555	4976852	BA_K13	BM_DSM319	4.999×10-08	--	--	7.783×10-04	--	--	--	--	--
76	6208415	6209130	BA_K13	BM_QMB1551	1.828×10-09	--	--	--	--	2.671×10-26	--	--	--
77	1815888	1816179	BA_K13	BM_DSM319	7.429×10-07	--	--	--	--	2.286×10-07	--	--	--
78	2801009	2801149	BA_K13	BM_DSM319	1.192×10-			2.215×10-			--	--	--

					06			06					
79	4231781	4312940	BA_K13	BM_QMB1551	1.845×10-06			7.119×10-10			--	--	--
80	4239273	4239563	BA_K13	BM_DSM319	2.945×10-06	--	--	--	--	1.195×10-298	--	--	--
81	2586892	2586938	BA_K13	BM_QMB1551	1.323×10-05	--	--	--	--	9.393×10-03	--	--	--
82	1717136	1082092	BA_K13	BM_QMB1551	1.399×10-05	--	--	--	--	6.537×10-13	--	--	--
83	2560138	2560293	BA_K13	BM_QMB1551	2.157×10-05	--	--	--	--	3.371×10-08	--	--	--
84	1717136	1717536	BA_K13	BM_QMB1551	1.399×10-05	--	--	--	--	6.537×10-13	--	--	--
85	3654084	3659146	BA_K13	BM_DSM319	5.501×10-23	--	--	2.441×10-23	2.887×10-05	1.478×10-09	--	--	--
86	1428943	1430138	BA_K13	BM_DSM319	1.888×10-11	--	--	6.484×10-03	--	--	--	--	--
87	4064920	4072171	BM_DSM319	BM_QMB1551	3.026×10-02	--	--	7.058×10-05	--	--	--	--	--

Table S2. Recombination analysis of a total 64 regions for whole-genome nucleotide sequences of NR1.

Sr.No.	Position of Break and Endpoint		Parents		Methods and *p*-values								
	Begin	End	Major	Minor	RDP	GENECONV	Bootscan	MaxChi	Chimaera	SiScan	PhyloPro	LARD	3Seq
1	5237150	5241434	BC_E33L	BA_AmesAncestor	1.435×10-316	---	---	3.468×10-41	6.015×10-42	---	--	--	--
2	2965711	2982598	BC_E33L	BA_Sterne	--	1.035×10-307	---	3.987×10-82	8.223×10-87	1.015×10-46	4.964×10-13	--	--
3	5257593	5259083	BC_E33L	BA_AmesAncestor	1.241×10-165	---	--	1.674×10-27	8.771×10-16	--	---	---	---
4	1867940	1868696	BC_E33L	BA_AmesAncestor	2.062×10-133	--	---	4.586×10-22	4.031×10-23	1.450×10-28	--	--	--
5	2084134	2085285	BC_E33L	BA_AmesAncestor	1.172×10-128	--	--	1.172×10-21	4.821×10-16	--	---	---	---
6	4726524	4726972	BC_E33L	BA_Sterne	2.084×10-84	--	--	4.689×10-20	8.934×10-21	---	---	----	---
7	1641016	1649541	BC_E33L	BA_AmesAncestor	---	--	--	2.758×10-63	2.928×10-66	1.391×10-58	--	--	--
8	551847	554948	BC_E33L	BA_AmesAncestor	1.282×10-54	--	--	4.838×10-15	--	--	--	--	--
9	657160	658425	BC_E33L	BA_AmesAncestor	2.593×10-54	--	--	2.279×10-14	9.261×10-15	--	--	--	--
10	658680	660799	BC_E33L	BA_AmesAncestor	1.301×10-51	--	--	1.055×10-13	3.412×10-14	9.820×10-145	--	--	--
11	5541712	5542088	BC_E33L	BA_AmesAncestor	2.541×10-46	--	--	2.553×10-10	1.256×10-11	--	--	--	--

12	3786073	3788328	BC_E33L	BA_AmesAncestor	6.859×10-46	--	--	--	2.392×10-11	--	--	--	--
13	2421050	2421755	BC_E33L	BA_AmesAncestor	1.832×10-43	--	--	2.253×10-12	1.765×10-14	--	--	--	--
14	3833296	3837446	BA_AmesAncestor	BC_E33L	7.808×10-36	--	--	1.173×10-18	8.965×10-09	--	--	--	--
15	1898039	1898558	BC_E33L	BA_AmesAncestor	2.200×10-33	--	--	3.288×10-08	4.859×10-03	--	--	--	--
16	322718	327312	BA_AmesAncestor	BC_E33L	7.961×10-35	--	--	1.650×10-15	7.334×10-13	1.169×10-92	--	--	--
17	1314495	1314965	BC_E33L	BA_AmesAncestor	5.719×10-26	--	--	3.913×10-08	2.124×10-08		--	--	--
18	1635230	1636218	BC_E33L	BA_AmesAncestor	9.026×10-26	--	--	--	4.998×10-03	--	--	--	--
19	4407381	4414891	BA_AmesAncestor	BC_E33L	1.013×10-24	--	--	7.504×10-10	7.646×10-09	--	--	--	--
20	4543080	4544046	BC_E33L	BA_AmesAncestor	8.139×10-23	--	--	3.678×10-04	--	--	--	--	--
21	Undetermined	3481450	BC_E33L	BA_AmesAncestor	1.086×10-20	--	--	9.1322×10-11	3.145×10-08	--	--	--	--
22	Undetermined 1282269	Undetermined 1285542	BC_E33L	BA_AmesAncestor	8.366×10-21	--	--	5.586×10-12	6.016×10-09	--	--	--	--
23	4896879	4899444	BC_E33L	BA_AmesAncestor	--	--	--	1.325×10-48	7.385×10-53	2.087×10-39	--	--	--
24	1933428	1942329	BA_AmesAncestor	BC_E33L	6.189×10-19	--	--	9.723×10-19	1.865×10-07	7.782×10-49	--	--	--
25	2313910	2314300	BC_E33L	BA_AmesAncestor	1.096×10-	--	--	--	4.088×10-	--	--	--	--

					18				12				
26	1333750	1333941	BC_E33L	BA_AmesAncestor	3.229×10-18	--	--	5.076×10-06	--	--	--	--	--
27	1532633	1532775	BC_E33L	BA_AmesAncestor	3.649×10-17	--	--	4.223×10-05	--	--	--	--	--
28	3227044	3227257	BC_E33L	BA_AmesAncestor	1.836×10-16	--	--	7.545×10-05	3.856×10-05	--	--	--	--
29	4277527	4335919	BA_AmesAncestor	BC_E33L	5.975×10-08	--	--	9.787×15-15	7.591×10-06	8.049×10-233	--	--	--
30	Undetermined	2605761	BC_E33L	BA_AmesAncestor	8.159×10-14	--	--	6.789×10-07	--	--	--	--	--
31	788494	788668	BC_E33L	BA_AmesAncestor	2.552×10-13	--	--	7.464×10-12	--	--	--	--	--
32	4257549	4257981	BC_E33L	BA_AmesAncestor	9.432×10-12.	--	--	1.306×10-12.	1.872×10-07	--	--	--	--
33	2834532	2835120	BC_E33L	BA_AmesAncestor	2.442×10-12.	--	--	3.727×10-07	--	--	--	--	--
34	3032646	3032834	BC_E33L	BA_AmesAncestor	3.449×10-12	--	--	3.332×10-07	--	--	--	--	--
35	2193051	2193321	BC_E33L	BA_AmesAncestor	6.248×10-12	--	--	2.854×10-07	--	--	--	--	--
36	4951340	4951403	BC_E33L	BA_AmesAncestor	7.507×10-12	--	--	1.754×10-03	4.633×10-03	--	--	--	--
37	1882713	1882941	BC_E33L	BA_AmesAncestor	7.043×10-11	--	--	6.124×10-07	7.283×10-05	--	--	--	--
38	3149514	3149702	BC_E33L	BA_AmesAncestor	1.195×10-10	--	--	--	--	3.288×10-112	--	--	--

39	4415288	4415699	BC_E33L	BA_AmesAncestor	1.325×10-10	--	--	--	--	1.534×10-103	--	--	--
40	1954543	1954700	BC_E33L	BA_AmesAncestor	1.294×10-09	--	--	3.494×10-02	--	--	--	--	--
41	Undetermined	1831174	BC_E33L	BA_AmesAncestor	2.772×10-09	--	--	--	--	2.397×10-94	--	--	--
41	2657568	2657694	BC_E33L	BA_AmesAncestor	3.526×10-09	--	--	3.447×10-02	--	--	--	--	--
43	6073354	6073537	BC_E33L	BA_AmesAncestor	6.066×10-09	--	--	--	--	5.114×10-18	--	--	--
44	2519898	Undetermined	BC_E33L	BA_AmesAncestor	1.645×10-08	--	--	4.353×10-05	--	--	--	--	--
45	1031943	1032627	BC_E33L	BA_AmesAncestor	1.078×10-04	--	--	7.677×10-08	--	--	--	--	--
46	3249698	3240877	BC_E33L	BA_AmesAncestor	5.668×10-07	--	--	3.043×10-04	--	--	--	--	--
47	Undetermined	505986	BC_E33L	BA_AmesAncestor	1.291×10-06	--	--	1.489×10-06	--	--	--	--	--
48	3165128	3165371	BC_E33L	BA_AmesAncestor	1.292×10-06	--	--	3.344×10-04	--	--	--	--	--
49	2809624	2809705	BC_E33L	BA_AmesAncestor	1.519×10-06	--	--	2.492×10-02	--	--	--	--	--
50	1170374	1170824	BC_E33L	BA_AmesAncestor	2.155×10-06	--	--	--	--	1.246×10-62	--	--	--
51	2355328	Undetermined	BC_E33L	BA_AmesAncestor	1.184×10-03	--	--	9.852×10-09	--	--	--	--	--
52	3314941	3315103	BC_E33L	BA_AmesAncestor	5.212×10-	--	--	1.773×10-	--	--	--	--	--

					06			02					
53	854316	854560	BC_E33L	BA_AmesAncestor	5.958×10-06	--	--	--	--	2.366×10-50	--	--	--
54	3732417	3732853	BC_E33L	BA_AmesAncestor	8.015×10-04	--	--	8.152×10-06	--	---	--	--	--
55	947960	948116	BC_E33L	BA_AmesAncestor	1.021×10-05	--	--	1.882×10-03	--	--	--	--	--
56	2929324	Undetermined	BC_E33L	BA_AmesAncestor	3.222×10-05	3.222×10-05	3.222×10-05	3.222×10-05	3.222×10-05	3.400×10-49	--	--	--
57	3028430	3028817	BC_E33L	BA_AmesAncestor	4.212×10-05	--	--	--	--	1.661×10-174	--	--	--
58	483567	483949	BC_E33L	BA_AmesAncestor	7.140×10-05	--	--	5.995×10-06	--	--	--	--	--
59	874994	875156	BC_E33L	BA_AmesAncestor	2.272×10-04	--	--	--	--	7.943×10-183	--	--	--
60	2244165	2244556	BC_E33L	BA_Sterne	1.205×10-03	--	--	1.301×10-05	--	--	--	--	--
61	Undetermined	Undetermined	BC_E33L	BA_AmesAncestor	2.731×10-03	--	--	1.006×10-05	--	--	--	--	--
62	3904287	3904562	BC_E33L	BA_AmesAncestor	4.358×10-02	--	--	1.353×10-02	--	--	--	--	--
63	Undetermined	2591495	BC_E33L	BA_AmesAncestor	1.693×10-02	--	--	6.634×10-06	--	--	--	--	--
64	4817634	Undetermined	BC_E33L	BA_AmesAncestor	1.746×10-02	--	--	--	--	1.083×10-25	--	--	--

Table S3. NM1-A2 Secondary structure conformation.

Sr. N0	Name of Proteins	Alpha helix<br>Alpha helix	Beta strand<br>Beta strand	Disorder<br>? Disordered	TM helix
1	Cysteine synthase A.1	33%	16%	1%	-----
2	Cysteine synthase A.2	36%	15%	1%	10%
3	Serine O-acetyltransferase	31%	25%	14%	14%
4	FMNH2-dependent alkanesulfonate monooxygenase.1	34%	16%	7%	4%
5	ABC transporter permease.1	80%	0%	2%	57%
6	ABC transporter permease.2	74%	0%	14%	50%
7	ABC transporter permease.3	76%	0%	9%	53%
8	Methionine gamma-lyase	34%	16%	3%	-----
9	Sulfate ABC transporter substrate-binding protein	34%	14%	12%	5%
10	LLM class flavin-dependent oxidoreductase	40%	14%	5%	-----
11	PepSY domain-containing protein	47%	12%	19%	30%
12	Sulfurtransferase TusA family protein.1	28%	34%	3%	-----

13	Sulfurtransferase TusA family protein.2	27%	35%	2%	-----
14	Rhodanese-like domain-containing protein.1	36%	16%	3%	-----
15	Rhodanese-like domain-containing protein.2	35%	12%	0%	30%
16	Rhodanese-like domain-containing protein.3	40%	12%	5%	13%
17	Rhodanese-like domain-containing protein.4	24%	17%	4%	-----
18	Bifunctional cystathionine gamma-lyase	33%	20%	1%	-----
19	Aliphatic sulfonate ABC transporter substrate-binding protein	36%	15%	6%	-----
20	Bifunctional oligoribonuclease	39%	18%	1%	-----
21	Gamma carbonic anhydrase family protein	14%	37%	6%	-----
22	Adenylyl-sulfate kinase	34%	14%	11%	-----
23	Sulfate adenylyltransferase	27%	20%	3%	-----
24	Phosphoadenylyl-sulfate reductase	28%	10%	3%	-----
25	(Fe-S)-binding protein	43%	3%	4%	16%

Table S4. NR1 Secondary structure conformation.

Sr. N0	Name of Proteins	Alpha helix<br>Alpha helix	Beta strand<br>Beta strand	Disorder<br>? Disordered	TM helix
1	Cysteine synthase A.1	33%	16%	1%	10%
2	Cysteine synthase A.2	33%	15%	1%	10%
3	Serine O-acetyltransferase	26%	27%	14%	10%
4	FMNH2-dependent alkanesulfonate monooxygenase.1	37%	15%	1%	4%
5	ABC transporter permease.1	79%	0%	8%	54%
6	ABC transporter permease.2	80%	0%	3%	55%
7	ABC transporter permease.3	71%	0%	15%	50%
8	Methionine gamma-lyase	34%	17%	2%	-----
9	Sulfate ABC transporter substrate-binding protein	36%	13%	12%	5%
10	LLM class flavin-dependent oxidoreductase	39%	19%	5%	-----
11	PepSY domain-containing protein	49%	10%	16%	33%

12	Sulfurtransferase TusA family protein.1	24%	20%	4%	-----
13	Sulfurtransferase TusA family protein.2	24%	14%	2%	-----
14	Rhodanese-like domain-containing protein.1	42%	13%	2%	13%
15	Rhodanese-like domain-containing protein.2	35%	17%	0%	-----
16	Rhodanese-like domain-containing protein.3	40%	11%	6%	13%
17	Rhodanese-like domain-containing protein.4	28%	17%	5%	-----
18	Bifunctional cystathionine gamma-lyase	34%	19%	0%	-----
19	Aliphatic sulfonate ABC transporter substrate-binding protein	38%	16%	6%	-----
20	Bifunctional oligoribonuclease	40%	19%	5%	5%
21	Gamma carbonic anhydrase family protein	14%	41%	3%	-----
22	Adenylyl-sulfate kinase	31%	15%	8%	-----
23	Sulfate adenylyltransferase	33%	19%	4%	-----

24	Phosphoadenylyl-sulfate reductase	33%	11%	6%	-----
25	(Fe-S)-binding protein	44%	4%	5%	15%

Figure S1. Multiple sequence alignment between the common variants of NM1-A2 and NR1.

Cysteine synthase A.1

```
NM1.(Fe-S)-binding protein  MTGLLWVNFLFFIIVTAYAISLFVYLIRTRIEYIQLGKKVEFDQRLKERLQKIWVNVFGQKKLLKDKKSG  70
NR1.(Fe-S)-binding protein  MNSLLIINWLAAIAVIAYAGYLFVYLIRTRMAYIQLGKKIEFDRRFKERWDLLKVNVFGQKKLLKDKKSG  70

NM1.(Fe-S)-binding protein  IMHVMFFYGFILVQFGALDFIIKGLLPGKHLPLGPLYPGFTFFQEIVTFLILVAVVWAFHRRYVEKLVRL  140
NR1.(Fe-S)-binding protein  IIHVMFFYGFILVQFGAIDFVWKGLAPGSHLPLGPLYPAFTFFQEIVTLVILIAVFWAFHRRYVEKLVRL  140

NM1.(Fe-S)-binding protein  KRGFKSGLVLIFIGALMLSVLVGNGMSIIWHNHEPAWTEPIASSIAILFKGINDTAAISIFYVSWWIHLA  210
NR1.(Fe-S)-binding protein  KRNFKSGLVLIFIGGLMISVLLGNGMGLIWHGEELSWSEPIASAIAYVFSGINETVAISVFYFSWWVHLL  210

NM1.(Fe-S)-binding protein  VLLTFLVYVPQSKHAHLLAGPANVFFNRVSKPGKLEKIDFEDETQETFGVGKIEEFTQHQLIDLYACVEC  280
NR1.(Fe-S)-binding protein  ILLTFLVYVPQSKHAHLIAGPANVFFGRLSNPGKLEKIDFEDETQETFGVGKIEDFRQNQLIDLYACVEC  280

NM1.(Fe-S)-binding protein  GRCTNMCPATGTGKILSPMDLILKMRDHLTEKGAVVTSKAPWVPTYAFSKTKGNQLAMMAASQGGAEAAA  350
NR1.(Fe-S)-binding protein  GRCTNMCPATGTGKMLSPMDLILKLRDHLTDKGAAVTSKAPWVPVVAFNNTQGNQLAMMAAGKGQQESAS  350

NM1.(Fe-S)-binding protein  T-AEYNPALIGDVITEEEIWACTTCRNCEDQCPVMNEHVDKIIDLRRFLVLTEGKMDADAQRAMTNIERQ  419
NR1.(Fe-S)-binding protein  TTLAYDPSLIGDVITEEEIWACTTCRNCEDQCPVMNEHVDKIIDLRRYLVLTEGKMDAEAQRAMTNIERQ  420

NM1.(Fe-S)-binding protein  GNPWGLNRKEREEWRHVREDVHIPTVKEVSKAGETFEYLFWVGSMGSYDNRSQKIALSFAKLLNEAGVSF  489
NR1.(Fe-S)-binding protein  GNPWGLNRKERETWRQGDDEVTVPTVKEKSKAGEEFEYLFWVGSMGSYDNRSQKIAISFAKLMNEAGISF  490

NM1.(Fe-S)-binding protein  AILGNKEKNSGDTPRRLGNEFLFQELASKNIAEFEKNEIKKIVTIDPHAYNIFKNEYPDFGFEGEVYHHT  559
NR1.(Fe-S)-binding protein  AILGNKEKNSGDTPRRLGNEFVFQEMATKNIEEFEKAGVKKIVTIDPHAYNTFKNEYPDFGLQAEVYHHT  560

NM1.(Fe-S)-binding protein  ELLAMLVKEGKLTPKHAVNEKITFHDSCYLGRYNEVYSPPREILKAIPGVSLIEMDRNRENGMCCGAGGG  629
NR1.(Fe-S)-binding protein  ELLAQWVKEGRLKPVHAIEETVTYHDSCYLGRYNEVYEAPRDILKAIPGVNLVEMARNRETGMCCGAGGG  630

NM1.(Fe-S)-binding protein  LMWTEETTGQRINVARTEQALAVNPSIISSGCPYCLTMLSDGTKAKEVEEKVHTYDVAELLEKSIYGDVQ  699
NR1.(Fe-S)-binding protein  LMWMEETTGSRINVARTEQALAVQPSIIGTGCPYCLTMISDGTKAKEVEEKVQTLDVTEILERSVIGQKK  700

NM1.(Fe-S)-binding protein  EQVS                                                                    703
NR1.(Fe-S)-binding protein  EAM-                                                                    703
```

Cysteine synthase A.2

```
NM1.Cysteine synthase A.2  MKVVNNMADLIGETPLVKLNRLQPADGASVYLKLEFFNPSRSVKDRAAFNMIVEAEKAGLLNENSTIIEP  70
NR1.Cysteine synthase A.2  MKLCENVTELIGDTPVVRLSKFIPEDAADVYVKLEMFNPSRSVKDRAAYNLLHVAEENGLIKPGDTIIEP  70

NM1.Cysteine synthase A.2  TSGNTGIGLAMNAAARGYRSILVMPDTMTQERINLLKAYGAEVVLTPGDEKMPGAIRKAEELTKEIPNAF  140
NR1.Cysteine synthase A.2  TSGNTGIGLAMNAAAKGYKAILIMPDNMSKERINLLKAYGAEVVLTPAEQRMPGAIAKALELQKQIPNSF  140

NM1.Cysteine synthase A.2  MPMQFENNANPDAHRKTTAKEIIEAMNDLGKDLSAFVATAGTGGTITGTGEVLRENYPNMTVHVVEPAGS  210
NR1.Cysteine synthase A.2  IPQQFENPANPNIHRYTTALEIYEQMDG---ELDAFVATAGTGGTITGTGETLKEKLPNLYIAVVEPKGS  207

NM1.Cysteine synthase A.2  PVLSGGRPGKHKLVGTSPGFIPDTLNIEVYDEILKIKDEQAYDITRRLASEEGILVGPSSGAACYAAIEV  280
NR1.Cysteine synthase A.2  PVLSGGVPGPHKLVGTSPGFIPKNLNTEVYNEIIQIADEEALTTMRNLARQEGLLVGPSSGASVYAAIMI  277

NM1.Cysteine synthase A.2  AKKLSPDQVVVCIACDTGERYLSSDLFSYE  310
NR1.Cysteine synthase A.2  AKRLGVGKKVLCIAPDTGERYLSMGLFE--  305
```

Serine O-acetyltransferase

```
NM1.serine O-acetyltransferase  MMLKLLKEDIEAIFEQDPAARSYIEVILTYSGLHAIWAHRFAHGLFKRKWFFLARVISQISRFFTGIEIH  70
NR1.Serine O-acetyltransferase  -MFKRLREDIEVVFEQDPAARGYFEVILTYSGLHAVWAHRIAHAFYKKNFFFLARFVSQVSRFFTGIEIH  69

NM1.serine O-acetyltransferase  PGAKIGRRFFIDHGMGIVIGETCEIGDNVTVYQGVTLGGTGKEKGKRHPTIEDNVLIATGAKVLGSITIH  140
NR1.Serine O-acetyltransferase  PGATIGRRFFIDHGMGVVIGETCEIGDNVTIYQGVTLGGTGKEKGKRHPTIQDNVLIATGAKVLGSITVG  139

NM1.serine O-acetyltransferase  AHSKIGAGSVVLNDVPENSTVVGIPGRVVIQNGVRIPKDLNHQDLPDPDAERFKQLEKEILHLQNQLKEL  210
NR1.Serine O-acetyltransferase  ENSKIGAGSVVLKEVPAHSTVVGIPGRVVIQNGVKIGQELNHSDLPDPIFDKLKAMEVELDKLKKQLELK  209

NM1.serine O-acetyltransferase  KEGKIHHGN---  219
NR1.Serine O-acetyltransferase  VERKDKNDYSHL  221
```

FMNH2-dependent alkanesulfonate monooxygenase.1

```
NM1.serine O-acetyltransferase  MMLKLLKEDIEAIFEQDPAARSYIEVILTYSGLHAIWAHRFAHGLFKRKWFFLARVISQISRFFTGIEIH  70
NR1.Serine O-acetyltransferase  -MFKRLREDIEVVFEQDPAARGYFEVILTYSGLHAVWAHRIAHAFYKKNFFFLARFVSQVSRFFTGIEIH  69

NM1.serine O-acetyltransferase  PGAKIGRRFFIDHGMGIVIGETCEIGDNVTVYQGVTLGGTGKEKGKRHPTIEDNVLIATGAKVLGSITIH  140
NR1.Serine O-acetyltransferase  PGATIGRRFFIDHGMGVVIGETCEIGDNVTIYQGVTLGGTGKEKGKRHPTIQDNVLIATGAKVLGSITVG  139

NM1.serine O-acetyltransferase  AHSKIGAGSVVLNDVPENSTVVGIPGRVVIQNGVRIPKDLNHQDLPDPDAERFKQLEKEILHLQNQLKEL  210
NR1.Serine O-acetyltransferase  ENSKIGAGSVVLKEVPAHSTVVGIPGRVVIQNGVKIGQELNHSDLPDPIFDKLKAMEVELDKLKKQLELK  209

NM1.serine O-acetyltransferase  KEGKIHHGN---                                                            219
NR1.Serine O-acetyltransferase  VERKDKNDYSHL                                                            221
```

ABC transporter permease.1

```
NM1.ABC transporter permease.1  --MKKYGAS----VILVVILLAAWEIGARIVN-YPFILPTPSGILTKLWELRMDLLFKHLPATLSIVVIG  63
NR1.ABC transporter permease.1  MNMQNQKQSKLITTIWLILLIAIWEGSVSLFKIEPWILPKPSAIVQELIGMK-DLLLPNTMQTLQEVIIG  69

NM1.ABC transporter permease.1  LVISVVLGVLLAVWMNWSPLIERAFYPLIIASQMIPTIAIAPVFVLWFGYSIWSKVIVTVLITFFPITVS  133
NR1.ABC transporter permease.1  LFFAILLGTSIAIIMDVIPLFRILINPLLVISQTIPIVVLAPLFIIWFGYGMLPKVMVVILVCFFPIALS  139

NM1.ABC transporter permease.1  TFDGLRSTNKELKELMLTMGATKKDIFFKLNIPSALPHFYSGLKVAVTFSIIGAAIGEWLGAQAGLGYFS  203
NR1.ABC transporter permease.1  ILEGFQTVDKNMLKLLQTMKATKWQVYQKVKFPAVLPYFFSGLKIAVTYSVMGAIIGEWLGASEGLGVML  209

NM1.ABC transporter permease.1  RRMMTQFDAAGVF--APIVILSALGILFFIIVVGFEKRSLKWRKTE--                        247
NR1.ABC transporter permease.1  TRATKSFLTARVFGVAAIIVMVTLCLYFIVEFMARITAPWIYRKDGRK                        257
```

ABC transporter permease.2

```
NM1.ABC transporter permease.2  MSKTSVVSGHVQSSAAHLQQVKKRKEEAKNKQGIFLRSLVVPILLIVVWQIIGSAGYVSKTVLPTPVDIV  70
NR1.ABC transporter permease.2  ----------------------------MNRLKELVPAITLSSILLAVWEIG-ARIVDEMYILPSPSAIL  41

NM1.ABC transporter permease.2  LSFKQLIVSGELGANLQISILRAAIGFAVGAGLGLLFGIIVGFSKKTEHYMDPSIQMLRTVPHLALAPLF  140
NR1.ABC transporter permease.2  MKIWKLKDI-LFTVHLPATLYVVLIGVVISIVLGVGLAMLMNASTWMERAFYPLLVASQTIPITALAPLF  110

NM1.ABC transporter permease.2  ILWFGFGEISKILLIANGAFFPIYVNTYLGIRGVDSKLFDVARVLQFSKWKQITKLILPAALPNILLGVR  210
NR1.ABC transporter permease.2  VLWFGYTIWSKVVVTVLITFFPIAVNTYDGLRSTKKEWEELLVTYGATKRDIFLKLKLPSALPYFFSALK  180

NM1.ABC transporter permease.2  LSLGIAWLGLVVAELMGASEGVGYMIMDARQFSQTDVVFVGIIIFAVVGKLTDSFVRVLEKRLLKWRDSY  280
NR1.ABC transporter permease.2  IAVPLSVIGAAIGEWLGAQAGLGYFSKRMMTQLDGAGVFAPIVLLSLLAIFFVVIISILEKKFISWRKHS  250

NM1.ABC transporter permease.2  AGEK                                                                    284
NR1.ABC transporter permease.2  ----                                                                    250
```

ABC transporter permease.3

```
NM1.ABC transporter permease.3  -------MKPTSSLDTLHESYLRKVRGEKRLVLSVQMVIFLAFFSFWELASRLKWIDPLIFSSPTKVWHL  63
NR1.ABC transporter permease.3  MENTKAVMKPAS--ITIEKNRVKNVRKLNVKVLVRAITIPVIILIIWQLAGVFGLVSKTVLPTPLDIFLA  68

NM1.ABC transporter permease.3  FLIKLADGSLAEHIGFTLFETVLGFILGTLLGILLATALWYSTRLANILDPYLVILNAMPKVALGPILIV  133
NR1.ABC transporter permease.3  FQELIKTGELFGHLSISVFRAAAGFFIGGGLGIILGTIVGFSTRSEQYLDPSVQMLRTVPHLAVAPLFVL  138

NM1.ABC transporter permease.3  AIGPGFFSILTMGAIISVIITSIVVYTAFKEVDPNYIKLLKSFGATKTRCFKEAILPASMPAIISTFKVN  203
NR1.ABC transporter permease.3  WFGFGETSKVLLIADGAFFPLYVNAFLGIRGVDSKLFDVARVLEFSKRKLITKLILPSALPNLLLGARLS  208

NM1.ABC transporter permease.3  VGLSWVGVIVGEFLVSSKGLGYMIIYGFQVFNFTLVLLSLLLIAIFATIMYQGVAYLEKKLIRRS-----  268
NR1.ABC transporter permease.3  LGVAWVSLVVAELMGSTEGIGYMIMDARQFSNTDIVFVGIIIFAFVGKFSDSLVRLLEAKFLRWRDNFKG  278

NM1.ABC transporter permease.3  ----                                                                    268
NR1.ABC transporter permease.3  ETGN                                                                    282
```

Methionine gamma-lyase

```
NM1.Methionine gamma-lyase  MNEKKKYQFETKAIHAGYESKHHFDSLAPPIYQTSTFTFSSLEQGANRFSGEENGYVYSRLSNPTVTILE  70
NR1.Methionine gamma-lyase  ---MKKKHMETALIHHGYKPEEYKGSLTPPLFQTSTFTFETAQQGEASFAGVDPSYIYSRLGNPTVKLFE  67

NM1.Methionine gamma-lyase  ERMAQLEEGEAALAFGSGMAAVSAVLIGLTKAGDHILCSKGVYGCTFGLLEMLEEKYQIHHSFSNLETEE  140
NR1.Methionine gamma-lyase  ERMAVLEGGEEALAFGSGMAAISATLIGFLKAGDHIICSNGLYGCTYGFLEVLEEKFMITHSFCDMETEA  137

NM1.Methionine gamma-lyase  EILAAIKEDTACIYIETPINPTMTLVDLELVTCIAKQKGIPVVVDNTFSTPYLQQPLKLGCDLVIHSATK  210
NR1.Methionine gamma-lyase  DIENKIRPNTKLIFVETPINPTMKLIDLKQVIRVAKRKGLLVIVDNTFCSPYLQRPLELGCDAVVHSATK  207

NM1.Methionine gamma-lyase  FIGGHGDVVAGIVVGDKEVISVLRKTTQKDIGGILSPFDAWLLLRGLKTLAVRMDRHCENAEHIAKQLSL  280
NR1.Methionine gamma-lyase  YIGGHGDVVAGVTICKTKALAEKIRPMRKDIGGIMAPFDAWLLLRGLKTLAVRMDRHCDNAEKIVSFLRK  277

NM1.Methionine gamma-lyase  HPKVKAVYYPGDKNSTAYSLMKKQMKKGGGLLSFEVEGGYKETVKVVNQLKLISIAVSLGDAETLIQHPA  350
NR1.Methionine gamma-lyase  HDAVEGVWYPEGE------LASRQMKRGGGVISFSVKGGKEETQAFINDLHFITIAVSLGDTETLIQHPA  341

NM1.Methionine gamma-lyase  SMTHAVVPEEARKEMGISNELLRLSVGLEAWEDIMRDLQQALDSI-----                     395
NR1.Methionine gamma-lyase  TMTHAAIPAELRKEMGIFDNLIRLSVGLESWEDIVSDLEQALKKISTVNQ                     391
```

Methionine gamma-lyase

```
NM1.Methionine gamma-lyase  MNEKKKYQFETKAIHAGYESKHHFDSLAPPIYQTSTFTFSSLEQGANRFSGEENGYVYSRLSNPTVTILE  70
NR1.Methionine gamma-lyase  ---MKKKHMETALIHHGYKPEEYKGSLTPPLFQTSTFTFETAQQGEASFAGVDPSYIYSRLGNPTVKLFE  67

NM1.Methionine gamma-lyase  ERMAQLEEGEAALAFGSGMAAVSAVLIGLTKAGDHILCSKGVYGCTFGLLEMLEEKYQIHHSFSNLETEE  140
NR1.Methionine gamma-lyase  ERMAVLEGGEEALAFGSGMAAISATLIGFLKAGDHIICSNGLYGCTYGFLEVLEEKFMITHSFCDMETEA  137

NM1.Methionine gamma-lyase  EILAAIKEDTACIYIETPINPTMTLVDLELVTCIAKQKGIPVVVDNTFSTPYLQQPLKLGCDLVIHSATK  210
NR1.Methionine gamma-lyase  DIENKIRPNTKLIFVETPINPTMKLIDLKQVIRVAKRKGLLVIVDNTFCSPYLQRPLELGCDAVVHSATK  207

NM1.Methionine gamma-lyase  FIGGHGDVVAGIVVGDKEVISVLRKTTQKDIGGILSPFDAWLLLRGLKTLAVRMDRHCENAEHIAKQLSL  280
NR1.Methionine gamma-lyase  YIGGHGDVVAGVTICKTKALAEKIRPMRKDIGGIMAPFDAWLLLRGLKTLAVRMDRHCDNAEKIVSFLRK  277

NM1.Methionine gamma-lyase  HPKVKAVYYPGDKNSTAYSLMKKQMKKGGGLLSFEVEGGYKETVKVVNQLKLISIAVSLGDAETLIQHPA  350
NR1.Methionine gamma-lyase  HDAVEGVWYPEGE------LASRQMKRGGGVISFSVKGGKEETQAFINDLHFITIAVSLGDTETLIQHPA  341

NM1.Methionine gamma-lyase  SMTHAVVPEEARKEMGISNELLRLSVGLEAWEDIMRDLQQALDSI-----                     395
NR1.Methionine gamma-lyase  TMTHAAIPAELRKEMGIFDNLIRLSVGLESWEDIVSDLEQALKKISTVNQ                     391
```

Sulfate ABC transporter substrate-binding protein

```
NM1.Sulfate ABC transporter substrate-binding protein  MKKKAVQFLMVLTALLVVLAGCGNGKSTETAGKDSSASKNDSKKPVELLNVSYDPTRELYQEFNKSFASY  70
NR1.Sulfate ABC transporter substrate-binding protein  MKKWGKTAFMKSTGVLLSAVVLLSGCGSATSTSNSEGSGD--KKTVELLNVSYDPTRELYQDFNKDFAKY  68

NM1.Sulfate ABC transporter substrate-binding protein  WKDKTGQDVTIQQSHGGSGKQGRAVIDGLEADVVTLALAYDIDEIAQTRQLLNKDWEKELAHNSTPYTST  140
NR1.Sulfate ABC transporter substrate-binding protein  WKEKHGQTVNVKQSHGGSGSQARSVIDGLEADVVTLALAYDIDAISKKKQLA-EDWQKRLANNSTPYTST  137

NM1.Sulfate ABC transporter substrate-binding protein  IVFLVRKGNPKGIKDWDDLTKKGTSVITPNPKTSGGARWNYLAAWAYAKDKYNGDNKKIEDFMSKLYGNV  210
NR1.Sulfate ABC transporter substrate-binding protein  IVFLVRKGNPKGIKDWDDLTKKGVSVITPNPKTSGGARWNYLAAWGYALKKYNNSEDKAKEFVSQIYKNV  207

NM1.Sulfate ABC transporter substrate-binding protein  EVLDSGARGATTTFVERGIGDVLIAWENEAYLSLNELGKDKFEIVTPSLSILAEPPVAVVDKVAKKKGTT  280
NR1.Sulfate ABC transporter substrate-binding protein  EVLDSGARGATTTFVEKGIGDVLIAWENEALLSQKELGKDKFEIVTPSISVLAEPPVAVVDKVVDKKGTK  277

NM1.Sulfate ABC transporter substrate-binding protein  KVAKAYLKYLYTEKGQEIAAENYYRPRNKKVLEKHKDQFPSLNLVTIKD-FGGWKKAQETHFNDGGTFDQ  349
NR1.Sulfate ABC transporter substrate-binding protein  KVAEGYLEYLYSEKGQEIAAKNFYRPRNEKVAEKYTSQFPKVQLFTVDELFGGWKKAQEKHFNDGGVFDK  347

NM1.Sulfate ABC transporter substrate-binding protein  IYQPK                                                                   354
NR1.Sulfate ABC transporter substrate-binding protein  IYQK-                                                                   351
```

LLM class flavin-dependent oxidoreductase

```
NM1.LLM class flavin-dependent oxidoreductase  -----------MKFALFSLIQNIPNPVTGETLTAQEKFQHVLNQAVLAEKLGFDAYGVGERHGAPFLSSS  59
NR1.LLM class flavin-dependent oxidoreductase  MEKYRIDTRKGIEFGLYSIGDHVLNPHNGDKITPEKRIHELIETAKLADEAGLDVFAVGESHQTHFTTQA  70

NM1.LLM class flavin-dependent oxidoreductase  PPVVLSAIAAKTSHIRLLTTVTVLSILDPVRVAEDYATLDHLSGGRLELIIGKGNDPRHYPLFGITEEEQ  129
NR1.LLM class flavin-dependent oxidoreductase  HTVILGAVAQATKNIKIASSATILSTSDPVRVYEDFATIDLISNGRAEIVAGRGSRIGGYSLLGYDVNDY  140

NM1.LLM class flavin-dependent oxidoreductase  WESLAERYTLLKQLWTEENVTWSGRYRPPLTNVTTQPRPFQPSIPVWHGSASSPLSTELAAKYGEPLFSS  199
NR1.LLM class flavin-dependent oxidoreductase  EELFEEKMDLLLKINNEEHVTWNGQFRAPLAHASVIPRAKNNNLPIWRAVGGPPASAIKAGRAGVPMMIT  210

NM1.LLM class flavin-dependent oxidoreductase  NSFHPQAKYKALIDHYRERFAYYGHDPSKAIVGSGASSLYISDTTEEAIRRYRPYYNA-FSNTEAAKHNQ  268
NR1.LLM class flavin-dependent oxidoreductase  TLGGPAINFKVSVDAYREAAQQSGFDPASLPVAT-TSLFYTAKNSQDALSEYYPHINAGMLTLRGDGYPK  279

NM1.LLM class flavin-dependent oxidoreductase  SPFTSLEDIVQHGPALVGSPEQIIEKIIDYHHAYGN--EVLSISVDGLSEAEQREQLERFASDIAPVLRK  336
NR1.LLM class flavin-dependent oxidoreductase  QQFT--NAIDYRDALMVGSPQQIIEKMLYQYELFGQQRFMAQIDFGGVPFDKIEKNIELIATEILPAVRK  347

NM1.LLM class flavin-dependent oxidoreductase  EIPGSVWENEKRGQGQDITK                                                    356
NR1.LLM class flavin-dependent oxidoreductase  HTAK----------------                                                    351
```

PepSY domain-containing protein

```
NM1.PepSY domain-containing protein  MQEKREESASTTKKSTARSQGLYKAIWRWHFYAGLMFTPFLLILAVTGGMYLFKPQIEEKLYHDFYH-VQ  69
NR1.PepSY domain-containing protein  ---------------MKVNRSLHYILWRWHFYAGLFITPLLITLSLSGIGYLFREEVEDFIYKDLYFGKS  55

NM1.PepSY domain-containing protein  AQSQSVSPSAQVQAVKEKYPDADVLTYKPSDRSTRSSEVGISLKDHTYTMFVNPHNGHIIGKVDDSSRLM  139
NR1.PepSY domain-containing protein  AQTESISMSDSISLTEKKYPHYSVAKISEFNGDYNTRLTIANEYTGQQKYVYLDSNNQIVGDQNASETFA  125

NM1.PepSY domain-containing protein  NQIEEFHGELMAG-TAGDRIVELAACWAIVLIVTGAFLWWPRKKDKIKGVLIPRFSKGKNVLIRDLHAVP  208
NR1.PepSY domain-containing protein  NIMRELHSSLLVGGTVVNYTVELAACWTIFLIVTGLYMSIRQFKN--------TPSSNKREKAKRRHSII  187

NM1.PepSY domain-containing protein  AFWISAGMLFLVLTGLPWSGLWGNAFQQVATNAGVGYPPSIWVGSAPTSTVQTKDVADVPWGAETLEVPS  278
NR1.PepSY domain-containing protein  GIIFTIPLFLLVASGLPWSGFMGNQIYKIASSNESLGYPKLYMAPPES------KVKELPWATRKEAPPE  251

NM1.PepSY domain-containing protein  STASQYTKVSLDDIVGIAKEQHMHDGYTISIPQDAQGVYTLSVFSPR--------AQDEATIHLDQYTGA  340
NR1.PepSY domain-containing protein  SNSNEPKAISVDELQ---KGIEIKKPYVISLPADPKGVFTVSKSSGSGITGMHVAPNEEITAYFDQYSGE  318

NM1.PepSY domain-containing protein  VLADYRYDNYGFMGKLIALGITLHKGTQFGFINQLMGLIICIGIAGIAISGSLLWWKRKPAKNMGAP-KV  409
NR1.PepSY domain-containing protein  LISKTDYRDYGLLAQWFTYGIPLHEGHLFGWPNKILCLLTTLSLLLLIYYGIKMWLARKPKGKLAAPPKQ  388

NM1.PepSY domain-containing protein  PEGSAMRIVTCIIVVFGILFPLVGLSLVIVWLLDFFVIKRIPALKRFLNA                    459
NR1.PepSY domain-containing protein  RDKKSIFVFFIMMVILGAVMPLFGLSVLVIFTIELLIYVFLKIRS-----                    433
```

Sulfurtransferase TusA family protein.1

```
NM1.Sulfurtransferase TusA family protein.1 --MESNKVLDAKGLACPMPIVKTKKAMNELEPGQVLEIHATDKGAKNDLTAWAKSGGHELLQTE-EGDVL 67
NR1.Sulfurtransferase TusA family protein.1 MSIKVDMSLDCKGLACPMPIVKTKKAIEGLAPGQVIEVKATDKGSTVDIKSWASKVGHQYIGTKHEGDVL 70

NM1.Sulfurtransferase TusA family protein.1 KFWIKKG--------------------------------------------------------------- 74
NR1.Sulfurtransferase TusA family protein.1 VHYVRKANEHEVNEVVKYPHTITNAELQSILSHGEESIVLDVREAAEFAFGHIPSAISIPLGELDSAVLD 140

NM1.Sulfurtransferase TusA family protein.1 ----------------------------------------------                         74
NR1.Sulfurtransferase TusA family protein.1 ETKQIYVICRTGNRSDVACQMLKEKGFSNVKNVIPGMLGWQGNVEK                         186
```

Sulfurtransferase TusA family protein.2

```
NM1.Sulfurtransferase TusA family protein.2 MMQANFILDAKGLACPMPIVKTKKMNELEAGQVLEIQATDKGSTADLQAWAKSTGHEYLGTEAAGDVLH 70
NR1.Sulfurtransferase TusA family protein.2 MMNIKQVLDAKGLACPMPIVRTKRAMDTLQTGEVLEIHVTDKGSVKDIPAWANKCGHDIVKLEEEGDVLK 70

NM1.Sulfurtransferase TusA family protein.2 HFLRKGGAEENVTPIPEISLEEFAKKVENDKHLHILDVREVEEYDKAHIPGVVHIPLGEVEKRFNELNKE 140
NR1.Sulfurtransferase TusA family protein.2 FWIRKA---------------------------------------------------------------- 76

NM1.Sulfurtransferase TusA family protein.2 DEIYIICHSGRRSEMAGQTMKKQGFKHLINVVPGMRDWTGKVE                            183
NR1.Sulfurtransferase TusA family protein.2 -------------------------------------------                            76
```

Rhodanese-like domain-containing protein.1

```
NM1.Phosphoadenylyl-sulfate reductase MADLYTYENWSDTFPEFDSKDETKGALSALQWAYDTYGDSIIYASSFGIEGIVLIDLISKVKKDAEIVFL 70
NR1.Phosphoadenylyl-sulfate reductase ---MLTYETWEENVVSFSEEDETKGALSVLNWAYKEYKDEVVYACSFGVEGMVLLHIINQVNPSAKVVFL 67

NM1.Phosphoadenylyl-sulfate reductase DTGVHFKETYEVIDAIKERFPDLRIHMKKPDLTLAEQAEKHGDELWKSQPNLCCQIRKIIPLRESLAPYD 140
NR1.Phosphoadenylyl-sulfate reductase DTNVHFQETYELIRKVRERFPSLNIIEKQPELTLDEQAKLHGEKLWESNPNLCCKIRKILPLEKSLVVEK 137

NM1.Phosphoadenylyl-sulfate reductase AWISGLRREQSESRANTNYFNKDEKFKKVKVCPLIHWSWKEIWRYVYKHDLPYNKLHDQGYPSIGCEPCT 210
NR1.Phosphoadenylyl-sulfate reductase AWISGLRREQSETRKHTKFINQDHRFQSIKVCPLIHWTWKEVWRYVYKHSLPYNPLHDVGYPSIGCEKCT 207

NM1.Phosphoadenylyl-sulfate reductase APAYNVDDLRSGRWAGTQKVECGLHES                                            237
NR1.Phosphoadenylyl-sulfate reductase LPVGDGGDSRDGRWAGKVKTECGLHYQ                                            234
```

Rhodanese-like domain-containing protein.2

```
NM1.Rhodanese-like domain-containing protein.2  MIINALIIVFLLWFLYQRFATVKGIQQIATTELKAKLKNKDNQ-FIDVRTPHEFRTKHIKGFRNIPLSEL  69
NR1.Rhodanese-like domain-containing protein.2  ------------------------MKEMTTKELEEKLLRKEVVNIVDVREVEEVAEGKIPEACNIPLGLL  46

NM1.Rhodanese-like domain-containing protein.2  PAQTGQLSKDREVVVICQSGMRSMKASKLLKKQGFIAITNVKGGMNTWR----                   118
NR1.Rhodanese-like domain-containing protein.2  EFRMHELNKNQEYIIVCRSGGRSARAVQFLESYGFQVIN-VVGGMLAWEGKVE                   98
```

Rhodanese-like domain-containing protein.3

```
NM1.Rhodanese-like domain-containing protein.3  MSPITAVLILLGVIII---YSVVMFFIQRRIMKTLTEEEFRSGYRKAQLVDIREPNEFENGHILGARNIP  67
NR1.Rhodanese-like domain-containing protein.3  MS--TTWIILLAVIVAFIGYTVWMYFYQKKLIKTLTEEEFRAGYRKAQLIDIREADEYNAGHILGARNIP  68

NM1.Rhodanese-like domain-containing protein.3  LSQFRMRIKEFRPDQPVYIYCQSGMRTGRAAQMLRRHGVQEIYDLKGGFKMWNGKVKKKK            127
NR1.Rhodanese-like domain-containing protein.3  LSQIRLRHKELRQDQPVYLYCQSGFRTGRAAQYLKKQGYKDFYQLQGGFKSWTGKIKKK-            127
```

Rhodanese-like domain-containing protein.4

```
NM1.Rhodanese-like domain-containing protein.4  MEEVKVITPEELQKRVENGEELHLVDVREDEEVAEGMIPTAKHIRMNDIPTNVDYFDKEKEYIFICRSGR  70
NR1.Rhodanese-like domain-containing protein.4  MTEVKTITTEEVQERLENGETLFLVDVREDEEVAAGKIPEAVHIKMGDIPHKVDFFNKENEYIFICRSGM  70

NM1.Rhodanese-like domain-containing protein.4  RSENVCYYLQEQGYKVTNMVGGMLEWEGKTVNK                                      103
NR1.Rhodanese-like domain-containing protein.4  RSENVCHYLNEQGFKTVNMVGGMLQYEGETK--                                      101
```

Bifunctional cystathionine gamma-lyase

```
NM1.Bifunctional cystathionine gamma-lyase  MKRKTQLIHGGIVGDEQTGAVSVPIYQVSTYKQEGAGKHTGYEYSRTGNPTRHALEELIKEIEGGYAGFA  70
NR1.Bifunctional cystathionine gamma-lyase  MRAKTKLIHGIRIGEPSTGSVNVPIYQTSTYKQEAVGKHQGYEYSRTGNPTRAALEEMIAVLENGHAGFA  70

NM1.Bifunctional cystathionine gamma-lyase  FGSGMAATTAVFMLFNSGDHVLITDDVYGGTYRVITKVLSRIGIEATFIDTSDIQNIEKEIRPNTKAIFI  140
NR1.Bifunctional cystathionine gamma-lyase  FGSGMAAITATIMLFSKGDHVILTDDVYGGTYRVITKVLNRFGIEHTFVDTTNLEEVKEAIRPNTKAIYV  140

NM1.Bifunctional cystathionine gamma-lyase  ETPTNPLLKITDLQHASSVAKQHHLLTIVDNTFSTPYWQNPIEKGADIVLHSATKYLGGHSDVVAGLAVV  210
NR1.Bifunctional cystathionine gamma-lyase  ETPTNPLLKITDIKKISTLAKEKDLLTIIDNTFMTPYWQSPISLGADIVLHSATKYLGGHSDVVAGLVVV  210

NM1.Bifunctional cystathionine gamma-lyase  NSPKLAEDLHFIQNATGGILGPQDSWLLMRGIKTLGIRMEEHEFNTGKIVEFLLAHPAVAKVYYPGLKTH  280
NR1.Bifunctional cystathionine gamma-lyase  NSPQLAEDLHFVQNSTGGILGPQDSFLLLRGLKTLGIRMEEHETNSRAIAEFLNNHPKVNKVYYPGLESH  280

NM1.Bifunctional cystathionine gamma-lyase  PNHLIAKKQARGFGGMVSFDVGSEEKAEEVLSKVKYFTLAESLGAIESLISIPSKMTHASIPSDRRKELG  350
NR1.Bifunctional cystathionine gamma-lyase  QNHELATEQANGFGAIISFDVDSEETLNKVLEKLQYFTLAESLGAVESLISIPSQMTHASIPADRRKELG  350

NM1.Bifunctional cystathionine gamma-lyase  ITDGLIRISVGLEDVEDLIEDLERALE                                            377
NR1.Bifunctional cystathionine gamma-lyase  ITDTLIRISVGIEDGEDLIEDLAQALA                                            377
```

Aliphatic sulfonate ABC transporter substrate-binding protein

```
NM1.Aliphatic sulfonate ABC transporter substrate-binding protein  MKKLLLTAAIVLVLALAGCGTKEKASGEDSKNVTINIGIQQSLGPLMLAQNQKWFEKEFKKIGVNVKWTE  70
NR1.Aliphatic sulfonate ABC transporter substrate-binding protein  MYKKFKILSFALAISVCLLGCEKSTASSKKEDVTIQIGIQQGLSPLLLAKKKGWFEEEFKKEGVKVKWTE  70

NM1.Aliphatic sulfonate ABC transporter substrate-binding protein  FQSGPPQFEGLASGHLDFGQVGNSPVISGQGADIPFLEIANSSDGLKGNAILVGKNSKIKSVKDLKGKKI  140
NR1.Aliphatic sulfonate ABC transporter substrate-binding protein  FQSGPPYFEAIASNRLDFGEVGNSPVISAQAAGIGFTEIANTSYARKGTGILVQKDSKIASVKELKGKKI  140

NM1.Aliphatic sulfonate ABC transporter substrate-binding protein  AVAKGSSGFNLLYRALDQNGLKPSDVKIIQLQPDEAQPAFENGSVDAWSIWEPFISLQNLKNEARILADG  210
NR1.Aliphatic sulfonate ABC transporter substrate-binding protein  AVAKGSSAFNLLYRALDKEGIDAKEVNVIQLQPDEAQPAFESGSVDAWAIWDPFISLHTLNKGAKVIADG  210

NM1.Aliphatic sulfonate ABC transporter substrate-binding protein  DSLKVASPGFTIVREGFAKDHPELVVKFLQVYQKALEWQNEHFEESVDILAKQKNLDKDVVRQVLKNNPA  280
NR1.Aliphatic sulfonate ABC transporter substrate-binding protein  ETLNVSSPEFLITRTKFAKEHPELVEKFLKVYEKARVWQDANLDEAIKVYTSVKKIDAEIVKEVFNHDKP  280

NM1.Aliphatic sulfonate ABC transporter substrate-binding protein  YNRPTSKEIIAEQQRTADFQQSLGVIKKKIDTGDVVDNSFIEKALKEK                        328
NR1.Aliphatic sulfonate ABC transporter substrate-binding protein  ILVPVTKEIIAEQQKTADFQYKLGSIKKEIKAEKVVDNFFVEKALKAK                        328
```

Gamma carbonic anhydrase family protein

```
NM1.Gamma carbonic anhydrase family protein  MIYPYKEKMPNIAPSCFLADYVTITGDVTIGEESSIWFNTVIRGDVSPTIIGKRVNIQDQSTLHQSPNAP  70
NR1.Gamma carbonic anhydrase family protein  MIYPYKEKKPKIASSAFIADYVTITGDVYVGEESSIWFNTVIRGDVSPTIIGDRVNVQDQCTLHQSPQYP  70

NM1.Gamma carbonic anhydrase family protein  LLIEDDVTVGHQVILHSSIIRKRALIGMGSIILDGAEIGEGAFIGAGSLVPPGKKIPPNTLALGRPAKVI  140
NR1.Gamma carbonic anhydrase family protein  LILEDDVTVGHQVILHSCHIKKDALIGMGSIILDGAEIGEGAFIGAGSLVSQGKKIPPNTLAFGRPAKVI  140

NM1.Gamma carbonic anhydrase family protein  RTLTEEDLKDMQRIRTEYVEKGQYYKSIKKSDSSL                                      175
NR1.Gamma carbonic anhydrase family protein  RELTAEDRKDMDRIRTQYVEKGQYYKSLQK-----                                      170
```

Bifunctional oligoribonuclease

```
NM1.Bifunctional oligoribonuclease  MKAQILEAIKQFDTIIIHRHIRPDPDAYGSQCGLAELLKTSFPEKKVYVTGEEAESLKFLYRMDDIADDT  70
NR1.Bifunctional oligoribonuclease  MHEQILGAIKEFDTIIIHRHVRPDPDALGSQGGLGTILQESFPEKNIYTVGYNEPSLAYLRVMDDIQDSV  70

NM1.Bifunctional oligoribonuclease  YEDALVIVCDTANEARVSDQRYASGKMIIKIDHHPNQTPYGDLLWVETSASSTSEMIYEFYLEGKEKGLA  140
NR1.Bifunctional oligoribonuclease  YENALVIVCDTANQERVDDQRYTKGKMLIKIDHHPNEDPYGDITWVDTTASSTSEMIYEFYNYGKDKGLK  140

NM1.Bifunctional oligoribonuclease  LNDEGAKLIFTGIVGDTGRFLFPNTKPKTFCYASELIQYGFNFKDVYDQLYKTKENVVHLHGYVLQNFTM  210
NR1.Bifunctional oligoribonuclease  ITKEAARLILAGIVGDTGRFLFPNTTAKTLRYVSELVDMGVKFTDLYNEMYKTKEKIARLNGYILQNFTM  210

NM1.Bifunctional oligoribonuclease  SPAGVAHMKIPNEILRKYDVTPADASQLVGSLGHIDGVKTWVFFVEEENQIRVRLRSKELVINTIARKYN  280
NR1.Bifunctional oligoribonuclease  VEEGAAYIKLTKEVLEEFDVLPSEASGVVGALGNIDGLKAWVLFLEEDDVIRVRLRSKGPVINKLAMQYN  280

NM1.Bifunctional oligoribonuclease  GGGHPLASGASIDSWEEVEPLLLDLEELCSQK                                        312
NR1.Bifunctional oligoribonuclease  GGGHPMASGAKASSWEEADRLFADLREICK--                                        310
```

Adenylyl-sulfate kinase

```
NM1.Adenylyl-sulfate kinase  MSKSTNITWHDAGITKQERREQNNHHSFVLWFTGLSGSGKSTVANAVAKALFDKNIRNYVLDGDNVRFGL  70
NR1.Adenylyl-sulfate kinase  --MDTNITWHTASVSKDERRVKNGHHSFVIWFTGLSASGKSTVANAVARKLFEKNIGNYVLDGDNIRHGL  68

NM1.Adenylyl-sulfate kinase  NKNLGFSAEDRTENIRRIGEVSKLFVDSGQVVLTAFISPFQEDRAQVREILEGNEFLEVYVECPLEECEK  140
NR1.Adenylyl-sulfate kinase  NKDLGFSESDRMENIRRIGEVAKLFVDQGAVVLTAFISPFRVDRKQVRDLLAADEFIEIFVKCPIEECEK  138

NM1.Adenylyl-sulfate kinase  RDPKGLYKKARSGEIRDFTGIDSPYESPANPEVTINTSTQSVEECVQTVIEYLSNRKFI            199
NR1.Adenylyl-sulfate kinase  RDPKGLYKKARKGDIKDFTGIDSPYEEPEQAELIVETHKYSIEECAEQIVKYLQERSFI            197
```

Sulfate adenylyltransferase

```
NM1.Sulfate adenylyltransferase  MTTIQAHGGELINLYQPTYDYTSLTKEIEVDNMALSDLELIGIGAYSPITGFLGEKDYQSVVENMRLADG  70
NR1.Sulfate adenylyltransferase  MSIVN----ELVNRIDETYDVSQIEKEIKLDNIALSDLELLATGGYSPLTGFLGKEDYDSVVETLRLANG  66

NM1.Sulfate adenylyltransferase  TVWSIPITLPVTEEQAKELNIGDKVKLVQSGVTYGVLEVSEVYTPNKEKEAENVYRTAELAHPGVKKMME  140
NR1.Sulfate adenylyltransferase  SVWSIPITLPVTEKVAESLKAGEEVKLVNNGNIYGVIQIEDIFVPDKEKEALLVYKTTDEAHPGVKKLYE  136

NM1.Sulfate adenylyltransferase  RPNVYVAGPIVLVERTPKTRFEKYYLDPTETRAAFEERGWKTVVGFQTRNPVHRAHEYIQKTALEIVDGL  210
NR1.Sulfate adenylyltransferase  RPNVYVGGTIILTKRFENNQFPSYHLDPIETREAFKKRGWKTVVGFQTRNPVHRAHEYIQKSALEIVDGL  206

NM1.Sulfate adenylyltransferase  FLNPLVGETKSDDIPADIRMESYEVLLENYYPSDRVALAVFPAAMRYAGPREAIFHAMVRKNFGCTHFIV  280
NR1.Sulfate adenylyltransferase  FLNPLVGETKSDDIPADVRMESYEVLLQNYYPKNRVFLSVFPAAMRYAGPREAIFHALVRKNFGCTHFIV  276

NM1.Sulfate adenylyltransferase  GRDHAGVGDYYGTYDAQKIFSNFTADELGITPLFFEHSFYCKKCEAMASTKTCPHSKEDHMILSGTKVRE  350
NR1.Sulfate adenylyltransferase  GRDHAGVGDYYGTYEAQEIFTNFTIEELGITPLFFEHSFYCTKCEAMASTKTCPHGKEDHVILSGTKVRE  346

NM1.Sulfate adenylyltransferase  MLRNGEVPPSTFSRKEVIEVLIKGLAKQKVSSK                                      383
NR1.Sulfate adenylyltransferase  LLRNGEIPPSTFSRKEVVEVLIKGLKTEVVTE-                                      378
```

Phosphoadenylyl-sulfate reductase

```
NM1.Phosphoadenylyl-sulfate reductase  MADLYTYENWSDTFPEFDSKDETKGALSALQWAYDTYGDSIIYASSFGIEGIVLIDLISKVKKDAEIVFL  70
NR1.Phosphoadenylyl-sulfate reductase  ---MLTYETWEENVVSFSEEDETKGALSVLNWAYKEYKDEVVYACSFGVEGMVLLHIINQVNPSAKVVFL  67

NM1.Phosphoadenylyl-sulfate reductase  DTGVHFKETYEVIDAIKERFPDLRIHMKKPDLTLAEQAEKHGDELWKSQPNLCCQIRKIIPLRESLAPYD  140
NR1.Phosphoadenylyl-sulfate reductase  DTNVHFQETYELIRKVRERFPSLNIIEKQPELTLDEQAKLHGEKLWESNPNLCCKIRKILPLEKSLVVEK  137

NM1.Phosphoadenylyl-sulfate reductase  AWISGLRREQSESRANTNYFNKDEKFKKVKVCPLIHWSWKEIWRYVYKHDLPYNKLHDQGYPSIGCEPCT  210
NR1.Phosphoadenylyl-sulfate reductase  AWISGLRREQSETRKHTKFINQDHRFQSIKVCPLIHWTWKEVWRYVYKHSLPYNPLHDVGYPSIGCEKCT  207

NM1.Phosphoadenylyl-sulfate reductase  APAYNVDDLRSGRWAGTQKVECGLHES                                             237
NR1.Phosphoadenylyl-sulfate reductase  LPVGDGGDSRDGRWAGKVKTECGLHYQ                                             234
```

(Fe-S)-binding protein

```
NM1.(Fe-S)-binding protein  MTGLLWVNFLFFIIVTAYAISLFVYLIRTRIEYIQLGKKVEFDQRLKERLQKIWVNVFGQKKLLKDKKSG  70
NR1.(Fe-S)-binding protein  MNSLLIINWLAAIAVIAYAGYLFVYLIRTRMAYIQLGKKIEFDRRFKERWDLLKVNVFGQKKLLKDKKSG  70

NM1.(Fe-S)-binding protein  IMHVMFFYGFILVQFGALDFIIKGLLPGKHLPLGPLYPGFTFFQEIVTFLILVAVVWAFHRRYVEKLVRL  140
NR1.(Fe-S)-binding protein  IIHVMFFYGFILVQFGAIDFVWKGLAPGSHLPLGPLYPAFTFFQEIVTLVILIAVFWAFHRRYVEKLVRL  140

NM1.(Fe-S)-binding protein  KRGFKSGLVLIFIGALMLSVLVGNGMSIIWHNHEPAWTEPIASSIAILFKGINDTAAISIFYVSWWIHLA  210
NR1.(Fe-S)-binding protein  KRNFKSGLVLIFIGGLMISVLLGNGMGLIWHGEELSWSEPIASAIAYVFSGINETVAISVFYFSWWVHLL  210

NM1.(Fe-S)-binding protein  VLLTFLVYVPQSKHAHLLAGPANVFFNRVSKPGKLEKIDFEDETQETFGVGKIEEFTQHQLIDLYACVEC  280
NR1.(Fe-S)-binding protein  ILLTFLVYVPQSKHAHLIAGPANVFFGRLSNPGKLEKIDFEDETQETFGVGKIEDFRQNQLIDLYACVEC  280

NM1.(Fe-S)-binding protein  GRCTNMCPATGTGKILSPMDLILKMRDHLTEKGAVVTSKAPWVPTYAFSKTKGNQLAMMAASQGGAEAAA  350
NR1.(Fe-S)-binding protein  GRCTNMCPATGTGKMLSPMDLILKLRDHLTDKGAAVTSKAPWVPVVAFNNTQGNQLAMMAAGKGQQESAS  350

NM1.(Fe-S)-binding protein  T-AEYNPALIGDVITEEEIWACTTCRNCEDQCPVMNEHVDKIIDLRRFLVLTEGKMDADAQRAMTNIERQ  419
NR1.(Fe-S)-binding protein  TTLAYDPSLIGDVITEEEIWACTTCRNCEDQCPVMNEHVDKIIDLRRYLVLTEGKMDAEAQRAMTNIERQ  420

NM1.(Fe-S)-binding protein  GNPWGLNRKEREEWRHVREDVHIPTVKEVSKAGETFEYLFWVGSMGSYDNRSQKIALSFAKLLNEAGVSF  489
NR1.(Fe-S)-binding protein  GNPWGLNRKERETWRQGDDEVTVPTVKEKSKAGEEFEYLFWVGSMGSYDNRSQKIAISFAKLMNEAGISF  490

NM1.(Fe-S)-binding protein  AILGNKEKNSGDTPRRLGNEFLFQELASKNIAEFEKNEIKKIVTIDPHAYNIFKNEYPDFGFEGEVYHHT  559
NR1.(Fe-S)-binding protein  AILGNKEKNSGDTPRRLGNEFVFQEMATKNIEEFEKAGVKKIVTIDPHAYNTFKNEYPDFGLQAEVYHHT  560

NM1.(Fe-S)-binding protein  ELLAMLVKEGKLTPKHAVNEKITFHDSCYLGRYNEVYSPPREILKAIPGVSLIEMDRNRENGMCCGAGGG  629
NR1.(Fe-S)-binding protein  ELLAQWVKEGRLKPVHAIEETVTYHDSCYLGRYNEVYEAPRDILKAIPGVNLVEMARNRETGMCCGAGGG  630

NM1.(Fe-S)-binding protein  LMWTEETTGQRINVARTEQALAVNPSIISSGCPYCLTMLSDGTKAKEVEEKVHTYDVAELLEKSIYGDVQ  699
NR1.(Fe-S)-binding protein  LMWMEETTGSRINVARTEQALAVQPSIIGTGCPYCLTMISDGTKAKEVEEKVQTLDVTEILERSVIGQKK  700

NM1.(Fe-S)-binding protein  EQVS                                                                    703
NR1.(Fe-S)-binding protein  EAM-                                                                    703
```

Section 2

NM1-A2 Sulfur genes

NM1_A2_00094	cysK	100.0	312	0	0	1	312	1	312	2.3e-172	605.5
NM1_A2_02829	ssuD	100.0	356	0	0	1	356	1	356	3.0e-205	714.9
NM1_A2_04883	glpE	74.0	100	26	0	1	100	1	100	1.7e-36	152.5
NM1_A2_04969	cysH	100.0	237	0	0	1	237	1	237	1.6e-141	502.7
NM1_A2_01583	metB	68.1	392	125	0	1	392	1	392	1.0e-153	543.9
NM1_A2_03400	pspE	65.3	95	33	0	1	95	3	97	1.1e-29	129.8
NM1_A2_04894	cysE	75.6	168	41	0	1	168	1	168	3.3e-48	192.2
NM1_A2_04509	glpE	100.0	127	0	0	1	127	1	127	7.5e-66	250.4
NM1_A2_04905	ssuC	70.8	260	76	0	8	267	5	264	9.5e-95	347.4
NM1_A2_00856	MET17	62.5	429	151	3	7	426	5	432	1.4e-148	526.9
NM1_A2_03316	cysJ	99.6	459	2	0	1	459	1	459	4.0e-266	917.5
NM1_A2_05232	hdrD	79.1	702	143	3	1	702	1	698	0.0e+00	1133.2
NM1_A2_00911	ssuD	100.0	377	0	0	1	377	1	377	9.9e-207	719.9
NM1_A2_03187	cysI	100.0	573	0	0	1	573	1	573	0.0e+00	1159.4

NM1_A2_00928	ssuC	69.0	239	74	0	7	245	10	248	7.5e-8	317.8
NM1_A2_04876	cysK	100.0	310	0	0	1	310	1	310	1.1e-174	613.2
NM1_A2_02975	ssuD	99.7	356	1	0	1	356	1	356	6.5e-208	7.23.8
NM1_A2_04817	nrnA	100.0	312	0	0	1	312	1	312	2.3e-180	632.1
NM1_A2_04744	ssuD	100.0	374	0	0	1	374	1	374	1.3e-214	746.1
NM1_A2_03397	tusA	100.0	74	0	0	1	74	1	74	1.1e-37	156.0
NM1_A2_05315	nrnA	60.3	416	165	0	236	651	1	416	1.5e-138	494.2
NM1_A2_03188	cysJ	99.8	602	1	0	2	603	1	602	0.0e+00	194.9
NM1_A2_04622	mccB	100.0	377	0	0	1	377	1	377	2.6e-215	748.4
NM1_A2_02120	sat	66.0	309	103	2	48	354	496	804	9.3e-114	411
NM1_A2_03401	pspE	100.0	118	0	0	1	118	1	118	1.3e-59	229.6
NM1_A2_01901	metB	99.8	404	1	0	1	404	1	404	9.0e-230	796.6
NM1_A2_03403	tusA	100.0	75	0	0	1	75	1	75	4.8e-36	150.6
NM1_A2_00115	cysE	100.0	219	0	0	1	219	1	219	2.6e-122	438.7
NM1_A2_04747	ssuA	63.6	316	113	1	12	327	13	326	3.5e-107	389.0
NM1_A2_04746	ssuC	73.4	259	68	1	24	282	19	276	5.0e-102	371.7
NM1_A2_01472	metB	100.0	381	0	0	1	381	1	381	8.2e-217	753.4

NM1_A2_03399	tusA	100.0	183	0	0	1	183	1	183	3.8e-103	374.8
NM1_A2_04966	cysC	100.0	199	0	0	1	199	1	199	9.2e-111	400.2
NM1_A2_04745	ssuE	100.0	184	0	0	1	184	1	184	5.5e-94	344.4
NM1_A2_04967	sat	100.0	383	0	0	1	383	1	383	1.2e-223	776.2

NM1-A2 Nucleotide sequencing

>NM1_A2_00094 cysteine synthase A

ATGGCACGTATTGCAAATTCAATTACAGAGTTAATTGGTCAAACACCAATCGTAAAACTAAACCGTCTAGTTGAAGAA

GATATGGCGGATGTATATTTAAAATTAGAATTTATGAACCCAGGAAGCAGCGTAAAAGATCGTATCGCATTAGCAATG

ATCGAAGATGCTGAAGCAAAAGGTGTATTAAAGCCTGGAGACACCATCATTGAACCAACAAGCGGTAACACAGGAATT

GGCTTAGCAATGGTAGCTGCAGCAAAAGGATACCGTGCTATTTTAACAATGCCTGAAACGATGAGTATTGAGCGTCGT

AATCTGCTTCGTGCGTACGGTGCAGAGTTAGTGTTAACACCAGGTCCAGAAGGAATGAAAGGTGCGGTTAATAAAGCA

ACTGAGCTTGCAAAAGAGCACGGCTACTTTATTCCTCAGCAGTTCCAAAATGAAGCTAACCCAGAAGTGCACCGTCAA

ACAACGGCTAAAGAGATCATCGAGCAGTTTGGCGATCAGCTAGATGGATTTGTGGCAGGTATTGGTACTGGCGGTACT

ATTACAGGTGCTGGCGAAGTATTAAAAGAAAAGTATCCAAACATCAAAATTTATGCGGTAGAGCCAGCTGATTCTCCG

ATCTTAAGTGGTGGTCAACCAGGACCTCATAAAATTCAAGGAATCGGTGCAAACTTTGTTCCGGATACGCTTAACACGG

AAGTATATGACGAAGTTGTCGCTGTTCAAAACGACCAAGCTTTTGAATATGCACGTAAAGTAGCAAAAACAGAAGGCG

TACTAGTAGGTATCTCTTCTGGGGCTGCGATTTATGCTGCGTTAAAAGCTGCAAAACAGCTAGGAAAAGGTAAAAAAG

TGTTAGCGATTATTCCAAGTAACGGTGAGCGTTACTTAAGTACTCCATTGTTCCAATTCGAGGAACAAACACAAGCATA

A

>NM1_A2_00115 serine O-acetyltransferase

ATGATGCTAAAGTTGTTAAAGGAAGACATTGAGGCCATATTTGAACAAGATCCTGCAGCAAGAAGTTATATTGAAGTG

ATATTAACCTATTCAGGGTTGCATGCTATTTGGGCACATAGATTCGCTCACGGGCTTTTTAAAAGAAAGTGGTTTTTTCT

GGCACGTGTAATCTCACAAATTAGCCGATTCTTCACAGGGATTGAAATTCATCCGGGTGCGAAAATTGGCCGCCGCTTT

TTTATTGACCACGGAATGGGAATTGTTATTGGGGAAACGTGCGAAATTGGAGATAATGTAACAGTATATCAAGGAGTA

ACACTCGGAGGAACAGGGAAAGAAAAAGGAAAGCGTCACCCGACGATTGAAGACAATGTCTTAATTGCAACAGGTGC

TAAAGTGCTAGGGTCGATTACGATTCATGCGCACTCCAAAATTGGAGCAGGCTCAGTTGTGTTAAATGACGTCCCAGAA

AACTCAACGGTTGTTGGTATCCCTGGCCGAGTCGTCATTCAAAATGGCGTCCGGATTCCAAAAGATTTGAATCATCAAG

ATTTGCCAGATCCAGATGCAGAGCGTTTTAAACAGCTAGAGAAAGAAATTTTACACCTGCAAAATCAATTAAAGGAAT

TAAAAGAAGGGAAGATTCACCATGGCAATTAA

>NM1_A2_00856 homocysteine synthase

ATGTCTGCTAAGAAACCATTTCGTCCTGAAACACAAGCCATCCATTCTGGTCAACAGCTCGATCCTGCTACTTTTTCGCG

TGCCGTACCTATTTATCAAACAAGCTCATTTGGATTTAAAGATACGGAGCATGCTGCTTCTTTATTTAATTTAAGTGAAC

AAGGCTATATCTACACTCGAATTGTAAACCCTACTACGGACGTTTTTGAACAGCGAATTGCTGAATTAGAAGGCGGAGT

TGGTGCTCTAGGAGTAGCTTCGGGTCAGTCAGCTACTACCTTTTCCATTTTGAATATCGCTTCCGCAGGAGATGAAATTG

TATCAGCAAGCAGTTTATATGGGGGAACATATAACCTCTTTTCGTCTACGCTTCCTAAACTAGGCATTACTGTAAAATTT

GTAAACGCCGATAATCCAGAAAATTTTAGAAGTGCAATCACTTCCAAAACAAAGGCAATTTATGCTGAATCCGTTGGA

AATCCGCAGGGAAATGTGCTAGACATCGAGGCAGTTGCAGACATTGCCCACGAACATGGAATTCCTCTTATTATTGATA

ATACTGTTCCAAGCCCTTACCTTCTTCGTCCCATCGATTTCGGAGCAGATATTGTCGTTCATTCTGCTACTAAGTTTCTTG

GGGGGCATGGGACAGCAATTGGCGGTGTTATCGTAGACAGTGGAAAATTTGATTGGGAAGCAAGCGGGAAATTTCCAG

ACTTAACAACTCCAGATCCAAGTTACCATGGCCTCGTATACACAGAAGCCGCCGGTGAAGCTGCTTATATTACAAAAGC

GCGCGTTCAGCTTTTACGAGATATTGGCGCTGCTTTATCACCTTTTAATTCCTTTTTACTTCTTCAAGGAGTAGAAACAC

TCCATCTGCGATTAGAGCGTCACAGCGAAAACGCATTAAAGGTAGCGAAATTTTTAGAGCAGCATGAATTAGTAGACT

GGGTACATTATGCGGGGCTTCCTTCTCACCCTTCCTATTCATTGGCACAAAAATATTTGCCTAAAGGACAAGGAGCCAT

TTTAACCTTTGGTGTCAGGGGAGGAAAAAACGCTGCGGCTAAGTTAATTGATTCCGTTCAGTTATTTCTCATTTAGCCA

ATATCGGCGACTCGAAGTCACTCATTATTCACCCGGCAAGCACCACTCATCAACAGCTATCTGAAGATGAACAAAAAG

CTTCCGGCGTGACGCCAGAACTCATTCGTCTATCGGTTGGAACAGAAGCAATCGACGACCTTCTTGAAGATTTAGATTA

TGCCTTAAAAGCCAGCCAAAAAGTGAATACAACTGTTTAA

>NM1_A2_00911 FMNH2-dependent alkanesulfonate monooxygenase

GTGGAGTTATTTTGGTTTATTCCAACGTACGGAGATGGACGTTATTTAGGAAGCCATGAAGGAGCCAGGGCTGCAAGCT

ATTCTTATTGCAAACAAGTTGCACAAGCTGCGGATGAGCTAGGATATAGCGGGGTTTTGCTTCCGACGGGAAAGTCATG

CGAGGATGCGTGGATTGCTGCATCTACTTTAGTGCCAGTCACTGAAAATCTGAAGTTTTTAGTGGCGGTACGCCCCGGT

TTAATGTCCCCTACACAAGCCGCAAGAATGGCGGCAACATTTGACCGCTTTTCTAAAGGAAGGCTTCTTATTAATGTCG

TAGCAGGAGGAGATCCTGTGGAGCTAGAAGGAGACGGGGTGTTTTTGAACCATCACGACCGCTATGAGCTGACAGATG

AATTTTTAACGATATGGAGAAGGGTATTAAATGAATCCGATGTTCACTTCGACGGAGACTACTTGAGTGTTAAAGGTGG

AGACGTGCTTTATCCGCCGATCCAAAAACCGTATCCTCCGCTTTATTTTGGAGGTTCTTCTCCTGTAGCGATGGACGTTG

CCTCAAGGCATATAGACGTCTATTTAACTTGGGGAGAGCCACCTGCGCAAGTAAAAGAAAAAATTGAACGAATGAAAG

AAAAAGCTAGGCAGACAGGAAGAGAAATTCGATTTGGCATCAGGCTTCATGTCATTGTTCGTGAAACTGAAGAACAAG

CCTGGAAAGCGGCAGATGAACTGATTCAACATGTAGATGAAGAAGCCGTTAAATCAGCCCAAAAAGTATTTTCTCGAA

TGGATTCAGAAGGTCAAAAACGTATGAGCGCCTTGCATCAAGGCGACCGTTCAAATTTAGAGGTAAGCCCTAATCTAT

GGGCTGGTGTAGGGTTAGTTCGAGGAGGCGCAGGGACTGCTTTAGTAGGGGCTGCTGATACGGTGGCACAGCGAATCA

AAGAATATGCTGAGTTAGGTATCGAAACATTTATTTTATCTGGATATCCTCATTTAGAAGAAGCATATCGAACAGCAGA

GCTTTTGTTTCCAAGACTTCCAGTTCAGCGTAAGCAAGATGAGAACGAGCGCACATTTATCAGTCCATTTGGTGAAGTG

AAAGTGAATGATAAAGCACCCGCTAAAAGGTGA

>NM1_A2_00928 ABC transporter permease

ATGAAAAAGTACGGGGCTTCAGTGATTTTAGTAGTCATTTTGCTGGCTGCTTGGGAAATTGGAGCACGCATTGTAAACT

ATCCGTTTATTTTACCAACGCCCAGCGGTATTTTAACGAAGCTTTGGGAGCTGCGAATGGATTTATTGTTTAAGCATTTA

CCAGCTACGTTATCTATTGTCGTAATTGGGCTTGTAATATCCGTTGTGCTAGGGGTTCTGCTGGCTGTGTGGATGAATTG

GAGTCCGCTTATTGAGCGTGCTTTTTATCCGCTTATTATTGCGTCACAAATGATTCCGACCATTGCCATTGCTCCTGTATT

TGTGCTGTGGTTTGGCTACTCCATTTGGAGCAAAGTGATTGTGACCGTATTAATTACGTTTTTTCCCATTACCGTTAGCA

CATTTGACGGGCTTCGTTCTACAAACAAAGAGTTAAAAGAACTCATGTTAACGATGGGAGCAACAAAAAAAGATATTT

TCTTTAAGTTGAACATTCCTTCTGCACTGCCTCATTTTTATTCGGGACTCAAAGTAGCGGTTACCTTTAGTATAATAGGT

GCTGCCATTGGGGAATGGTTGGGTGCGCAAGCAGGCTTAGGATATTTCAGTCGTCGCATGATGACACAGTTTGATGCAG
CTGGTGTATTTGCACCAATTGTGATTTTATCCGCGCTCGGTATTTTATTTTTATTATTGTAGTAGGTTTCGAAAAACGTT
CATTAAAGTGGAGGAAAACAGAATGA

>NM1_A2_01472 PLP-dependent transferase

ATGAAAATTGAAACTTTATTAGTTCGTTCAGGAGTAGGTCGTGACCCTTCAACAGGTTCTATTACAACACCCATTTACC
AAGCATCAACATTTGCTCATCCTGCTTTAGGGCAAAGTACAGGATTTGACTATGCACGTACGGCAAATCCTACTCGTAC
GGCATTGGAAGAAGCAATTGCTGCTTTAGAAAAAGGCGAAGTAGGAGTAGCATTCGCTTCAGGAATGGCGGGTGTAAT
GTCAGTTTTAGCGCTATTTAAAAACGGAGATCATTTAATTGTATCAGAGGATTTATACGGAGGAACGTACCGAGTACTA
AATGAAATCTTTTCAGAGCAAGGAATTACTGTTTCGTACGTCAACACAGCCCATCTCGAGCAAGTAAAAGATGCGCTTC
GTCCCAACACGAAAGCTCTATTCATTGAAACACCTACAAATCCAATGATGCACGTGACTGATTTGCCAGAAGCAATTGC
GTTAGCTAAACAGCATGATTTGCTTACTATCGTTGACAATACATTCATGTCCCCTTACTATCAGCGTCCTTTAGAGCTCG
GAGCAGATATTGTGATACATAGTGCAAGCAAATACATTGGGGGTCATAATGATGTAGTTGCTGGTCTTGTAGTCGCTCG
ATGTAGTAGCTTAGGAGAGAAAATTCGTTTTTATCAAAATGCAGCTGGGGCAATCTTAGGTCCACAGGATAGCTGGCTG
TTATTGCGCGGTATTAAGACGCTAGCGCTTCGTATGGAAAAACATAACGAAAACGCACTGAAAATTGCAAATTGGCTT
ACTGCTCATGATCTCGTGGAAAAAGTGTATTACCCTGGTCTTGAAACACATCCGGGCTATGAAATCATGAAGAAACAA
GCAACTGGATTTGGAGGCATGATTTCTTTTGCTGTCTCACACCCTGACATCGTGTCAGTACTTTTAGAAAATGTTAAAGT
TATTACATTTGCAGAAAGCTTAGGAGGCGTGGAAAGTTTAATGACATTCCCAGCTCGTCAAACACACGCTGATATTCCG

GAGGAAATTCGAAATCGTGTCGGAGTAACAAACTGTTTACTTCGATTATCAGTTGGAATTGAGCACGCAGATGATTTAA

TTAAAGATTTGAAGGAGGCATTTGATGCATACCAACAATAA

>NM1_A2_01583 methionine gamma-lyase

ATGAACGAAAAAAAGAAATATCAGTTTGAAACAAAAGCCATTCACGCAGGGTACGAATCAAAACATCATTTTGATAGT

TTAGCTCCACCTATTTATCAAACGTCTACATTTACATTTTCATCTCTAGAACAAGGTGCAAACCGATTTAGCGGAGAAG

AAAATGGTTATGTATATTCTCGGTTATCTAATCCGACGGTCACCATTTTAGAAGAGCGTATGGCACAGCTTGAAGAAGG

CGAAGCCGCTTTGGCATTTGGATCAGGAATGGCTGCAGTATCGGCTGTATTGATTGGACTTACAAAAGCAGGCGATCAT

ATTTTATGTTCTAAAGGAGTGTATGGATGCACGTTTGGTTTACTGGAAATGCTTGAGGAAAAATATCAAATCCATCATT

CTTTTTCCAACTTGGAAACTGAGGAAGAAATTCTTGCTGCTATCAAAGAAGATACAGCATGTATTTATATTGAAACACC

GATTAACCCGACTATGACTTTAGTAGATTTAGAGTTAGTGACTTGTATCGCCAAACAAAAAGGAATTCCCGTGGTAGTA

GACAATACCTTTTCAACTCCTTATTTGCAGCAGCCGCTGAAGCTTGGATGCGACTTAGTTATTCATAGCGCAACTAAATT

TATTGGTGGACACGGAGATGTTGTGGCAGGAATTGTAGTAGGGGACAAAGAGGTTATTTCCGTACTGCGCAAAACGAC

GCAAAAAGATATTGGAGGTATTCTTTCACCATTTGATGCGTGGCTGTTACTCCGAGGCTTAAAAACACTGGCCGTTCGA

ATGGATCGCCACTGTGAGAATGCTGAACATATCGCAAAACAATTAAGTCTCCATCCCAAGGTAAAAGCCGTTTATTATC

CAGGGGATAAAAACAGCACTGCTTATTCTCTCATGAAAAAGCAAATGAAAAAAGGAGGGGGGCTTCTCTCTTTTGAAG

TGGAAGGCGGTTATAAAGAAACCGTAAAAGTGGTGAATCAGCTTAAGCTCATTTCCATTGCGGTGAGTCTTGGGGATG

CTGAAACACTTATTCAGCATCCTGCCTCGATGACGCATGCAGTTGTGCCGGAAGAAGCTCGAAAAGAAATGGGCATCT

CAAATGAACTTCTTCGTTTATCAGTTGGGCTGGAAGCTTGGGAAGATATTATGAGAGATTTACAGCAGGCATTAGATAG

TATATAA

>NM1_A2_01901 cystathionine gamma-synthase family protein

ATGACAGACAAACAGTTTAGAAATGTACAAGACGGCACAAAAGCGGTATGGGCAGGAGAAAAAGAATCGCTTGCTTA

CAATGCCACTCAAGTTCCCGTCGTCTTCAGCGTAGCTTACAATTATGATGATGTAGACGAATGGCAAGAAGTTGCGTTA

GGAAACAAACCAGGCTATACGTATAATCGCATGAGTAATCCTACGGTTAAAGCTTTTGAAGAAAAAGTAAGGATACTT

GAAGAAGCAGAAGAGTCGGTCGCATTTTCCTCCGGCATGGCGGCTATTAGCAGCACATTGTACACTTTTTTAAAACCCG

GAGACCGAGTCGTATCGGTAAAAGATACATATGGAGGAACAAATAAAATCTTCACTGAGTTCCTTCCTAACATTGGAG

TGGATGTAACCTTATGTAATACGGGAAATCATGAAGAAATTGAAGCGGAAGTAAATAAAGGATGTAAAGTGCTGTATT

TAGAGTCACCTACAAATCCTACGATGAAAATTATTGATATTGAAAGAATAGCGAAAGCAGGAAAATCGGTAGGGGCAG

TGGTCATTATTGATAATACGTTTGCGACACCAATTAACCAAAACCCTCTTCAATTAGGAGTAGATCTTGTCATTCACAGC

GCCACAAAATTTTTAAGCGGACACGCCGATGCTTTAGGCGGGGTAGTATGCGGCTCTAAAGAATTAATGCAGCATGTGT

ATCACTACCGTGAGATCAATGGCGCGACCATGGACCCTATGTCAGCGTATCTTATTTTAAGAGGAATGAAGACGTTAAA

GCTGCGTATACGTCAACAAGAACGAAGCGCTTTGGAAATTGCTAAATTTCTTCAAAAGAAAGAAGCTGTTGAAGCCGT

CTATCATCCTGGATTGGAAACACACCCTCATCATCATATTGCCAAAAAACAAATGAAAGGCTTTGGCGGAATATTAAGC

TTTGTGTTAAAAGGAGAAATGGAAGCTATTAAAATTTTGCTGCCAAAGTTAACGTATGCAAATAAAGCCGGTAATTTAG

GAGCAGTTGAAACGATTTACGGTCCGGCAAGAACGACTAGTCACGTAGAATGTACGCTTGAAGAACGTAAAGCTCTTG

GAATATCCGAAGGACTGGTTCGCATATCTGTAGGAATTGAAGATACGGAAGATTTAATTGCAGACTTAGAACAAGCAT

TTGCTCACTTAGAATCTGCATCACCTATTAGTAATTAA

>NM1_A2_02120 sulfate ABC transporter substrate-binding protein

ATGAAGAAAAAAGCAGTTCAGTTTTTAATGGTGCTGACAGCACTGCTCGTGGTGCTAGCAGGTTGCGGCAATGGAAAA

TCTACTGAAACAGCAGGGAAAGATAGTTCTGCGAGTAAAAACGATTCAAAAAAACCAGTAGAACTTTTAAACGTTTCA

TATGATCCTACACGTGAGCTTTATCAGGAGTTCAATAAGAGCTTTGCGTCTTATTGGAAAGATAAAACAGGGCAAGACG

TCACTATTCAGCAATCACACGGTGGCTCTGGTAAGCAAGGCCGAGCAGTTATTGACGGTTTAGAAGCGGATGTAGTAA

CGCTGGCGCTTGCTTACGATATTGATGAAATCGCTCAAACTCGTCAATTATTAAACAAAGACTGGGAGAAAGAATTGGC

TCATAATTCTACACCATACACATCAACGATTGTTTTTTAGTTCGTAAAGGAAATCCAAAAGGAATTAAAGATTGGGAT

GATTTAACGAAAAAAGGTACATCTGTTATTACGCCAAATCCAAAAACTTCTGGAGGCGCGCGCTGGAATTATTTAGCTG

CATGGGCATATGCGAAGGATAAATATAACGGCGATAACAAAAAGATTGAAGACTTCATGAGTAAGCTTTATGGAAATG

TCGAAGTGTTAGACTCAGGCGCACGCGGAGCTACCACTACCTTTGTAGAGCGAGGCATTGGAGACGTGTTAATTGCATG

GGAAAACGAAGCTTACTTATCGTTAAATGAGCTAGGAAAAGATAAATTTGAAATTGTAACACCGTCATTAAGTATTTTA

GCAGAGCCACCTGTTGCTGTAGTAGATAAAGTAGCTAAAAAGAAAGGCACGACAAAGGTAGCAAAAGCATACTTAAA

ATACCTATATACAGAAAAAGGTCAGGAAATCGCAGCGGAAAACTACTATCGTCCTCGAAATAAAAAAGTATTAGAGAA

ACACAAAGATCAATTCCCTTCGCTTAACCTAGTCACTATTAAAGATTTTGGCGGCTGGAAAAAAGCTCAAGAAACGCAT

TTTAATGATGGGGGAACATTCGATCAGATTTATCAGCCAAAGTAA

>NM1_A2_02829 LLM class flavin-dependent oxidoreductase

ATGAAATTTGCTTTATTTAGTCTTATTCAAAATATCCCGAACCCGGTTACTGGAGAAACATTAACTGCTCAAGAAAAGT

TTCAGCATGTGTTAAATCAAGCAGTGCTGGCAGAAAAATTAGGTTTTGATGCTTACGGAGTCGGCGAAAGACACGGAG

CTCCCTTTTTGTCTTCTTCTCCTCCCGTTGTTTTAAGTGCAATCGCTGCTAAAACGTCGCACATTCGTCTTCTTACTACCG

TTACCGTTCTAAGCATTTTAGATCCTGTACGAGTAGCTGAAGATTATGCAACGCTTGATCACCTATCGGGTGGACGCCTT

GAACTGATTATCGGAAAAGGCAATGATCCGCGTCATTATCCGCTTTTTGGCATTACGGAAGAAGAACAATGGGAATCTC

TTGCTGAAAGATATACTCTTTTGAAACAGCTTTGGACAGAAGAAAATGTCACGTGGAGCGGCCGTTATCGTCCGCCTTT

AACTAACGTTACGACTCAGCCTCGACCGTTTCAGCCTTCTATTCCCGTTTGGCATGGAAGCGCGTCAAGCCCGCTGTCT

ACAGAACTTGCAGCTAAATATGGAGAGCCGCTATTTTCATCCAATTCCTTTCACCCTCAAGCTAAATATAAAGCGCTGA

TTGACCATTACCGCGAACGTTTCGCTTATTATGGTCATGACCCAAGCAAGGCTATCGTCGGTTCAGGGGCAAGCAGCCT

ATATATTTCTGATACAACGGAAGAAGCGATTCGCCGCTATCGTCCATACTATAACGCATTTAGCAATACAGAAGCAGCG

AAGCATAATCAATCGCCATTCACGTCACTAGAAGACATTGTTCAACACGGCCCTGCTTTAGTAGGAAGCCCAGAGCAA

ATCATTGAAAAAATAATAGACTATCACCATGCTTATGGAAACGAAGTGCTGAGCATCAGCGTAGACGGTCTAAGCGAA

GCAGAGCAGCGCGAACAGCTAGAGCGCTTTGCAAGTGATATTGCACCTGTTCTTCGAAAAGAAATTCCCGGCAGCGTG

TGGGAAAATGAAAAAAGAGGCCAAGGCCAAGATATAACGAAATGA

>NM1_A2_02975 FMNH2-dependent alkanesulfonate monooxygenase

ATGAACATCTTATGGTTTTTCCCTACAGCAGGCGACGGACATTATCTAGGCACAACCGAAGGAAGTAGAACAAGTGAT

ATTCACTACTTAAAGCAAATCGCACACGGGCTGGATTATCTAGGATATGACGGTGCTTTGTTGCCTACTGGTTCAAATT

GTGAAGATTCGTGGGTGATTGCATCGGCACTTGCTTCCGTTACAAACCGACTGAAGTTTTGATTGCACTTCGCCCGGGT

GTCATGTCTCCCACACTTTCAGCACGGATGGCTTCGACATTCGATCAAATATCAGACGGCCGACTTATGTTAAATATTGT

GACGGGAGGAGATCCCGTGGAGCAAGCAACGTACGGTAACTATTTAAGCCATGATAAACGCTACGAGTTAACAGATGA

ATTTTTAACGATTTGGCGAGATGTAATGGAAGGAAAAAAAGTGGACTATAAAGGAGAACATCTTGATGTAACAGGTGC

GTACATTCCGTCGCCTCCTGTCCAAAAACCGTATCCTCCGCTGTACTTTGGCGGGTCTTCTCCAGCGGGCAAAGAAGTA

GGCGCAAAGCATGCAGATGTATATTTGTTATGGGGAGAACCTCCTGCAGTGATCAAGACGAAAATTGATGAGATGAAA

GAAAAAGCTGCAAATGAAGGCAGGGACATTCGCTTTGGTATTCGACTGCACGTTATTGTAAGAGAATCGGAAGACGAA

GCGTGGGAATCTGCAGATAAATTAATTAAACATGTTAATGATGATACGATCAAAGCGTTTCAAGATAAGTTTGCGTCAT

TTGACTCCACTGCCCAAAAAACACAGTCAAGTTTGCACAGCGGCACAAAAGATCGAGGTGCGCTGGAGATTGCTCCGA

ATCTATGGGCTGGAATCGGCTTAGCACGAGAAGGAGCTGGAACCGCTCTTGTTGGAAGTCCTGAAATTGTAGCGGATC

GTTTAAAAGAGTACAAAGAGCTAGGAATTGATACGTTTATTTTATCAGGATACCCGCATCTAGAAGAAGCATATACGTT

TGCTGAGCTAGTATTTCCACATATTCAAAAAGAACGAAGCAAGAGATAA

>NM1_A2_03187 assimilatory sulfite reductase (NADPH) hemoprotein subunit

ATGGTAAACAAAATTTTAAAAGCGCCCGAAGGTCCTCCAAGTGACGTTGAGCGCATAAAAGATGAAAGTAATTATTTA

CGCGGTACATTAGGAGAAACAATGCTAGACCGCATCAGCTCAGGTATTTCTGAAGACGATAATCGTTTGATGAAGTTCC

ACGGAAGCTACTTACAAGATGATCGAGATCTTCGTAATGAACGTCAAAAACAAAAGCTAGAGCCAGCTTACCAATTCA

TGCTTCGCGTTCGTACGCCAGGCGGTGTTTCAACACCAGAACAGTGGCTAGTGATGGATGATTTAGCACAAAAATACGG

AAATGGAACGTTAAAGCTGACAACTCGTCAGGCGTTCCAAATGCACGGAATTTTAAAGTGGAACATGAAGAAAACGAT

TCAAGAAATTCATGCTTCTTTATTAGACACAATTGCAGCTTGCGGAGATGTAAACCGTAACGTAATGTGCAATCCGAAT

CCATATCAGTCAGAAGTGCACGCTGAAGTATTCGAATGGTCAAAAAAATTAAGTGATTATTTATTGCCTCGTACAAGAG

CGTACCACGAACTTTGGCTAGATGAAGAGAAAGTAATTAGCACACCGGAAGTGGAAGAAGAAGTGGAACCAATGTAT

GGTCCGCTCTATTTGCCAAGAAAGTTCAAAATTGGCGTAGCTGTGCCACCTTCTAACGATATTGACGTGTATTCACAAG

ACCTTGGCTTTATTGCCATTTTAGAAGACGAAAAACTTGTTGGATTTAACGTAGCAATCGGCGGCGGTATGGGTATGAC

GCACGGGGATAAAGCAACTTACCCTCAGCTTGCAAAAGTGATTGGCTTCTGCCGACCTGACCAAATTCTAGAAGTAGC

GGAAAAAATCATTACGATTCAACGTGATTACGGAAACCGCTCTGTGCGTAAAAACGCGCGTTTTAAATACACGGTTGAT

CGTCTTGGATTAGAAACGGTAAAAGAAGAGCTTGAAAACCGTCTGGGCTGGAGCTTAGACGAAGCAAAATCTTATCAC

TTTGATCATAACGGAGACCGCTACGGCTGGGAAAAAGGTGTGAAAGGAAAATGGCACTTTACTCTTTTTGTTCAAGGTG

GACGTATTGCTGACTTTGAAGACTATAAGCTTATGACGGGTCTTCGTGAAATTGCAAAAGTTCATAGCGGTGATTTCCG

TTTAACGGCCAATCAAAATTTAATTATCGCGAACGTATCCACTCAAAAGAAAAAGCAAATTAGTGACTTGATTGAACA

GTATGGATTAACAGACGGCAAGCATTATTCGGCTCTTCGCCGCAGCTCATTAGCTTGCGTATCGCTTCCGACTTGCGGG

CTTGCGATGGCAGAAGCTGAGCGTTATCTTCCAGTTCTTCTTGAGAAAATTGAAGCAATTGTAGATGAAAACGGTCTTC

GTGATAAAGAAATCACGATTCGTATGACGGGCTGTCCTAACGGCTGTGCACGTCCTGCTTTAGGTGAAATTGCTTTTAT

TGGTAAAGCACCGGGTAAATACAATATGTATCTTGGAGCAGCATTTGATGGCAGCCGCTTAAGCAAAATGTATCGTGA
AAATATCAGTGAAGAAGAAATTTTAAATGAACTGCGCGTCTTACTTCCTCGCTATGCAAAAGAAAGAGAAGAAGGCGA
GCACTTTGGTGACTTTGTCATCCGCGCTGGAGTAATTGAAGCTGTAACAGACGGCACGAATTTCCACGCATAA

>NM1_A2_03188 assimilatory sulfite reductase (NADPH) flavoprotein subunit

GTGTTGCAACTTAAGGTAGTAAACAGCCCTTTTAATCAAGAACAAGCAGATTTGCTTAATCGCCTTCTGCCGACATTAA
CAGAAGCACAAAAAATGTGGTTGAGTGGTTATTTAACAGCATCTCAATCTACGTCTGCCGAAGGAACGCCAGACGTTTC
TACAGCAGCGCCTGCTCAAGCGGAGCAGACGATTTCAAAAGACGTAACGATTCTTTACGGATCACAGACAGGAAATGC
TCAAGGTCTTGCTGAAAATACAGGCAAAACGCTTGAAGCAAAAGGTTTTAATGTAACTGTATCTTCTATGAATGATTTC
AAACCAAATACTTTAAAGAAACTTGAAAATTTATTAATTGTTGTAAGTACACATGGAGAAGGAGAGCCGCCTGATAAC
GCGCTATCTTTCCACGAATTTCTTCACGGCCGTCGAGCGCCAAAGCTTGAAAATTTCCGTTTTTCTGTCTTGTCGCTTGG
AGACAGCTCATACGAATTTTTCTGTCAAACAGGGAAAGAATTTGATGTGCGCTTAGCAGAACTTGGCGGTGAAAGACT
GTATCCGCGCGTTGACTGTGATTTAGATTTTGAAGAGCCCGCAAATAAATGGCTTAAAGGTGTTATTGACGGATTAAGC
GAAGCGAAAGGACACAGCGCTTCGGCAGCTGTTCCGGCGGAAGCTCCTGCAGGAACTTCGCCGTACTCAAGAACAAAT
CCTTTTAAAGCAGAAGTGCTTGAGAACTTAAACTTGAACGGCCGCGGATCAAATAAAGAAACGCGACACTTAGAACTA
TCTCTAGAAGGTTCAGGTTTGACGTATGAACCAGGAGACAGTTTAGGTATTTATCCTGAAAATGATCCTGAGCTTGTTG
ATCTTCTTCTTAACGAATTCAAGTGGGATGCAAGTGAAAGTGTAACGGTTAATAAAGAAGGAGAAACGCGTCCTCTTA
GAGAAGCGCTAACCTCTAATTTTGAAATTACCGTGTTAACAAAACCGCTTTTAAAGCAAGCAGCTGAGCTTACTGGAAA

TGATAAATTAAAAGCGCTTGTAGAAAATCGCGAGGAATTAAAAGCATACACACAAGGCCGTGATGTAATTGACTTAGT
TCGTGACTTCGGCCCATGGAACGTATCAGCACAAGAGTTTGTAGCTATTTTACGCAAAATGCCAGCGCCTTTATTCA
ATTGCAAGCAGCTTGTCAGCAAATCCTGACGAAGTTCATCTAACAATTGGAGCAGTACGTTACGAAGCGCATGGACGC
GAGCGTAAAGGTGTTTGTTCAGTCCTATGTTCAGAACGTTTGCAGCCAGGTGATACGATTCCTGTATACCTTCAAAGCA
ATAAAAACTTTAAGCTTCCTCAAAACCAAGAAACGCCGATTATTATGGTGGGACCTGGTACAGGTGTGGCTCCGTTCCG
CTCATTTATGCAAGAGCGTGAAGAAACAGGAGCAAAAGGTAAGTCATGGATGTTCTTTGGAGATCAGCACTTTGTAAC
AGACTTCCTTTACCAAACAGAGTGGCAAAAGTGGTTAAAAGACGGCGTGCTAACGAAAATGGACGTGGCGTTTTCACG
CGATACTGAAGAAAAAGTATACGTACAAAACCGTATGCTTGAACATAGTAAAGAATTATTCCAGTGGTTAGAAGAAGG
CGCATTTTTTTATGTGTGCGGAGATAAAACAAATATGGCACGCGACGTGCACAACACGCTAGTTGAAATTGTTGAAACA
GAAGGCAAGATGAGCCGCGAAGAGGCGGAAGCTTACCTTGCTGAAATGAAGAAACAAAAACGTTATCAGCGTGATGT
ATACTGA

>NM1_A2_03316 PepSY domain-containing protein

ATGCAGGAAAAGCGCGAAGAATCAGCGAGTACAACGAAAAAAAGCACGGCGCGTTCTCAAGGTCTATACAAAGCCAT
ATGGCGCTGGCATTTTTATGCAGGTTTAATGTTTACTCCGTTTCTTCTTATATTAGCTGTTACTGGCGGGATGTATTTATT
CAAGCCTCAAATTGAAGAAAAACTGTATCACGACTTCTATCATGTACAAGCTCAGTCACAGTCCGTATCCCCTTCAGCA
CAAGTACAGGCTGTAAAGGAAAAGTATCCAGACGCTGATGTGTTGACGTATAAGCCCAGTGATCGCTCTACTCGTTCTT
CAGAAGTAGGAATTTCATTAAAAGACCATACATATACGATGTTTGTTAACCCTCACAATGGTCATATCATAGGAAAAGT

AGATGATTCCAGTCGCTTGATGAATCAAATTGAAGAATTTCACGGAGAGCTGATGGCGGGAACAGCAGGTGATCGTAT

CGTAGAACTTGCGGCCTGCTGGGCAATTGTCTTAATTGTAACCGGCGCTTTTTATGGTGGCCGCGCAAAAAAGACAAA

ATAAAAGGCGTGCTGATTCCTCGGTTTTCAAAAGGTAAAAATGTACTGATACGTGACCTTCATGCAGTACCGGCCTTTT

GGATTTCAGCGGGGATGCTTTTTCTCGTACTAACAGGACTTCCGTGGTCAGGACTGTGGGGCAATGCGTTTCAGCAAGT

CGCCACAAACGCAGGTGTTGGGTACCCTCCGTCTATTTGGGTAGGCAGTGCTCCTACTTCAACCGTGCAAACAAAAGAT

GTTGCGGACGTGCCTTGGGGAGCTGAAACGTTAGAAGTGCCAAGCTCCACAGCTTCGCAATATACAAAAGTATCGCTT

GATGACATTGTAGGTATTGCAAAAGAACAGCATATGCACGACGGCTACACCATTTCTATTCCGCAAGACGCTCAAGGT

GTGTATACGCTCTCTGTTTTTTCACCAAGAGCTCAAGATGAAGCAACCATTCACCTTGATCAATATACCGGAGCTGTGTT

AGCCGATTATCGGTACGACAACTATGGATTTATGGGCAAATTGATTGCGCTTGGTATTACGCTTCATAAAGGCACACAG

TTTGGATTTATCAATCAGCTTATGGGCCTTATTATTTGTATCGGAATTGCTGGTATCGCTATCAGCGGATCCTTACTGTG

GTGGAAGAGAAAACCAGCTAAAAACATGGGGGCTCCTAAAGTTCCTGAAGGAAGTGCAATGCGAATCGTGACGTGTAT

AATCGTCGTGTTTGGTATCCTCTTTCCTTTAGTCGGACTGTCCTTAGTTATCGTGTGGCTTTTAGATTTCTTTGTTATCAA

ACGAATTCCAGCATTGAAGCGATTTTTAAACGCATGA

>NM1_A2_03397 sulfurtransferase TusA family protein

ATGGAATCAAATAAAGTACTAGATGCAAAAGGGCTGGCATGTCCAATGCCGATTGTTAAAACAAAAAAAGCAATGAAT

GAACTAGAACCAGGTCAAGTGTTAGAAATTCACGCAACGGATAAAGGAGCGAAAAATGACCTTACAGCTTGGGCAAA

ATCTGGCGGTCATGAGCTTCTTCAGACAGAAGAAGGCGACGTTCTTAAATTTTGGATTAAAAAAGGATAA

>NM1_A2_03399 sulfurtransferase TusA family protein

ATGATGCAAGCAAACTTCATATTAGATGCAAAAGGGTTAGCATGTCCAATGCCAATCGTAAAAACAAAGAAGAAAATG

AATGAATTAGAAGCAGGTCAAGTGCTAGAAATTCAAGCAACGGATAAAGGCTCTACGGCTGACTTACAAGCGTGGGCA

AAAAGCACAGGTCATGAGTATTTAGGTACCGAGGCAGCAGGTGACGTTCTTCACCACTTTCTTCGTAAAGGCGGAGCTG

AAGAAAACGTAACGCCAATTCCTGAAATTTCATTAGAAGAATTTGCGAAAAAAGTAGAAAATGATAAACATCTTCATA

TTTTAGACGTTCGTGAAGTAGAAGAATACGATAAAGCTCATATTCCAGGCGTAGTTCATATTCCGCTTGGTGAAGTAGA

AAAACGTTTCAATGAGTTAAATAAAGAAGATGAAATTTATATTATTTGCCACTCTGGAAGACGAAGTGAAATGGCAGG

ACAAACAATGAAAAAGCAAGGATTTAAACATTTAATTAACGTGGTTCCTGGTATGCGTGACTGGACGGGTAAAGTAGA

ATAA

>NM1_A2_03400 rhodanese-like domain-containing protein

GTGAAGACAATAGCAGCACAAGAAGTAGAACAATTAGTACAGAACCAAAAACAAATTCACATTTTAGACGTGCGTGA

AGTAGAGGAAGTAAAAACAGGTAAAATACCAAATGCGTTAAATATTCCGCTTCCTTTATTGGAATTCCGTATGCATGAG

TTAGATAAAGCAAAGAATTATATTGTTGTCTGTCGTTCTGGTGGCAGAAGCGGAATGGCAGCTCGTTTTCTTGAGCAGC

AAGGCTACAGTGTAACGAATATGACCGGCGGCATGATGGAGTGGAAAGGGGAAACGGTCTAA

>NM1_A2_03401 rhodanese-like domain-containing protein

ATGATTATTAATGCGCTTATTATCGTCTTTCTGCTGTGGTTTTTGTATCAGCGTTTTGCAACAGTAAAAGGTATTCAGCA

AATAGCCACAACGGAGTTAAAAGCAAAGTTAAAAAATAAAGATAACCAGTTTATCGATGTGCGAACACCACATGAATT

TCGTACCAAACATATTAAAGGGTTTCGAAACATTCCTTTATCAGAATTGCCAGCGCAAACAGGTCAGCTTTCAAAAGAC

AGAGAAGTAGTCGTTATTTGTCAAAGCGGAATGAGAAGTATGAAAGCAAGTAAGCTGTTGAAAAAGCAAGGTTTTATA

GCCATTACCAACGTTAAAGGCGGCATGAATACGTGGAGATAA

>NM1_A2_03403 sulfurtransferase TusA family protein

ATGAAAATTGATCAAGTTTTAGATGCAAAAGGATTAGCGTGTCCAATGCCAATCGTCAAAACAAAAAAGGCAATGGAT

ACTTTAACAACAGGCCAAGTATTAGAAGTGCAAACGACAGATAAAGGTGCTAAAAGTGATCTAACAGCTTGGGCAAAA

TCAACAGGACACGAGCTTATTGATTTTAAAGAAGAAGGAAGCACATTTATCTTTTACATTCAAAAGAGCTAA

>NM1_A2_04509 rhodanese-like domain-containing protein

ATGTCACCAATTACAGCAGTACTCATTTTACTTGGAGTAATCATCATATACTCTGTCGTTATGTTCTTTATCCAACGTCG

CATTATGAAAACGTTAACTGAAGAAGAATTCCGCAGCGGCTACCGCAAGGCACAGCTAGTTGATATTCGCGAACCGAA

TGAATTCGAAAACGGACATATTTTAGGCGCACGAAATATTCCTCTTTCACAGTTCCGTATGCGCATTAAAGAATTTCGT

CCAGATCAGCCTGTGTACATCTATTGTCAAAGCGGAATGCGCACAGGACGTGCAGCACAAATGCTTCGTCGCCACGGT

GTTCAAGAAATCTATGATTTAAAAGGCGGCTTCAAAATGTGGAACGGTAAAGTAAAAAAGAAAAAATAA

>NM1_A2_04622 bifunctional cystathionine gamma-lyase/homocysteine desulfhydrase

ATGAAACGTAAAACACAGTTGATTCATGGTGGAATTGTTGGCGATGAACAGACGGGTGCTGTCTCTGTTCCTATTTATC

AAGTGAGTACTTATAAACAAGAGGGAGCGGGAAAACATACGGGATATGAGTATTCGAGAACCGGTAACCCTACACGTC

ATGCCCTAGAAGAACTTATCAAAGAAATTGAAGGAGGATATGCTGGGTTTGCGTTTGGATCTGGTATGGCCGCTACTAC

TGCCGTGTTCATGCTGTTTAACAGCGGAGATCATGTGTTAATTACAGATGACGTATACGGAGGAACGTATCGAGTGATC

ACAAAGGTACTAAGCCGTATCGGCATTGAGGCTACTTTTATTGATACAAGCGATATCCAAAATATCGAGAAAGAAATT

CGCCCTAACACAAAAGCCATTTTTATTGAAACTCCAACGAATCCTCTTCTAAAAATTACTGACTTGCAGCATGCTTCTTC

AGTAGCTAAACAACACCATTTACTTACCATCGTTGACAACACATTCAGTACGCCGTATTGGCAAAATCCTATCGAAAAA

GGCGCAGATATTGTTCTGCACAGTGCAACTAAGTATTTAGGGGGACACAGTGACGTTGTAGCAGGGCTAGCAGTGGTG

AATTCGCCTAAATTAGCTGAAGACCTTCATTTTATCCAAAATGCGACTGGAGGCATTTTAGGACCTCAGGATTCTTGGC

TTTTAATGAGAGGGATTAAAACATTAGGAATACGAATGGAAGAACACGAATTCAACACGGGGAAAATAGTAGAATTTT

TACTTGCCCATCCTGCTGTGGCAAAAGTATATTATCCAGGATTAAAAACCCACCCAAACCACCTCATTGCTAAAAAGCA

AGCAAGAGGTTTTGGGGGAATGGTTTCGTTTGATGTGGGAAGCGAAGAGAAAGCGGAAGAAGTACTGAGCAAAGTGA

AGTATTTTACATTAGCAGAAAGTTTAGGAGCCATCGAAAGCCTCATTTCCATTCCTTCTAAAATGACTCATGCTTCTATA

CCGAGCGACCGACGTAAAGAGCTAGGAATTACAGATGGATTAATTCGTATTTCTGTAGGTCTTGAAGATGTAGAAGATT

TAATTGAAGACTTAGAAAGAGCATTGGAATAA

>NM1_A2_04744 FMNH2-dependent alkanesulfonate monooxygenase

ATGAAAATGTTATGGTTTATTCCTTCCCATGGAGATGGAAGATATCTAGGGACAAATCGAGGAGGTCGTTCAGCAGACT

ATTCATATTTTCGGCAAGTTGCTCAAGCAGCTGACCGGTTAGGATTTGAAGGAGTTCTGATTCCAACGGGGAAATCATG

CGAAGACCCGTGGCTATTGGCTGCTTCACTTATACCTGAAACAGAGCGTTTGAAATTTTTAGTAGCTGTACGTCCGGGC

ATTATGGCTCCTTCCGTGGCAGCGCATGACTTCGACTCTTGATCGAATTTCAAACGGTCGTTTACTAGTTAACGTAGT

AGCAGGAGGAGATCCAGCAGAATTAGCCGGAGACGGCCTTTTCTTATCACATGATGAACGTTATGAAGCTGCGGATGA

ATTTTTAGATGTGTGGAAAGGGTTATTGGCTGGAGATACGGTTAATTATGAAGGAAAACATATTCATGTAGAGAACAGT

GAGCTGTTGTATCCACCTGTTCAAAAACCTTCTCCACCTATTTATTTTGGCGGATCTTCACCCGCAGGACAAAAAGTTGC

AGCAAAACATTCTGACGTGTATTTAACGTGGGGAGAGCCTCCTGAACAAGTAAGAGAAAAAATAACGGCTGTGAGAAA

GCAAGCAGAAGCAGAAGGCAGAACCGTTAAGTTTGGGATTCGTTTGCATATTATTGTACGGGAAACGGAAGAAGAAGC

TTGGGAAGCAGCCGATCGATTAATTCAGCATTTGGACGACGACACAATTCAAGCTGCACAGCGTACTTTTGCAAGGTAT

GACTCAGTTGGGCAGCAGCGAATGGCGAGCCTTCATAGAGGAACTCGAGAGGAACTTACGATTAGCCCTAACTTATGG

GCAGGTGTGGGGCTCGTCCGGGGAGGTGCAGGAACTGCCCTGGTAGGCAGTGCTGAAAATATAGCGAAACGAATAAA

AGAATATGAAGCTTTAGGAATTGAATCTTTTATTTTATCAGGCTATCCGCATTTAGAAGAAGCTTATCACGTAGGAGAG

TTATTGTTCCCGCTTTTAAATATTGATGAGGGTGAAGGAAAGAGTGAAAGCATCGGTGAGATTATTGCAAACGATAGGT

TTCCAAAAGCTTCAACCTCGTAA

>NM1_A2_04745 NADPH-dependent FMN reductase

ATGAGTCAGTTTACAATTATTACGGGAAGCCCTTCAAAACAATCAAGGTCAGCTGCTTTAAGTGAGTATATTGCAGCAT

ATCTGCAAAATGAAAAACATGAAGTAAAAACCGTGCATGTCCGCGATCTACCAGCTGAAGACTTACTATATGCTAACTT

TTCTAGCCCTGCGATTCAAGAAGCTCAAAAGAAAGTGGCAGATGCTGATGCCGTTATTATTGTCAGCCCAATCTATAAA

GCTAGCTACACAGGAATCTTAAAAACATTTTTAGATTTAATACCTGAAAAAGGTTTGCACAATAAAACCGTGCTGCCGA

TAGCTACAGGGGGAACCATTGCTCATCTACTGGCTCTTGATTATGTATTTAAGCCGACATTATCCGTTTTAGGAGCTACT

GCTCTTATTCATGGAGTTTATATTGTTGATTCTGAGGTGGCCTATACGAAAGAAAATGAAATTGAGTTTATTGCGGAAG
AAGCAGAAGCGAGAATTAAACGTTCATTAAAAGAGCTTGAATATCATCTAGAACAAACGAAAAGGAGTGTTGAATCAT
GA

>NM1_A2_04746 ABC transporter permease

ATGAGTAAAACATCCGTGGTTTCAGGACATGTCCAGTCTTCAGCAGCTCACTTGCAACAAGTGAAAAAAAGAAAGGAA
GAAGCGAAAAATAAGCAGGGAATTTTCCTTCGTTCTCTCGTCGTGCCAATTTTGCTTATCGTCGTGTGGCAAATCATTGG
TTCTGCGGGCTATGTATCTAAAACCGTTCTTCCTACACCTGTTGATATTGTACTTTCTTTCAAACAGCTCATCGTATCGG
GAGAACTCGGAGCTAATCTTCAAATCAGTATTTTACGTGCTGCCATAGGTTTTGCCGTTGGAGCTGGTCTAGGTTTACTA
TTTGGTATTATTGTCGGTTTCTCAAAGAAAACGGAGCACTATATGGATCCATCCATTCAAATGCTTAGAACTGTTCCTCA
CCTTGCCTTGGCTCCTTTATTTATCTTATGGTTTGGCTTTGGGGAAATTTCAAAGATATTGCTAATTGCAAACGGTGCGT
TTTTCCCAATCTATGTTAATACGTATTTAGGCATTCGCGGCGTTGATTCGAAATTATTTGATGTTGCGCGTGTTCTGCAA
TTTAGTAAATGGAAACAAATCACGAAACTCATTTTACCAGCCGCTCTTCCTAATATTTTACTTGGTGTCCGATTGTCTTT
AGGTATTGCATGGCTCGGACTGGTAGTAGCTGAATTAATGGGAGCAAGTGAAGGTGTAGGATATATGATTATGGATGC
TAGGCAGTTTTCTCAAACAGATGTAGTTTTTGTCGGAATTATCATTTTTGCTGTTGTAGGCAAATTAACTGATTCGTTTG
TCCGAGTATTAGAAAAGCGTCTTCTTAAATGGAGAGACAGCTACGCAGGTGAAAAATAA

>NM1_A2_04747 aliphatic sulfonate ABC transporter substrate-binding protein

ATGAAAAAGCTACTTTTAACTGCAGCTATTGTACTTGTTCTCGCATTGGCTGGGTGCGGGACGAAAGAAAAAGCGAGC

GGCGAAGATTCGAAAAACGTTACGATCAATATTGGCATTCAACAAAGCCTAGGACCTTTAATGCTGGCACAAAACCAA

AAATGGTTTGAAAAAGAATTTAAAAAAATAGGTGTAAACGTTAAATGGACAGAGTTTCAAAGCGGTCCTCCGCAATTT

GAAGGGTTAGCATCTGGACACTTGGATTTCGGCCAAGTTGGAAACTCACCGGTTATTTCTGGTCAAGGGGCAGATATTC

CATTTTTAGAAATTGCTAATTCGAGCGACGGATTAAAAGGAAATGCTATTTTAGTAGGAAAAAACAGCAAGATTAAAA

GCGTAAAAGATTTAAAAGGTAAAAAGATTGCGGTTGCCAAAGGCAGCAGCGGTTTTAACTTATTGTACAGAGCTCTTG

ATCAAAATGGGTTAAAGCCAAGTGACGTAAAGATTATTCAGCTTCAGCCAGACGAAGCTCAGCCAGCATTTGAAAATG

GGTCAGTGGATGCATGGTCAATTTGGGAACCGTTTATCTCCCTGCAAAACTTAAAAAATGAAGCTCGTATTTTAGCAGA

CGGCGATTCGTTAAAGGTTGCTTCACCAGGCTTTACTATTGTAAGAGAAGGCTTTGCAAAAGATCATCCAGAGTTAGTC

GTTAAGTTTTTACAAGTTTATCAAAAAGCACTTGAATGGCAAAACGAACATTTTGAAGAATCTGTGGATATTCTAGCAA

AACAAAAAAATCTAGATAAAGATGTAGTAAGACAAGTGTTAAAAAATAATCCTGCCTATAATCGCCCAACCTCTAAAG

AAATTATTGCAGAACAACAGCGAACAGCTGATTTTCAACAATCTTTAGGCGTTATTAAAAAGAAAATTGATACAGGAG

ATGTCGTTGATAATTCATTTATTGAAAAAGCATTAAAAGAAAAATGA

>NM1_A2_04817 bifunctional oligoribonuclease/PAP phosphatase NrnA

ATGAAAGCACAAATATTAGAAGCTATTAAGCAGTTTGACACCATCATTATTCACAGGCACATTCGTCCAGATCCAGATG

CATACGGATCTCAGTGCGGATTAGCGGAATTGTTAAAAACATCTTTTCCGGAGAAGAAGGTATACGTAACGGGAGAAG

AAGCGGAGTCGTTAAAATTTTTATACCGTATGGATGATATCGCCGATGACACCTATGAAGATGCGCTTGTCATCGTTTG

TGATACGGCGAATGAAGCGCGTGTATCAGATCAGCGCTACGCAAGTGGAAAAATGATCATTAAAATCGATCACCATCC
GAATCAAACTCCTTACGGTGATTTACTTTGGGTTGAGACGTCTGCTTCTTCAACAAGTGAAATGATTTATGAATTTTACT
TGGAAGGAAAAGAAAAGGGGTTAGCTCTTAATGATGAAGGGGCAAAGTTAATTTTCACTGGTATTGTCGGTGATACGG
GCCGATTTTTATTTCCAAATACAAAGCCGAAAACGTTTTGCTATGCAAGTGAATTGATTCAATATGGCTTCAATTTTAAG
GATGTATATGATCAGCTATACAAAACAAAAGAAAACGTCGTTCACTTACATGGATATGTGCTGCAAAACTTCACAATGT
CTCCAGCGGGAGTTGCTCACATGAAAATTCCGAATGAAATTCTTCGCAAATATGATGTGACACCTGCTGATGCCTCTCA
GCTAGTCGGCTCGTTAGGACACATTGATGGAGTGAAAACGTGGGTATTTTTTGTAGAAGAAGAAAATCAGATTCGGGT
CCGCCTGCGTTCAAAAGAGCTTGTGATTAATACAATTGCTAGAAAATACAACGGAGGAGGCCATCCTCTTGCGTCCGGC
GCTTCCATTGACTCATGGGAAGAAGTAGAGCCTTTACTACTTGATTTAGAAGAGCTCTGCAGTCAGAAGTAA
>NM1_A2_04876 cysteine synthase A
ATGAAAGTTGTTAACAATATGGCTGATTTGATTGGCGAGACACCTCTTGTTAAATTGAATCGTCTTCAACCAGCAGATG
GAGCATCCGTTTACTTAAAACTAGAATTTTTTAATCCGAGCCGTAGTGTAAAAGACCGTGCTGCTTTTAATATGATCGTG
GAAGCTGAAAAAGCAGGCTTGCTCAATGAAAATTCCACCATTATTGAACCAACGAGCGGAAATACAGGTATTGGCTTA
GCTATGAATGCAGCAGCAAGAGGGTATCGTTCTATTTTAGTCATGCCCGATACGATGACTCAAGAACGAATTAACTTGT
TAAAAGCATATGGTGCTGAAGTTGTTCTAACACCTGGCGATGAAAAAATGCCTGGCGCAATCCGTAAGGCAGAAGAGC
TTACAAAAGAAATTCCAAATGCATTTATGCCCATGCAGTTTGAAAATAATGCAAACCCTGACGCACATCGAAAAACGA
CTGCTAAGGAAATTATTGAGGCAATGAACGATTTAGGAAAAGACCTATCTGCTTTTGTTGCTACTGCTGGAACAGGCGG

AACGATCACGGGCACAGGTGAAGTCCTAAGAGAAAACTACCCAAATATGACCGTTCATGTTGTGGAACCAGCTGGGTC

TCCTGTATTATCTGGAGGAAGACCTGGAAAGCACAAGCTTGTAGGAACAAGCCCTGGATTTATTCCTGATACGCTTAAC

ATAGAGGTTTACGACGAAATTTTAAAAATTAAAGATGAACAAGCCTACGATATTACGCGACGCCTGGCTTCTGAAGAA

GGGATTTTAGTCGGTCCTTCATCTGGCGCTGCGTGCTATGCTGCTATCGAGGTAGCCAAAAAATTATCTCCTGATCAAGT

GGTCGTATGTATTGCTTGCGATACAGGTGAACGTTATTTATCAAGCGACCTATTCTCATATGAATAA

>NM1_A2_04883 rhodanese-like domain-containing protein

ATGGAAGAAGTAAAAGTGATTACGCCTGAAGAGCTTCAAAAACGCGTTGAAAATGGAGAAGAGCTTCATTTAGTTGAT

GTTCGCGAAGATGAGGAAGTAGCAGAAGGAATGATTCCAACTGCTAAACACATTCGTATGAATGATATTCCAACCAAT

GTGGATTATTTCGATAAAGAAAAAGAATATATTTTCATTTGCCGTTCTGGACGCCGCAGTGAAAACGTATGTTACTACC

TTCAAGAACAAGGTTATAAAGTAACAAATATGGTTGGCGGTATGTTAGAGTGGGAAGGAAAAACGGTTAATAAATAA

>NM1_A2_04894 gamma carbonic anhydrase family protein

ATGATTTATCCGTATAAAGAAAAAATGCCTAATATTGCACCCAGCTGCTTCCTCGCAGACTATGTCACGATTACTGGAG

ATGTCACCATTGGTGAAGAAAGCAGCATTTGGTTTAACACCGTTATTAGAGGAGATGTTTCGCCTACTATTATCGGGAA

ACGAGTAAATATACAAGATCAATCAACACTCCATCAAAGCCCAAATGCTCCTTTGCTTATTGAGGATGATGTGACAGTA

GGACATCAAGTTATTTTACACAGCTCCATCATCCGCAAACGCGCGCTCATTGGCATGGGCTCTATTATTTTAGACGGTG

CGGAAATTGGTGAAGGTGCTTTTATCGGTGCAGGAAGTTTAGTGCCTCCCGGTAAAAAAATACCACCTAATACTCTCGC

ACTCGGTCGACCTGCTAAAGTAATCCGCACTCTGACTGAGGAGGATTTAAAAGATATGCAGCGCATTCGTACAGAATAT

GTAGAAAAAGGGCAGTATTATAAGTCTATTAAGAAATCTGATTCTTCATTATAA

>NM1_A2_04905 ABC transporter permease

TTGAAACCCACTAGCTCCCTAGATACTCTTCACGAAAGCTACCTTCGCAAGGTTCGGGGCGAAAAACGATTAGTACTGA

GCGTGCAGATGGTCATTTTTTTAGCCTTTTTTAGCTTCTGGGAACTAGCTTCTCGTTTAAAGTGGATTGATCCTTTAATTT

TCAGTTCCCCTACTAAAGTGTGGCATTTATTTTTAATTAAACTTGCCGACGGCTCGCTAGCAGAACATATTGGTTTTACA

TTATTTGAAACGGTTCTTGGCTTCATTTTAGGCACATTGCTAGGTATTTTACTTGCAACGGCCCTTTGGTATTCAACTAG

GCTTGCAAACATATTAGACCCTTACCTCGTTATTTTAAACGCCATGCCTAAAGTAGCGCTGGGCCCTATTTTAATAGTAG

CGATCGGTCCAGGATTTTTCTCAATTTTAACGATGGGAGCGATTATATCCGTTATTATTACATCCATCGTCGTTTACACA

GCCTTTAAAGAAGTGGATCCAAACTATATTAAATTACTAAAAAGCTTCGGGGCAACGAAAACCCGCTGTTTTAAAGAA

GCAATTCTTCCAGCTTCCATGCCTGCCATTATTTCCACATTTAAAGTAAACGTCGGTTTATCTTGGGTAGGCGTCATCGT

AGGAGAATTTTTAGTTTCTTCAAAAGGGCTTGGATATATGATTATTTATGGTTTCCAAGTCTTCAACTTTACTCTTGTTCT

TCTCAGCTTATTATTAATTGCCATTTTCGCTACGATTATGTACCAAGGCGTGGCTTATTTAGAAAAGAAATTGATCCGAC

GCTCATAA

>NM1_A2_04966 adenylyl-sulfate kinase

GTGAGTAAATCAACAAACATTACGTGGCATGATGCTGGAATTACAAAACAGGAAAGACGCGAGCAAAATAATCATCAC

AGTTTTGTATTATGGTTTACGGGCCTTTCGGGTTCAGGTAAATCTACCGTAGCAAACGCAGTGGCAAAAGCGCTTTTTG

ATAAAAATATTCGCAATTATGTATTAGATGGTGACAATGTTCGTTTTGGCTTAAACAAGAATTTAGGGTTTTCTGCTGAA

GATCGTACAGAAAATATTCGCCGTATCGGTGAGGTTTCTAAGCTGTTCGTAGACAGCGGTCAAGTGGTACTAACAGCAT

TTATCTCTCCATTCCAAGAAGATCGCGCTCAAGTGCGTGAGATCTTAGAGGGGAATGAATTTTTGGAAGTATACGTAGA

GTGTCCGTTAGAAGAATGTGAAAAAAGAGATCCAAAAGGCCTTTACAAAAAAGCAAGAAGCGGAGAAATCCGTGATT

TTACAGGAATTGATTCTCCCTATGAGTCACCTGCTAATCCTGAAGTAACAATTAATACAAGCACACAGTCTGTAGAAGA

ATGTGTACAAACTGTTATTGAATATTTATCTAACCGAAAATTCATCTAA

>NM1_A2_04967 sulfate adenylyltransferase

ATGACAACAATTCAAGCACACGGTGGAGAATTAATTAATTTATATCAACCAACATATGACTACACTTCACTAACAAAA

GAAATTGAAGTAGACAACATGGCTCTAAGTGATTTAGAACTAATTGGGATTGGTGCTTACAGCCCAATTACGGGGTTTT

TAGGAGAAAAAGATTATCAATCAGTTGTTGAAAATATGCGTCTAGCAGATGGAACTGTATGGAGTATTCCTATTACTCT

TCCCGTAACAGAAGAGCAGGCTAAAGAATTAAACATTGGCGACAAAGTAAAACTAGTTCAAAGCGGTGTGACATATGG

TGTTTTAGAAGTTTCTGAAGTATATACGCCAAATAAAGAAAAAGAAGCGGAAAATGTGTACCGCACAGCAGAACTTGC

CCACCCTGGTGTGAAGAAAATGATGGAACGCCCAAATGTATATGTAGCAGGTCCAATCGTACTTGTAGAACGTACACC

TAAAACGCGTTTTGAAAAGTATTACCTAGATCCAACTGAAACAAGAGCAGCATTTGAAGAGCGCGGTTGGAAAACGGT

TGTTGGATTCCAAACTCGTAACCCTGTTCACCGTGCGCATGAGTATATTCAAAAAACAGCATTAGAAATTGTAGACGGC

CTGTTTTTAAATCCACTTGTAGGAGAAACAAAGTCAGATGATATCCCAGCAGATATCCGTATGGAAAGTTATGAAGTAC

TTTTAGAGAACTATTATCCTAGCGATCGTGTAGCTTTGGCAGTTTTCCCTGCTGCAATGCGCTATGCTGGACCTAGAGAA

GCTATTTTCCATGCAATGGTTCGAAAAAACTTTGGATGCACGCATTTCATTGTAGGCCGTGACCACGCTGGTGTTGGTG

ACTACTACGGAACATATGATGCACAAAAAATCTTCAGTAACTTTACGGCAGATGAATTAGGAATTACACCTTTATTTTT

TGAACACAGCTTCTACTGCAAGAAGTGTGAAGCGATGGCATCAACGAAAACATGCCCTCACAGCAAGGAAGATCATAT

GATTCTATCTGGTACCAAGGTACGTGAAATGCTTCGTAACGGAGAAGTGCCACCAAGCACATTCAGCCGTAAAGAAGT

AATTGAAGTGTTAATTAAGGGATTAGCAAAACAAAAAGTATCTTCAAAATAA

>NM1_A2_04969 phosphoadenylyl-sulfate reductase

ATGGCAGACTTATATACGTACGAAAACTGGAGCGATACATTTCCAGAGTTCGACAGTAAAGATGAAACAAAAGGAGCC

CTTTCAGCATTGCAGTGGGCGTATGATACCTACGGAGATTCAATTATTTATGCGTCAAGCTTTGGGATCGAAGGAATCG

TGTTAATTGATTTAATTTCAAAAGTGAAAAAAGACGCTGAAATTGTCTTTTTAGATACAGGCGTTCATTTTAAAGAAAC

ATATGAAGTGATTGATGCAATTAAAGAACGCTTCCCTGATTTACGTATTCATATGAAAAAGCCGGATTTAACTCTTGCA

GAACAAGCTGAAAAACATGGAGATGAGCTTTGGAAGAGTCAGCCTAACTTATGCTGTCAAATTCGAAAAATCATTCCG

CTAAGAGAATCCCTTGCTCCTTATGATGCGTGGATTTCTGGTTTGAGACGCGAGCAGTCTGAGTCTCGTGCCAACACGA

ATTATTTTAATAAAGATGAGAAATTCAAGAAGGTGAAAGTTTGTCCGTTAATTCATTGGTCATGGAAAGAAATTTGGCG

CTATGTGTATAAGCATGATTTGCCGTACAACAAACTTCATGATCAAGGCTACCCAAGTATCGGCTGTGAGCCCTGTACC

GCACCTGCCTATAATGTAGACGATTTACGTTCAGGAAGATGGGCAGGTACACAAAAAGTTGAGTGCGGATTACACGAA

TCTTAA

>NM1_A2_05232 (Fe-S)-binding protein

ATGACAGGGTTGCTGTGGGTGAATTTTCTCTTTTTATTATTGTAACCGCTTACGCTATCTCTTTGTTTGTATATTTAATT

CGCACACGCATTGAGTACATTCAATTAGGAAAAAAAGTTGAATTTGATCAGCGGTTAAAAGAAAGGCTGCAAAAAATT

TGGGTGAATGTGTTTGGACAAAAGAAACTGTTAAAAGATAAAAAAAGTGGAATTATGCACGTCATGTTTTTTTATGGGT

TTATTTTAGTTCAGTTCGGCGCGTTAGACTTCATTATAAAAGGTTTGCTTCCAGGTAAACATTTGCCGCTTGGACCTTTA

TATCCCGGCTTTACATTTTTCCAAGAAATTGTGACGTTTTTGATTTTAGTAGCAGTTGTCTGGGCTTTTCACCGTCGCTAT

GTAGAAAAGCTTGTGCGTTTAAAAAGAGGATTTAAATCTGGTTTAGTACTGATTTTTATTGGAGCACTTATGCTGTCAGT

GCTTGTTGGAAACGGCATGTCAATCATCTGGCATAATCATGAACCAGCATGGACAGAACCGATTGCGTCATCTATTGCC

ATCTTGTTTAAGGGAATCAATGATACTGCCGCTATCTCCATTTTTTATGTTTCATGGTGGATTCACTTAGCCGTGTTATTG

ACGTTTTTAGTATATGTTCCTCAATCAAAACATGCTCATTTACTGGCTGGACCGGCTAACGTATTTTTTAACCGTGTTTCT

AAGCCAGGTAAGCTTGAAAAGATTGATTTTGAAGATGAGACGCAGGAGACGTTTGGGGTTGGTAAGATTGAAGAATTC

ACGCAGCATCAGCTCATTGATTTATATGCCTGTGTAGAATGCGGTCGATGTACGAATATGTGTCCGGCAACTGGTACGG

GGAAAATTTTGTCTCCAATGGATTTAATTTTGAAGATGCGCGATCACTTAACAGAAAAAGGAGCGGTTGTGACATCTAA

GGCTCCTTGGGTTCCTACGTATGCATTCTCTAAAACAAAAGGAAATCAGCTGGCGATGATGGCGGCCAGCCAAGGCGG

AGCAGAAGCCGCAGCAACAGCGGAATATAATCCCGCCCTAATTGGGGATGTCATTACGGAAGAAGAAATTTGGGCATG

TACAACGTGCCGAAACTGTGAAGATCAATGTCCCGTAATGAACGAGCATGTAGATAAGATCATAGATTTACGACGTTTT

TTAGTATTAACCGAAGGAAAGATGGACGCGGATGCTCAGCGTGCCATGACGAATATTGAGCGTCAAGGAAACCCTTGG

GGATTAAACCGAAAAGAGCGTGAAGAATGGCGACATGTAAGAGAAGATGTTCACATTCCGACTGTTAAAGAAGTAAGT

AAAGCGGGTGAAACGTTTGAGTATTTGTTCTGGGTAGGTTCGATGGGATCTTATGATAACCGCAGTCAAAAAATCGCTC

TATCGTTTGCTAAGCTTCTCAATGAAGCGGGTGTTTCATTTGCGATTTTAGGAAATAAAGAAAAAAATTCTGGTGATAC

GCCCCGCAGGTTGGGCAATGAATTTTTATTCCAAGAGTTAGCTTCTAAAAATATCGCTGAATTTGAAAAAAATGAAATC

AAAAAAATTGTTACGATTGATCCACATGCTTACAATATCTTCAAAAATGAATATCCAGATTTCGGTTTTGAAGGAGAAG

TGTATCATCACACAGAATTATTAGCTATGTTGGTGAAAGAAGGTAAGCTGACACCAAAACATGCGGTTAATGAAAAAA

TTACCTTCCATGATTCCTGTTATTTAGGAAGATATAACGAAGTATACAGTCCACCAAGAGAAATTTTGAAAGCGATTCC

AGGTGTTAGTTTGATTGAAATGGATCGCAACCGTGAAAATGGTATGTGCTGTGGAGCAGGAGGAGGGTTAATGTGGAC

GGAAGAAACGACCGGCCAGCGCATTAACGTAGCGCGCACGGAACAAGCCTTAGCCGTTAATCCAAGCATTATCAGTTC

AGGTTGTCCGTACTGCTTAACGATGCTAAGTGACGGAACAAAAGCAAAAGAAGTAGAAGAAAAGGTTCATACGTATGA

CGTAGCAGAACTGCTTGAAAAGTCAATTTATGGTGACGTGCAAGAACAGGTTTCATAA

>NM1_A2_05315 DHH family phosphoesterase

ATGCCTGTCTTTACAAAAAAGAACGTCATTCGCTATCCATTTTACTCATTGTATAGTATAGCCCTGATTTTAGTAGGTAT

CGTGACCTATCATAATTGGATCATAGGAATGATAGGCTTTATATTACTATTAGCCTGTCTTTTTCTATACATGAGAATGG

AGCGAATGCTGTCCGATGAGTTTGAAACCTATATTTCAATGCTGTCTCATCGGTTGAAAAAAGTAGGCGAAGAGGCATT

AATGGAAATGCCGATTGGGATTATGCTATTTAATGATGAATATCAAATTGAATGGACCAATCCTTTTCTAGCATCTTGTC

TGGGTGAAGACACACTGGTGGGAAGATCATTATACGATGTGGCAGAGAGTATTATTCCACTGATCAAACAAGAAGTGG

AAACAGAAGTTGTCACGTTGCATGATCGGAAATTCAAAGTGGTGATTAAACGAGACGAGCGCTTACTGTACTTCTTTGA

TATTACAGAGCAAATAGAAATCGAAAAATTGTATGAGGAAGAACGAACAAGTTTAGGCATTATCTTTTTAGATAATTAT

GATGAACTAACGCAAGGAATGGATGATCAAGTTAAAAGTAACTTGAATAGCCAAGTAACTTCTATGTTAAATAGTTGG

GCTCAAGAATACGGTATTTTTATTAAACGTACGTCTTCTGAGAAATTTATTGCGATTATGAATGAACAAATCTTAATTCA

TTTAGAGCGAAGCAAATTTTCTATCTTAGACCAAGTTCGAGAAGAGACGTCTAAACAAAATATACCGCTTACATTGAGC

ATTGGTATCGGAGCTGGTGCTGCTGATTTACCTGAGCTGGGTGCTTTAGCTCAATCTAGCTTGGATTTGGCCTTAGGCCG

AGGTGGAGATCAAGTAGCGATTAAACAGCCGAATGGAAAAGTGAAATTCTTTGGCGGCAAGACTAATCCAATGGAAA

AACGGACGCGCGTGCGTGCCAGAGTTATTTCTCACGCGTTAAAAGAGCTCATTACAGAGAGTGGAAAAGTGATTATCA

TGGGTCATAAGTATCCGGATATGGATGCTGTAGGAGCTGCTATTGGGATATTAAAAGTAGCGCAAGTAAATCAGAAAG

ACGGTTTTATCGTATTAGATCCTGATCATATTGATACAGGTGTTCAGCGAATGCTAGAAGAGATTAAAAAGAAAGAAG

GCCTGTGGGAACGATTTATTACGCCTGAAGAAGCCTTAAATCTTGTGAGTGATGATACTCTTTTAGTAGTAGTAGATAC

GCATAAGCCGTCGCTCGTTATTGAAGAAAGGTTATTAAATCGTATTGAAAATGTCGTGGTCATTGATCATCATAGACGA

GGAGAAGACTTTATCGAAGACCCTCTTCTTGTCTATATGGAGCCATATGCTTCCTCGACAGCAGAACTCGTTACTGAGC

TTCTTGAATACCAGCCAAAACGATTTAAAATTGATATGCTAGAAGCAACAGCACTTTTAGCCGGTATTATTGTCGATAC

AAAAAGCTTTACATTACGCACGGGATCTCGTACGTTTGATGCAGCGTCCTTTTTACGTTCTCAAGGCGCAGATACGGTT

CTTGTTCAAAAGTTTTTAAAAGAAGATATTACGCAATATGTACAGCGAGCACGCTTTATTGAACATGCAGAGATTTATA

CAGCAGGTATTGCCATTTCACGCGCTGAACCGAACAAAATGTATGATCAAGTGTTAATCGCGCAAGCTGCAGATACGCT

GCTTTCAATCAGCGGAGTGGTTGCTTCGTTTGTTATTTCTAAGCGACGGGATAATCTAATTGGCATTAGTGCTAGATCGC

TTGGAGATATCAATGTACAAGTTATTATGGAAAGTCTCCAAGGCGGAGGCCACTTAACGAATGCTGCTACTCAGCTTCA
AGACATTAGTTTGGATGAAGCTGAAGAAAGGCTTAAACAAGCAATTGATGAGTACTTAGATGGAGGTAAAAAATCATG
A

NR1 Sulfur genes

NR1_01119	sat	69.9	309	91	2	42	348	492	800	3.7e-123	442.2
NR1_01903	ssuD	100.0	351	0	0	1	351	1	351	7.6e-201	700.3
NR1_04343	doxD	61.4	153	59	0	1	153	1	153	8.1e-52	204.1
NR1_04665	metB	61.9	386	141	1	7	386	10	395	1.1e-139	497.3
NR1_00078	cysK	100.0	307	0	0	1	307	1	307	1.3e-169	596.3
NR1_04366	mccB	100.0	377	0	0	1	377	1	377	7.1e-213	740.3
NR1_01448	sir	100.0	540	0	0	1	540	1	540	0.0e+00	1082.8
NR1_00114	cysE	100.0	221	0	0	1	221	1	221	4.4e-122	438.0
NR1_04757	cysJ	99.8	433	1	0	1	433	1	433	1.5e-246	852.4
NR1_05337	hdrD	79.5	702	139	2	1	702	1	697	0.0e+00	1125.5
NR1_04617	nrnA	99.7	310	1	0	1	310	47	356	1.1e-179	629.8
NR1_01603	sseA	100.0	277	0	0	1	277	1	277	5.7e-167	587.4
NR1_00820	tusA	100.0	186	0	0	1	186	6	191	6.6e-103	374.0
NR1_04251	metB	100.0	370	0	0	1	370	1	370	3.2e-210	731.5
NR1_02813	ssuC	100.0	282	0	0	1	282	1	282	1.1e-149	530.0

NR1_04367	cysK	60.9	261	101	1	44	303	31	291	2.3e-84	313.2
NR1_00200	doxD	99.4	168	1	0	1	168	1	168	9.9e-90	330.1
NR1_04736	glpE	100.0	101	0	0	1	101	22	122	2.2e-52	205.3
NR1_00775	ssuC	100.0	250	0	0	1	250	1	250	1.8e-132	472.6
NR1_01446	sat	100.0	378	0	0	1	378	1	378	1.0e-219	763.1
NR1_04202	glpE	70.2	114	34	0	14	127	6	119	1.3e-41	169.9
NR1_02814	ssuA	100.0	328	0	0	1	328	1	328	1.4e-180	632.9
NR1_04785	ssuC	100.0	268	0	0	1	268	1	268	1.6e-142	506.1
NR1_04252	mccB	71.1	381	110	0	3	383	5	385	1.7e-153	543.1
NR1_00823	pspE	100.0	119	0	0	1	119	1	119	1.2e-60	233.0
NR1_01799	cysK	100.0	305	0	0	1	305	1	305	6.4e-172	604.0
NR1_02812	ssuD	100.0	370	0	0	1	370	1	370	4.2e-210	731.1
NR1_03411	fccB	99.5	399	2	0	1	399	1	399	9.4e-232	803.1
NR1_01445	cysH	100.0	234	0	0	1	234	1	234	9.4e-139	493.4
NR1_04761	cysE	68.5	168	53	0	1	168	1	168	4.0e-46	185.3
NR1_00822	tusA	100.0	76	0	0	1	76	5	80	3.0e-38	157.9
NR1_00824	glpE	100.0	98	0	0	1	98	1	98	6.4e-49	193.7

NR1_01447	cysC	100.0	197	0	0	1	197	1	197	8.5e-109	393.7
NR1_00449	ssuC	100.0	257	0	0	1	257	9	265	5.0e-133	474.6

NR1 Nucleotide sequencing

>NR1_00078 cysteine synthase A

ATGCGAGTGGCACAATCAGTTTCAGAATTAATCGGGAAAACGCCGATCGTTAAGTTGAACCGCATCGTAGAATCAGAC

AGCGCAGATATATACTTAAAATTAGAATTTATGAATCCGGGGAGTAGTGTTAAAGATCGTATCGCATTAGCTATGATTG

AAGATGCTGAAAAAAAGGGATTATTAAAAGAGGGCGATACAATCATTGAGCCGACAAGTGGTAACACAGGAATTGGT

TTAGCGATGGTAGCGGCTGCTAAAGGATATAATGCAATCTTAGTAATGCCAGAAACAATGAGTATTGAGCGTCGTAATT

TATTACGTGCTTACGGTGCGGAATTAGTATTGACTCCAGGGCCTGAAGGAATGGGTGGAGCAATTCGTAAAGCGACTG

AATTAGCAAAAGAACATGGCTACTTTATACCACAACAGTTCAAAAACCAATCGAATCCAGAAATTCACCGTTTAACAA

CAGGTCCAGAAATTATTGAACAAATGGGCGACCAATTAGATGCGTTTATTGCAGGTATTGGTACAGGTGGAACGATTA

CTGGTGCTGGTGAAGTACTGAAGAAAGCGTATAAAGATATTAAAATTTATGCAGTAGAACCTGCAGATTCACCAGTATT

ATCTGGTGGGAAGCCAGGTCCACATAAAATCCAAGGAATTGGGGCGGGATTTGTTCCGGAGACGTTGGATGTAGAAGT

ATATGATGAAATTATTCAAGTGAAAACAGAGCAAGCATTTGAATATGCGAGAAGAGTGGCTAAAGAGGAAGGTATTTT

AGTTGGTATCTCTTCAGGCGCAGTTATTTATGCAGCAACAGAGGTTGCGAAAAAGTTAGGTAAAGGAAAAAGGTACT

TGTTATTATCCCGAGTAACGGTGAACGTTATTTAAGTACACCACTTTATCAATTTGAATCCTAA

>NR1_00114 serine O-acetyltransferase

ATGTTTAAGAGGCTTCGGGAAGATATTGAAGTCGTTTTTGAACAGGATCCAGCGGCAAGAGGTTATTTCGAAGTCATTT

TAACTTACTCTGGATTACATGCAGTTTGGGCCCATCGAATTGCACATGCTTTTTATAAAAAAAATTTTTTCTTTCTTGCA

CGTTTTGTTTCACAAGTGAGCCGCTTTTTTACTGGCATTGAGATCCATCCAGGAGCAACTATTGGTCGACGTTTTTTCAT

AGATCATGGAATGGGAGTTGTAATCGGAGAAACTTGTGAAATTGGTGATAATGTAACGATTTATCAAGGGGTTACATT

AGGTGGTACAGGAAAAGAAAAAGGAAAGAGGCATCCAACAATTCAGGATAATGTATTAATTGCGACGGGTGCTAAAG

TTCTTGGTTCTATTACTGTGGGAGAGAATTCTAAAATCGGAGCAGGGTCTGTCGTATTAAAAGAAGTTCCTGCACATTC

TACAGTTGTAGGTATACCTGGCCGTGTCGTTATTCAAAATGGAGTAAAGATTGGTCAAGAATTAAATCACTCTGACCTT

CCGGATCCGATTTTTGATAAATTAAAGGCTATGGAAGTAGAACTTGATAAATTGAAGAAACAATTGGAATTAAAGGTA

GAAAGGAAGGATAAAAATGACTATTCACATTTATAA

>NR1_00200 DoxX family protein

ATGGTTATCAATTTTTTAAGAACTGATAAACGTGCTACTTTCATATTATTACTTTTACGGCTTTACATAGGGTATACATG

GCTCGCTGCTGGGATAGGCAAAGTCTTCGGACAATCTTTTGACGCAAGTGGCTTTCTAAAGGGTGCTATTGCTCAAGCA

TCAGGTGACCACCCTGCAGTACAAAGTTGGTGGGCAGATTTTCTTCAACATTTTGTCCTTCCAAACGCAGACCTATTTAG

CTTTTTAGTTCAATGGGGAGAAATTTTAGTAGGTCTAGGTTTAATTTTAGGCGGATTAACAAAAACAGCTGCATTTTTCG

GAATCATAATGAATCTTTCATTTTTATTAAGTGGAACCGTTAGTGTAAACCCAAACCTGCTTATTTTGACTATGTTCATT

TTAGTTGCAGGGCAGAACGCTGGACGTATTGGATTAGATGGCTATGTTTTCCCTAAACTTTTCCGTAAAAACAACCACG

GAACATATAAATTAAGTAAAACTGCATAG

>NR1_00449 ABC transporter permease

ATGAATATGCAAAACCAGAAGCAATCTAAACTTATTACAACTATTTGGCTTATCCTTCTCATTGCGATATGGGAAGGAT
CTGTTTCATTATTTAAAATCGAACCGTGGATTTTACCAAAGCCTTCTGCCATTGTTCAAGAATTAATTGGGATGAAAGAT
TTACTATTACCAAATACGATGCAAACACTCCAGGAAGTTATAATTGGACTATTCTTTGCAATTTTATTGGGGACGAGCA
TCGCAATTATTATGGACGTTATACCGTTATTTCGTATTTTAATAAATCCATTGCTCGTTATTTCGCAAACAATTCCAATTG
TTGTACTTGCACCGCTATTTATTATTTGGTTTGGATATGGGATGTTGCCGAAAGTAATGGTCGTAATACTCGTTTGTTTCT
TCCCAATTGCGCTTAGTATTTTAGAAGGTTTTCAAACAGTAGATAAAAACATGCTGAAACTGTTACAAACAATGAAGGC
GACAAAATGGCAAGTTTACCAGAAAGTAAAATTTCCAGCAGTGCTTCCATACTTTTTCTCAGGTTTAAAAATTGCAGTT
ACATATAGCGTAATGGGGGCAATTATCGGAGAATGGCTCGGTGCAAGTGAAGGATTAGGCGTTATGCTTACGAGGGCT
ACAAAATCCTTTTTGACTGCCCGAGTATTTGGTGTTGCAGCAATCATCGTTATGGTGACATTATGCCTGTATTTTATCGT
AGAGTTTATGGCAAGAATAACAGCACCATGGATATATAGAAAGGACGGCAGAAAATGA

>NR1_00775 ABC transporter permease

ATGAACCGATTGAAAGAATTAGTTCCTGCTATTACACTTTCTAGTATTTTACTAGCTGTGTGGGAAATAGGAGCAAGAA
TTGTAGATGAGATGTACATTTTACCGTCACCTTCTGCGATTTTAATGAAGATATGGAAACTAAAAGACATATTATTTAC
AGTTCATTTACCTGCAACGTTATACGTCGTTTTAATAGGTGTTGTTATCTCTATCGTATTAGGTGTAGGGCTAGCGATGT
TAATGAATGCGAGTACATGGATGGAGAGAGCATTTTATCCATTATTAGTTGCTTCACAAACGATTCCGATTACTGCGCT
TGCTCCGCTATTTGTTTTATGGTTTGGATATACAATTTGGAGTAAGGTTGTTGTCACAGTTTTAATTACGTTTTTCCCAAT
TGCGGTCAATACGTATGATGGACTGCGTAGTACGAAAAAAGAATGGGAGGAGCTCTTAGTTACATATGGAGCAACAAA

AAGAGATATTTTTCTTAAATTAAAGTTGCCGTCTGCTCTTCCTTATTTTTCTCAGCTTTAAAAATTGCAGTTCCGCTTAG

TGTTATCGGAGCAGCAATTGGAGAATGGCTCGGTGCACAAGCTGGGCTTGGATATTTCAGTAAGAGAATGATGACGCA

GTTAGATGGAGCTGGCGTATTTGCACCTATTGTATTGTTATCATTATTAGCTATTTTCTTCGTCGTAATTATTTCCATATT

AGAAAAGAAATTCATTAGTTGGAGGAAGCATTCATGA

>NR1_00820 sulfurtransferase TusA family protein

ATGAGTATAAAAGTGGATATGAGTTTAGATTGTAAAGGTTTGGCTTGTCCGATGCCTATTGTGAAAACGAAGAAGGCG

ATAGAAGGATTGGCACCAGGGCAAGTGATTGAAGTTAAGGCAACAGATAAAGGATCTACGGTAGATATAAAAAGTTG

GGCGAGTAAAGTAGGACATCAATATATCGGGACGAAACACGAGGGTGATGTATTGGTGCATTATGTTAGGAAAGCAAA

CGAGCATGAAGTGAATGAAGTTGTGAAATATCCACATACAATTACGAATGCAGAATTACAGTCTATATTGTCTCATGGA

GAAGAGAGCATTGTATTAGATGTTCGCGAAGCAGCGGAATTTGCTTTTGGCCATATTCCATCCGCCATTTCGATACCAT

TAGGTGAATTAGACAGCGCAGTGTTAGATGAGACGAAGCAAATTTATGTTATTTGTCGAACTGGTAACCGCAGTGATGT

GGCTTGCCAAATGTTGAAAGAGAAAGGTTTTTCAAATGTGAAAAATGTCATTCCTGGTATGTTAGGGTGGCAAGGAAA

TGTGGAGAAATAA

>NR1_00822 sulfurtransferase TusA family protein

ATGATGAATATAAAACAAGTATTAGATGCGAAAGGTTTAGCATGTCCAATGCCGATTGTAAGAACAAAGAGAGCAATG

GATACTTTGCAAACTGGAGAAGTGTTAGAAATACATGTAACGGATAAAGGGTCAGTTAAGGATATTCCAGCGTGGGCA

AATAAATGTGGCCATGACATCGTAAAGCTTGAAGAAGAAGGCGATGTGCTGAAGTTTTGGATTAGGAAGGCGTAG

>NR1_00823 rhodanese-like domain-containing protein

ATGAACACAATATTAAGTACGCTTTTCATTGTACTTGCTGCATGGTTTGTTATTTCACGATTTTTACCAGTGAAAGGTGT

TCAAAATATAAATGGCAAAGAATTGAAAAGTATAGTGGGGAAAAAGGGGAAGTATTTTATTGATGTCCGTACAGTAGG

TGAATATAGAGGGAATCATATGAAAGGCTTTCAAAATATCCCCCTAAATGATCTAGCAAGTAAGGCGAATCAATTAGA

TAAGAATAAGGAAGTAATCGTTATTTGCCAAAGTGGAATGAGAAGTAAGCAAGCAGCAAAGGTATTGAAAAAACTAG

GGTTTCAGCAAGTAATAAACGTTTCAGGCGGGATGAATAGTTTGTGA

>NR1_00824 rhodanese-like domain-containing protein

ATGAAAGAAATGACTACAAAAGAATTAGAAGAGAAATTGTTGCGTAAAGAAGTGGTAAATATTGTGGATGTACGTGAA

GTAGAAGAAGTAGCTGAAGGGAAAATTCCAGAAGCATGTAATATTCCACTAGGACTATTAGAGTTTCGTATGCATGAA

TTAAATAAAAATCAGGAATATATTATCGTTTGTCGTTCAGGCGGGAGAAGTGCAAGAGCTGTTCAATTTTTAGAAAGTT

ACGGTTTTCAAGTAATCAATGTGGTAGGTGGTATGTTAGCTTGGGAAGGAAAAGTAGAATAG

>NR1_01119 sulfate ABC transporter substrate-binding protein

ATGAAAAAGTGGGGTAAGACAGCGTTTATGAAAAGTACAGGTGTTTTATTATCAGCAGTTGTACTTTTATCAGGGTGTG

GTTCGGCAACATCCACTAGTAATTCTGAAGGAAGTGGAGATAAAAAAACAGTTGAGCTTTTAAATGTTTCTTATGATCC

AACGAGGGAGTTATATCAAGATTTCAATAAAGATTTTGCGAAGTATTGGAAAGAGAAGCATGGGCAAACAGTAAATGT

AAAACAATCACACGGGGGATCTGGAAGTCAAGCGCGCTCTGTTATTGATGGCTTAGAAGCAGACGTTGTTACACTAGC

ACTTGCTTATGATATTGATGCGATTAGTAAGAAAAAACAATTAGCAGAAGATTGGCAGAAGCGACTTGCGAATAATTC

CACTCCATACACGTCAACGATTGTATTTCTTGTTCGAAAAGGAAACCCGAAAGGTATTAAAGACTGGGATGATTTAACG

AAAAAAGGTGTGTCTGTCATTACACCAAACCCAAAAACATCAGGCGGGGCACGTTGGAACTATTTAGCGGCGTGGGGA

TACGCACTGAAAAAATATAATAATAGTGAAGATAAGGCGAAAGAGTTTGTTAGCCAAATTTATAAAAACGTAGAAGTA

TTAGATTCAGGAGCGCGCGGAGCAACGACAACATTTGTTGAAAAAGGAATTGGTGACGTATTAATCGCATGGGAAAAT

GAAGCGCTATTATCCCAAAAAGAGCTTGGAAAAGATAAATTTGAAATTGTAACTCCTTCTATTAGTGTACTTGCAGAAC

CACCAGTAGCGGTTGTAGATAAAGTCGTTGATAAAAAAGGAACGAAAAAAGTAGCAGAAGGGTATCTTGAATATTTGT

ACTCAGAAAAAGGACAGGAAATTGCGGCAAAAAACTTTTATCGTCCGCGTAATGAAAAAGTAGCAGAAAAATATACAT

CCCAATTTCCGAAAGTTCAATTATTTACGGTAGATGAACTATTTGGTGGCTGGAAAAAAGCACAAGAGAAACACTTTAA

TGACGGCGGCGTGTTCGATAAAATTTATCAAAAATAA

>NR1_01445 phosphoadenylyl-sulfate reductase

ATGTTGACGTATGAAACGTGGGAAGAAAATGTTGTTTCGTTTTCGGAAGAAGATGAAACAAAAGGTGCGTTATCAGTA

TTAAATTGGGCTTATAAAGAATATAAAGATGAGGTTGTCTACGCATGTAGCTTTGGGGTGGAAGGTATGGTATTGCTGC

ACATTATAAACCAAGTAAATCCATCTGCTAAAGTTGTATTTTTGGATACAAATGTTCATTTTCAAGAAACGTATGAATT

AATTCGAAAAGTGCGGGAACGATTTCCTTCATTGAATATTATAGAAAAACAGCCAGAACTTACACTTGATGAACAGGC

GAAGTTGCATGGTGAAAAACTGTGGGAGAGCAATCCGAATCTTTGTTGTAAGATTAGAAAAATTTTACCTTTAGAAAA

ATCATTAGTCGTGGAAAAAGCATGGATATCGGGCTTAAGAAGGGAACAATCAGAAACACGTAAGCATACAAAGTTTAT

AAATCAAGATCATCGTTTTCAATCTATTAAAGTTTGTCCACTCATTCATTGGACGTGGAAAGAAGTTTGGCGATATGTAT

ACAAACATAGCTTGCCGTATAACCCGTTGCATGATGTTGGCTATCCAAGTATTGGGTGTGAGAAATGTACGTTACCTGT

AGGGGATGGTGGCGATTCAAGAGATGGTAGATGGGCTGGAAAAGTGAAAACTGAATGTGGTCTTCATTACCAATAA

>NR1_01446 sulfate adenylyltransferase

ATGAGCATAGTAAACGAATTAGTAAACCGTATAGATGAGACATATGACGTATCACAAATTGAAAAAGAAATTAAATTA

GACAATATTGCGTTAAGTGATTTAGAACTATTGGCGACAGGTGGATATAGCCCCCTTACTGGTTTTTAGGGAAGGAAG

ATTATGATTCTGTTGTAGAAACGCTTCGTTTAGCAAATGGTAGTGTGTGGAGTATACCGATTACATTACCGGTAACAGA

AAAAGTCGCAGAAAGCCTGAAGGCTGGAGAGGAAGTAAAGCTTGTCAACAATGGAAATATATACGGTGTCATTCAAAT

AGAAGATATTTTTGTACCAGATAAAGAAAAAGAAGCGTTACTTGTATATAAAACAACGGATGAGGCTCATCCAGGAGT

GAAAAAATTATATGAACGACCAAACGTTTACGTTGGAGGAACTATTATTCTAACAAAACGCTTTGAAAATAATCAATTT

CCTTCTTATCATTTAGACCCAATTGAAACGAGGGAAGCATTTAAAAAACGTGGGTGGAAAACAGTGGTCGGATTTCAA

ACGAGAAACCCGGTACATCGTGCCCATGAGTATATTCAAAAATCTGCTCTTGAAATTGTAGATGGTCTCTTTTTAAATC

CGCTCGTTGGAGAAACGAAATCGGATGATATTCCTGCTGATGTAAGAATGGAAAGCTATGAAGTACTTCTTCAAAACTA

TTATCCGAAAAATCGTGTCTTTTTAAGTGTATTTCCTGCTGCCATGCGTTACGCAGGACCAAGGGAAGCGATATTTCATG

CGTTAGTAAGAAAGAATTTTGGATGTACCCACTTTATAGTAGGGCGTGATCATGCTGGAGTAGGAGATTATTACGGAAC

GTATGAAGCGCAAGAAATCTTCACAAATTTTACGATAGAAGAGTTAGGAATTACACCACTATTCTTTGAGCATAGTTTT

TACTGCACGAAATGTGAAGCGATGGCTTCAACGAAAACATGTCCGCACGGAAAAGAAGATCATGTTATTTTATCGGGT

ACAAAAGTAAGGGAATTATTAAGAAACGGTGAGATTCCGCCAAGTACATTTAGTCGTAAAGAAGTAGTGGAAGTGCTA

ATTAAAGGCTTGAAAACAGAAGTAGTAACAGAATAG

>NR1_01447 adenylyl-sulfate kinase

ATGGATACGAATATTACTTGGCATACAGCTTCGGTTTCAAAAGATGAGAGAAGAGTGAAGAATGGACATCATAGTTTT

GTAATTTGGTTTACTGGTTTATCAGCTTCGGGTAAATCTACAGTAGCGAATGCGGTCGCTCGTAAACTGTTTGAAAAGA

ATATTGGAAATTATGTGCTGGATGGGGATAACATTCGCCACGGTTTAAATAAAGACTTAGGATTTTCTGAAAGTGATCG

CATGGAAAATATAAGACGTATTGGCGAAGTGGCAAAGTTATTTGTAGACCAAGGAGCAGTTGTCCTTACAGCATTTATT

TCACCATTTCGAGTAGACCGCAAACAAGTAAGAGATTTATTAGCGGCAGATGAATTTATTGAAATTTTTGTGAAGTGTC

CGATTGAAGAGTGTGAGAAACGTGATCCGAAAGGACTTTATAAAAAGGCAAGAAAAGGTGATATTAAGGATTTCACAG

GTATTGATTCGCCTTATGAAGAGCCAGAGCAAGCGGAATTAATTGTAGAAACGCACAAATATTCTATTGAAGAATGTG

CAGAACAAATCGTGAAGTACTTACAAGAGCGTAGTTTTATATAG

>NR1_01448 ferredoxin--nitrite reductase

ATGAGTTATGAAAAAGTATGGGCTAACAATGAAAAATTAAATCAGACCGAGAAAAATAAATTAGAAAAAGATGGCTT

AGAAATTTTTAATGATATTCCTTACTATGCGGAAAATGGTTTCGAATCTATTCCGAAAGAAGAGTGGGATGCCTTTAAA

TGGGCAGGCTTGTATTTACAAAGACCGAAAGAAGCTGGTTATTTTATGATGCGGGTAAATATTCCGTCAGGGATTATTA

CAAATGCACAGGCAGAAGTACTTGCTTCTATCGCTGAAGATTATGGACGTAATGTAATAGATATTACGACGAGACAGG

CGATTCAGTTTCACTGGTTAGAAATTCAGCAAATTCCAGATATTTTTAAAAGATTAGCAAGAGTGGGGTTATCTTCAGC

GGGGGCTTGTGGTGATATAACGCGGAATATTACAGGTAATCCGCTTGCTGGCATTGATGCAAATGAACTATTCGATACA

ACGCATATTGTAAAAGAAGTATATGATTACTTCCAACATAATGAAGAGTTTTCTAACTTGCCACGTAAATTTAAAATGT

CTATTAGTTCTAATATTTATAATTCAGCAAACGCAGAAATTAACTGTGTTGCATTTACGCCTGCAACGAAAGAAATAGA

TGGTGAGAAAAAAGTTGGTTTTCATATAAAAGTAGGTGGAGGATTATCAGCTCGACCGTATTTAGCTGATGAATTAGAT

GTATTCGTTTTACCAGAAGAAGTGAAGGCGGTGGCGATTGCGATTGCTACTATATTCCGTGATTTTGGATATCGTGAAA

AACGTCATTTAGCTCGGTTGAAATTTCTTGTTGCGGACTGGGGAGCAGAGAAATTTAAAGAGAAACTAATAGAGTATA

CAGGGCCTTTACAAAGTAAAGGGGAAAGTGCACTCAAAGGATGGAATGCAGGCTATTTTTATGGTGTTCAAGACCAAA

AACAAAAGGGATTAAAATATGTAGGTTTTAATGTACCGGTAGGTCGTTTACATGCAGAAGAAATGTTTGAGATTGCAA

GAATCGCAAAGCAATATGGAAATGGACAAATTCGTACATGTAACTCGCAAAACTTCATTATTCCGAACGTTCCTCCGGA

AAATGTAAAAGGATTACTTAGTGAACCGCTGTTTGAAGCGATATCTGCTAGCCCGAAATCATTTATTGGTCATGCGGTT

TCATGTACCGGTATTGAATATTGTAATCTTGCACTAGTAGAAACGAAAGAAAGATTACGAAAAATTGCAGAATACTTA

GATACACAAATTGCACTCGATGTTCCAGTTCGAATTCATATGGTAGGCTGCCCAAATTCATGTGGTCAACGTCAAATTG

CTGACATTGGGCTACAAGGGGTGAAAATGAAAACGAAGGAAAAAGGTATTGTTGAAGCGTTTGAAATATACGTAGGTG

GAACATTGTTAGATGGCGGTGCCTACAATCAAAAGTTAAAAGGAAAAATAGATGGCGAGGATCTACCTGATGTACTCG

CATCATTTATAAGTTATTTCAAAGAGAATAAATTACCAGCCGAAACATTTTATGATTTTGTAGGTCGTGTTGGTGTAGAT

ACATTACAAATAGCGTTAAATAACGTGCTAGAAGAAGTAATAGCGTCTTAG

>NR1_01603 sulfurtransferase

ATGATAGTTACAGTCGAATGGTTACGTGAGCATATAGAAGATGAGAATGTCCGTATAATCGATTGTCGTTTTGATTTAG

CGAATCCTAATTGGGGTAGAGAAAAGTATGAAGAAGGACATATTCCTCATGCGTTATATTTTGATTTGAATTTAGATTT

ATCAAGTCCTATAGCAGAACATGGTGGCCGCCATCCTTTACCAAATATTGAGGAGTTTGCAGACAAGCTTTCGGAGGCT

GGTATTGATGAACATACGAAAGTAATTGCGTATGACAGTCAAGCCGGTGCAAATGCTGCTCGTTTATGGTGGTTATGTA

ACTATGTAGGACATGAGAAAGTATATATATTAGATGGTGGTTTCCCGGCTTGGAAAGAGAATGGACTACCGACAACAA

CGGAAATTCCTGTTGTTATACGAAAAACATTCAAAACAAACATACAAGATCATATGCTTGTAACGATGGAAACGGTCA

GAGAGAATATCCGTGCAGGTGCGGATGTTACATTAATTGATTCAAGAGAGCCGAAACGTTATGCTGGTGTAGAGGAAC

TTGTCGATCATAAAGCAGGTCATATTCCGACAGCGGTAAACCATTTTTGGAAGGATGGAATGTTGCAATCAGGACAATT

TAAGAATGGAGCTGGGCAACAAGAACGCTTCCAAAATCTTTCGAAAGATAAGGAAACCATTGTATATTGTGGTTCTGG

TGTTACAGCGTGCCCAAATATCGTTGCATTAAAATTAGCTGGATTCCAAAATGTTAAATTGTATGCAGGTAGCTGGAGT

GATTGGATTTCTTATCCAGAAAATCAAATTGCAAAAGAAGAAGATTAA

>NR1_01799 cysteine synthase A

ATGAAATTGTGTGAAAACGTTACGGAATTAATAGGAGATACACCTGTCGTCCGATTATCTAAATTTATTCCGGAAGACG

CAGCAGATGTGTATGTGAAACTAGAAATGTTTAATCCATCGCGCAGTGTGAAAGACCGTGCTGCTTATAATTTACTTCA

CGTTGCAGAAGAAAATGGTCTCATCAAACCAGGAGATACAATTATTGAACCGACAAGCGGGAATACAGGGATTGGTTT

AGCGATGAATGCAGCAGCTAAAGGGTATAAAGCGATTTTAATTATGCCAGATAATATGTCAAAAGAGCGTATTAATTT

ATTGAAAGCATACGGAGCAGAAGTAGTTTTAACACCAGCAGAACAAAGAATGCCAGGAGCAATTGCGAAGGCGTTAG

AACTGCAAAAACAAATACCGAATAGTTTTATCCCGCAACAATTTGAAAATCCAGCTAATCCGAATATTCACCGTTATAC

GACTGCACTTGAAATTTACGAACAAATGGATGGAGAGCTTGATGCTTTTGTTGCAACGGCAGGAACAGGCGGAACGAT

TACAGGGACTGGTGAAACGTTAAAAGAGAAATTGCCAAACTTATATATTGCAGTAGTGGAACCGAAAGGATCTCCCGT

TTTATCCGGTGGTGTACCAGGTCCTCATAAACTAGTAGGAACAAGCCCAGGATTTATTCCGAAAAACTTAAATACAGAA

GTGTATAACGAAATTATTCAAATTGCAGATGAAGAGGCTTTAACGACAATGAGAAACTTAGCTAGACAAGAAGGGTTA

TTAGTTGGGCCATCTTCTGGAGCATCTGTTTACGCTGCAATCATGATAGCGAAACGCCTAGGCGTTGGTAAAAAAGTTT

TATGTATTGCGCCTGATACAGGTGAACGTTATTTAAGCATGGGGTTATTTGAATAA

>NR1_01903 LLM class flavin-dependent oxidoreductase

ATGGAAAAATACCGTATAGATACAAGAAAAGGAATTGAGTTTGGATTATATTCAATTGGTGATCATGTTTTAAATCCAC

ATAATGGGGACAAAATTACGCCAGAAAAAAGAATTCACGAACTAATTGAAACAGCTAAGTTAGCAGATGAGGCAGGA

CTTGATGTGTTTGCTGTCGGCGAAAGTCACCAAACACATTTTACAACGCAAGCTCATACAGTTATTTTAGGTGCGGTCG

CGCAAGCTACGAAAAATATAAAAATTGCAAGTTCCGCAACGATATTAAGTACATCTGATCCAGTTCGAGTATATGAGG

ATTTCGCTACCATTGACTTGATTTCTAATGGACGTGCAGAAATTGTGGCTGGTCGTGGATCTCGTATTGGAGGATATAGT

TTACTTGGTTATGACGTGAATGATTATGAAGAATTATTTGAAGAGAAGATGGATCTTTTATTAAAAATTAATAACGAGG

AACATGTAACATGGAATGGACAGTTCAGAGCACCGCTCGCACATGCATCGGTTATTCCAAGAGCGAAAAATAATAACT

TACCAATTTGGCGTGCAGTTGGAGGTCCACCAGCTAGTGCAATTAAAGCAGGACGTGCAGGTGTGCCAATGATGATAA

CAACACTTGGTGGTCCAGCAATTAACTTTAAAGTGTCAGTAGATGCTTACCGCGAGGCTGCTCAGCAAAGCGGATTTGA

TCCAGCTAGTTTACCAGTTGCGACAACGAGTTTATTTTATACGGCAAAAAATTCACAAGATGCACTTAGTGAATATTAC

CCTCACATTAATGCTGGTATGCTTACACTGCGCGGTGATGGGTATCCGAAACAGCAATTTACAAATGCAATAGATTACC

GTGATGCTTTAATGGTTGGTAGCCCACAACAAATCATTGAGAAAATGCTTTACCAATATGAATTGTTTGGCCAACAACG

CTTTATGGCACAAATTGATTTTGGCGGTGTACCATTTGATAAAATTGAGAAAAATATTGAATTAATTGCTACTGAAATTT

TACCAGCCGTTAGAAAACATACAGCAAAATAA

>NR1_02812 FMNH2-dependent alkanesulfonate monooxygenase

ATGGAATTATTATGGTTTATTCCGGCTTATGGAGATGGTCGATATTTAGGAACGACAAAACGAGGAAGAGCAGCTGAA

TATGGTTATTATAAGCAAGTTGCTGCGGCAGCTGATTATTTAGGATATACGGGTGTTTTACTTCCAACTGGTCAAGGAT

GTGAAGACCCTTGGGTGTTAGCTTCAGCTCTTGCTGCTGAAACAGAAAAACTAAAATTTTTAGTAGCAGTAAGACCAGG

ACTAATGTCCCCTACAGTTGCAGCGAGGATGGCATCTACATTTGATAGAATTTCAGATGGGAGACTGCTTATTAATGTA

GTAGCTGGAGGAGATCCAGTTGAACTACAAGGAGATGGATTATATCTTAATCATGATGAAAGGTATGAAGCAGCGGAT

GAATTTTTAAAAGTTTGGAAATCGACATTGCAAGGTGAAACGATCTCTTTAGAAGGAAAACATATTCAAGTTACAGATA

GTAAAGTTGTATTCCCACCAGTTCAAACGCCATATCCTCCTATATATTTCGGAGGATCATCAGCTGCTGGAAAAGAAGT

AGCAGCTGAACATAGTGATGTATATTTAACGTGGGGAGAGCCGCCAGAGCAAGTAAAAGAAAAAGTAGAAGAAGTAA

GAAAACTTGCAGAAGAGAAGGGGCGTACAGTCCGATTTGGCATTAGATTACACGTAATTGTAAGAGAGACAGAAGAA

GAAGCATGGGAAGAGGCCGAACGTTTAATTCAATATGTGGATAATGAAACAATTGAGCTTGCTCAAAAGACATTTGCA

AGATATGATTCGGTTGGTCAAAAACGGATGACACATTTAAATAAAGGTACGAGAGAGTCGTTAGAGATAAGCCCTAAT

TTATGGGCTGGTATCGGACTTGTAAGAGGTGGGGCAGGTACTGCACTTGTAGGCGATCCGCATACAGTAGCAGAAAGG
ATAAAAGAATATGAATCGTTAGGAATTGATACGTTTGTTTATCAGGATATCCGCATTTAGAAGAAGCGTATGAAGTGG
CAGAATTGTTATTCCCATTATTGAAAGACAAGAAGAAAGAAGAAAATAAAATTGTTGGTGAAATGATTGCTGATGCAT
ATGCATTAAAAAAATAG

>NR1_02813 ABC transporter permease

GTGGAAAATACGAAAGCTGTTATGAAACCAGCGAGTATAACGATTGAGAAAAACAGAGTTAAGAATGTGCGAAAGTT
AAATGTAAAAGTTTTAGTAAGGGCGATTACTATACCGGTTATTATATTAATAATTTGGCAGTTAGCTGGCGTATTCGGC
CTTGTTTCTAAAACAGTTTTACCAACACCATTAGATATTTTTTTAGCTTTCCAAGAGCTTATTAAAACAGGAGAGTTATT
CGGACATTTAAGTATTAGTGTATTCAGGGCTGCGGCTGGTTTCTTTATTGGTGGGGGCTTAGGAATCATTTTAGGAACG
ATTGTTGGATTTTCAACGAGAAGTGAGCAATATTTAGATCCATCTGTGCAAATGTTAAGAACTGTACCTCATCTAGCTG
TTGCGCCACTTTTCGTATTATGGTTCGGTTTTGGTGAAACATCGAAAGTGTTATTAATTGCAGATGGTGCATTTTTTCCTT
TATACGTAAACGCATTTTTAGGTATTAGAGGTGTGGATTCAAAGTTATTTGATGTTGCGAGAGTGTTAGAGTTTAGCAA
AAGAAAACTAATTACGAAACTCATTTTACCATCTGCATTGCCTAACCTTTTACTAGGAGCAAGGTTATCGTTAGGTGTT
GCATGGGTGAGTTTAGTAGTAGCAGAACTTATGGGATCTACAGAAGGTATTGGTTATATGATTATGGATGCAAGGCAAT
TTTCAAATACAGATATCGTATTTGTCGGCATAATTATCTTTGCATTTGTTGGGAAGTTTTCTGACTCTCTAGTACGTTTAC
TAGAAGCGAAGTTTTTAAGATGGCGAGATAATTTTAAAGGTGAGACTGGGAATTAA

>NR1_02814 aliphatic sulfonate ABC transporter substrate-binding protein

ATGTATAAAAAATTTAAAATTCTTTCTTTTGCTTTAGCTATATCAGTATGTTTATTAGGATGTGAAAAAAGTACAGCAAG

TAGTAAGAAAGAAGATGTAACAATACAAATTGGTATACAGCAAGGGTTAAGCCCGCTATTACTAGCAAAGAAAAAGG

GGTGGTTTGAAGAGGAGTTTAAAAAAGAAGGAGTTAAAGTGAAATGGACAGAGTTCCAAAGTGGACCTCCTTATTTTG

AAGCAATCGCATCAAACCGATTAGACTTTGGTGAAGTAGGGAATTCTCCAGTCATTTCAGCGCAAGCAGCAGGTATTG

GATTTACCGAAATCGCCAATACAAGTTATGCGAGAAAAGGAACTGGAATTCTCGTTCAAAAAGATAGCAAGATTGCGA

GTGTAAAAGAGTTAAAAGGTAAAAAAATTGCAGTAGCAAAAGGAAGTAGTGCCTTTAATTTATTGTATCGAGCACTTG

ATAAAGAAGGGATTGATGCAAAAGAGGTAAATGTCATTCAATTACAACCAGATGAAGCGCAGCCTGCATTCGAATCAG

GATCAGTAGATGCATGGGCGATTTGGGACCCTTTCATATCATTACATACGTTAAATAAAGGTGCGAAGGTGATTGCAGA

CGGTGAAACATTAAATGTCTCTTCACCAGAATTTTTAATTACGAGAACAAAATTTGCGAAAGAACATCCAGAATTAGTT

GAGAAATTCCTCAAAGTTTATGAGAAAGCACGTGTATGGCAAGATGCTAACTTAGATGAAGCAATAAAAGTATATACT

TCTGTAAAAAAGATAGATGCGGAGATCGTAAAAGAAGTGTTTAATCACGACAAACCAATTTTAGTTCCGGTAACGAAG

GAAATAATAGCAGAACAACAAAAGACAGCGGATTTTCAATATAAGCTCGGTTCTATAAAGAAAGAAATTAAGGCGGA

AAAAGTTGTAGATAATTTTTTCGTAGAAAAAGCATTGAAAGCGAAGTGA

>NR1_03411 NAD(P)/FAD-dependent oxidoreductase

ATGAAGACCAGAGATAGTTATAAAATTATTGTAATTGGTGCTGGGACAGCAGGACTATCTTCTACTGCACATTTATTAC

GAAATGTACCCCTATTGAAAGAAAGTATAGCAATTATTGATCCATCAAAAAAACATTACTTTCAGCCACTATGGAGTTT

AGTGGGGGGAGGAATTGTTTCAAAGGAAAGTACGATGCGTAATCAGGAATCGCTTATTCCAAAAGGGGCAACGTGGAT

CCCTAAAAGTGTTGTTGAGTTATTTCCATCTGAAAATAAGATACTTTTAGATGACGGACTGCTACTTGAGTATGAAATTC

TTATTGTAGCAGCTGGTATCCAAATAAATTGGGACAGTATTAAAGGCTTAAAAGAGTCTATTGGCACTAATGGGGTATG

TAGCAATTATTCTTATACATATGTTGATTCTACTTGGAGAGAAATTGAAAAATTTAAAGGAGGAAATGCGCTTTTTACC

CACCCTAATACTCCTATTAAATGCGGTGGTGCTCCACAAAAAATTATGTACTTAGCAGAGGAGTATTTTTGTAATAGTG

GTGTAAGAAACAGAAGTAAAGTAATATTTTATTCTGCAAACAATAACATATTTCAGGTTCCACGATATGCAAATACTTT

AGAACAAGTACTAGAAAGAAAGCAAATTATAACGAATTATAATAAAAATCTAGTGGAAATTATCGCCGAAAAGAGAG

AGGCAATTTTTGAAGATACGCAAACACTAAAAAGAGAAACCGTACCATATAGTATGATACATGTTGTTCCGCCAATGG

GGCCACCTAATTTTATTAAAGAGAGTGAGATAAGTGATCATCAGGGGTGGGTAGATATAAGCCCTTATACTTTGCAGCA

TGTGCAATATAAAAATATTTTTGGACTTGGAGATTGTACCAATTTACCTACCTCTAAAACTGGAGCAGCAATTCGAAAA

CAAATACCCGTCTCAAAACAAAATATTATGGACGTACTTAGCGGAAGAGATTTACAGGCTAAATATGATGGATATACA

TCATGTCCGATTGTTACAGGATATAAAAGTCTTATACTTGCTGAATTTAACTATGAACATGAGCCTCAAGAAATGTTTCC

ATTTAATCAAGCGAAAGAACGGTATAGTATGTTTTTACTTAAAAGATATATGTTGCCATATATGTATTGGAATTTAATGT

TGAAAGGGATTCTATAG

>NR1_04202 rhodanese-like domain-containing protein

GTGTCAACAACTTGGATTATTTTATTAGCCGTAATCGTAGCATTTATCGGCTACACTGTATGGATGTACTTCTATCAGAA

AAAATTAATTAAAACTCTTACAGAAGAAGAATTTCGCGCTGGCTACCGTAAAGCACAGCTTATCGATATTCGTGAAGCA

GACGAATATAACGCAGGGCATATTTTAGGTGCACGTAACATTCCGTTATCACAAATTCGCCTTCGCCATAAAGAACTTC

GTCAAGATCAACCTGTTTACTTATATTGCCAAAGCGGATTCCGTACAGGTCGTGCAGCGCAATACTTAAAAAAACAAGG

CTACAAAGATTTCTACCAATTACAAGGTGGATTTAAATCTTGGACAGGCAAAATTAAAAAGAAATAA

>NR1_04251 cystathionine gamma-synthase/O-acetylhomoserine thiolyase

ATGTCAACAATCGAAACAAAACTAGCACAAATCGGAAATCGCAGTGAAACTACAACCGGAACGGTTAATCCACCAGTT

TATTTCTCAACCGCTTATCGTCACGAAGGAATTGGTAAATCTACTGGCTTTGACTATTCACGAACTGGCAATCCAACTCG

AGGTCTTTTAGAACAGGCGATCGCAGACTTAGAATATGGCGAACAAGGTTATGCCTGTAGTTCAGGGATGGCGGCTGTT

CTCCTCGTCCTTTCTCTATTCCGCTCTGGAGACGAACTTATTGTATCTGAAGATTTATACGGGGGAACGTATCGATTATT

TTCTGAGCATGAAAAAAAGTGGAATGTTCGATGTAGATACGTAAATACACAGTCTATTAAACAAATTGAGCAAGCTAT

CACAACTGAAACGAAGGCTATTTTCATAGAAACTCCAACTAATCCATTAATGCAAGTTACTGATATTGCTGCTGTCGCA

ACTGTAGCGAAAAGGAACGGACTACTCCTTATTGTAGACAACACTTTCTACACACCTTATATACAGCAACCATTAACAG

AAGGTGCTGACATTGTACTTCATAGCGCAACGAAATATTTAGGGGGACATAACGATGTACTAAGCGGGCTTGTTGTTGC

AAAAGGAAAGGAACTTTGCGAGGAAATCGCTCATTATCATAATGCATCAGGTGCAGTTTTAAGCCCATTTGACTCATGG

TTATTAATTCGCGGTATGAAAACGTTAGCGCTTCGCATGAGGCAACATGAAGAAAATGCGAAAGCAGTTGTTGCGTATT

TAAATGATGAAGACGGGGTGACAGATGTATTTTATCCAGGAAGAGGTGGCATGATCTCATTCCGCCTTAAAGATGAAG

CTTGGATTAATCCATTCTTACAATCTTTATCCTTAATTACATTTGCCGAAAGTCTTGGTGGTGTAGAAAGTTTAATGACT

TATCCAGCAACGCAAACACATGCTGATATTCCTGAAGAAATTAGAACAGCAAACGGCGTATGCAATCGTCTTCTTCGAT

TCTCAGTTGGCATTGAAAATAGTAACGATTTAATTCAAGACTTAAAGCAAGCAATTAAACTCGTAAAAGAAGGTGTAA

GAATATGA

>NR1_04252 cystathionine beta-lyase

ATGAGCTATTCTATAGATACACTCTTACTACACAACCAATATAAACACGACTCGCAAACAGGCGCTGTGAACGTTCCTA

TTTATAACACATCAACTTTTCACCAGTTTGATGTAGATACGTTCGGAAAATATGACTATAGCAGGTCAGGAAACCCAAC

TCGTGAAGCTCTTGAAGATATCATTGCTTTATTAGAAGGCGGAACAAAAGGATTCGCCTTTGCATCAGGCATTGCAGCG

ATTTCCACTGCATTCCTCCTTCTTTCACAAGGTGATCACGTACTCATTTCAGAAGATGTGTACGGAGGCACTTACCGAGT

GATAACTGAGGTACTCTCCCGTTACGGTGTTTCACATACATTTGTTGATATGACCAATTTAGAAGAAATAAAACAAAAC

ATTAAACCAAATACAAAGCTCTTTTATGTAGAAACACCTTCTAACCCACTTTTAAAAGTGACAGATATTCGCGAGGTTT

CTAAACTCGCAAAATCTATTGGGGCTCTTACTTTTGTTGATAATACATTTTTGACACCACTATTCCAAAAACCACTTGAA

CTTGGAGCTGATGTCGTTCTTCATAGTGCTACAAAATTTATTGCTGGTCATAGTGATGTTACTGCCGGATTAGCAGTAGT

AAAAGATTCAGAACTCGCTCAAAAGCTTGGATTTTTACAAAATGCATTCGGTGCTATTTTAGGACCTCAAGATTGTTCT

CTCGTACTTCGCGGTCTAAAAACATTGCATGTACGCCTCGAACATTCAGCTAAAAATGCCAATAAAATCGCACATTATT

TACAAGAGCACTCTAAAGTAAAAAATGTCTATTATCCGGGATTACAAACTCATCTCGGATTTGATATTCAACAATCTCA

AGCAACATCAGCTGGAGCGGTCTTATCGTTTACTTTGCAGTCAGAAGATGCACTCCGCAAATTTTTATCAAAAGTAAAA

TTGCCTGTTTTTGCAGTTAGTTTAGGAGCTGTAGAGTCAATTCTTTCTTATCCAGCAAAAATGTCACATGCAGCACTGTC

GCAAGAAGCTCGTGATAAAAGAGGAATCTCTAATTCATTACTTCGGTTATCAGTCGGTCTTGAAAATGTTAACGATTTA

ATATCCGATTTTGAAAATGCCCTTTCTTATGTAGAAGAACCTGTAAATGCATAG

>NR1_04343 DoxX family protein

ATGGTTATTCAATTTTTAAGAGAAAACAAAGCAGTTTCTTTTGCATTAGCAGTAATTCGAGTATATCTTGGTTACACATG

GTTAATGGCTGGAATCGGTAAACTACAAGGAAAAGGATTCGATGCAACAGGTTATTTGCAAGGGGCAATTGAAAAATC

TAAAGGTGCACAACCGGCTGTTCAATCTTGGTGGGCATCATTCTTACAAGAATTTGCAATTCCAAATGTAGATTTGTTTA

ATACACTTGTAACATGGGGAGAAATTTTAGTCGGAATTGGTTTAATTGTAGGCTGTTTAACAAAAACAGCGGTCTTTTT

TGGTCTTGTAATGAACTTTTCCTATATGTTTAGTGGGTCAATTGGTGTGAACCCTGAGATGGTTATCTTATCTATGTTTGT

TCTTGTTTCCGGTATGAACGCTGGGAAATTTGGAATGGATGGCTTCGTTATCCCGAAAGTATTAGGATCAAAAACTCCA

AAACGCCAAAAGCAAGCTGCTTAA

>NR1_04366 bifunctional cystathionine gamma-lyase/homocysteine desulfhydrase

ATGAGAGCAAAGACAAAGTTAATTCATGGTATTCGCATAGGAGAACCTTCAACTGGATCTGTAAACGTACCGATTTATC

AAACAAGTACGTACAAACAAGAAGCAGTTGGTAAGCATCAAGGATATGAATATTCACGTACAGGTAACCCAACACGTG

CAGCTTTAGAAGAAATGATTGCTGTATTAGAAAATGGGCATGCTGGATTTGCATTTGGTTCAGGAATGGCTGCTATTAC

AGCGACAATTATGTTGTTCTCAAAAGGTGACCACGTTATTTTAACAGATGATGTTTATGGCGGAACATACCGCGTTATT

ACAAAAGTATTAAACCGTTTCGGTATTGAGCATACATTTGTAGATACAACAAACTTAGAGGAAGTTAAAGAAGCGATT

CGTCCAAATACGAAAGCAATTTATGTGGAAACACCAACGAACCCACTACTAAAAATTACTGATATTAAGAAAATATCT

ACTCTTGCTAAAGAGAAAGACTTATTAACAATTATTGATAACACATTCATGACGCCATATTGGCAGTCGCCGATTTCTTT

AGGAGCAGATATTGTGCTTCATAGTGCAACGAAATATTTAGGAGGTCATAGTGACGTAGTTGCAGGTCTAGTAGTTGTA

AATAGCCCACAACTAGCAGAAGATCTTCACTTTGTACAAAACTCAACAGGAGGTATTCTTGGGCCGCAAGATAGCTTCT

TACTACTTCGTGGTTTAAAAACATTAGGAATTCGTATGGAAGAACATGAAACGAATTCACGCGCTATTGCTGAGTTCTT

AAATAATCATCCAAAAGTAAATAAAGTATATTACCCAGGTCTTGAATCACATCAAAACCACGAATTAGCAACAGAACA

AGCAAATGGATTTGGTGCTATTATCTCATTTGATGTAGATAGTGAAGAGACATTAAATAAAGTACTTGAGAAATTGCAG

TACTTTACACTTGCTGAAAGTTTAGGAGCAGTAGAAAGTTTAATTTCTATTCCATCTCAAATGACACATGCATCAATCCC

AGCTGATCGCCGCAAAGAATTAGGAATTACAGATACATTAATTCGTATTTCTGTCGGTATTGAAGATGGTGAAGATTTA

ATTGAAGATTTAGCACAAGCGCTAGCATAA

>NR1_04367 O-acetylserine dependent cystathionine beta-synthase

ATGAATGTATATCGTGGAGTTCATGAGTTAATTGGTCATACACCAATTGTAGAAATTACTCGTTTTTCACTTCCAACAGG

GGTCCGTTTATTTGCAAAGCTTGAATTTTATAATCCAGGCGGAAGCGTTAAGGATCGTTTAGGAAGAGAATTAATCGAA

GATGCGCTAGAAAAAGGGCTTGTTACCCAGGGTGGAACAATTATTGAACCGACTGCTGGGAATACTGGTATTGGACTG

GCACTTGCAGCATTAGAACATGATTTACGTGTTATTGTTTGTGTACCAGAGAAATTTAGTATTGAAAAACAAGAATTAA

TGAAAGCACTAGGTGCAACTGTCGTGCATACACCGACTGAGCAAGGAATGACCGGTGCAATTGCCAAAGCAAAAGAGT

TAGTAAATGAAATACCGAATTCATACTCTCCAAGTCAGTTCGCAAATGAGGCAAATCCTCGTGCATATTTTAAAACATT

AGGACCTGAACTGTGGGACGCATTGAATGGAGAGATTAACATATTTGTTGCTGGAGCAGGAACTGGCGGTACATTTAT

GGGGACTGCATCTTATTTGAAAGAAAAAAATATAGATATTAAAACAGTTATCGTAGAGCCAGAAGGATCTATTTTAAA

TGGTGGTAAGGCTGGTTCACATGAGACCGAAGGAATTGGACTGGAATTTATCCCGCCATTTTTGAAGACATCTTATTTT

GATGAAATTCATACGATTTCTGATCGAAATGCATTTTTACGAGTGAAAGAATTAGCGCAAAAAGAAGGACTTCTCGTTG

GGAGCTCTTCAGGAGCAGCATTTCATGCGAGCTTACTTGAGGCAGAGAAGGCAGCACCAGGTACAAATATTGTAACCA

TTTTTCCTGATAGCAGTGAGCGCTATTTAAGTAAAGACATATACAAAGGATGGGAATAA

>NR1_04617 bifunctional oligoribonuclease/PAP phosphatase NrnA

ATGCATGAGCAAATTTTAGGAGCAATTAAAGAGTTTGATACAATTATTATTCATCGCCACGTGCGTCCGGATCCAGATG

CGTTAGGTTCACAGGGTGGTCTTGGTACAATTCTACAAGAATCGTTTCCAGAGAAAAATATTTATACGGTTGGGTACAA

TGAGCCGTCACTAGCATACTTACGAGTAATGGATGATATTCAAGATAGTGTATACGAAAATGCGCTTGTTATTGTTTGT

GATACTGCGAATCAAGAACGTGTTGACGATCAACGCTATACAAAAGGGAAGATGTTAATTAAAATTGATCATCATCCA

AATGAAGATCCATATGGAGATATTACGTGGGTAGATACGACAGCGAGTTCTACAAGTGAAATGATTTATGAGTTTTATA

ACTATGGGAAAGATAAAGGATTAAAAATAACAAAAGAAGCAGCCCGCCTTATTTTAGCAGGAATTGTTGGAGATACAG

GTCGTTTCTTATTCCCGAATACGACAGCAAAAACACTTCGTTACGTAAGTGAGCTTGTTGACATGGGTGTGAAATTCAC

AGATTTATACAATGAGATGTATAAGACAAAAGAAAAAATTGCTCGTTTAAACGGTTATATTTTACAAAACTTTACGATG

GTAGAAGAAGGAGCAGCTTACATTAAATTAACGAAAGAAGTATTAGAAGAGTTTGATGTACTTCCTTCTGAAGCATCT

GGTGTTGTTGGAGCGCTTGGCAATATTGATGGATTAAAGGCGTGGGTTCTATTTTTAGAGGAAGACGACGTAATTCGTG

TTCGTCTTCGTTCAAAAGGACCAGTTATAAACAAATTAGCAATGCAATATAACGGCGGGGGCATCCGATGGCTTCTGG

TGCGAAGGCATCTTCTTGGGAAGAAGCGGATCGTCTTTTTGCTGATTTACGTGAGATTTGTAAATAA

>NR1_04665 methionine gamma-lyase

ATGAAAAAGAAGCATATGGAGACAGCGTTAATTCATCACGGTTATAAACCTGAGGAATATAAAGGAAGTTTAACACCA

CCTTTATTTCAAACTTCTACATTTACATTTGAGACTGCGCAGCAAGGAGAGGCGAGTTTTGCGGGAGTGGATCCATCTT

ATATTTACTCACGGCTTGGAAATCCAACTGTGAAATTATTTGAAGAACGTATGGCGGTGTTAGAAGGAGGAGAAGAGG

CACTTGCCTTCGGGTCTGGTATGGCAGCTATTTCAGCGACTTTAATCGGTTTTTTAAAGGCTGGAGATCATATTATTTGT

TCAAATGGATTATACGGATGCACGTACGGTTTTTTAGAAGTGTTAGAAGAAAAATTTATGATTACGCATTCGTTTTGTG

ATATGGAGACAGAGGCTGATATTGAAAATAAAATTCGCCCAAATACAAAGCTTATTTTCGTTGAAACACCAATTAATCC

AACGATGAAATTAATTGATTTAAAACAAGTGATTCGGGTTGCGAAGCGAAAAGGCTTACTTGTTATTGTTGATAATACA

TTTTGTTCACCTTATTTACAAAGACCGCTTGAGCTTGGCTGTGACGCTGTTGTGCATAGTGCGACAAAATATATTGGTGG

TCACGGTGACGTTGTAGCGGGCGTAACAATTTGTAAAACGAAAGCATTAGCTGAAAAGATTCGCCCGATGCGAAAGGA

TATCGGCGGTATTATGGCACCGTTTGATGCGTGGTTATTATTACGCGGGTTAAAGACGTTAGCAGTAAGAATGGATCGT

CATTGTGATAATGCAGAAAAAATTGTATCGTTCTTAAGAAAACATGATGCGGTAGAAGGTGTTTGGTATCCAGAAGGA

GAGTTAGCATCTCGTCAAATGAAACGCGGGGGCGGTGTTATTTCTTTTTCAGTAAAAGGCGGAAAAGAAGAGACGCAA

GCGTTTATTAATGATCTTCACTTTATTACAATTGCGGTGAGTTTAGGAGATACAGAAACGTTAATTCAGCATCCAGCAA

CGATGACGCACGCTGCGATTCCAGCTGAACTAAGAAAAGAAATGGGCATTTTCGATAATTTAATACGCTTATCAGTCGG

TTTAGAATCGTGGGAGGATATCGTTTCTGATTTAGAGCAGGCATTAAAGAAAATATCTACTGTTAATCAATAA

>NR1_04736 rhodanese-like domain-containing protein

ATGACAGAAGTTAAAACAATTACTACAGAAGAGGTACAAGAGCGTTTAGAAAATGGAGAAACGTTATTTTTAGTAGAC

GTAAGAGAAGATGAAGAAGTAGCGGCAGGAAAGATTCCAGAAGCTGTACATATTAAAATGGGCGATATCCCACATAA

AGTAGATTTCTTTAATAAAGAGAATGAATATATCTTTATTTGTCGTTCAGGAATGCGCAGTGAAAATGTATGTCATTATT

TAAATGAGCAAGGATTTAAAACAGTGAATATGGTTGGCGGTATGCTTCAATATGAAGGTGAAACGAAATAG

>NR1_04757 PepSY domain-containing protein

ATGAAAGTAAATCGTTCGCTTCATTACATTCTTTGGCGTTGGCATTTTTATGCTGGGCTTTTTATTACGCCGCTTCTTATT

ACGTTGTCACTGAGCGGAATTGGGTATTTATTTCGGGAGGAAGTTGAAGATTTCATCTATAAAGATTTATATTTTGGGA

AGAGTGCTCAAACAGAATCTATTTCGATGTCTGATTCTATTTCCTTAACAGAGAAAAAATATCCACATTATAGCGTGGC

GAAAATTAGTGAGTTTAATGGGGATTATAATACGAGACTTACAATTGCAAATGAGTATACTGGGCAACAAAAATATGT

GTATTTAGACAGCAATAATCAAATTGTTGGGGATCAAAACGCAAGTGAAACATTTGCCAATATAATGAGGGAATTACA

TAGTTCTCTTTTAGTTGGTGGCACTGTCGTAAACTATACTGTAGAACTCGCGGCATGCTGGACAATCTTTTTAATCGTAA

CCGGATTGTACATGAGTATACGACAATTCAAAAACACACCATCATCCAATAAGCGAGAAAAAGCAAAAAGACGTCATT

CTATTATCGGTATTATATTTACAATTCCTCTCTTTCTGCTAGTCGCATCTGGATTGCCATGGTCAGGATTTATGGGGAAC

CAAATTTATAAAATCGCATCGTCAAATGAATCACTCGGATATCCAAAATTGTACATGGCTCCGCCTGAATCAAAGGTAA

AAGAATTGCCGTGGGCAACAAGAAAAGAAGCTCCGCCTGAATCAAATTCAAATGAACCGAAAGCAATTTCTGTCGATG

AATTACAAAAAGGAATTGAAATAAAGAAGCCATATGTTATTTCACTACCAGCTGATCCGAAAGGTGTATTCACTGTTTC

GAAATCGAGCGGTTCTGGTATTACAGGTATGCATGTTGCACCAAATGAAGAGATAACAGCTTACTTTGACCAATATAGC

GGGGAGCTCATTTCAAAAACGGACTATCGTGATTATGGATTACTTGCACAATGGTTCACTTACGGTATCCCGCTTCATG

AAGGACATTTATTCGGATGGCCAAATAAAATATTATGCTTACTAACGACATTATCTCTACTACTTCTCATTTATTACGGA

ATAAAAATGTGGTTAGCAAGAAAGCCGAAAGGAAAATTAGCAGCACCTCCAAAACAAAGAGATAAGAAAAGTATATT

CGTTTTCTTTATCATGATGGTGATACTAGGCGCTGTCATGCCTTTATTTGGACTATCTGTTTTAGTTATTTTTACAATTGA

ACTTCTTATATATGTATTTTTAAAAATACGATCATAA

>NR1_04761 gamma carbonic anhydrase family protein

ATGATATATCCTTACAAAGAAAAAAAACCGAAAATTGCGAGTAGTGCTTTTATCGCTGACTATGTTACGATTACAGGCG

ATGTTTACGTTGGCGAGGAATCAAGTATTTGGTTTAATACAGTCATCCGTGGTGATGTGTCACCAACAATCATTGGAGA

CCGAGTAAATGTACAAGACCAATGTACACTCCACCAAAGCCCTCAGTATCCTCTTATCTTAGAAGATGATGTTACAGTT

GGGCATCAAGTTATTTTACATAGTTGTCATATTAAAAAAGATGCTTTAATTGGAATGGGATCCATTATATTAGATGGTG

CTGAAATTGGCGAAGGAGCTTTTATCGGCGCTGGAAGCCTCGTTTCACAAGGAAAGAAAATTCCACCAAACACGTTAG

CTTTCGGTCGTCCCGCGAAAGTCATTCGTGAATTAACAGCAGAAGACCGTAAAGATATGGATCGTATTCGCACGCAATA

CGTTGAAAAAGGTCAATATTATAAATCATTACAGAAGTAA

>NR1_04785 ABC transporter permease

TTGGATAATATAAAACAACTACATGAACAATTTCGAAAGAACGAACGCAGACGCGCTTGGATTGCTCGCTCGCTACAA

CTTTTACTACTTATTCTTTTCTTTGCACTATGGGAAATAGCTAGCAAAAAAGAATGGATTGATCCTTTACTCTTTAGCTCT

CCTTCGAGTATTTGGGATCTCTTCTTAACGAAATGGATCGACGGTTCACTTTGGGTCCACATATGGACGACATTGCTGG

AAACAGGAGTAGGCTTCATTCTCGGAACGGTACTCGGTGCTATTATTGCCACGTTCCTTTGGTGGATGCCACTTCTAGCC

CGCGTACTTGATCCTTACCTCGTCGTCCTAAATGCAATGCCAAAAGTTGCACTCGGTCCAATCATCATCGTTATTTTCGG

TCCAAACATTTCATCTTCTATCGCAATGGGAGTAATCATTTCCATCATCATTACCATTCTCGTTATTTACAGTGCATTTCA

AGAAGTCGATTCTAACTATATAAAAGTGATGGACACATTTGGCGCAAATAAATGGCAATGTTATAAGCAAGTCGTTCTC

CCTGCATCCTTTCCAGCAATTATCTCAACGTTAAAAGTAAATGTTGGTTTATCCTGGGTTGGTGTTATTTTCGGAGAACT

TCTCGTTTCCAAACAAGGACTTGGCTACTTAATTAGCTACGGATTCCAAGTCTTTAACTTCACACTCGTCTTACTTAGCG

TACTACTCACATGTGTTCTTGCAACTCTTATGTATGTATTTGTTGAGGCATTTGAAAAAATTCTAATTGGAAAAAGAAAA

AGAAGCTGA

>NR1_05337 (Fe-S)-binding protein

ATGAATAGCTTACTGATCATTAATTGGCTGGCTGCCATTGCTGTTATTGCTTATGCAGGATATTTGTTTGTATATCTTATA

CGGACGAGAATGGCCTACATACAATTAGGAAAAAAAATTGAATTTGACCGTCGTTTTAAAGAGCGTTGGGATCTCCTTA

AGGTCAATGTTTTCGGTCAAAAAAAGCTGCTGAAAGATAAGAAAAGCGGCATCATTCACGTTATGTTCTTTTACGGATT

TATTCTTGTCCAATTTGGAGCAATTGACTTCGTTTGGAAAGGACTCGCACCAGGATCACATCTTCCACTTGGACCACTAT

ACCCTGCATTTACATTCTTCCAGGAAATTGTCACACTTGTTATTTTAATTGCAGTATTTTGGGCTTTTCATAGACGTTATG

TAGAAAAGCTTGTTCGTTTAAAACGTAACTTCAAATCAGGTCTTGTTCTTATCTTTATCGGTGGCTTAATGATTTCTGTG

CTACTTGGTAACGGTATGGGACTTATATGGCATGGCGAGGAGCTTTCATGGAGTGAACCAATTGCTTCTGCAATCGCTT

ACGTTTTTAGCGGGATAAATGAAACCGTAGCCATTTCAGTGTTCTATTTTTCTTGGTGGGTGCATTTACTAATTTTGTTA

ACGTTTTTAGTGTATGTTCCACAATCAAAACATGCGCATTTAATTGCAGGACCAGCTAATGTATTCTTCGGTCGTCTTTC

AAACCCAGGGAAGCTTGAAAAGATTGATTTTGAAGATGAAACGCAAGAAACATTTGGTGTTGGTAAAATTGAAGACTT

TAGACAAAATCAACTTATTGACTTATACGCTTGTGTAGAGTGTGGCCGTTGTACAAATATGTGTCCGGCAACAGGAACA

GGAAAAATGTTATCGCCGATGGACTTAATTTTAAAACTTCGCGATCATTTAACTGATAAAGGAGCTGCGGTAACATCAA

AAGCACCGTGGGTTCCAGTAGTTGCCTTCAATAATACACAAGGAAATCAGTTAGCGATGATGGCAGCTGGAAAAGGAC

AACAAGAATCAGCGTCTACAACGCTCGCTTACGATCCGAGTTTAATCGGAGATGTTATTACAGAAGAAGAGATTTGGG

CATGTACAACGTGTCGTAACTGTGAAGATCAATGCCCAGTTATGAATGAGCATGTTGACAAAATTATTGATTTACGTCG

ATATCTCGTTTTAACAGAAGGAAAAATGGACGCGGAAGCACAACGCGCGATGACAAATATCGAGCGTCAAGGGAATCC

GTGGGGTCTGAACCGTAAAGAGCGTGAAACATGGCGCCAAGGTGATGACGAAGTAACAGTTCCAACTGTAAAAGAAA

AATCAAAAGCTGGGGAGGAATTCGAGTATTTATTCTGGGTTGGTTCAATGGGATCATACGACAATCGTAGTCAGAAGA

TTGCGATATCGTTTGCGAAGTTAATGAACGAAGCAGGCATTTCATTCGCAATTCTCGGTAATAAAGAAAAGAATTCTGG

AGATACACCACGCCGCCTCGGAAATGAATTTGTATTCCAAGAGATGGCGACAAAGAATATCGAAGAATTTGAAAAGGC

AGGAGTGAAGAAAATCGTTACGATTGATCCTCATGCTTATAACACATTTAAAAATGAGTATCCGGACTTTGGCTTGCAA

GCAGAAGTCTATCATCATACAGAATTGTTAGCTCAGTGGGTGAAAGAAGGGCGCTTAAAGCCTGTTCACGCTATTGAA

GAAACAGTTACGTACCATGATTCCTGTTATTTAGGAAGATACAACGAAGTGTACGAAGCGCCACGTGACATTTTGAAA

GCGATTCCTGGAGTGAATCTTGTAGAAATGGCACGTAACCGTGAAACTGGAATGTGCTGTGGCGCAGGTGGTGGCTTA

ATGTGGATGGAAGAAACAACAGGTTCTCGTATTAACGTTGCTCGTACAGAACAAGCATTAGCTGTACAGCCATCCATTA

TCGGTACAGGTTGTCCATATTGCTTAACGATGATCAGTGATGGAACGAAAGCGAAAGAGGTAGAAGAGAAAGTTCAAA

CTCTTGATGTGACAGAGATTTTAGAACGATCTGTTATCGGACAGAAAAAAGAAGCAATGTAG

Section 3

NM1-A2 Amino acid sequencing

>NM1_A2_00094 cysteine synthase A

MARIANSITELIGQTPIVKLNRLVEEDMADVYLKLEFMNPGSSVKDRIALAMIEDAEAKG

VLKPGDTIIEPTSGNTGIGLAMVAAAKGYRAILTMPETMSIERRNLLRAYGAELVLTPGP

EGMKGAVNKATELAKEHGYFIPQQFQNEANPEVHRQTTAKEIIEQFGDQLDGFVAGIGTG

GTITGAGEVLKEKYPNIKIYAVEPADSPILSGGQPGPHKIQGIGANFVPDTLNTEVYDEV

VAVQNDQAFEYARKVAKTEGVLVGISSGAAIYAALKAAKQLGKGKKVLAIIPSNGERYLS

TPLFQFEEQTQA

>NM1_A2_00115 serine O-acetyltransferase

MMLKLLKEDIEAIFEQDPAARSYIEVILTYSGLHAIWAHRFAHGLFKRKWFFLARVISQI

SRFFTGIEIHPGAKIGRRFFIDHGMGIVIGETCEIGDNVTVYQGVTLGGTGKEKGKRHPT

IEDNVLIATGAKVLGSITIHAHSKIGAGSVVLNDVPENSTVVGIPGRVVIQNGVRIPKDL

NHQDLPDPDAERFKQLEKEILHLQNQLKELKEGKIHHGN

>NM1_A2_00856 homocysteine synthase

MSAKKPFRPETQAIHSGQQLDPATFSRAVPIYQTSSFGFKDTEHAASLFNLSEQGYIYTR

IVNPTTDVFEQRIAELEGGVGALGVASGQSATTFSILNIASAGDEIVSASSLYGGTYNLF

SSTLPKLGITVKFVNADNPENFRSAITSKTKAIYAESVGNPQGNVLDIEAVADIAHEHGI

PLIIDNTVPSPYLLRPIDFGADIVVHSATKFLGGHGTAIGGVIVDSGKFDWEASGKFPDL

TTPDPSYHGLVYTEAAGEAAYITKARVQLLRDIGAALSPFNSFLLLQGVETLHLRLERHS

ENALKVAKFLEQHELVDWVHYAGLPSHPSYSLAQKYLPKGQGAILTFGVRGGKNAAAKLI

DSVQLFSHLANIGDSKSLIIHPASTTHQQLSEDEQKASGVTPELIRLSVGTEAIDDLLED

LDYALKASQKVNTTV

>NM1_A2_00911 FMNH2-dependent alkanesulfonate monooxygenase

MELFWFIPTYGDGRYLGSHEGARAASYSYCKQVAQAADELGYSGVLLPTGKSCEDAWIAA

STLVPVTENLKFLVAVRPGLMSPTQAARMAATFDRFSKGRLLINVVAGGDPVELEGDGVF

LNHHDRYELTDEFLTIWRRVLNESDVHFDGDYLSVKGGDVLYPPIQKPYPPLYFGGSSPV

AMDVASRHIDVYLTWGEPPAQVKEKIERMKEKARQTGREIRFGIRLHVIVRETEEQAWKA

ADELIQHVDEEAVKSAQKVFSRMDSEGQKRMSALHQGDRSNLEVSPNLWAGVGLVRGGAG

TALVGAADTVAQRIKEYAELGIETFILSGYPHLEEAYRTAELLFPRLPVQRKQDENERTF

ISPFGEVKVNDKAPAKR

>NM1_A2_00928 ABC transporter permease

MKKYGASVILVVILLAAWEIGARIVNYPFILPTPSGILTKLWELRMDLLFKHLPATLSIV

VIGLVISVVLGVLLAVWMNWSPLIERAFYPLIIASQMIPTIAIAPVFVLWFGYSIWSKVI

VTVLITFFPITVSTFDGLRSTNKELKELMLTMGATKKDIFFKLNIPSALPHFYSGLKVAV

TFSIIGAAIGEWLGAQAGLGYFSRRMMTQFDAAGVFAPIVILSALGILFFIIVVGFEKRS

LKWRKTE

>NM1_A2_01472 PLP-dependent transferase

MKIETLLVRSGVGRDPSTGSITTPIYQASTFAHPALGQSTGFDYARTANPTRTALEEAIA

ALEKGEVGVAFASGMAGVMSVLALFKNGDHLIVSEDLYGGTYRVLNEIFSEQGITVSYVN

TAHLEQVKDALRPNTKALFIETPTNPMMHVTDLPEAIALAKQHDLLTIVDNTFMSPYYQR

PLELGADIVIHSASKYIGGHNDVVAGLVVARCSSLGEKIRFYQNAAGAILGPQDSWLLLR

GIKTLALRMEKHNENALKIANWLTAHDLVEKVYYPGLETHPGYEIMKKQATGFGGMISFA

VSHPDIVSVLLENVKVITFAESLGGVESLMTFPARQTHADIPEEIRNRVGVTNCLLRLSV

GIEHADDLIKDLKEAFDAYQQ

>NM1_A2_01583 methionine gamma-lyase

MNEKKKYQFETKAIHAGYESKHHFDSLAPPIYQTSTFTFSSLEQGANRFSGEENGYVYSR

LSNPTVTILEERMAQLEEGEAALAFGSGMAAVSAVLIGLTKAGDHILCSKGVYGCTFGLL

EMLEEKYQIHHSFSNLETEEEILAAIKEDTACIYIETPINPTMTLVDLELVTCIAKQKGI

PVVVDNTFSTPYLQQPLKLGCDLVIHSATKFIGGHGDVVAGIVVGDKEVISVLRKTTQKD

IGGILSPFDAWLLLRGLKTLAVRMDRHCENAEHIAKQLSLHPKVKAVYYPGDKNSTAYSL

MKKQMKKGGGLLSFEVEGGYKETVKVVNQLKLISIAVSLGDAETLIQHPASMTHAVVPEE

ARKEMGISNELLRLSVGLEAWEDIMRDLQQALDSI

>NM1_A2_01901 cystathionine gamma-synthase family protein

MTDKQFRNVQDGTKAVWAGEKESLAYNATQVPVVFSVAYNYDDVDEWQEVALGNKPG

YTYNRMSNPTVKAFEEKVRILEEAEESVAFSSGMAAISSTLYTFLKPGDRVVSVKDTYGGTNK

IFTEFLPNIGVDVTLCNTGNHEEIEAEVNKGCKVLYLESPTNPTMKIIDIERIAKAGKSV

GAVVIIDNTFATPINQNPLQLGVDLVIHSATKFLSGHADALGGVVCGSKELMQHVYHYRE

INGATMDPMSAYLILRGMKTLKLRIRQQERSALEIAKFLQKKEAVEAVYHPGLETHPHHH

IAKKQMKGFGGILSFVLKGEMEAIKILLPKLTYANKAGNLGAVETIYGPARTTSHVECTL

EERKALGISEGLVRISVGIEDTEDLIADLEQAFAHLESASPISN

>NM1_A2_02120 sulfate ABC transporter substrate-binding protein

MKKKAVQFLMVLTALLVVLAGCGNGKSTETAGKDSSASKNDSKKPVELLNVSYDPTRELY

QEFNKSFASYWKDKTGQDVTIQQSHGGSGKQGRAVIDGLEADVVTLALAYDIDEIAQTRQ

LLNKDWEKELAHNSTPYTSTIVFLVRKGNPKGIKDWDDLTKKGTSVITPNPKTSGGARWN

YLAAWAYAKDKYNGDNKKIEDFMSKLYGNVEVLDSGARGATTTFVERGIGDVLIAWENEA

YLSLNELGKDKFEIVTPSLSILAEPPVAVVDKVAKKKGTTKVAKAYLKYLYTEKGQEIAA

ENYYRPRNKKVLEKHKDQFPSLNLVTIKDFGGWKKAQETHFNDGGTFDQIYQPK

>NM1_A2_02829 LLM class flavin-dependent oxidoreductase

MKFALFSLIQNIPNPVTGETLTAQEKFQHVLNQAVLAEKLGFDAYGVGERHGAPFLSSSP

PVVLSAIAAKTSHIRLLTTVTVLSILDPVRVAEDYATLDHLSGGRLELIIGKGNDPRHYP

LFGITEEEQWESLAERYTLLKQLWTEENVTWSGRYRPPLTNVTTQPRPFQPSIPVWHGSA

SSPLSTELAAKYGEPLFSSNSFHPQAKYKALIDHYRERFAYYGHDPSKAIVGSGASSLYI

SDTTEEAIRRYRPYYNAFSNTEAAKHNQSPFTSLEDIVQHGPALVGSPEQIIEKIIDYHH

AYGNEVLSISVDGLSEAEQREQLERFASDIAPVLRKEIPGSVWENEKRGQGQDITK

>NM1_A2_02975 FMNH2-dependent alkanesulfonate monooxygenase

MNILWFFPTAGDGHYLGTTEGSRTSDIHYLKQIAHGLDYLGYDGALLPTGSNCEDSWVIA

SALASVTNRLKFLIALRPGVMSPTLSARMASTFDQISDGRLMLNIVTGGDPVEQATYGNY

LSHDKRYELTDEFLTIWRDVMEGKKVDYKGEHLDVTGAYIPSPPVQKPYPPLYFGGSSPA

GKEVGAKHADVYLLWGEPPAVIKTKIDEMKEKAANEGRDIRFGIRLHVIVRESEDEAWES

ADKLIKHVNDDTIKAFQDKFASFDSTAQKTQSSLHSGTKDRGALEIAPNLWAGIGLAREG

AGTALVGSPEIVADRLKEYKELGIDTFILSGYPHLEEAYTFAELVFPHIQKERSKR

>NM1_A2_03187 assimilatory sulfite reductase (NADPH) hemoprotein subunit

MVNKILKAPEGPPSDVERIKDESNYLRGTLGETMLDRISSGISEDDNRLMKFHGSYLQDD

RDLRNERQKQKLEPAYQFMLRVRTPGGVSTPEQWLVMDDLAQKYGNGTLKLTTRQAFQMH

GILKWNMKKTIQEIHASLLDTIAACGDVNRNVMCNPNPYQSEVHAEVFEWSKKLSDYLLP

RTRAYHELWLDEEKVISTPEVEEEVEPMYGPLYLPRKFKIGVAVPPSNDIDVYSQDLGFI

AILEDEKLVGFNVAIGGGMGMTHGDKATYPQLAKVIGFCRPDQILEVAEKIITIQRDYGN

RSVRKNARFKYTVDRLGLETVKEELENRLGWSLDEAKSYHFDHNGDRYGWEKGVKGKWHF

TLFVQGGRIADFEDYKLMTGLREIAKVHSGDFRLTANQNLIIANVSTQKKKQISDLIEQY

GLTDGKHYSALRRSSLACVSLPTCGLAMAEAERYLPVLLEKIEAIVDENGLRDKEITIRM

TGCPNGCARPALGEIAFIGKAPGKYNMYLGAAFDGSRLSKMYRENISEEEILNELRVLLP

RYAKEREEGEHFGDFVIRAGVIEAVTDGTNFHA

>NM1_A2_03188 assimilatory sulfite reductase (NADPH) flavoprotein subunit

MLQLKVVNSPFNQEQADLLNRLLPTLTEAQKMWLSGYLTASQSTSAEGTPDVSTAAPAQA

EQTISKDVTILYGSQTGNAQGLAENTGKTLEAKGFNVTVSSMNDFKPNTLKKLENLLIVV

STHGEGEPPDNALSFHEFLHGRRAPKLENFRFSVLSLGDSSYEFFCQTGKEFDVRLAELG

GERLYPRVDCDLDFEEPANKWLKGVIDGLSEAKGHSASAAVPAEAPAGTSPYSRTNPFKA

EVLENLNLNGRGSNKETRHLELSLEGSGLTYEPGDSLGIYPENDPELVDLLLNEFKWDAS

ESVTVNKEGETRPLREALTSNFEITVLTKPLLKQAAELTGNDKLKALVENREELKAYTQG

RDVIDLVRDFGPWNVSAQEFVAILRKMPARLYSIASSLSANPDEVHLTIGAVRYEAHGRE

RKGVCSVLCSERLQPGDTIPVYLQSNKNFKLPQNQETPIIMVGPGTGVAPFRSFMQEREE

TGAKGKSWMFFGDQHFVTDFLYQTEWQKWLKDGVLTKMDVAFSRDTEEKVYVQNR

MLEHSKELFQWLEEGAFFYVCGDKTNMARDVHNTLVEIVETEGKMSREEAEAYLAEM

KKQKRYQRDVY

>NM1_A2_03316 PepSY domain-containing protein

MQEKREESASTTKKSTARSQGLYKAIWRWHFYAGLMFTPFLLILAVTGGMYLFKPQIEEK

LYHDFYHVQAQSQSVSPSAQVQAVKEKYPDADVLTYKPSDRSTRSSEVGISLKDHTYTMF

VNPHNGHIIGKVDDSSRLMNQIEEFHGELMAGTAGDRIVELAACWAIVLIVTGAFLWWPR

KKDKIKGVLIPRFSKGKNVLIRDLHAVPAFWISAGMLFLVLTGLPWSGLWGNAFQQVATN

AGVGYPPSIWVGSAPTSTVQTKDVADVPWGAETLEVPSSTASQYTKVSLDDIVGIAKEQH

MHDGYTISIPQDAQGVYTLSVFSPRAQDEATIHLDQYTGAVLADYRYDNYGFMGKLIALG

ITLHKGTQFGFINQLMGLIICIGIAGIAISGSLLWWKRKPAKNMGAPKVPEGSAMRIVTC

IIVVFGILFPLVGLSLVIVWLLDFFVIKRIPALKRFLNA

>NM1_A2_03397 sulfurtransferase TusA family protein

MESNKVLDAKGLACPMPIVKTKKAMNELEPGQVLEIHATDKGAKNDLTAWAKSGGHELLQ

TEEGDVLKFWIKKG

>NM1_A2_03399 sulfurtransferase TusA family protein

MMQANFILDAKGLACPMPIVKTKKKMNELEAGQVLEIQATDKGSTADLQAWAKSTGHEYL

GTEAAGDVLHHFLRKGGAEENVTPIPEISLEEFAKKVENDKHLHILDVREVEEYDKAHIP

GVVHIPLGEVEKRFNELNKEDEIYIICHSGRRSEMAGQTMKKQGFKHLINVVPGMRDWTG

KVE

>NM1_A2_03400 rhodanese-like domain-containing protein

MKTIAAQEVEQLVQNQKQIHILDVREVEEVKTGKIPNALNIPLPLLEFRMHELDKAKNYIVVCR

SGGRSGMAARFLEQQGYSVTNMTGGMMEWKGETV

>NM1_A2_03401 rhodanese-like domain-containing protein

MIINALIIVFLLWFLYQRFATVKGIQQIATTELKAKLKNKDNQFIDVRTPHEFRTKHIKG

FRNIPLSELPAQTGQLSKDREVVVICQSGMRSMKASKLLKKQGFIAITNVKGGMNTWR

>NM1_A2_03403 sulfurtransferase TusA family protein

MKIDQVLDAKGLACPMPIVKTKKAMDTLTTGQVLEVQTTDKGAKSDLTAWAKSTGHELID

FKEEGSTFIFYIQKS

>NM1_A2_04509 rhodanese-like domain-containing protein

MSPITAVLILLGVIIIYSVVMFFIQRRIMKTLTEEEFRSGYRKAQLVDIREPNEFENGHI

LGARNIPLSQFRMRIKEFRPDQPVYIYCQSGMRTGRAAQMLRRHGVQEIYDLKGGFKMWN

GKVKKKK

>NM1_A2_04622 bifunctional cystathionine gamma-lyase/homocysteine desulfhydrase

MKRKTQLIHGGIVGDEQTGAVSVPIYQVSTYKQEGAGKHTGYEYSRTGNPTRHALEELIK

EIEGGYAGFAFGSGMAATTAVFMLFNSGDHVLITDDVYGGTYRVITKVLSRIGIEATFID

TSDIQNIEKEIRPNTKAIFIETPTNPLLKITDLQHASSVAKQHHLLTIVDNTFSTPYWQN

PIEKGADIVLHSATKYLGGHSDVVAGLAVVNSPKLAEDLHFIQNATGGILGPQDSWLLMR

GIKTLGIRMEEHEFNTGKIVEFLLAHPAVAKVYYPGLKTHPNHLIAKKQARGFGGMVSFD

VGSEEKAEEVLSKVKYFTLAESLGAIESLISIPSKMTHASIPSDRRKELGITDGLIRISVGLED

VEDLIEDLERALE

>NM1_A2_04744 FMNH2-dependent alkanesulfonate monooxygenase

MKMLWFIPSHGDGRYLGTNRGGRSADYSYFRQVAQAADRLGFEGVLIPTGKSCEDPWLLA

ASLIPETERLKFLVAVRPGIMAPSVAARMTSTLDRISNGRLLVNVVAGGDPAELAGDGLF

LSHDERYEAADEFLDVWKGLLAGDTVNYEGKHIHVENSELLYPPVQKPSPPIYFGGSSPA

GQKVAAKHSDVYLTWGEPPEQVREKITAVRKQAEAEGRTVKFGIRLHIIVRETEEEAWEA

ADRLIQHLDDDTIQAAQRTFARYDSVGQQRMASLHRGTREELTISPNLWAGVGLVRGGAG

TALVGSAENIAKRIKEYEALGIESFILSGYPHLEEAYHVGELLFPLLNIDEGEGKSESIG

EIIANDRFPKASTS

>NM1_A2_04745 NADPH-dependent FMN reductase

MSQFTIITGSPSKQSRSAALSEYIAAYLQNEKHEVKTVHVRDLPAEDLLYANFSSPAIQE

AQKKVADADAVIIVSPIYKASYTGILKTFLDLIPEKGLHNKTVLPIATGGTIAHLLALDY

VFKPTLSVLGATALIHGVYIVDSEVAYTKENEIEFIAEEAEARIKRSLKELEYHLEQTKR

SVES

>NM1_A2_04746 ABC transporter permease

MSKTSVVSGHVQSSAAHLQQVKKRKEEAKNKQGIFLRSLVVPILLIVVWQIIGSAGYVSK

TVLPTPVDIVLSFKQLIVSGELGANLQISILRAAIGFAVGAGLGLLFGIIVGFSKKTEHY

MDPSIQMLRTVPHLALAPLFILWFGFGEISKILLIANGAFFPIYVNTYLGIRGVDSKLFD

VARVLQFSKWKQITKLILPAALPNILLGVRLSLGIAWLGLVVAELMGASEGVGYMIMDAR

QFSQTDVVFVGIIIFAVVGKLTDSFVRVLEKRLLKWRDSYAGEK

>NM1_A2_04747 aliphatic sulfonate ABC transporter substrate-binding protein

MKKLLLTAAIVLVLALAGCGTKEKASGEDSKNVTINIGIQQSLGPLMLAQNQKWFEKEFK

KIGVNVKWTEFQSGPPQFEGLASGHLDFGQVGNSPVISGQGADIPFLEIANSSDGLKGNA

ILVGKNSKIKSVKDLKGKKIAVAKGSSGFNLLYRALDQNGLKPSDVKIIQLQPDEAQPAF

ENGSVDAWSIWEPFISLQNLKNEARILADGDSLKVASPGFTIVREGFAKDHPELVVKFLQ

VYQKALEWQNEHFEESVDILAKQKNLDKDVVRQVLKNNPAYNRPTSKEIIAEQQRTADFQ

QSLGVIKKKIDTGDVVDNSFIEKALKEK

>NM1_A2_04817 bifunctional oligoribonuclease/PAP phosphatase NrnA

MKAQILEAIKQFDTIIIHRHIRPDPDAYGSQCGLAELLKTSFPEKKVYVTGEEAESLKFL

YRMDDIADDTYEDALVIVCDTANEARVSDQRYASGKMIIKIDHHPNQTPYGDLLWVETSA

SSTSEMIYEFYLEGKEKGLALNDEGAKLIFTGIVGDTGRFLFPNTKPKTFCYASELIQYG

FNFKDVYDQLYKTKENVVHLHGYVLQNFTMSPAGVAHMKIPNEILRKYDVTPADASQLVG

SLGHIDGVKTWVFFVEEENQIRVRLRSKELVINTIARKYNGGGHPLASGASIDSWEEVEP

LLLDLEELCSQK

>NM1_A2_04876 cysteine synthase A

MKVVNNMADLIGETPLVKLNRLQPADGASVYLKLEFFNPSRSVKDRAAFNMIVEAEKAGL

LNENSTIIEPTSGNTGIGLAMNAAARGYRSILVMPDTMTQERINLLKAYGAEVVLTPGDE

KMPGAIRKAEELTKEIPNAFMPMQFENNANPDAHRKTTAKEIIEAMNDLGKDLSAFVATA

GTGGTITGTGEVLRENYPNMTVHVVEPAGSPVLSGGRPGKHKLVGTSPGFIPDTLNIEVY

DEILKIKDEQAYDITRRLASEEGILVGPSSGAACYAAIEVAKKLSPDQVVVCIACDTGER

YLSSDLFSYE

>NM1_A2_04883 rhodanese-like domain-containing protein

MEEVKVITPEELQKRVENGEELHLVDVREDEEVAEGMIPTAKHIRMNDIPTNVDYFDKEK

EYIFICRSGRRSENVCYYLQEQGYKVTNMVGGMLEWEGKTVNK

>NM1_A2_04894 gamma carbonic anhydrase family protein

MIYPYKEKMPNIAPSCFLADYVTITGDVTIGEESSIWFNTVIRGDVSPTIIGKRVNIQDQ

STLHQSPNAPLLIEDDVTVGHQVILHSSIIRKRALIGMGSIILDGAEIGEGAFIGAGSLV

PPGKKIPPNTLALGRPAKVIRTLTEEDLKDMQRIRTEYVEKGQYYKSIKKSDSSL

>NM1_A2_04905 ABC transporter permease

MKPTSSLDTLHESYLRKVRGEKRLVLSVQMVIFLAFFSFWELASRLKWIDPLIFSSPTKV

WHLFLIKLADGSLAEHIGFTLFETVLGFILGTLLGILLATALWYSTRLANILDPYLVILN

AMPKVALGPILIVAIGPGFFSILTMGAIISVIITSIVVYTAFKEVDPNYIKLLKSFGATK

TRCFKEAILPASMPAIISTFKVNVGLSWVGVIVGEFLVSSKGLGYMIIYGFQVFNFTLVL

LSLLLIAIFATIMYQGVAYLEKKLIRRS

>NM1_A2_04966 adenylyl-sulfate kinase

MSKSTNITWHDAGITKQERREQNNHHSFVLWFTGLSGSGKSTVANAVAKALFDKNIRNYV

LDGDNVRFGLNKNLGFSAEDRTENIRRIGEVSKLFVDSGQVVLTAFISPFQEDRAQVREI

LEGNEFLEVYVECPLEECEKRDPKGLYKKARSGEIRDFTGIDSPYESPANPEVTINTSTQ

SVEECVQTVIEYLSNRKFI

>NM1_A2_04967 sulfate adenylyltransferase

MTTIQAHGGELINLYQPTYDYTSLTKEIEVDNMALSDLELIGIGAYSPITGFLGEKDYQS

VVENMRLADGTVWSIPITLPVTEEQAKELNIGDKVKLVQSGVTYGVLEVSEVYTPNKEKE

AENVYRTAELAHPGVKKMMERPNVYVAGPIVLVERTPKTRFEKYYLDPTETRAAFEERGW

KTVVGFQTRNPVHRAHEYIQKTALEIVDGLFLNPLVGETKSDDIPADIRMESYEVLLENY

YPSDRVALAVFPAAMRYAGPREAIFHAMVRKNFGCTHFIVGRDHAGVGDYYGTYDAQKIF

SNFTADELGITPLFFEHSFYCKKCEAMASTKTCPHSKEDHMILSGTKVREMLRNGEVPPS

TFSRKEVIEVLIKGLAKQKVSSK

>NM1_A2_04969 phosphoadenylyl-sulfate reductase

MADLYTYENWSDTFPEFDSKDETKGALSALQWAYDTYGDSIIYASSFGIEGIVLIDLISK

VKKDAEIVFLDTGVHFKETYEVIDAIKERFPDLRIHMKKPDLTLAEQAEKHGDELWKSQP

NLCCQIRKIIPLRESLAPYDAWISGLRREQSESRANTNYFNKDEKFKKVKVCPLIHWSWK

EIWRYVYKHDLPYNKLHDQGYPSIGCEPCTAPAYNVDDLRSGRWAGTQKVECGLHES

>NM1_A2_05232 (Fe-S)-binding protein

MTGLLWVNFLFFIIVTAYAISLFVYLIRTRIEYIQLGKKVEFDQRLKERLQKIWVNVFGQ

KKLLKDKKSGIMHVMFFYGFILVQFGALDFIIKGLLPGKHLPLGPLYPGFTFFQEIVTFL

ILVAVVWAFHRRYVEKLVRLKRGFKSGLVLIFIGALMLSVLVGNGMSIIWHNHEPAWTEP

IASSIAILFKGINDTAAISIFYVSWWIHLAVLLTFLVYVPQSKHAHLLAGPANVFFNRVS

KPGKLEKIDFEDETQETFGVGKIEEFTQHQLIDLYACVECGRCTNMCPATGTGKILSPMD

LILKMRDHLTEKGAVVTSKAPWVPTYAFSKTKGNQLAMMAASQGGAEAAATAEYNPALIG

DVITEEEIWACTTCRNCEDQCPVMNEHVDKIIDLRRFLVLTEGKMDADAQRAMTNIERQG

NPWGLNRKEREEWRHVREDVHIPTVKEVSKAGETFEYLFWVGSMGSYDNRSQKIALSFAK

LLNEAGVSFAILGNKEKNSGDTPRRLGNEFLFQELASKNIAEFEKNEIKKIVTIDPHAYN

IFKNEYPDFGFEGEVYHHTELLAMLVKEGKLTPKHAVNEKITFHDSCYLGRYNEVYSPPR

EILKAIPGVSLIEMDRNRENGMCCGAGGGLMWTEETTGQRINVARTEQALAVNPSIISSG

CPYCLTMLSDGTKAKEVEEKVHTYDVAELLEKSIYGDVQEQVS

>NM1_A2_05315 DHH family phosphoesterase

MPVFTKKNVIRYPFYSLYSIALILVGIVTYHNWIIGMIGFILLLACLFLYMRMERMLSDE

FETYISMLSHRLKKVGEEALMEMPIGIMLFNDEYQIEWTNPFLASCLGEDTLVGRSLYDV

AESIIPLIKQEVETEVVTLHDRKFKVVIKRDERLLYFFDITEQIEIEKLYEEERTSLGII

FLDNYDELTQGMDDQVKSNLNSQVTSMLNSWAQEYGIFIKRTSSEKFIAIMNEQILIHLE

RSKFSILDQVREETSKQNIPLTLSIGIGAGAADLPELGALAQSSLDLALGRGGDQVAIKQ

PNGKVKFFGGKTNPMEKRTRVRARVISHALKELITESGKVIIMGHKYPDMDAVGAAIGIL

KVAQVNQKDGFIVLDPDHIDTGVQRMLEEIKKKEGLWERFITPEEALNLVSDDTLLVVVD

THKPSLVIEERLLNRIENVVVIDHHRRGEDFIEDPLLVYMEPYASSTAELVTELLEYQPK

RFKIDMLEATALLAGIIVDTKSFTLRTGSRTFDAASFLRSQGADTVLVQKFLKEDITQYV

QRARFIEHAEIYTAGIAISRAEPNKMYDQVLIAQAADTLLSISGVVASFVISKRRDNLIG

ISARSLGDINVQVIMESLQGGGHLTNAATQLQDISLDEAEERLKQAIDEYLDGGKKS

NR1 amino acid sequencing

>NR1_00078 cysteine synthase A

MRVAQSVSELIGKTPIVKLNRIVESDSADIYLKLEFMNPGSSVKDRIALAMIEDAEKKGL

LKEGDTIIEPTSGNTGIGLAMVAAAKGYNAILVMPETMSIERRNLLRAYGAELVLTPGPE

GMGGAIRKATELAKEHGYFIPQQFKNQSNPEIHRLTTGPEIIEQMGDQLDAFIAGIGTGG

TITGAGEVLKKAYKDIKIYAVEPADSPVLSGGKPGPHKIQGIGAGFVPETLDVEVYDEII

QVKTEQAFEYARRVAKEEGILVGISSGAVIYAATEVAKKLGKGKKVLVIIPSNGERYLST

PLYQFES

>NR1_00114 serine O-acetyltransferase

MFKRLREDIEVVFEQDPAARGYFEVILTYSGLHAVWAHRIAHAFYKKNFFFLARFVSQVS

RFFTGIEIHPGATIGRRFFIDHGMGVVIGETCEIGDNVTIYQGVTLGGTGKEKGKRHPTI

QDNVLIATGAKVLGSITVGENSKIGAGSVVLKEVPAHSTVVGIPGRVVIQNGVKIGQELN

HSDLPDPIFDKLKAMEVELDKLKKQLELKVERKDKNDYSHL

>NR1_00200 DoxX family protein

MVINFLRTDKRATFILLLLRLYIGYTWLAAGIGKVFGQSFDASGFLKGAIAQASGDHPA

VQSWWADFLQHFVLPNADLFSFLVQWGEILVGLGLILGGLTKTAAFFGIIMNLSFLLSGTV

SVNPNLLILTMFILVAGQNAGRIGLDGYVFPKLFRKNNHGTYKLSKTA

>NR1_00449 ABC transporter permease

MNMQNQKQSKLITTIWLILLIAIWEGSVSLFKIEPWILPKPSAIVQELIGMKDLLLPNTM

QTLQEVIIGLFFAILLGTSIAIIMDVIPLFRILINPLLVISQTIPIVVLAPLFIIWFGYGMLPK

VMVVILVCFFPIALSILEGFQTVDKNMLKLLQTMKATKWQVYQKVKFPAVLPYFFS

GLKIAVTYSVMGAIIGEWLGASEGLGVMLTRATKSFLTARVFGVAAIIVMVTLCLYFIVE

FMARITAPWIYRKDGRK

>NR1_00775 ABC transporter permease

MNRLKELVPAITLSSILLAVWEIGARIVDEMYILPSPSAILMKIWKLKDILFTVHLPATL

YVVLIGVVISIVLGVGLAMLMNASTWMERAFYPLLVASQTIPITALAPLFVLWFGYTIWS

KVVVTVLITFFPIAVNTYDGLRSTKKEWEELLVTYGATKRDIFLKLKLPSALPYFFSALK

IAVPLSVIGAAIGEWLGAQAGLGYFSKRMMTQLDGAGVFAPIVLLSLLAIFFVVIISILE

KKFISWRKHS

>NR1_00820 sulfurtransferase TusA family protein

MSIKVDMSLDCKGLACPMPIVKTKKAIEGLAPGQVIEVKATDKGSTVDIKSWASKVGHQY

IGTKHEGDVLVHYVRKANEHEVNEVVKYPHTITNAELQSILSHGEESIVLDVREAAEFAF

GHIPSAISIPLGELDSAVLDETKQIYVICRTGNRSDVACQMLKEKGFSNVKNVIPGMLGW

QGNVEK

>NR1_00822 sulfurtransferase TusA family protein

MMNIKQVLDAKGLACPMPIVRTKRAMDTLQTGEVLEIHVTDKGSVKDIPAWANKCGHDIV

KLEEEGDVLKFWIRKA

>NR1_00823 rhodanese-like domain-containing protein

MNTILSTLFIVLAAWFVISRFLPVKGVQNINGKELKSIVGKKGKYFIDVRTVGEYRGNHM

KGFQNIPLNDLASKANQLDKNKEVIVICQSGMRSKQAAKVLKKLGFQQVINVSGGMNSL

>NR1_00824 rhodanese-like domain-containing protein

MKEMTTKELEEKLLRKEVVNIVDVREVEEVAEGKIPEACNIPLGLLEFRMHELNKNQEYI

IVCRSGGRSARAVQFLESYGFQVINVVGGMLAWEGKVE

>NR1_01119 sulfate ABC transporter substrate-binding protein

MKKWGKTAFMKSTGVLLSAVVLLSGCGSATSTSNSEGSGDKKTVELLNVSYDPTRELYQD

FNKDFAKYWKEKHGQTVNVKQSHGGSGSQARSVIDGLEADVVTLALAYDIDAISKKKQLA

EDWQKRLANNSTPYTSTIVFLVRKGNPKGIKDWDDLTKKGVSVITPNPKTSGGARWNYLA

AWGYALKKYNNSEDKAKEFVSQIYKNVEVLDSGARGATTTFVEKGIGDVLIAWENEALLS

QKELGKDKFEIVTPSISVLAEPPVAVVDKVVDKKGTKKVAEGYLEYLYSEKGQEIAAKNF

YRPRNEKVAEKYTSQFPKVQLFTVDELFGGWKKAQEKHFNDGGVFDKIYQK

>NR1_01445 phosphoadenylyl-sulfate reductase

MLTYETWEENVVSFSEEDETKGALSVLNWAYKEYKDEVVYACSFGVEGMVLLHIINQVNP

SAKVVFLDTNVHFQETYELIRKVRERFPSLNIIEKQPELTLDEQAKLHGEKLWESNPNLC

CKIRKILPLEKSLVVEKAWISGLRREQSETRKHTKFINQDHRFQSIKVCPLIHWTWKEVW

RYVYKHSLPYNPLHDVGYPSIGCEKCTLPVGDGGDSRDGRWAGKVKTECGLHYQ

>NR1_01446 sulfate adenylyltransferase

MSIVNELVNRIDETYDVSQIEKEIKLDNIALSDLELLATGGYSPLTGFLGKEDYDSVVET

LRLANGSVWSIPITLPVTEKVAESLKAGEEVKLVNNGNIYGVIQIEDIFVPDKEKEALLV

YKTTDEAHPGVKKLYERPNVYVGGTIILTKRFENNQFPSYHLDPIETREAFKKRGWKTVV

GFQTRNPVHRAHEYIQKSALEIVDGLFLNPLVGETKSDDIPADVRMESYEVLLQNYYPKN

RVFLSVFPAAMRYAGPREAIFHALVRKNFGCTHFIVGRDHAGVGDYYGTYEAQEIFTNFT

IEELGITPLFFEHSFYCTKCEAMASTKTCPHGKEDHVILSGTKVRELLRNGEIPPSTFSR

KEVVEVLIKGLKTEVVTE

>NR1_01447 adenylyl-sulfate kinase

MDTNITWHTASVSKDERRVKNGHHSFVIWFTGLSASGKSTVANAVARKLFEKNIGNYVLD

GDNIRHGLNKDLGFSESDRMENIRRIGEVAKLFVDQGAVVLTAFISPFRVDRKQVRDLLA

ADEFIEIFVKCPIEECEKRDPKGLYKKARKGDIKDFTGIDSPYEEPEQAELIVETHKYSI

EECAEQIVKYLQERSFI

>NR1_01448 ferredoxin--nitrite reductase

MSYEKVWANNEKLNQTEKNKLEKDGLEIFNDIPYYAENGFESIPKEEWDAFKWAGLYLQR

PKEAGYFMMRVNIPSGIITNAQAEVLASIAEDYGRNVIDITTRQAIQFHWLEIQQIPDIF

KRLARVGLSSAGACGDITRNITGNPLAGIDANELFDTTHIVKEVYDYFQHNEEFSNLPRK

FKMSISSNIYNSANAEINCVAFTPATKEIDGEKKVGFHIKVGGGLSARPYLADELDVFVL

PEEVKAVAIAIATIFRDFGYREKRHLARLKFLVADWGAEKFKEKLIEYTGPLQSKGESAL

KGWNAGYFYGVQDQKQKGLKYVGFNVPVGRLHAEEMFEIARIAKQYGNGQIRTCNSQNFI

IPNVPPENVKGLLSEPLFEAISASPKSFIGHAVSCTGIEYCNLALVETKERLRKIAEYLD

TQIALDVPVRIHMVGCPNSCGQRQIADIGLQGVKMKTKEKGIVEAFEIYVGGTLLDGGAY

NQKLKGKIDGEDLPDVLASFISYFKENKLPAETFYDFVGRVGVDTLQIALNNVLEEVIAS

>NR1_01603 sulfurtransferase

MIVTVEWLREHIEDENVRIIDCRFDLANPNWGREKYEEGHIPHALYFDLNLDLSSPIAEH

GGRHPLPNIEEFADKLSEAGIDEHTKVIAYDSQAGANAARLWWLCNYVGHEKVYILDGGF

PAWKENGLPTTTEIPVVIRKTFKTNIQDHMLVTMETVRENIRAGADVTLIDSREPKRYAG

VEELVDHKAGHIPTAVNHFWKDGMLQSGQFKNGAGQQERFQNLSKDKETIVYCGSGVTAC

PNIVALKLAGFQNVKLYAGSWSDWISYPENQIAKEED

>NR1_01799 cysteine synthase A

MKLCENVTELIGDTPVVRLSKFIPEDAADVYVKLEMFNPSRSVKDRAAYNLLHVAEENGL

IKPGDTIIEPTSGNTGIGLAMNAAAKGYKAILIMPDNMSKERINLLKAYGAEVVLTPAEQ

RMPGAIAKALELQKQIPNSFIPQQFENPANPNIHRYTTALEIYEQMDGELDAFVATAGTG

GTITGTGETLKEKLPNLYIAVVEPKGSPVLSGGVPGPHKLVGTSPGFIPKNLNTEVYNEI

IQIADEEALTTMRNLARQEGLLVGPSSGASVYAAIMIAKRLGVGKKVLCIAPDTGERYLS

MGLFE

>NR1_01903 LLM class flavin-dependent oxidoreductase

MEKYRIDTRKGIEFGLYSIGDHVLNPHNGDKITPEKRIHELIETAKLADEAGLDVFAVGE

SHQTHFTTQAHTVILGAVAQATKNIKIASSATILSTSDPVRVYEDFATIDLISNGRAEIV

AGRGSRIGGYSLLGYDVNDYEELFEEKMDLLLKINNEEHVTWNGQFRAPLAHASVIPRAK

NNNLPIWRAVGGPPASAIKAGRAGVPMMITTLGGPAINFKVSVDAYREAAQQSGFDPASL

PVATTSLFYTAKNSQDALSEYYPHINAGMLTLRGDGYPKQQFTNAIDYRDALMVGSPQQI

IEKMLYQYELFGQQRFMAQIDFGGVPFDKIEKNIELIATEILPAVRKHTAK

>NR1_02812 FMNH2-dependent alkanesulfonate monooxygenase

MELLWFIPAYGDGRYLGTTKRGRAAEYGYYKQVAAAADYLGYTGVLLPTGQGCEDPWVLA

SALAAETEKLKFLVAVRPGLMSPTVAARMASTFDRISDGRLLINVVAGGDPVELQGDGLY

LNHDERYEAADEFLKVWKSTLQGETISLEGKHIQVTDSKVVFPPVQTPYPPIYFGGSSAA

GKEVAAEHSDVYLTWGEPPEQVKEKVEEVRKLAEEKGRTVRFGIRLHVIVRETEEEAWEE

AERLIQYVDNETIELAQKTFARYDSVGQKRMTHLNKGTRESLEISPNLWAGIGLVRGGAG

TALVGDPHTVAERIKEYESLGIDTFVLSGYPHLEEAYEVAELLFPLLKDKKKEENKIVGE

MIADAYALKK

>NR1_02813 ABC transporter permease

MENTKAVMKPASITIEKNRVKNVRKLNVKVLVRAITIPVIILIIWQLAGVFGLVSKTVLP

TPLDIFLAFQELIKTGELFGHLSISVFRAAAGFFIGGGLGIILGTIVGFSTRSEQYLDPS

VQMLRTVPHLAVAPLFVLWFGFGETSKVLLIADGAFFPLYVNAFLGIRGVDSKLFDVARV

LEFSKRKLITKLILPSALPNLLLGARLSLGVAWVSLVVAELMGSTEGIGYMIMDARQFSN

TDIVFVGIIIFAFVGKFSDSLVRLLEAKFLRWRDNFKGETGN

>NR1_02814 aliphatic sulfonate ABC transporter substrate-binding protein

MYKKFKILSFALAISVCLLGCEKSTASSKKEDVTIQIGIQQGLSPLLLAKKKGWFEEEFK

KEGVKVKWTEFQSGPPYFEAIASNRLDFGEVGNSPVISAQAAGIGFTEIANTSYARKGTG

ILVQKDSKIASVKELKGKKIAVAKGSSAFNLLYRALDKEGIDAKEVNVIQLQPDEAQPAF

ESGSVDAWAIWDPFISLHTLNKGAKVIADGETLNVSSPEFLITRTKFAKEHPELVEKFLK

VYEKARVWQDANLDEAIKVYTSVKKIDAEIVKEVFNHDKPILVPVTKEIIAEQQKTADFQ

YKLGSIKKEIKAEKVVDNFFVEKALKAK

>NR1_03411 NAD(P)/FAD-dependent oxidoreductase

MKTRDSYKIIVIGAGTAGLSSTAHLLRNVPLLKESIAIIDPSKKHYFQPLWSLVGGGIVS

KESTMRNQESLIPKGATWIPKSVVELFPSENKILLDDGLLLEYEILIVAAGIQINWDSIK

GLKESIGTNGVCSNYSYTYVDSTWREIEKFKGGNALFTHPNTPIKCGGAPQKIMYLAEEY

FCNSGVRNRSKVIFYSANNNIFQVPRYANTLEQVLERKQIITNYNKNLVEIIAEKREAIF

EDTQTLKRETVPYSMIHVVPPMGPPNFIKESEISDHQGWVDISPYTLQHVQYKNIFGLGD

CTNLPTSKTGAAIRKQIPVSKQNIMDVLSGRDLQAKYDGYTSCPIVTGYKSLILAEFNYE

HEPQEMFPFNQAKERYSMFLLKRYMLPYMYWNLMLKGIL

>NR1_04202 rhodanese-like domain-containing protein

MSTTWIILLAVIVAFIGYTVWMYFYQKKLIKTLTEEEFRAGYRKAQLIDIREADEYNAGH

ILGARNIPLSQIRLRHKELRQDQPVYLYCQSGFRTGRAAQYLKKQGYKDFYQLQGGFKSW

TGKIKKK

>NR1_04251 cystathionine gamma-synthase/O-acetylhomoserine thiolyase

MSTIETKLAQIGNRSETTTGTVNPPVYFSTAYRHEGIGKSTGFDYSRTGNPTRGLLEQAI

ADLEYGEQGYACSSGMAAVLLVLSLFRSGDELIVSEDLYGGTYRLFSEHEKKWNVRCRYV

NTQSIKQIEQAITTETKAIFIETPTNPLMQVTDIAAVATVAKRNGLLLIVDNTFYTPYIQ

QPLTEGADIVLHSATKYLGGHNDVLSGLVVAKGKELCEEIAHYHNASGAVLSPFDSWLLI

RGMKTLALRMRQHEENAKAVVAYLNDEDGVTDVFYPGRGGMISFRLKDEAWINPFLQSLS

LITFAESLGGVESLMTYPATQTHADIPEEIRTANGVCNRLLRFSVGIENSNDLIQDLKQA

IKLVKEGVRI

>NR1_04252 cystathionine beta-lyase

MSYSIDTLLLHNQYKHDSQTGAVNVPIYNTSTFHQFDVDTFGKYDYSRSGNPTREALEDI

IALLEGGTKGFAFASGIAAISTAFLLLSQGDHVLISEDVYGGTYRVITEVLSRYGVSHTF

VDMTNLEEIKQNIKPNTKLFYVETPSNPLLKVTDIREVSKLAKSIGALTFVDNTFLTPLF

QKPLELGADVVLHSATKFIAGHSDVTAGLAVVKDSELAQKLGFLQNAFGAILGPQDCSLV

LRGLKTLHVRLEHSAKNANKIAHYLQEHSKVKNVYYPGLQTHLGFDIQQSQATSAGAVLS

FTLQSEDALRKFLSKVKLPVFAVSLGAVESILSYPAKMSHAALSQEARDKRGISNSLLRL

SVGLENVNDLISDFENALSYVEEPVNA

>NR1_04343 DoxX family protein

MVIQFLRENKAVSFALAVIRVYLGYTWLMAGIGKLQGKGFDATGYLQGAIEKSKGAQPAV

QSWWASFLQEFAIPNVDLFNTLVTWGEILVGIGLIVGCLTKTAVFFGLVMNFSYMFSGSI

GVNPEMVILSMFVLVSGMNAGKFGMDGFVIPKVLGSKTPKRQKQAA

>NR1_04366 bifunctional cystathionine gamma-lyase/homocysteine desulfhydrase

MRAKTKLIHGIRIGEPSTGSVNVPIYQTSTYKQEAVGKHQGYEYSRTGNPTRAALEEMIA

VLENGHAGFAFGSGMAAITATIMLFSKGDHVILTDDVYGGTYRVITKVLNRFGIEHTFVD

TTNLEEVKEAIRPNTKAIYVETPTNPLLKITDIKKISTLAKEKDLLTIIDNTFMTPYWQS

PISLGADIVLHSATKYLGGHSDVVAGLVVVNSPQLAEDLHFVQNSTGGILGPQDSFLLLR

GLKTLGIRMEEHETNSRAIAEFLNNHPKVNKVYYPGLESHQNHELATEQANGFGAIISFD

VDSEETLNKVLEKLQYFTLAESLGAVESLISIPSQMTHASIPADRRKELGITDTLIRISV

GIEDGEDLIEDLAQALA

>NR1_04367 O-acetylserine dependent cystathionine beta-synthase

MNVYRGVHELIGHTPIVEITRFSLPTGVRLFAKLEFYNPGGSVKDRLGRELIEDALEKGL

VTQGGTIIEPTAGNTGIGLALAALEHDLRVIVCVPEKFSIEKQELMKALGATVVHTPTEQ

GMTGAIAKAKELVNEIPNSYSPSQFANEANPRAYFKTLGPELWDALNGEINIFVAGAGTG

GTFMGTASYLKEKNIDIKTVIVEPEGSILNGGKAGSHETEGIGLEFIPPFLKTSYFDEIH

TISDRNAFLRVKELAQKEGLLVGSSSGAAFHASLLEAEKAAPGTNIVTIFPDSSERYLSK

DIYKGWE

>NR1_04617 bifunctional oligoribonuclease/PAP phosphatase NrnA

MHEQILGAIKEFDTIIIHRHVRPDPDALGSQGGLGTILQESFPEKNIYTVGYNEPSLAYL

RVMDDIQDSVYENALVIVCDTANQERVDDQRYTKGKMLIKIDHHPNEDPYGDITWVDTTA

SSTSEMIYEFYNYGKDKGLKITKEAARLILAGIVGDTGRFLFPNTTAKTLRYVSELVDMG

VKFTDLYNEMYKTKEKIARLNGYILQNFTMVEEGAAYIKLTKEVLEEFDVLPSEASGVVG

ALGNIDGLKAWVLFLEEDDVIRVRLRSKGPVINKLAMQYNGGGHPMASGAKASSWEEADR

LFADLREICK

>NR1_04665 methionine gamma-lyase

MKKKHMETALIHHGYKPEEYKGSLTPPLFQTSTFTFETAQQGEASFAGVDPSYIYSRLGN

PTVKLFEERMAVLEGGEEALAFGSGMAAISATLIGFLKAGDHIICSNGLYGCTYGFLEVL

EEKFMITHSFCDMETEADIENKIRPNTKLIFVETPINPTMKLIDLKQVIRVAKRKGLLVI

VDNTFCSPYLQRPLELGCDAVVHSATKYIGGHGDVVAGVTICKTKALAEKIRPMRKDIGG

IMAPFDAWLLLRGLKTLAVRMDRHCDNAEKIVSFLRKHDAVEGVWYPEGELASRQMKRGG

GVISFSVKGGKEETQAFINDLHFITIAVSLGDTETLIQHPATMTHAAIPAELRKEMGIFD

NLIRLSVGLESWEDIVSDLEQALKKISTVNQ

>NR1_04736 rhodanese-like domain-containing protein

MTEVKTITTEEVQERLENGETLFLVDVREDEEVAAGKIPEAVHIKMGDIPHKVDFFNKEN

EYIFICRSGMRSENVCHYLNEQGFKTVNMVGGMLQYEGETK

>NR1_04757 PepSY domain-containing protein

MKVNRSLHYILWRWHFYAGLFITPLLITLSLSGIGYLFREEVEDFIYKDLYFGKSAQTES

ISMSDSISLTEKKYPHYSVAKISEFNGDYNTRLTIANEYTGQQKYVYLDSNNQIVGDQNA

SETFANIMRELHSSLLVGGTVVNYTVELAACWTIFLIVTGLYMSIRQFKNTPSSNKREKA

KRRHSIIGIIFTIPLFLLVASGLPWSGFMGNQIYKIASSNESLGYPKLYMAPPESKVKEL

PWATRKEAPPESNSNEPKAISVDELQKGIEIKKPYVISLPADPKGVFTVSKSSGSGITGM

HVAPNEEITAYFDQYSGELISKTDYRDYGLLAQWFTYGIPLHEGHLFGWPNKILCLLTTL

SLLLLIYYGIKMWLARKPKGKLAAPPKQRDKKSIFVFFIMMVILGAVMPLFGLSVLVIFT

IELLIYVFLKIRS

>NR1_04761 gamma carbonic anhydrase family protein

MIYPYKEKKPKIASSAFIADYVTITGDVYVGEESSIWFNTVIRGDVSPTIIGDRVNVQDQ

CTLHQSPQYPLILEDDVTVGHQVILHSCHIKKDALIGMGSIILDGAEIGEGAFIGAGSLV

SQGKKIPPNTLAFGRPAKVIRELTAEDRKDMDRIRTQYVEKGQYYKSLQK

>NR1_04785 ABC transporter permease

MDNIKQLHEQFRKNERRRAWIARSLQLLLLILFFALWEIASKKEWIDPLLFSSPSSIWDL

FLTKWIDGSLWVHIWTTLLETGVGFILGTVLGAIIATFLWWMPLLARVLDPYLVVLNAMP

KVALGPIIIVIFGPNISSSIAMGVIISIIITILVIYSAFQEVDSNYIKVMDTFGANKWQC

YKQVVLPASFPAIISTLKVNVGLSWVGVIFGELLVSKQGLGYLISYGFQVFNFTLVLLSV

LLTCVLATLMYVFVEAFEKILIGKRKRS

>NR1_05337 (Fe-S)-binding protein

MNSLLIINWLAAIAVIAYAGYLFVYLIRTRMAYIQLGKKIEFDRRFKERWDLLKVNVFGQ

KKLLKDKKSGIIHVMFFYGFILVQFGAIDFVWKGLAPGSHLPLGPLYPAFTFFQEIVTLV

ILIAVFWAFHRRYVEKLVRLKRNFKSGLVLIFIGGLMISVLLGNGMGLIWHGEELSWSEP

IASAIAYVFSGINETVAISVFYFSWWVHLLILLTFLVYVPQSKHAHLIAGPANVFFGRLS

NPGKLEKIDFEDETQETFGVGKIEDFRQNQLIDLYACVECGRCTNMCPATGTGKMLSPMD

LILKLRDHLTDKGAAVTSKAPWVPVVAFNNTQGNQLAMMAAGKGQQESASTTLAYDPSLI

GDVITEEEIWACTTCRNCEDQCPVMNEHVDKIIDLRRYLVLTEGKMDAEAQRAMTNIERQ

GNPWGLNRKERETWRQGDDEVTVPTVKEKSKAGEEFEYLFWVGSMGSYDNRSQKIAISFA

KLMNEAGISFAILGNKEKNSGDTPRRLGNEFVFQEMATKNIEEFEKAGVKKIVTIDPHAY

NTFKNEYPDFGLQAEVYHHTELLAQWVKEGRLKPVHAIEETVTYHDSCYLGRYNEVYEAP

RDILKAIPGVNLVEMARNRETGMCCGAGGGLMWMEETTGSRINVARTEQALAVQPSIIGT

GCPYCLTMISDGTKAKEVEEKVQTLDVTEILERSVIGQKKEAM

Section 4
NM1-A2 protein structure

NM1_A2_04876_cysteine_synthase_A 1

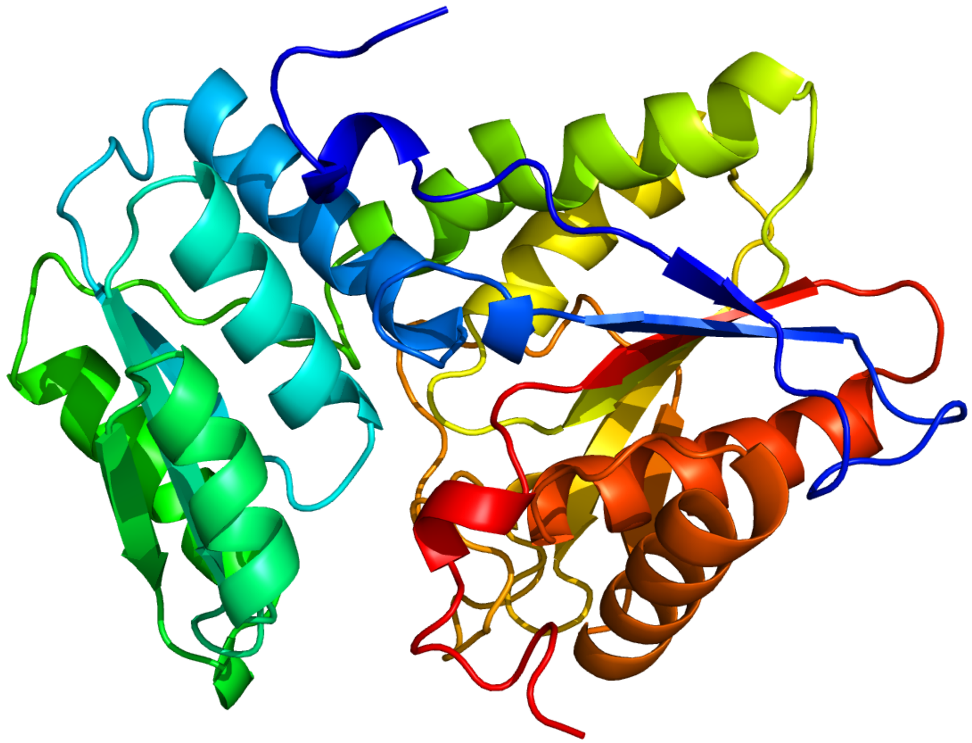

NM1_A2_00094_cysteine_synthase_A 2

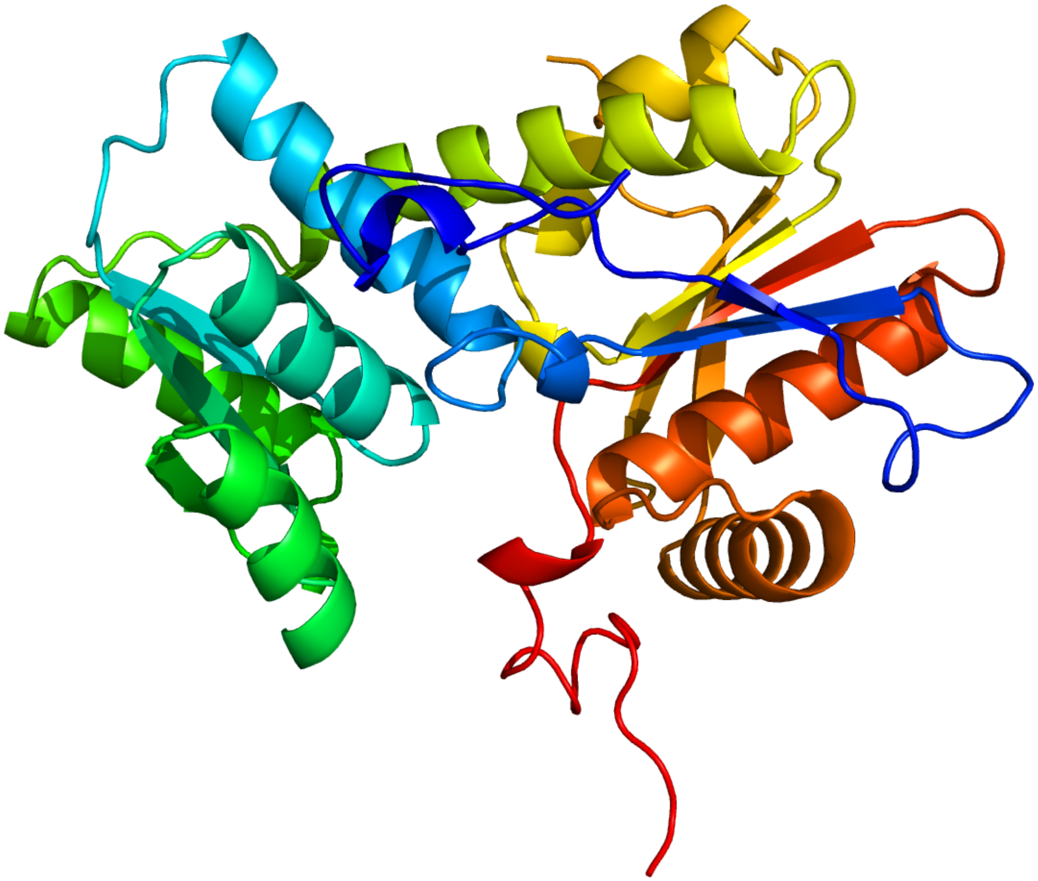

NM1_A2_00115_serine_O-acetyltransferase

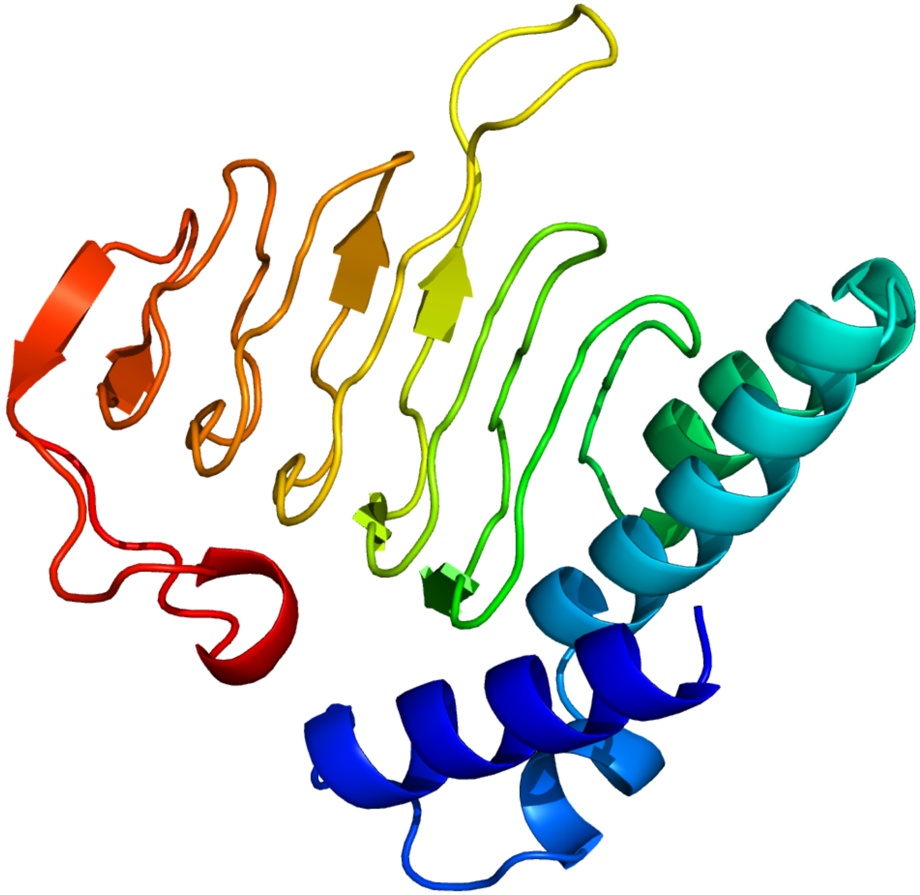

NM1_A2_00911_FMNH2-dependent_alkanesulfonate_monooxygenase

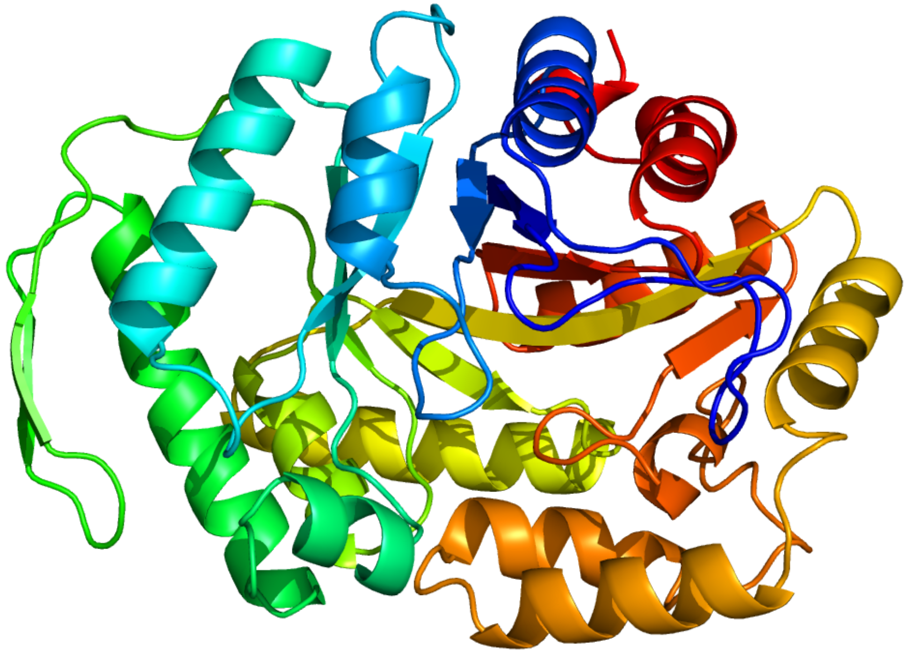

NM1_A2_00928_ABC_transporter_permease 1

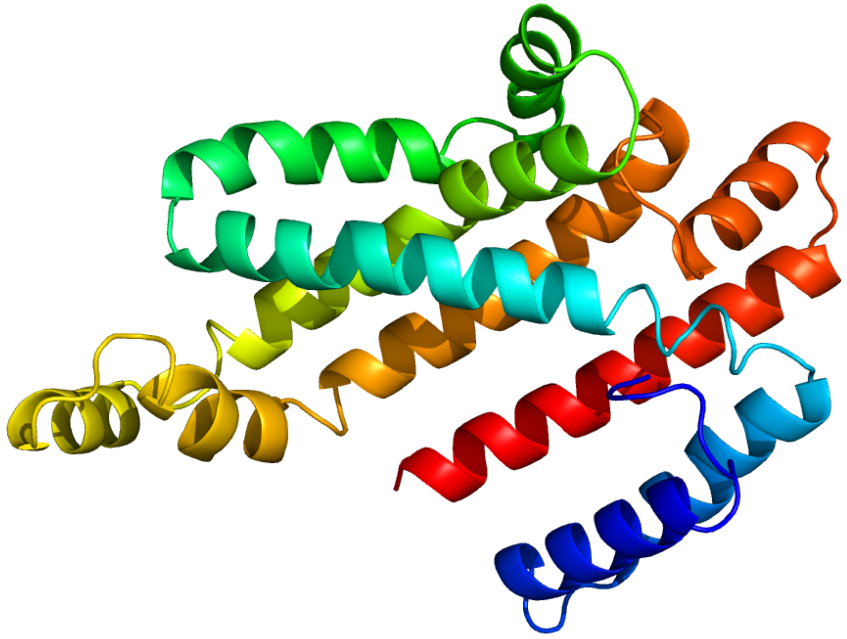

NM1_A2_04746_ABC_transporter_permease 2

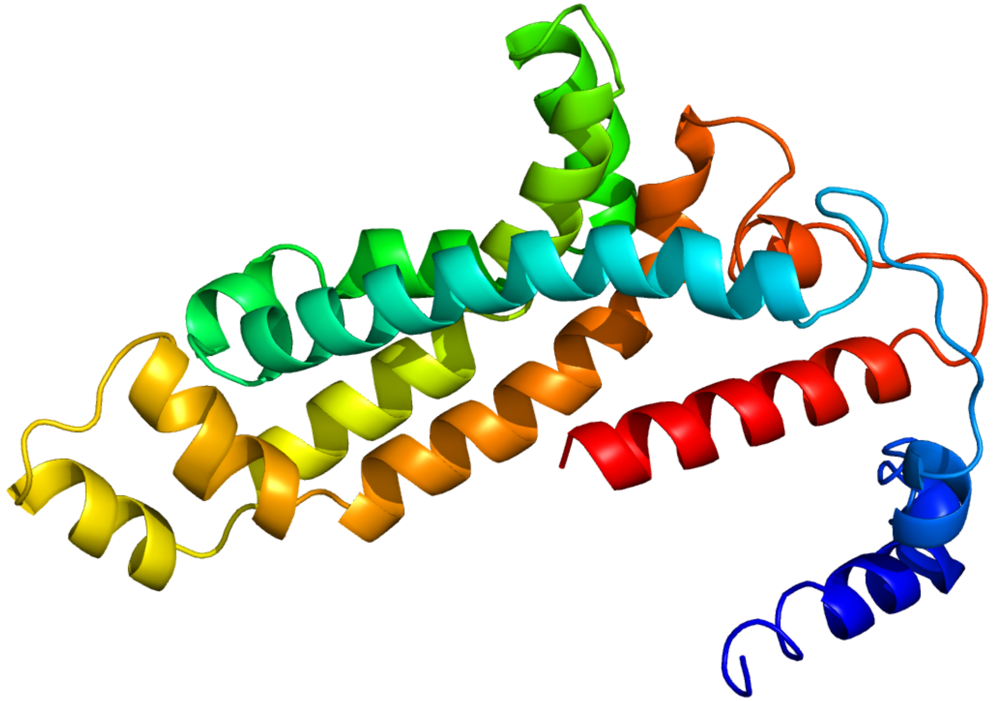

NM1_A2_04905_ABC_transporter_permease 3

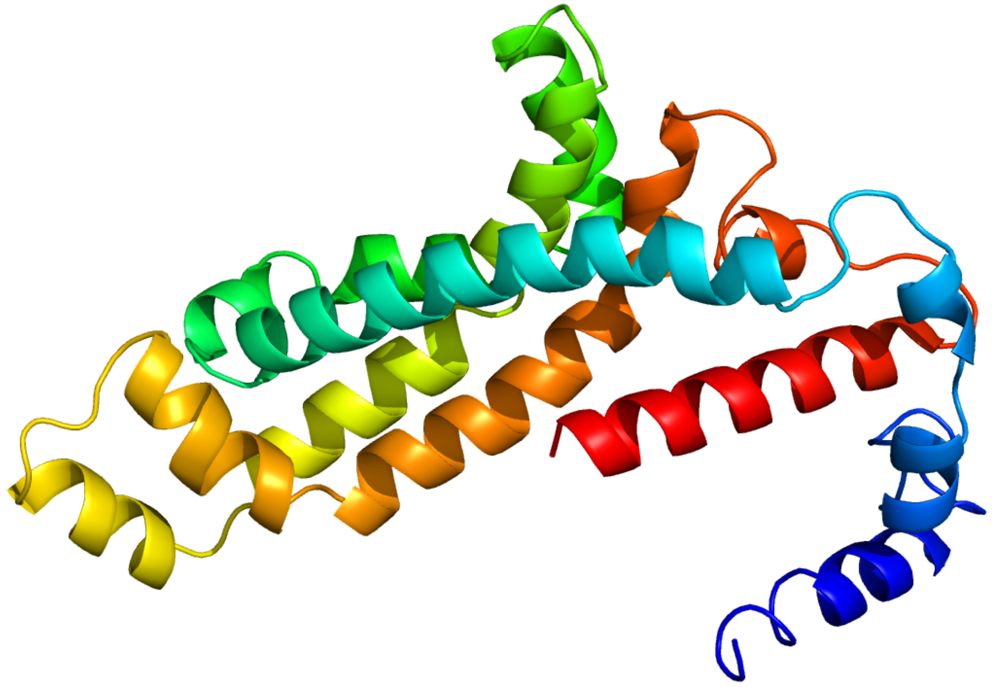

NM1_A2_01583_methionine_gamma-lyase

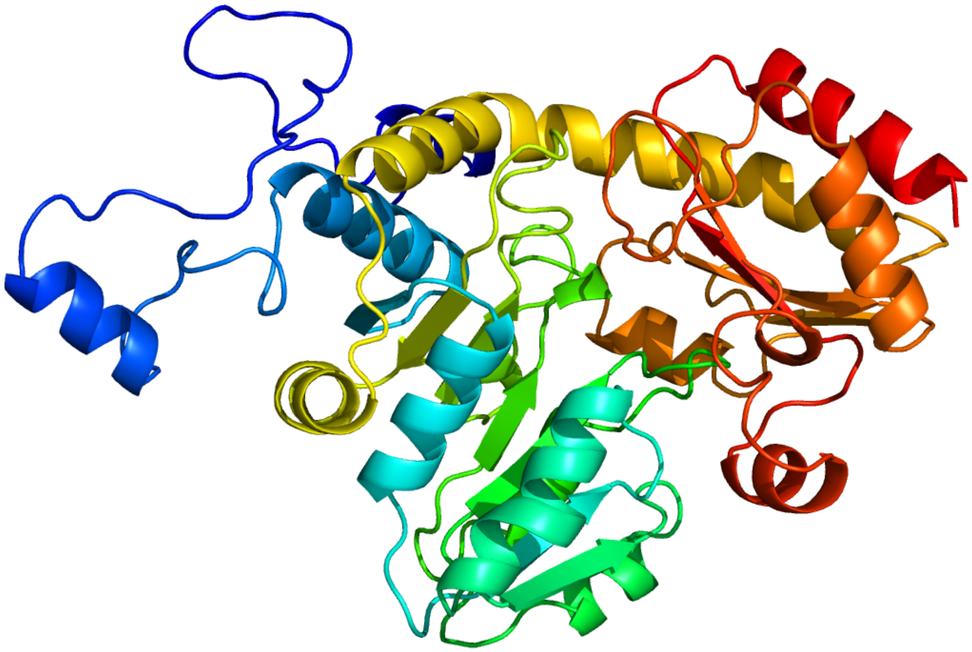

NM1_A2_02120_sulfate_ABC_transporter_substrate-binding_protein

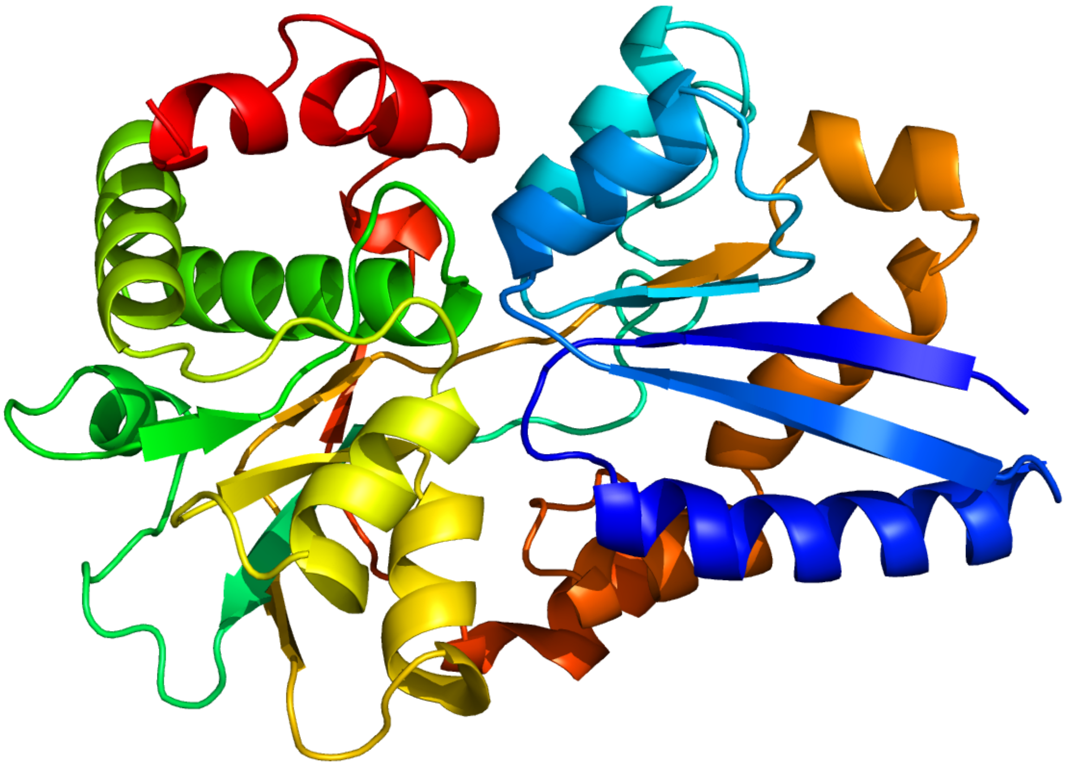

NM1_A2_02829_LLM_class_flavin-dependent_oxidoreductase

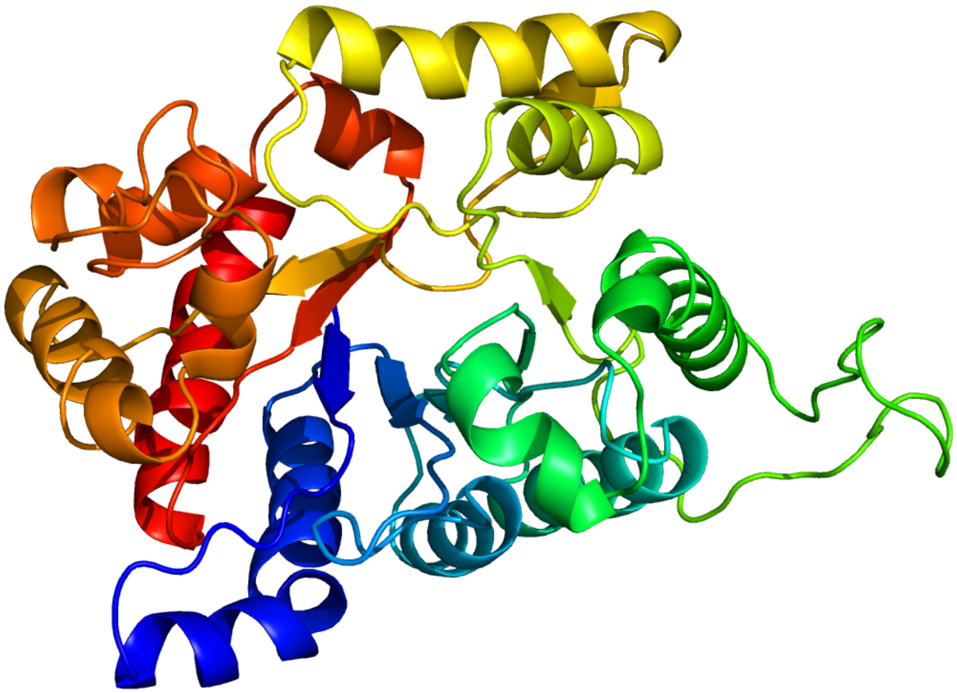

NM1_A2_03316_PepSY_domain-containing_protein

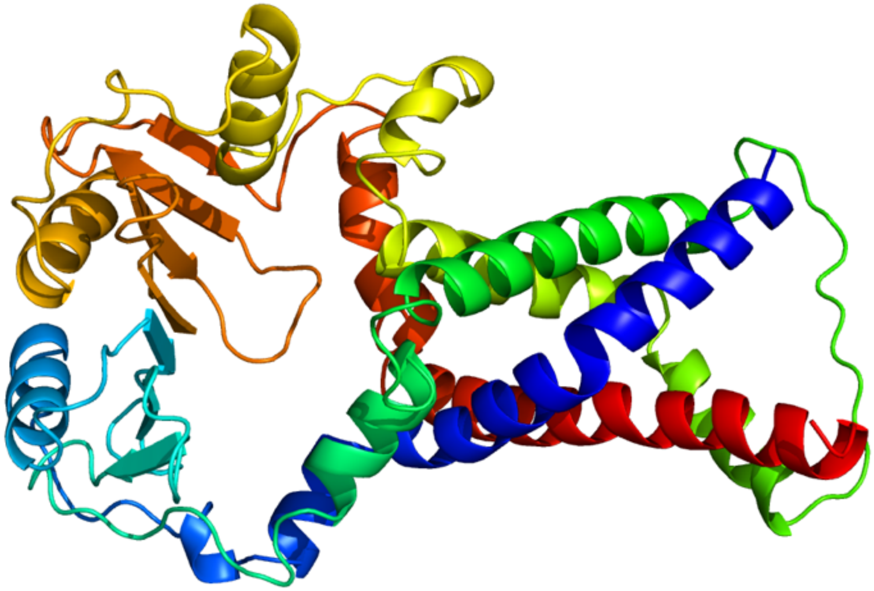

NM1_A2_03397_sulfurtransferase_TusA_family_protein 1

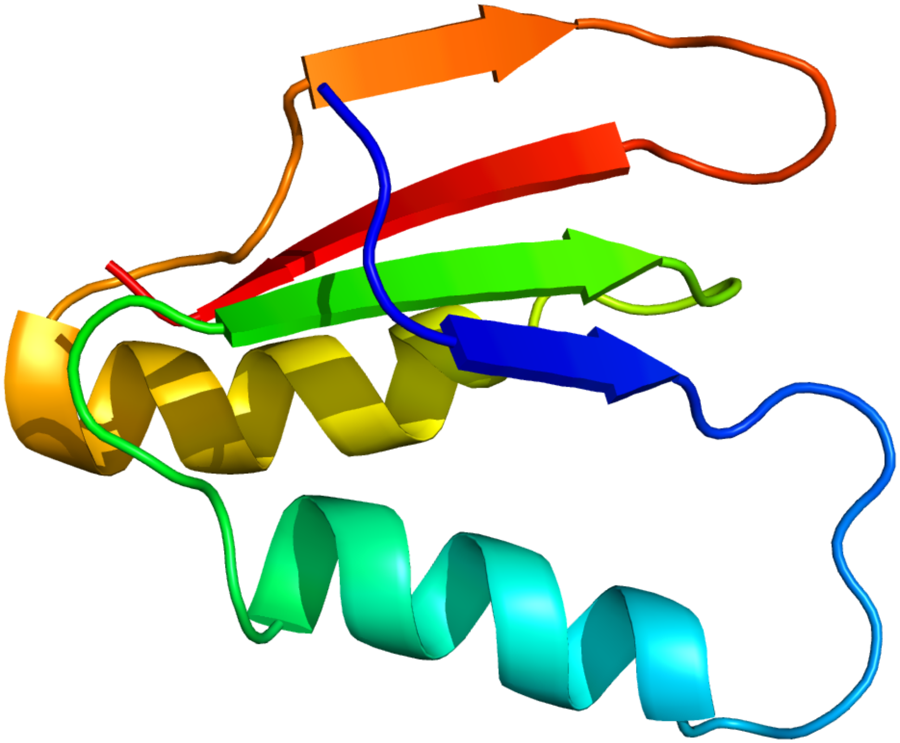

NM1_A2_03399_sulfurtransferase_TusA_family_protein 2

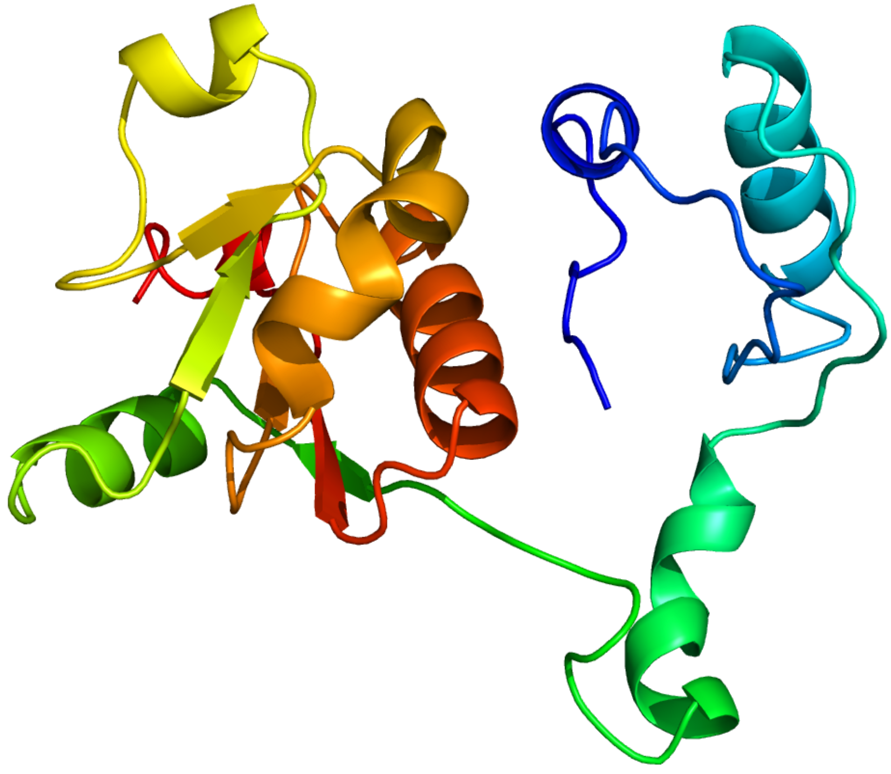

NM1_A2_04509_rhodanese- like_domain-containing_protein 1

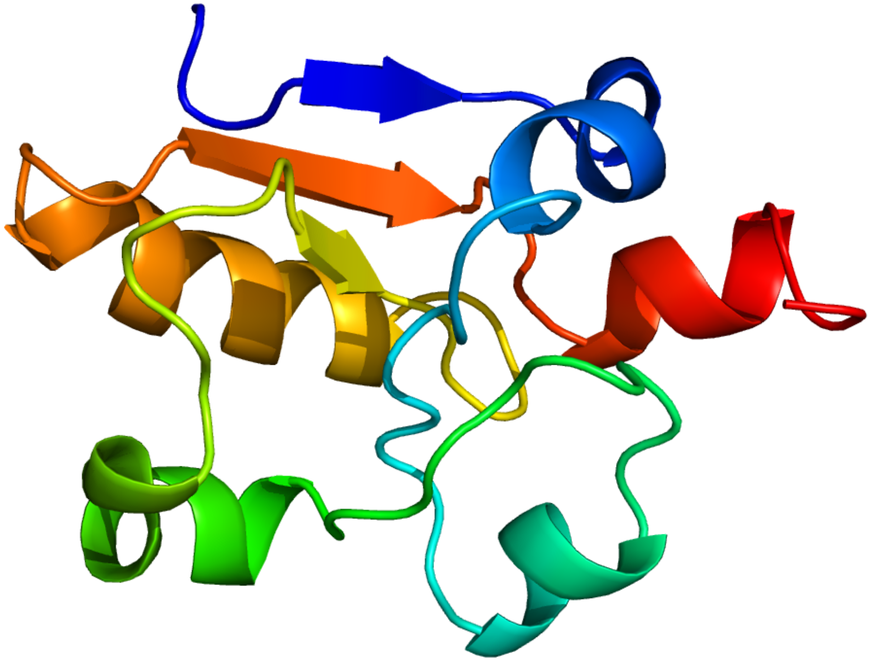

NM1_A2_03401_rhodanese-like_domain-containing_protein 2

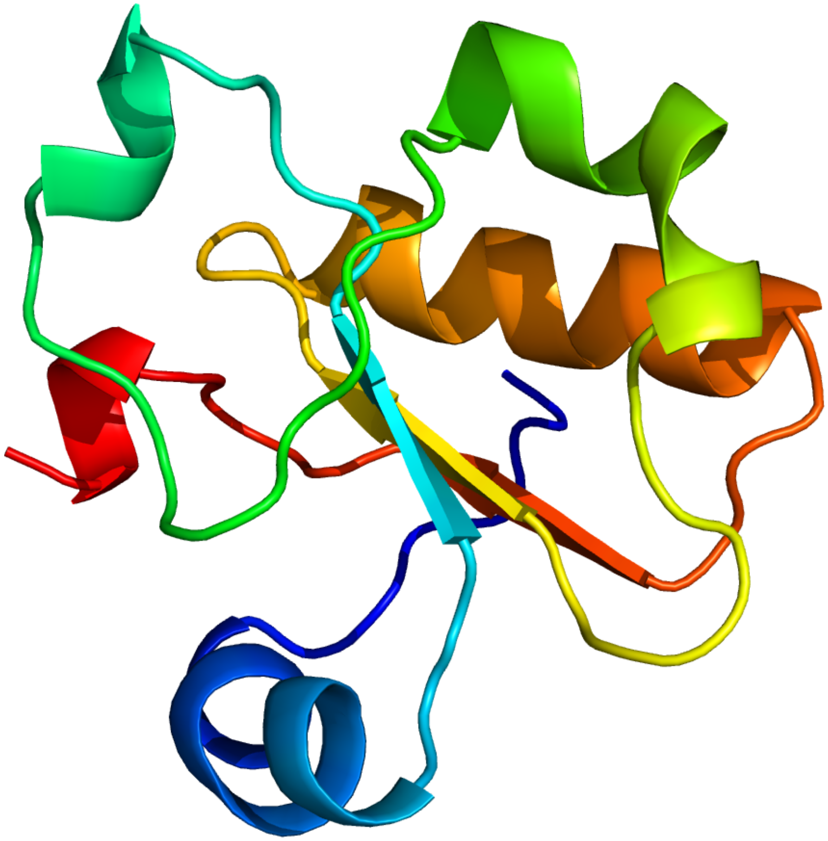

NM1_A2_04509_rhodanese-like_domain-containing_protein 3

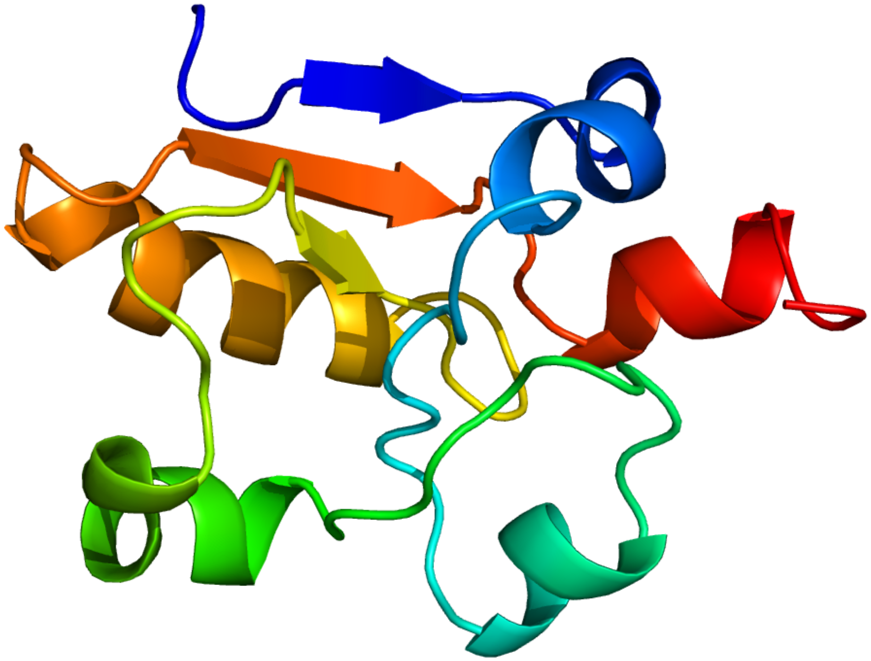

NM1_A2_04883_rhodanese-like_domain-containing_protein 4

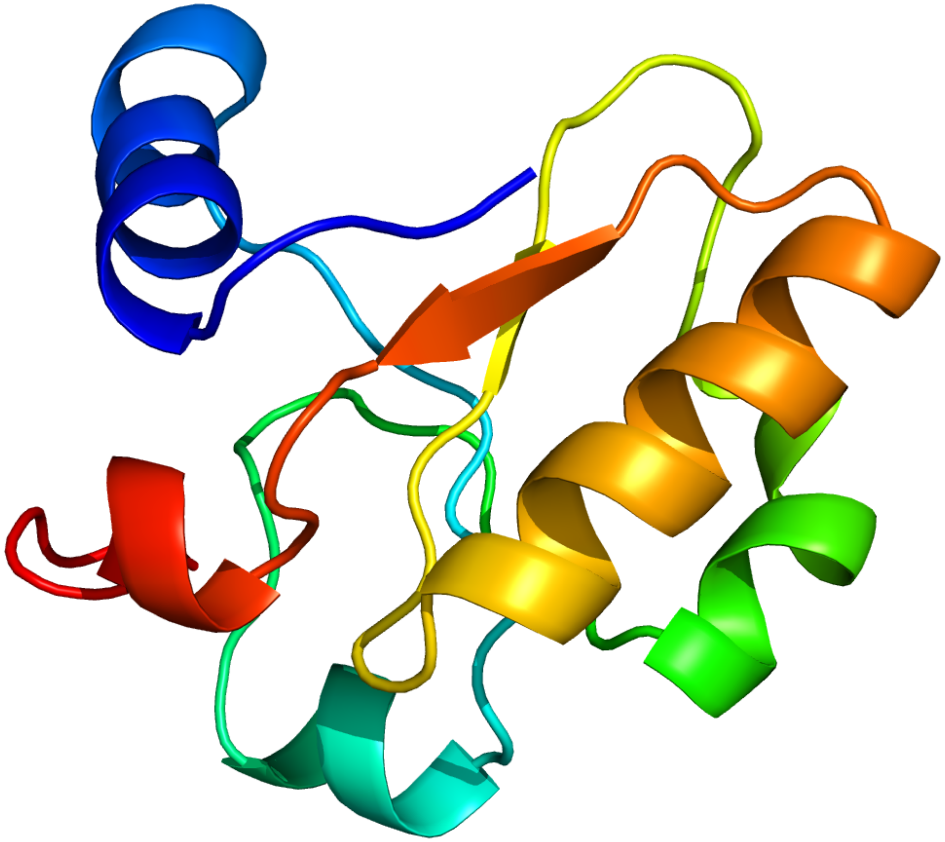

NM1_A2_04622_bifunctional_cystathionine_gamma-lyase_homocysteine_desulfhydrase

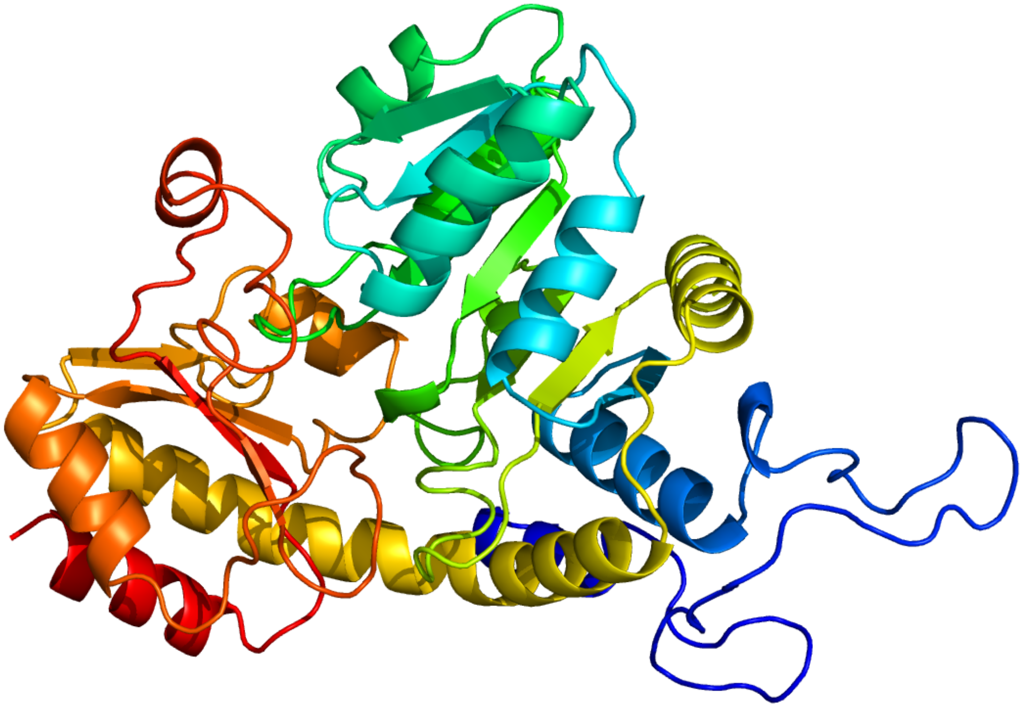

NM1_A2_04747_aliphatic_sulfonate_ABC_transporter_substrate-binding_protein

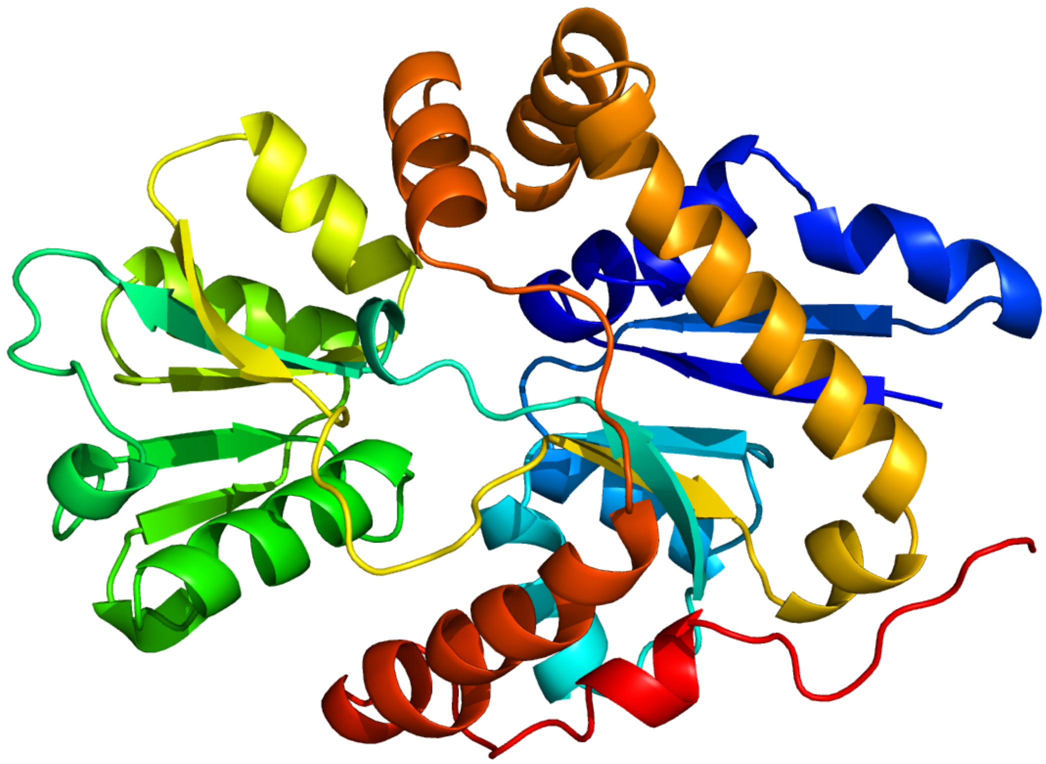

NM1_A2_04817_bifunctional_oligoribonuclease_PAP_phosphatase_NrnA

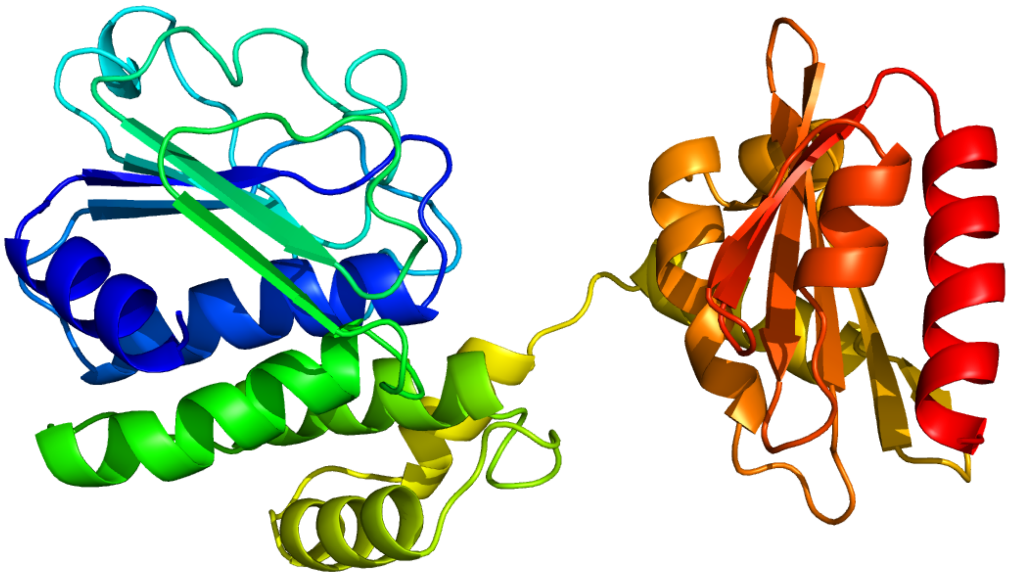

NM1_A2_04967_sulfate_adenylyltransferase

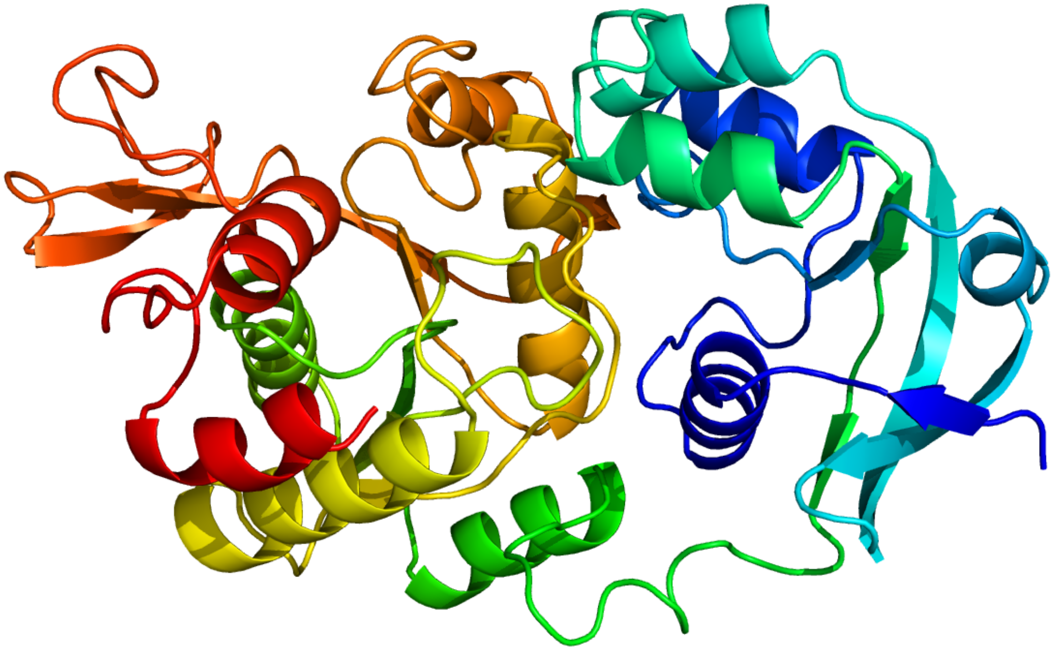

NM1_A2_04969_phosphoadenylyl-sulfate_reductase

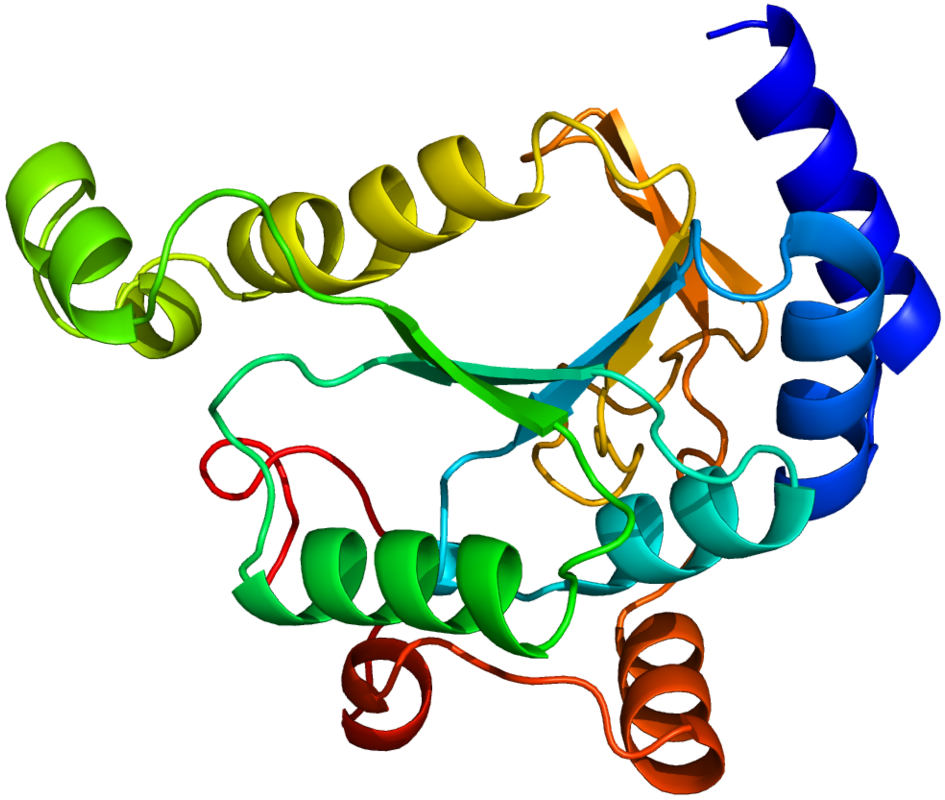

NM1_A2_05232_Fe-S_-binding_protein

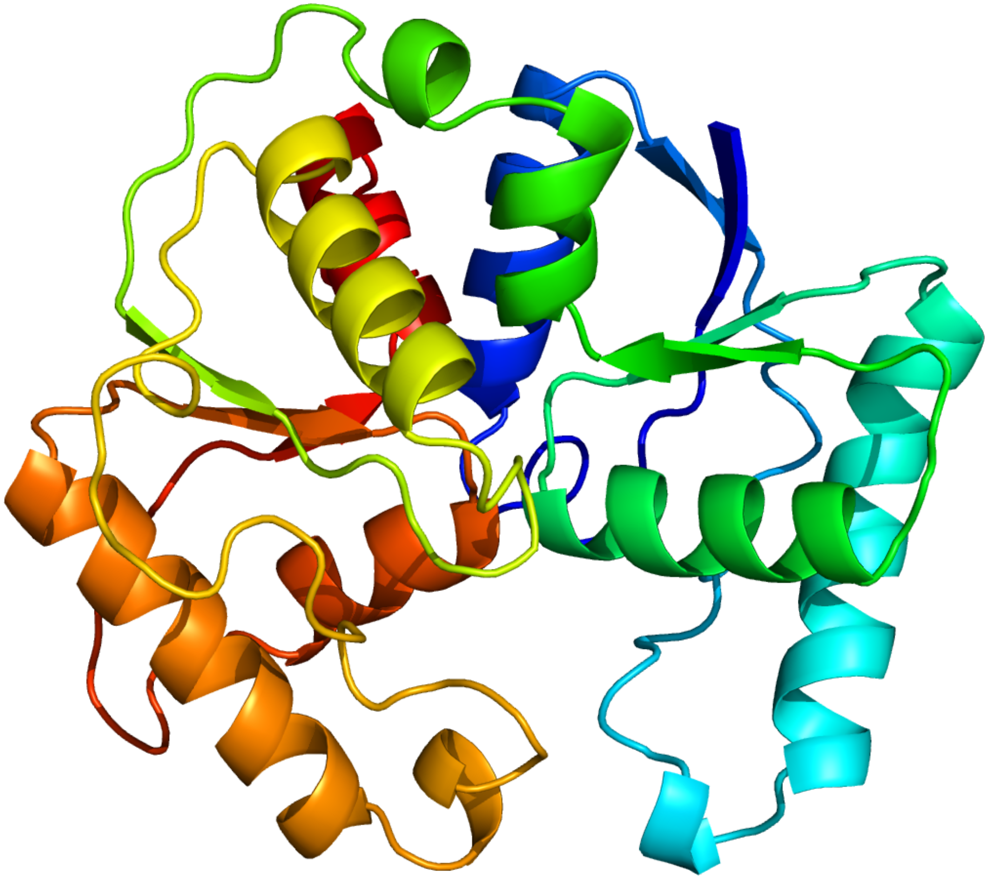

NM1_A2_04894_gamma_carbonic_anhydrase_family_protein

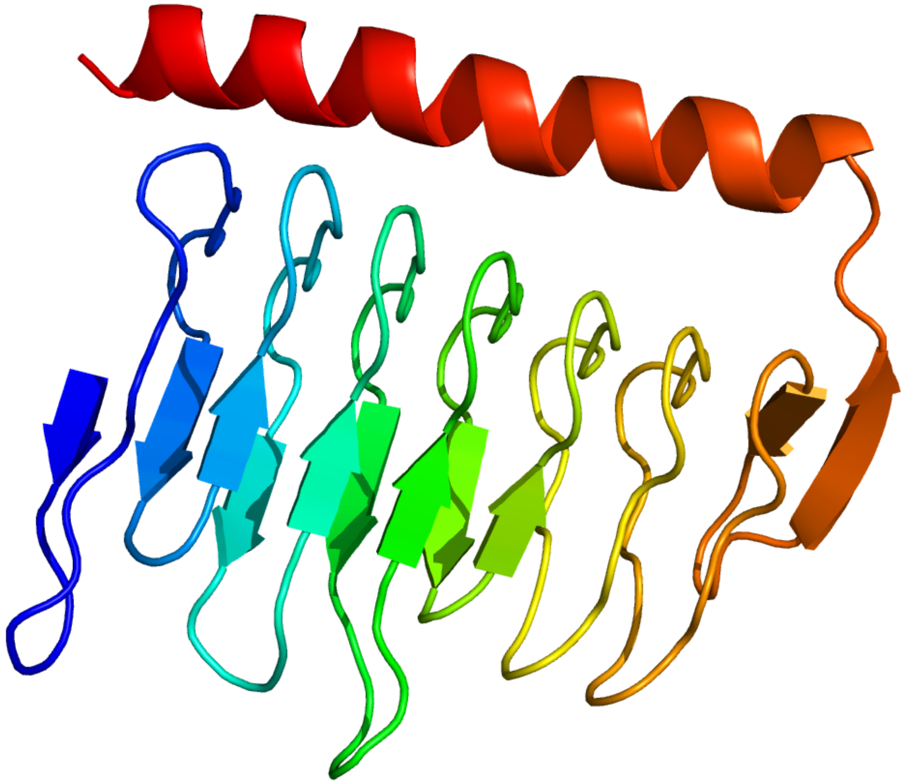

NM1_A2_04966_adenylyl-sulfate_kinase

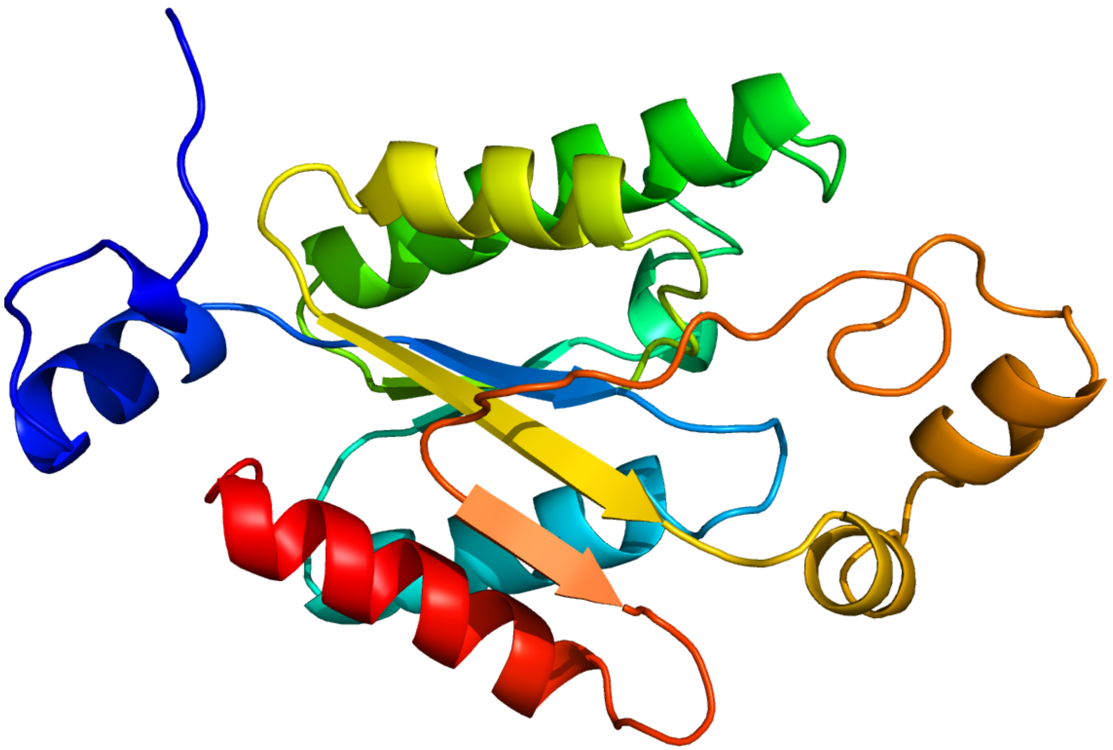

NR1 protein structure

NR1-cysteine synthase A.1

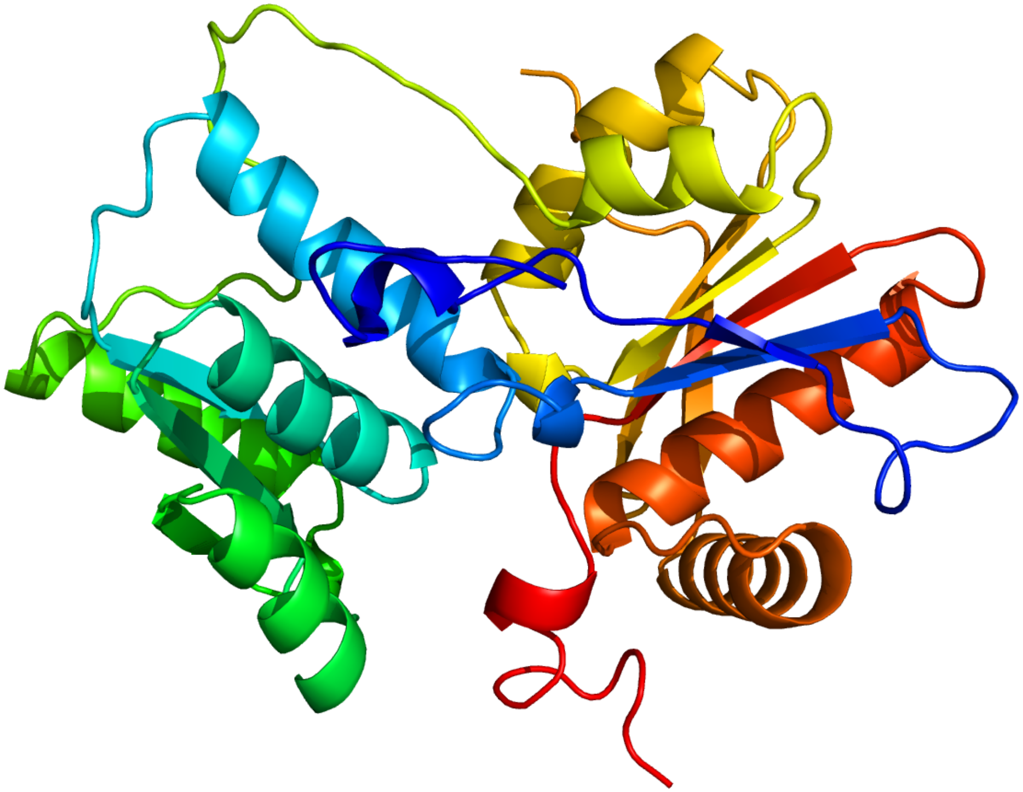

NR1_01799_cysteine_synthase_A2

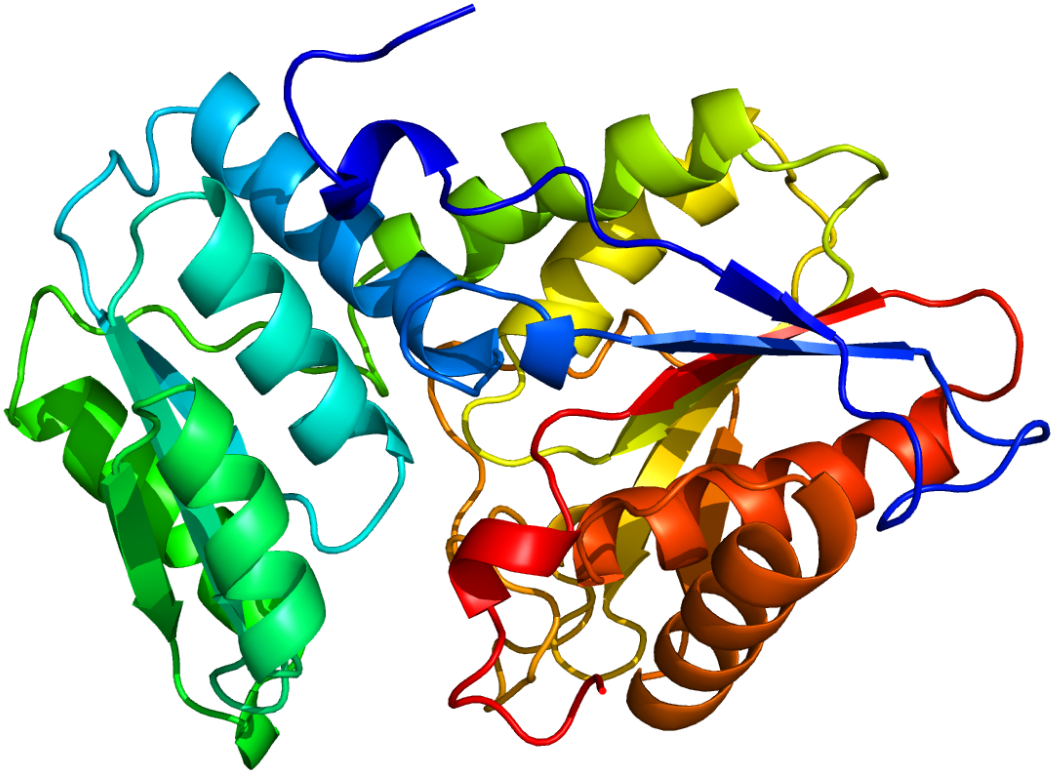

NR1_00114_serine_O-acetyltransferase

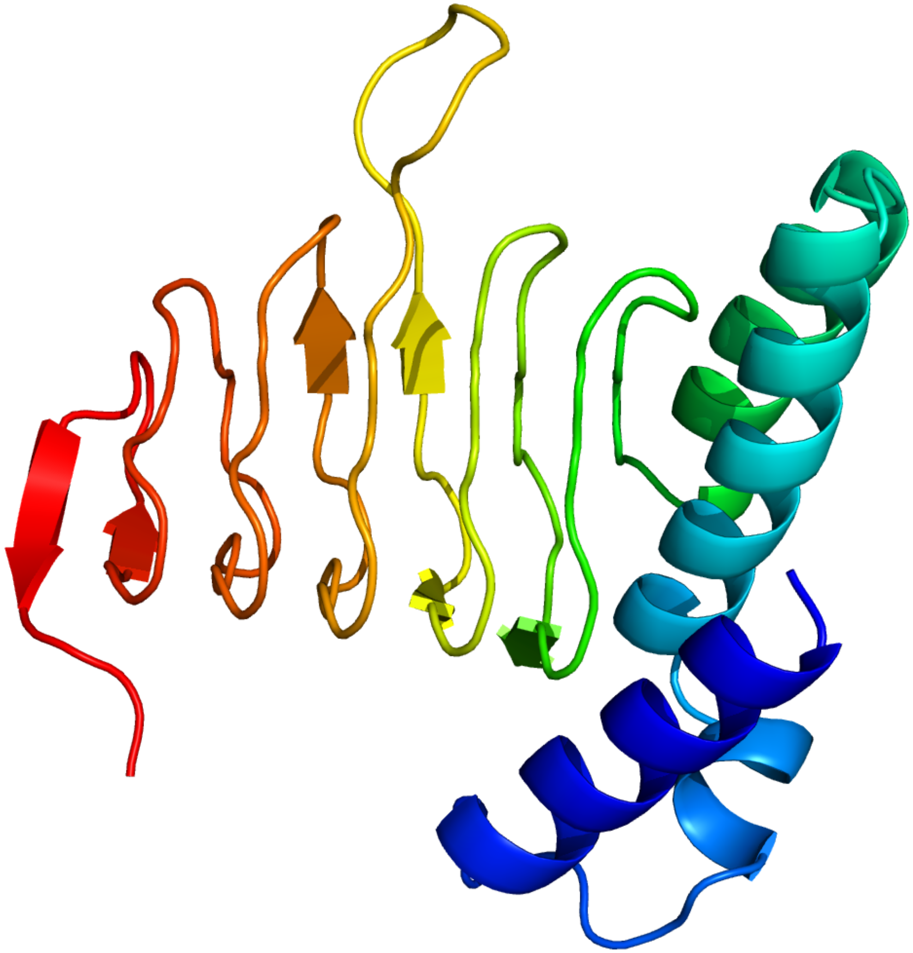

NR1_02812_FMNH2-dependent_alkanesulfonate_monooxygenase

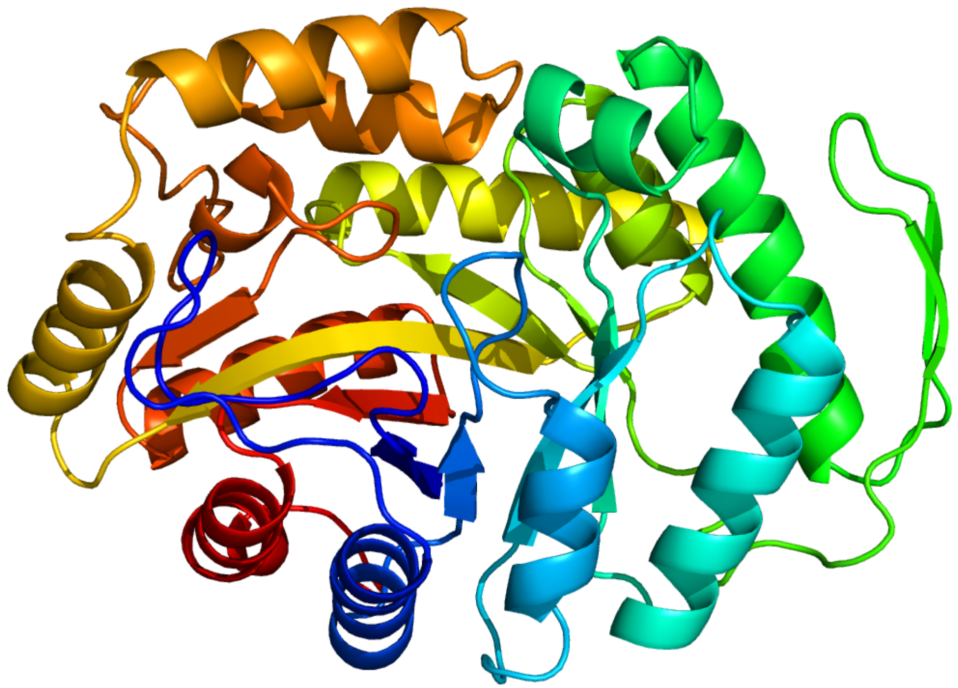

NR1_00449_ABC_transporter_permease 1

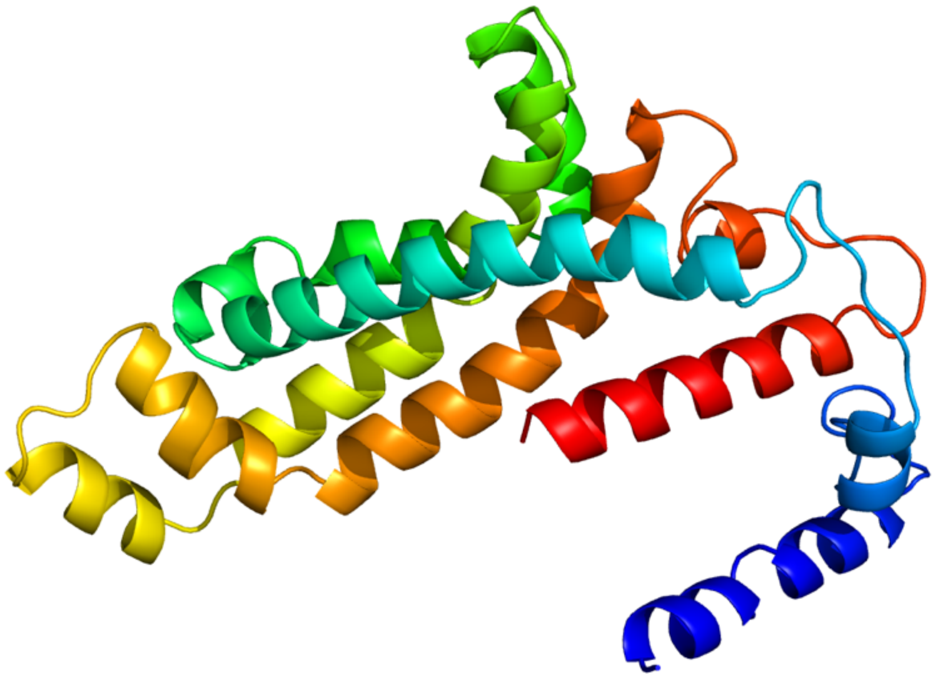

NR1_00775_ABC_transporter_permease2

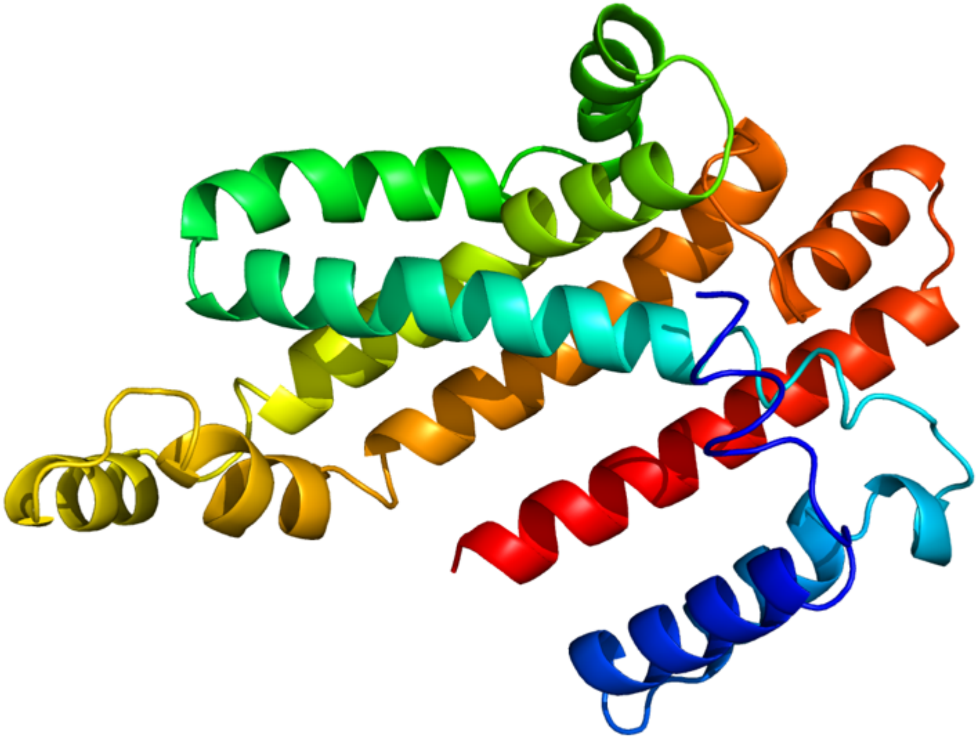

NR1_02813_ABC_transporter_permease 3

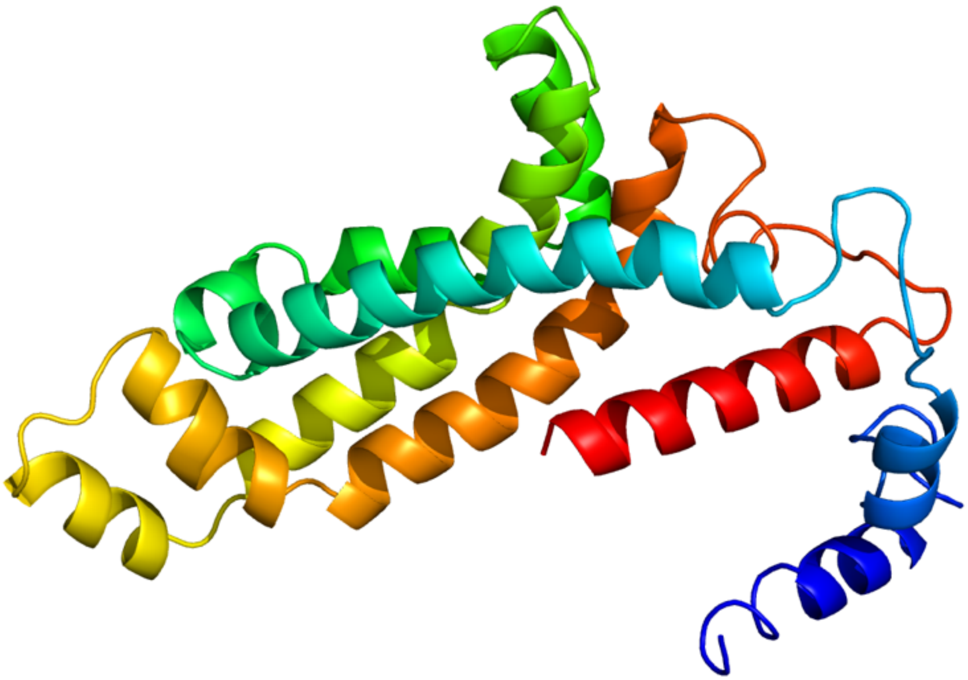

NR1_04665_methionine_gamma-lyase

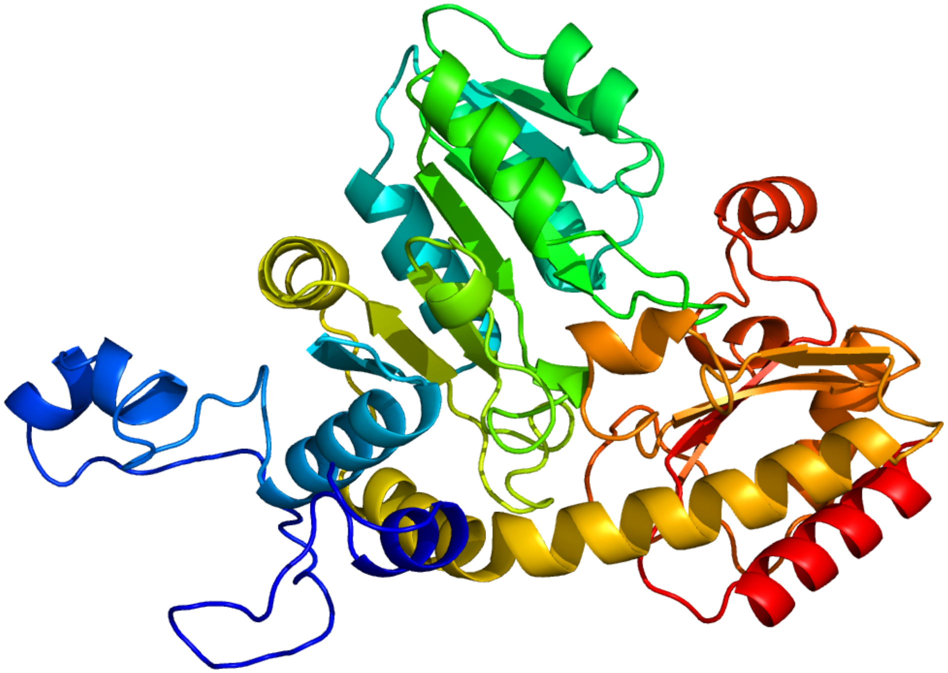

NR1_01119_sulfate_ABC_transporter_substrate-binding_protein

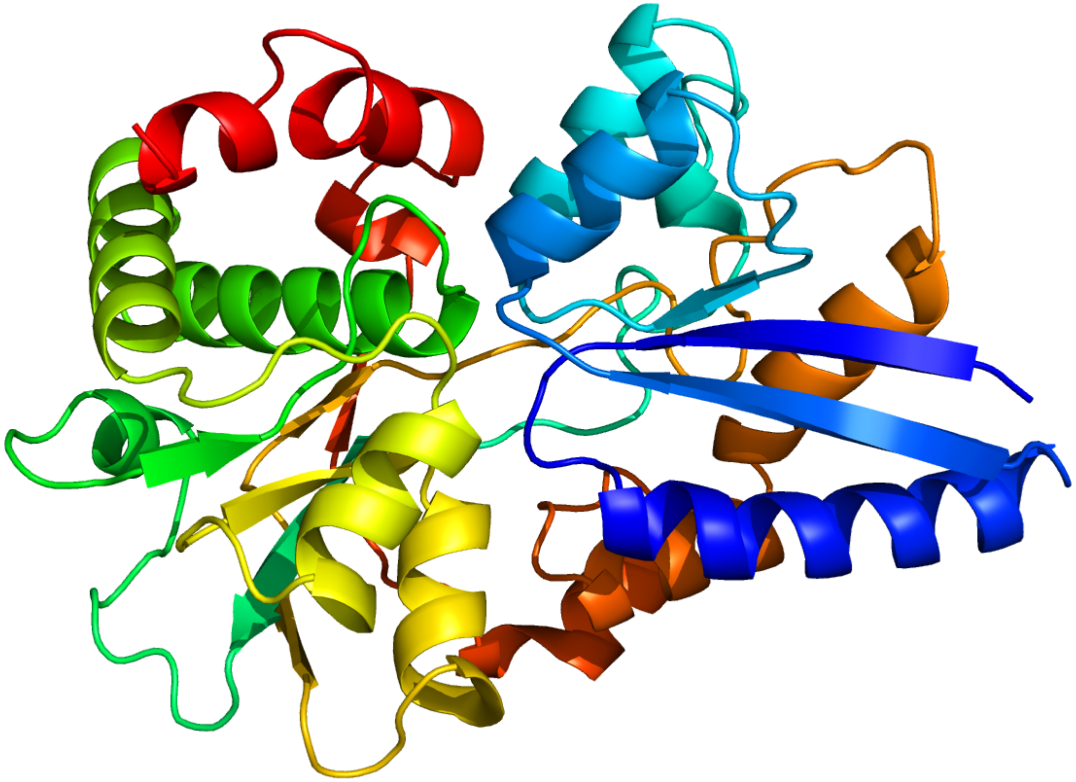

NR1_01903_LLM_class_flavin-dependent_oxidoreductase

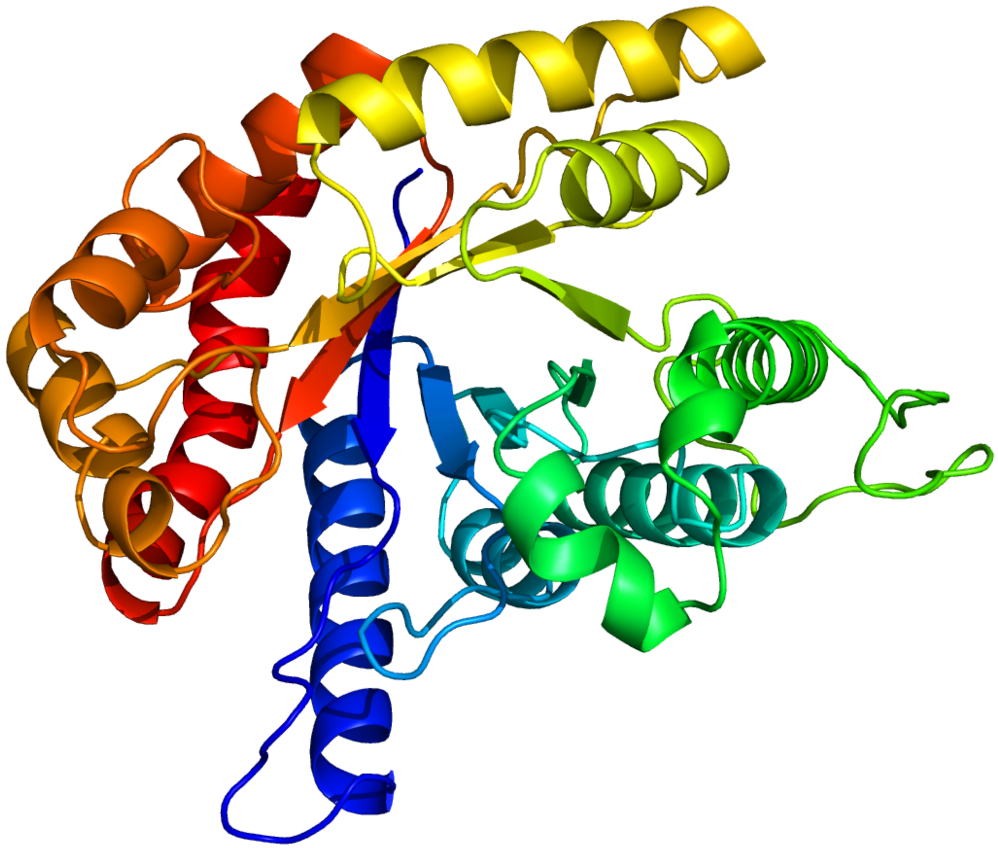

NR1_04757_PepSY_domain-containing_protein

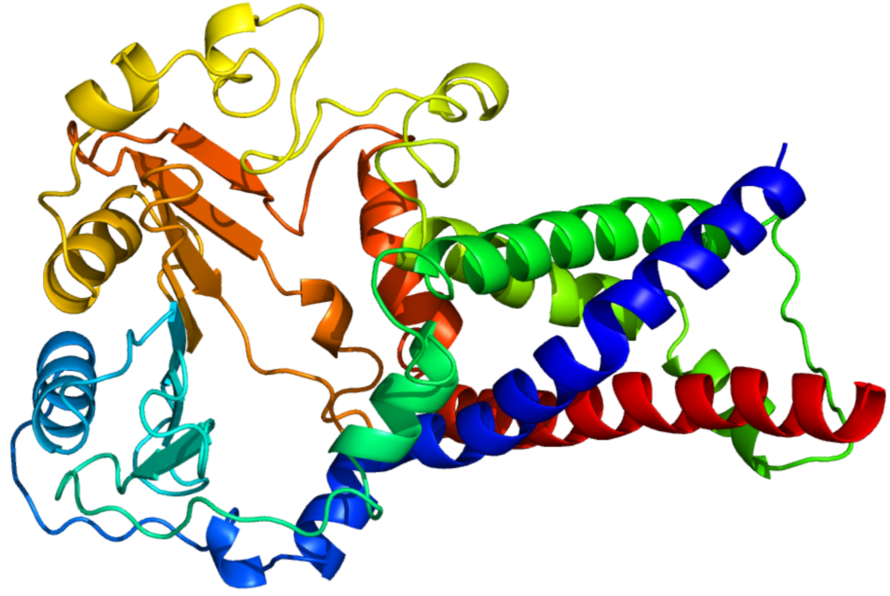

NR1_00820_sulfurtransferase_TusA_family_protein 1

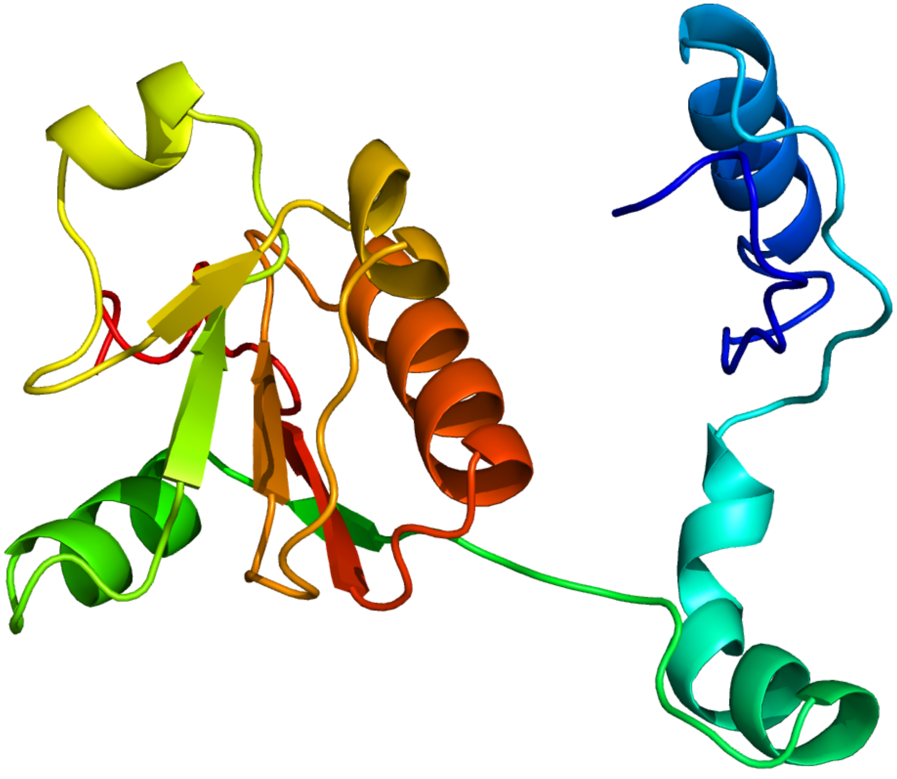

NR1_01603_sulfurtransferase TusA_family_protein 2

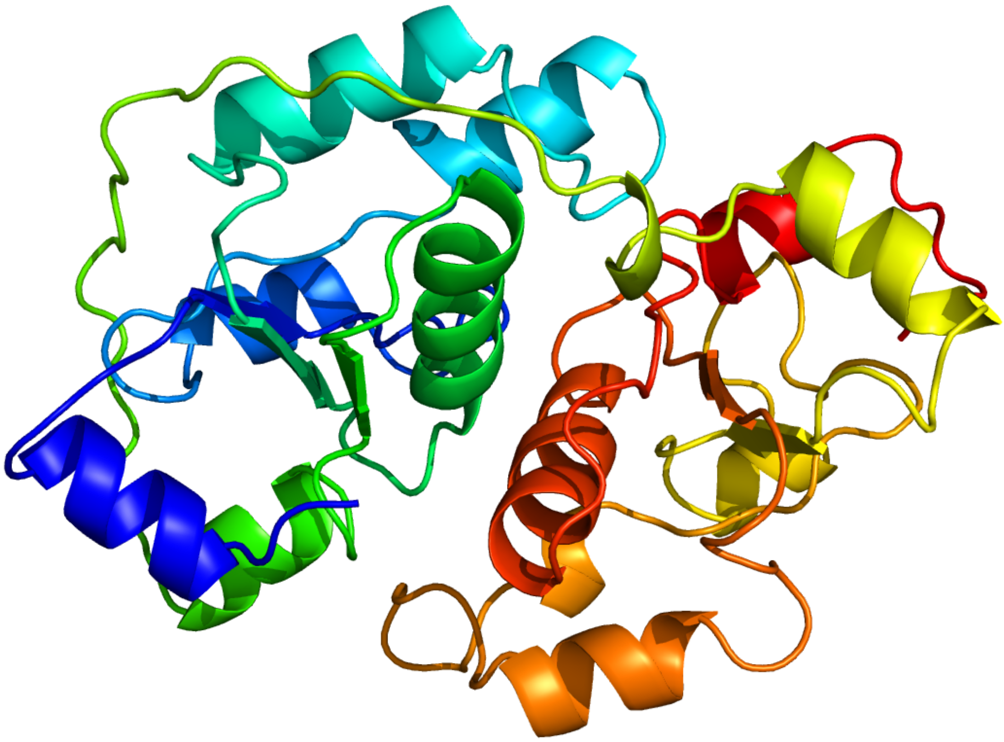

NR1_00823_rhodanese-like_domain-containing_protein 1

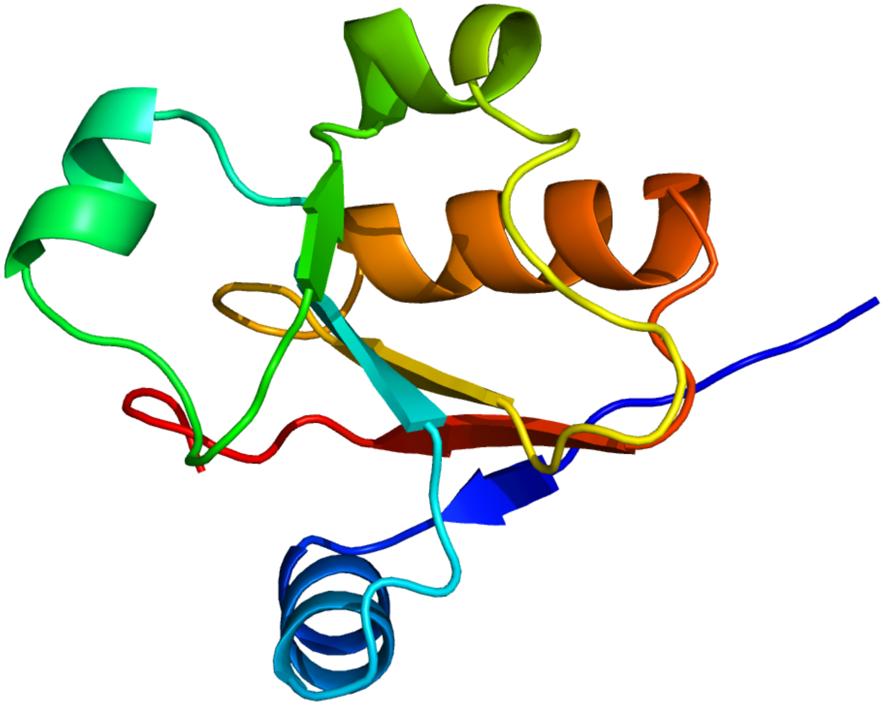

NR1_00824_rhodanese-like_domain-containing_protein 2

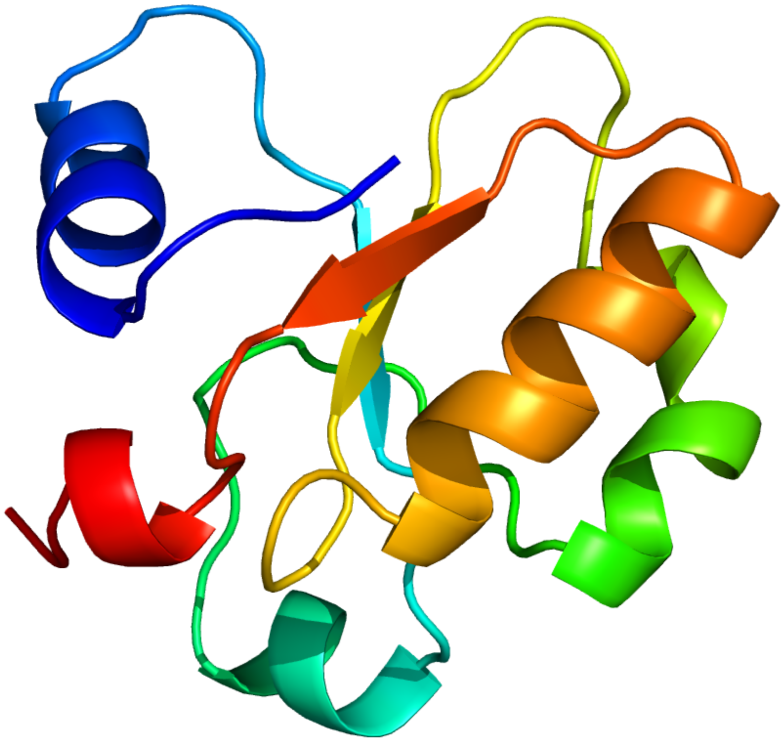

NR1_04202_rhodanese-like_domain-containing_protein 3

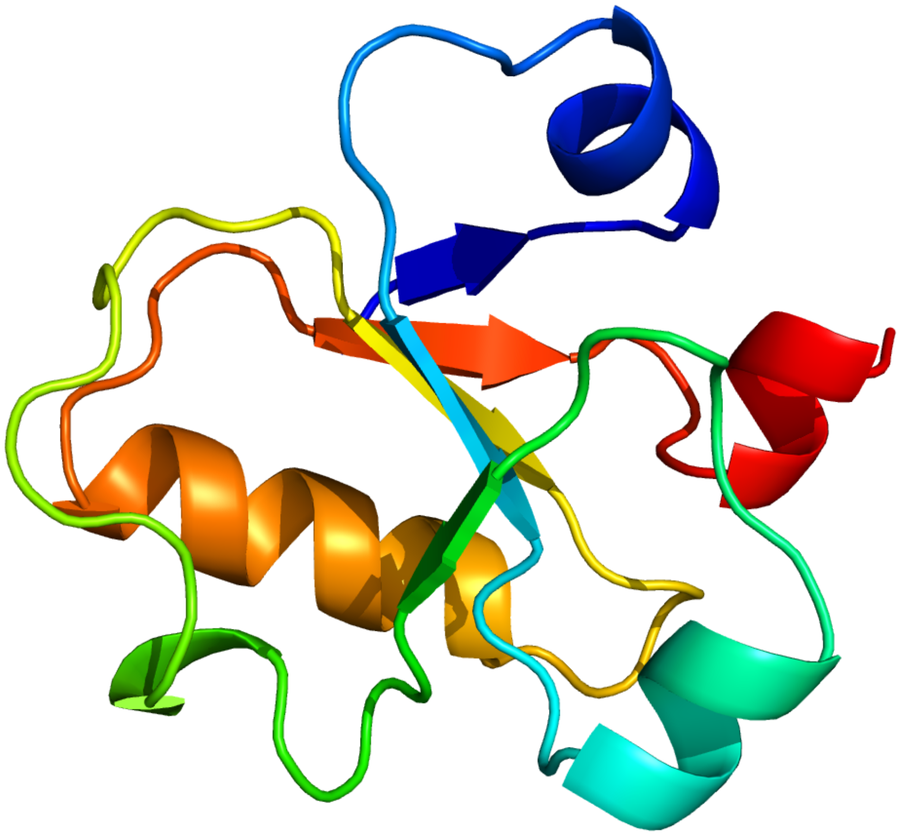

NR1_04736_rhodanese-like_domain-containing_protein 4

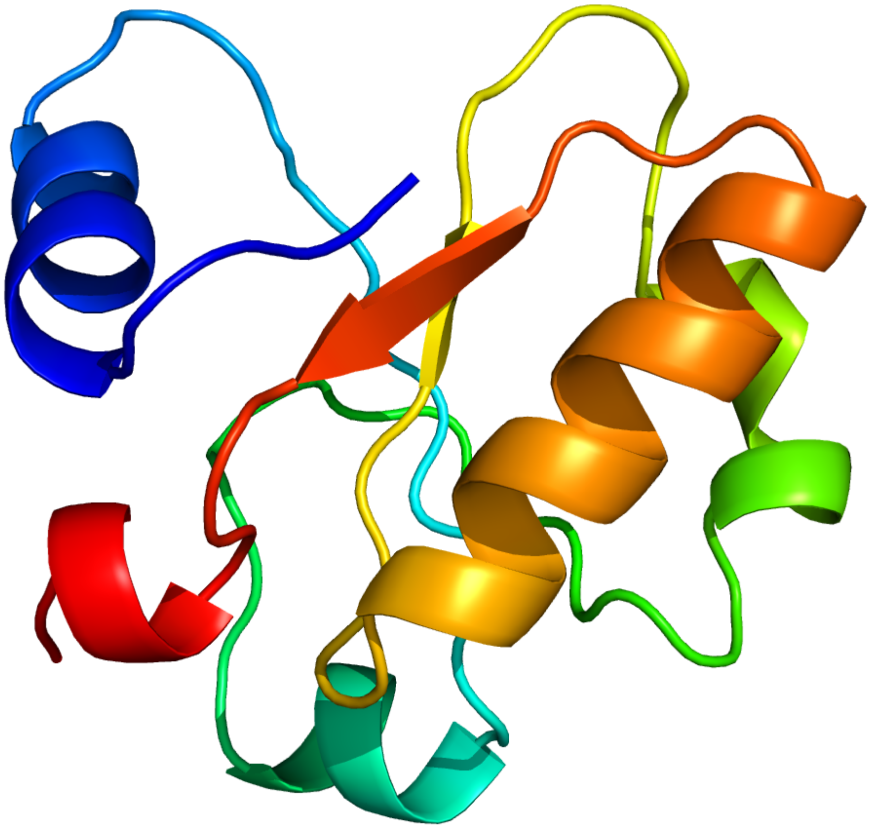

NR1_04366_bifunctional_cystathionine_gamma-lyase_homocysteine_desulfhydrase

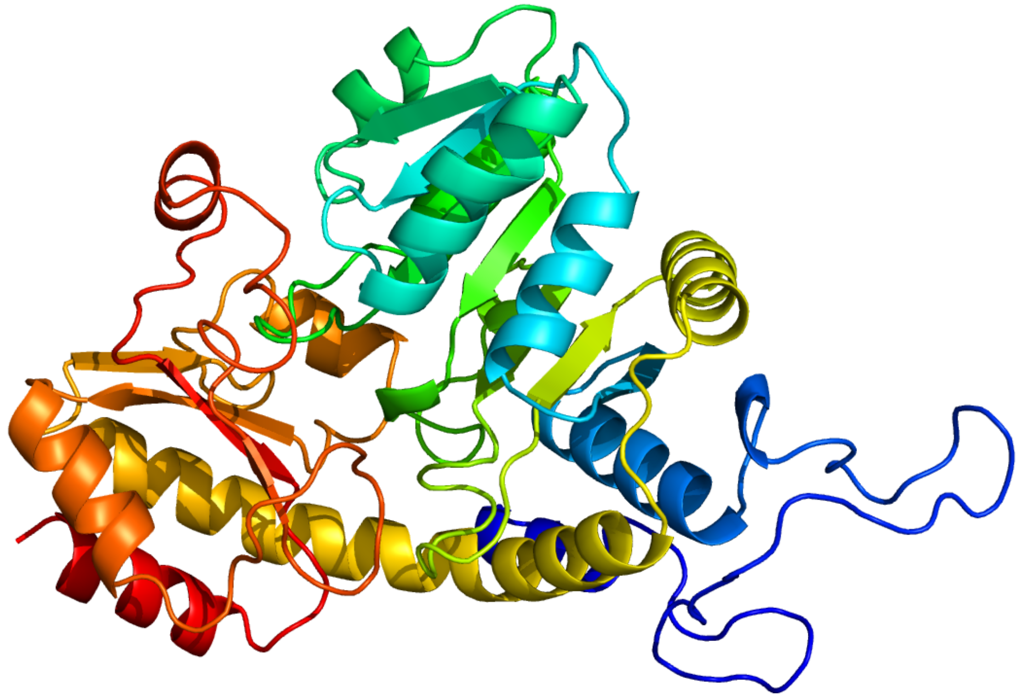

NR1_04761_gamma_carbonic_anhydrase_family_protein

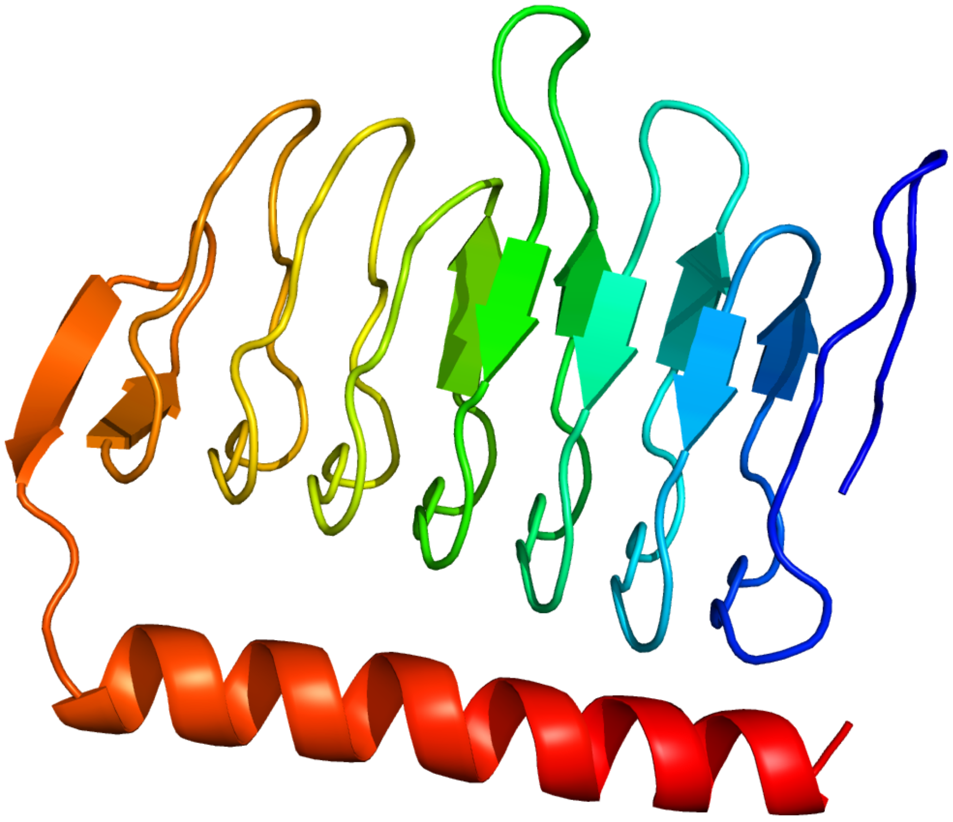

NR1_01447_adenylyl-sulfate_kinase

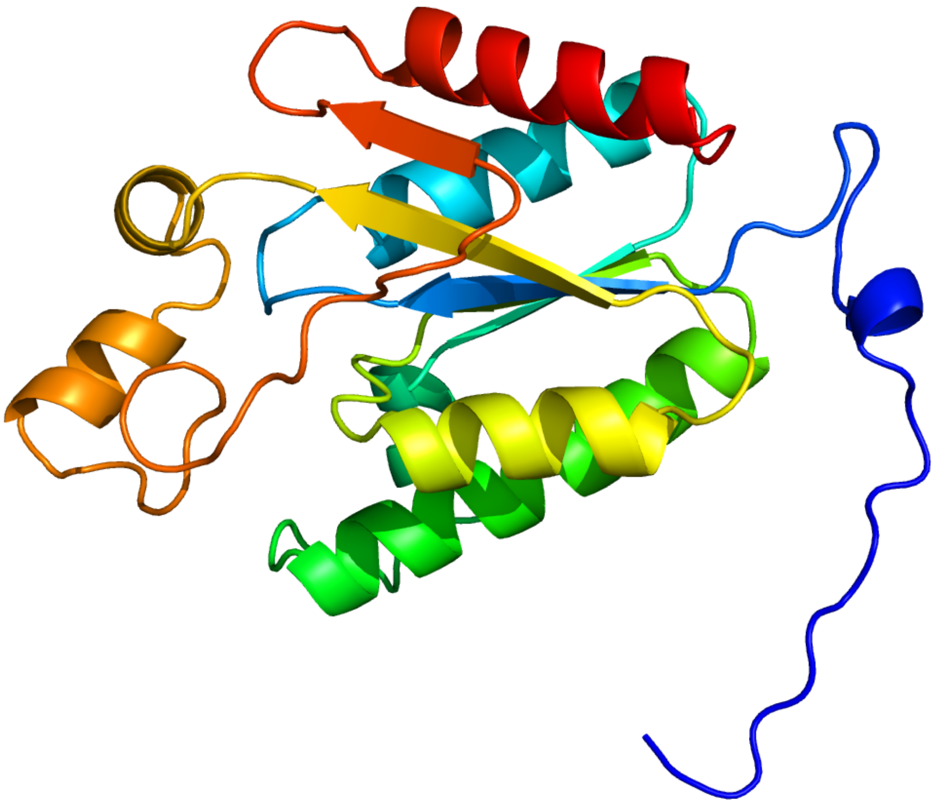

NR1_01446_sulfate_adenylyltransferase

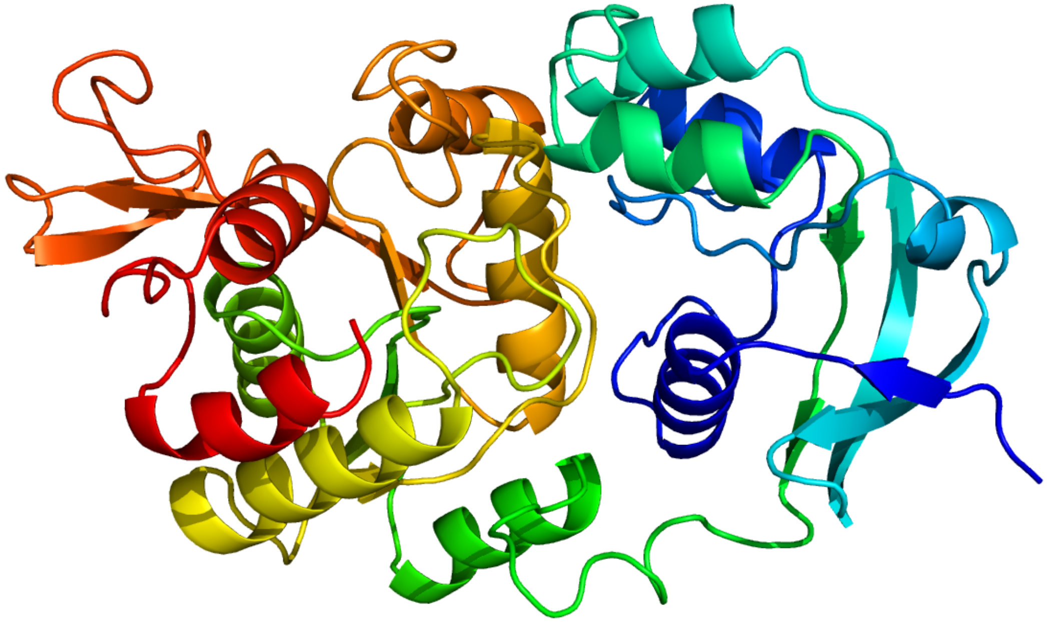

NR1_01445_phosphoadenylyl-sulfate_reductase

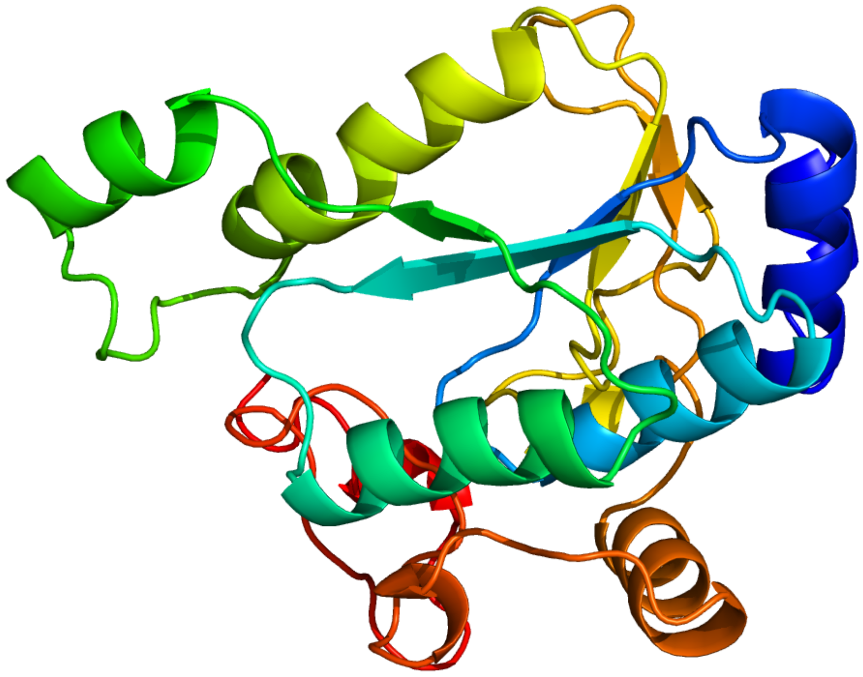

NR1_05337_Fe-S_-binding_protein

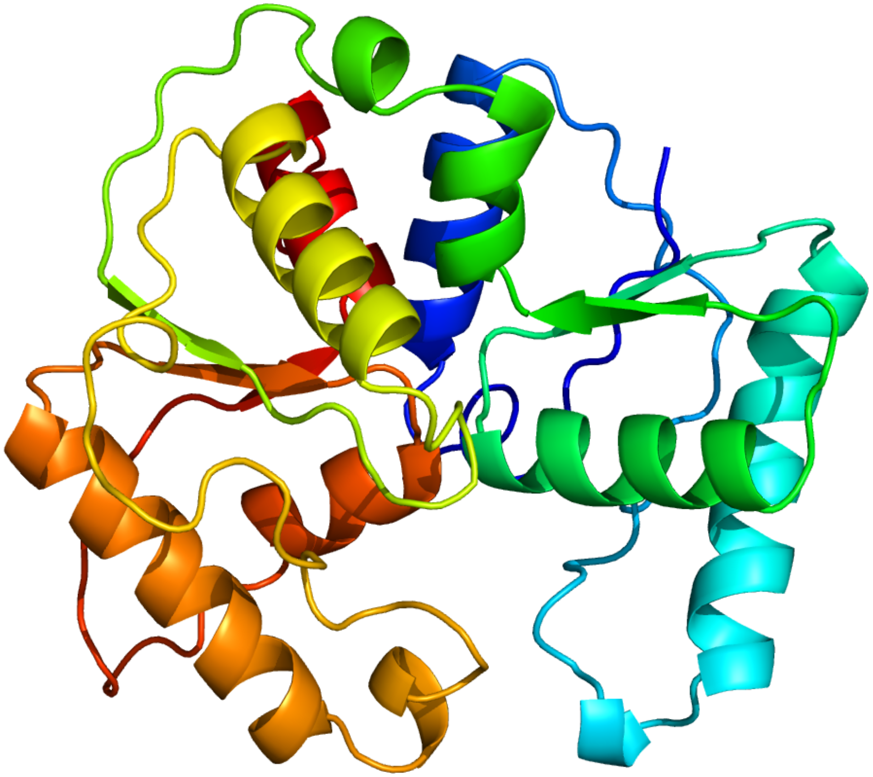

NR1_02814_aliphatic_sulfonate_ABC_transporter_substrate-binding_protein

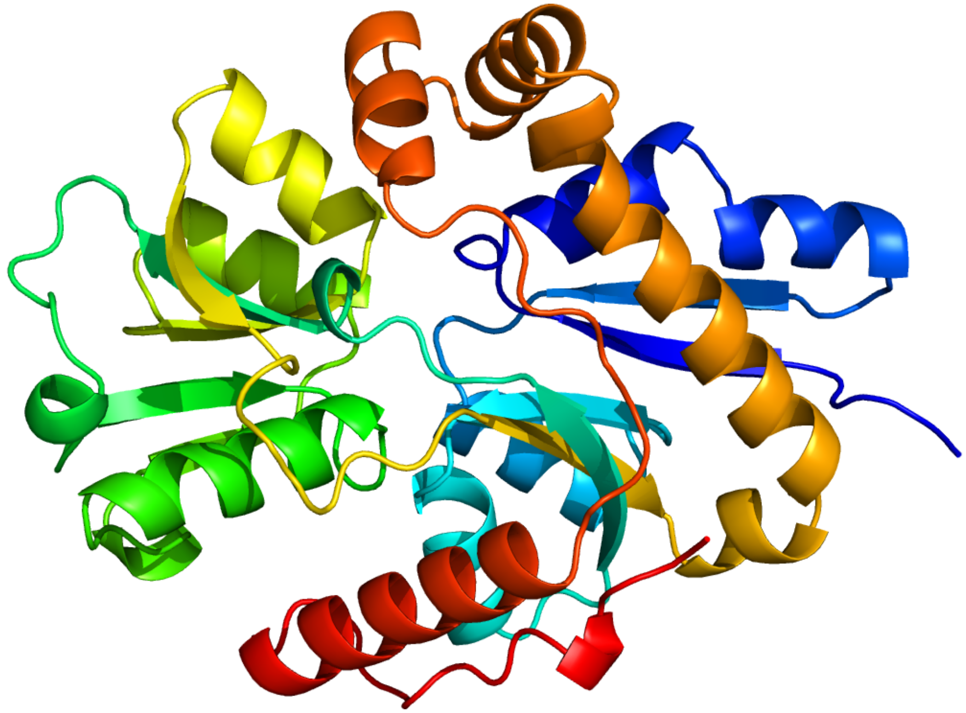

NR1_04617_bifunctional_oligoribonuclease_PAP_phosphatase_NrnA

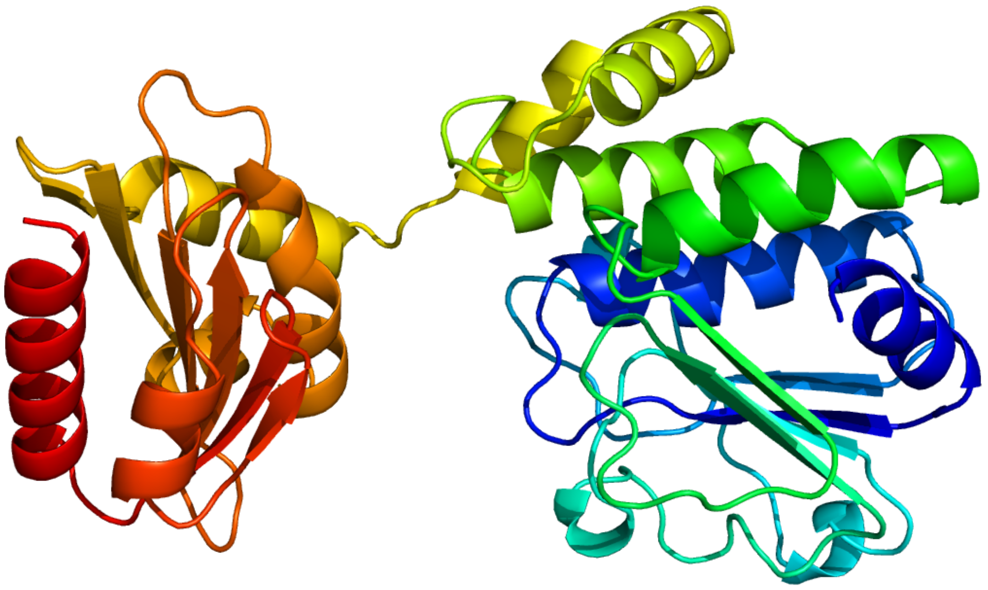

Supplement: Uncited Supplementary Material 1. [file mgen-12-01713-s001.pdf]
